# Nickel-catalyzed divergent Mizoroki–Heck reaction of 1,3-dienes

**DOI:** 10.1038/s41467-023-36237-1

**Published:** 2023-02-06

**Authors:** Wei-Song Zhang, Ding-Wei Ji, Ying Li, Xiang-Xin Zhang, Yong-Kang Mei, Bing-Zhi Chen, Qing-An Chen

**Affiliations:** 1 Dalian Institute of Chemical Physics, Chinese Academy of Sciences, Dalian, 116023 People’s Republic of China; 2 University of Chinese Academy of Sciences, Beijing, 100049 People’s Republic of China

**Keywords:** Homogeneous catalysis, Synthetic chemistry methodology

## Abstract

Developing efficient strategies to realize divergent arylation of dienes has been a long-standing synthetic challenge. Herein, a nickel catalyzed divergent Mizoroki–Heck reaction of 1,3-dienes has been demonstrated through the regulation of ligands and additives. In the presence of Mn/NEt_3_, the Mizoroki–Heck reaction of dienes delivers linear products under Ni(dppe)Cl_2_ catalysis in high regio- and stereoselectivities. With the help of catalytic amount of organoboron and NaF, the use of bulky ligand IPr diverts the selectivity from linear products to branched products. Highly aryl-substituted compounds can be transformed from dispersive Mizoroki–Heck products programmatically. Preliminary experimental studies are carried out to elucidate the role of additives.

## Abstract (web summary)

Developing efficient strategies to realize divergent arylation of dienes has been a longstanding synthetic challenge. Herein, a nickel-catalyzed divergent Mizoroki–Heck reaction of 1,3-dienes has been demonstrated through the modification of ligands and additives.

## Introduction

**Fig. 1:**
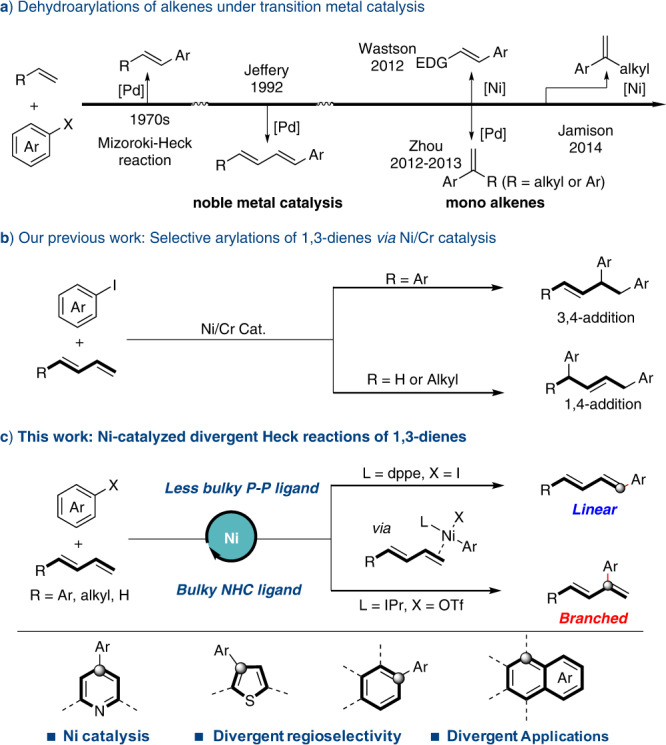
Transition metal-catalyzed arylations of 1,3-dienes. **a** Dehydroarylations of alkenes under transition metal catalysis. **b** Our previous work: Selective arylations of 1,3-dienes via Ni/Cr catalysis. **c** This work: Ni-catalyzed divergent Heck reactions of 1,3-dienes.

The transformation of metal aryl species is one of the most important topics in modern organometallics^1–4^. Taking advantage of the diversity from metal aryl species, a variety of valuable name reactions have been discovered to accomplish different arylations^5–8^. Among them, Mizoroki–Heck reaction has been recognized as an elegant strategy for carboarylations of alkenes^9–13^. However, most of current Mizoroki–Heck reactions focused on the arylation of mono alkenes^14–22^ or using noble palladium catalysts^23^ (Fig. 1a). Therefore, it is of great interest to develop divergent arylations of more challenging molecules under earth abundant metal catalysts.

As extensive feedstocks in nature and industry, the catalytic functionalization of 1,3-dienes is a straightforward strategy to build up the molecular complexity^24–37^. However, compared with that of mono alkenes, the arylation of dienes brings extra challenges in selective control owing to the existence of an additional C = C bond^38–40^. Theoretically, tremendous arylation products (Heck, hydroarylation^41–46^, and diarylation^47–58^ products) with different regio- and stereo-isomers would be expected to be observed through the coupling of aryl halides and dienes. Under Ni/Cr cocatalysis, we previously developed selective diarylation of 1,3-dienes. Besides the desired diarylation product, minor Heck or hydroarylation products were also observed (Fig. 1b)^59^. Similar side-products have also been observed by Koh et al. in their recent work on diarylation of aliphatic 1,3-dienes^60^. Inspired by these precedents^40–59^, we envisioned whether it would be possible to divert the reactivity of Ni−Ar species from diarylation to Heck reaction of dienes. Given the four reactive sites of 1,3-dienes, it will also be possible to obtain divergent regioisomers on Heck reaction.

Here, we develop a divergent Heck reaction of 1,3-dienes under nickel catalysis (Fig. 1c). Programmable transformations from dispersive Heck products have been demonstrated to construct a series of highly aryl-substituted compounds.

## Results

### Reaction optimization

Aryl triflate **1a** and diene **4a** were chosen as model substrates for the investigation of divergent Heck reactions under nickel catalysis (Table 1). In the presence of Mn, a small amount of linear Heck product **5a** (9% yield) was detected using Ni(dppe)Cl_2_ as catalyst precursor accompanied by trace of branched Heck product **6a** (1% yield) and hydroarylated products **7**–**9** (5% yield, entry 1). With the addition of NEt_3_, the yield of product **5a** was increased from 9 to 15% (entry 2). Obvious impact on reactivity and selectivity was observed through the evaluation of other leaving groups (entries 3 and 4). When 4-methoxyphenyl triflate **1a** was replaced by 4-iodoanisole **3a**, 40% yield of **5a** was obtained with excellent selectivity (entries 2 *vs* 4). As the amount of NEt_3_ increased (MeCN/NEt_3_ = 9:1), the yield of **5a** could be improved to 63% in the presence of less manganese powder (entry 5). Given their ability in promoting the circulation of Ni catalyst, some inorganic salts were added to facilitate the reaction (entries 6 and 7)^61^. In terms of reactivity, NaCl emerged as the preferred additive for the formation of **5a** (81% yield). Control experiments further confirmed that nickel was indispensable for this reaction (entry 8). Without the Mn powder, the yield of **5a** decreased significantly (entry 9).

**Table 1:** Optimization of reaction conditions.^a^


Entry	X	Catalyst	T/°C	Base	Additives	Solvent	Yield (%)			
							5a	6a	7 + 8 + 9	10
1	OTf (**1a**)	Ni(dppe)Cl_2_	80	–	Mn	MeCN	9	1	5	–
2	OTf	Ni(dppe)Cl_2_	80	NEt_3_	Mn	MeCN	15	1	6	–
3	Br (**2a**)	Ni(dppe)Cl_2_	80	NEt_3_	Mn	MeCN	5	–	5	–
4	I (**3a**)	Ni(dppe)Cl_2_	80	NEt_3_	Mn	MeCN	40	–	–	–
5	I	Ni(dppe)Cl_2_	80	NEt_3_^b^	Mn^c^	MeCN	63	–	–	–
6	I	Ni(dppe)Cl_2_	80	NEt_3_^b^	Mn/LiCl^d^	MeCN	50	–	–	–
7	I	Ni(dppe)Cl_2_	80	NEt_3_^b^	Mn/NaCl^d^	MeCN	81	–	–	–
8	I	–	80	NEt_3_^b^	Mn/NaCl^d^	MeCN	–	–	–	–
9	I	Ni(cod)_2_/dppe	80	NEt_3_^b^	NaCl^e^	MeCN	16	–	–	–
10	OTf	Ni(cod)_2_/dppe	80	NEt_3_	–	MeCN	9	1	7	–
11	OTf	Ni(cod)_2_/dppe	80	Cs_2_CO_3_	–	MeCN	–	3	–	–
12	OTf	Ni(cod)_2_/**L1**	40	Cs_2_CO_3_	–	MeCN	–	7	–	<1
13	OTf	Ni(cod)_2_/**L1**	40	Cs_2_CO_3_	B_2_pin_2_^f^	MeCN	<1	31	–	–
14	OTf	Ni(cod)_2_/**L1**	40	Cs_2_CO_3_	B_2_pin_2_^f^	1,4-dioxane	4	23	<1	–
15	OTf	Ni(cod)_2_/**L1**	40	Cs_2_CO_3_	B_2_pin_2_^f^	DMF	–	31	–	<1
16	OTf	Ni(cod)_2_/**L1**	40	KOEt	B_2_pin_2_^f^	DMF	–	18	12	–
17	OTf	Ni(cod)_2_/**L1**	40	LiOMe	B_2_pin_2_^f^	DMF	2	32	4	3
18	OTf	Ni(cod)_2_/**L1**	40	Cs_2_CO_3_	B_2_pin_2_/KO^*t*^Bu^g^	DMF	–	46	–	–
19	OTf	Ni(cod)_2_/**L1**	40	Cs_2_CO_3_	**B1**/KO^*t*^Bu^g^	DMF	–	66	–	–
20	OTf	Ni(cod)_2_/**L1**	40	Cs_2_CO_3_	**B1**/NaF^h^	DMF/Hexane^*i*^	–	74	–	–

^a^Conditions: **1a–3a** (0.15 mmol), **4a** (0.10 mmol), [Ni] (10 mol%), ligand (12 mol%), Mn (1.5 equiv.), base (1.5 equiv.), solvent (0.50 mL), 80 °C, 18 h. Yield and selectivity were determined by GC-FID analysis with mesitylene as the internal standard.

^b^NEt_3_ (56 μL), MeCN/NEt_3_ = 9:1 (*v/v*).

^c^Mn (1.0 equiv.).

^d^Mn (1.0 equiv.), MCl (3.0 equiv.).

^e^NaCl (3.0 equiv.).

^f^[B] (20 mol%).

^g^[B]/KO^*t*^Bu (20 mol%).

^h^NaF (20 mol%) was added instead of KO^*t*^Bu.

^i^DMF/Hexane = 3:2 (*v/v*).

When the reaction was conducted with Ni(0) precursor, it exhibited the similar results with that in the presence of Ni(II)/Mn combo (entry 10 vs 1). Replacing NEt_3_ with Cs_2_CO_3_ as base slightly increased the yield of **6a** and inhibited the formation of linear product **5a** (entry 11). With the aid of ligand IPr, milder condition (40 °C) and higher yield (31%) was achieved in the presence of catalytic amount of (Bpin)_2_ (entries 12 and 13). Then, through the screening of various solvents and bases, 1,4-dioxane as solvent brought poor selectivities of **5a** and **6a**. But there was not much difference between DMF and MeCN (entries 13–15). When Cs_2_CO_3_ was replaced by KOEt or LiOMe, undesirable hydroarylation and diarylation products increased distinctly (entries 16 and 17). Notably, the addition of catalytic amount of KO^*t*^Bu could further promote the reaction (46% yield of **6a**) (entry 18). Arylboron (**B1**) showed more effective when compared these results with other organoboron agents (entry 19 and see SI for details). A combination of **B1** and NaF led to an increase in the yield of **6a** (74% yield) in DMF/Hexane (3:2 *v/v*) (entry 20).

### Substrate scope

**Fig. 2:**
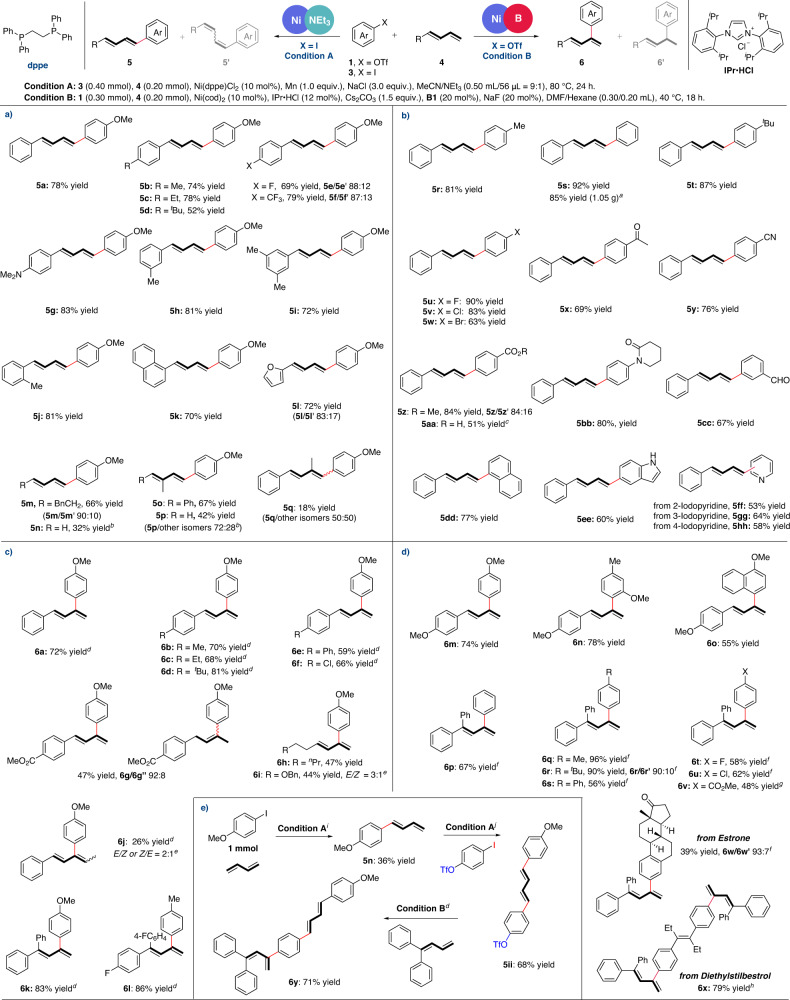
Substrate scope towards divergent Heck reactions. **a** Substrate scopes of dienes for linear products. **b** Substrate scopes of Ar-I for linear products. **c** Substrate scopes of dienes for branched products. **d** Substrate scopes of Ar-OTf for branched products. **e** Cascade Heck reactions. Isolated yields were given in all cases. Selectivities were >20:1 except for some cases with footnotes, which were determined by GC-FID or ^1^H NMR analysis. ^a^iodobenzene (9.0 mmol), **4a** (6.0 mmol); ^b^**3a** (0.20 mmol), 1,3-diene (0.60 mmol); ^c^Isolated yield of two steps (coversion of -CO_2_H to -CO_2_Me); ^d^**1** (0.20 mmol), **4** (0.25 mmol), 4-CNC_6_H_4_Bneop (**B2**, 20 mol%) was used instead of **B1**; ^e^A mixture of *Z*- and *E*-isomers of diene **4** was used; ^f^**1** (0.25 mmol); ^g^**1** (0.20 mmol), **4** (0.25 mmol), KO^*t*^Bu was used instead of NaF; ^h^**1** (0.10 mmol), **4** (0.25 mmol), Ni(cod)_2_ (20 mol%), IPr∙HCl (24 mol%), Cs_2_CO_3_ (3.0 equiv.), **B1** (40 mol%), KO^*t*^Bu (40 mol%); ^i^**3a** (1.0 mmol), 1,3-butadiene (3.0 mmol), MeCN/NEt_3_ (5.0 mL/0.60 mL = 9:1); ^j^Ar-I (0.60 mmol), **5n** (0.40 mmol), MeCN/NEt_3_ (1.0 mL/0.11 mL = 9:1).

With the optimized conditions in hand, we subsequently explored the scope of substrates in this divergent approach (Fig. 2). For linear Heck reaction, the 1,3-dienes bearing electron-donating groups on the aromatic ring, including Me (**5b, 5h-5j**), Et (**5c**), ^*t*^Bu (**5d**), NMe_2_ (**5** **g**), all reacted with 4-iodoanisole smoothly and delivered the corresponding products in moderate to good yields with excellent selectivities. In comparison, electron-deficient substrates showed slightly low stereoselectivities (**5e** and **5f**). Notably, naphthyl and furyl substituted 1,3-dienes were also compatible with this transformation, leading to 1,4-diaryldienes in 70 and 72% yields, respectively. In addition, when using aliphatic 1,3-dienes (*E/Z* = 1/1) for the coupling with 4-iodoanisole, product **5m** could be successfully afforded in 66% yield and 9:1 stereoselectivity. Satisfyingly, the reaction also proceeded with naked 1,3-butadiene, which may provide an alternative route for the synthesis of 1-arylbutadienes and avoid the huge waste during traditional Wittig reactions. Not only the mono-substituted dienes, but also the **5o** could be obtained from 1,2-disubstituted diene with high yield and selectivity under the standard condition. Lower reactivities and selectivities were obtained for isoprene and 1,3-disubstituted diene (**5p** and **5q**).

Then the iodoarene substrates were explored (Fig. 2b). A variety of iodoarenes bearing electron-withdrawing and electron-donating groups all underwent the desired cross-coupling smoothly. Generally, the corresponding linear Heck products were obtained with good to excellent yields, high stereoselectivities and exquisite regioselectivities (**5r**−**5** **y** and **5aa**−**5hh**, 60−92% yields). However, the coupling of methyl 4-iodobenzoate with 1,3-diene displayed moderate stereoselectivity (**5z**/**5z′ =** 84/16). It should be noted that the bromo group, which could offer useful handles for further synthetic manipulations, was well compatible under the current conditions (**5w**). Additionally, substrates bearing sensitive groups, such as carboxylic acid, aldehyde, unprotected indole and pyridines (**5aa**, **5ee**, and **5ff-5hh**), were all applicable, highlighting the general tolerance of this nickel catalysis. To illustrate the practical utility of this strategy, a scale-up experiment under the standard conditions was performed to afford the corresponding product **5s** in 1.05 g with 85% yield.

Next, we sought out to assess the substrate scope towards branched arylation. In order to facilitate the separation of products and additives, 4-cyanophenylboron **B2** was used instead of **B1** in some cases. As illustrated in Fig. 2c, dienes with either electron-donating or electron-withdrawing substituents at the 4-position of phenyl ring all reacted with diene **4a** in good reactivities and excellent regio- and stereo-selectivities (**6a**−**6f**, 59–81% yields). In addition, 1,3-diene possessing ester group was well tolerated in this case, but minor hydroarylation product could be observed (**6g/6g″**  = 92:8). Similar to linear Heck reactions, aliphatic-1,3-dienes was also well applicable to this strategy (**6h** and **6i**). Depending on the *E/Z* ratio of diene substrates, the stereoselectivities of aliphatic products would be maintained. Although the less impressive result (**6j**) was obtained for 1,4-disubstituted diene, 1,1′-diaryl-1,3-dienes showed good reactivities and regioselectivities for the current transformations and were not affected by steric hindrance of another aromatic ring (83% yield for **6k** and 86% yield for **6l**).

Meanwhile, the scope of aryl triflates in coupling with dienes was investigated under the Ni/IPr catalysis (Fig. 2d). Generally, the reaction exhibited no obvious loss in both reactivities and selectivities when electron-rich or electron-deficient were subjected (**6** **m**, **6n, 6o** and **6t-6v**). Good yields and selectivities could also be achieved even when ortho-substituted aryl triflates were employed, regardless of their steric hindrance (**6n** and **6o**). In addition, substrates derived from estrone and diethylstilbestrol could also react smoothly to obtain corresponding branched products (**6w** and **6x**).

Consecutive Heck reaction could even proceed from common material to deliver a hybrid of linear and branched diene (Fig. 2e). Under condition A, linear diene **5n** could be synthesized from 4-Iodoanisole and 1,3-butadiene with 36% yield in 1 mmol scale. Using bifunctional coupling reagent 4-iodophenyl triflate, nickel catalysis could selectively cleave C−I bond rather than C−O bond in condition A to yield (*E,E*)−1,4-diarydiene **5ii**. Without the influence of the two C=C bonds of **5ii**, polyene **6** **y** bearing linear and branched diene motifs was constructed in 71% yield under condition B.

### Transformations

**Fig. 3:**
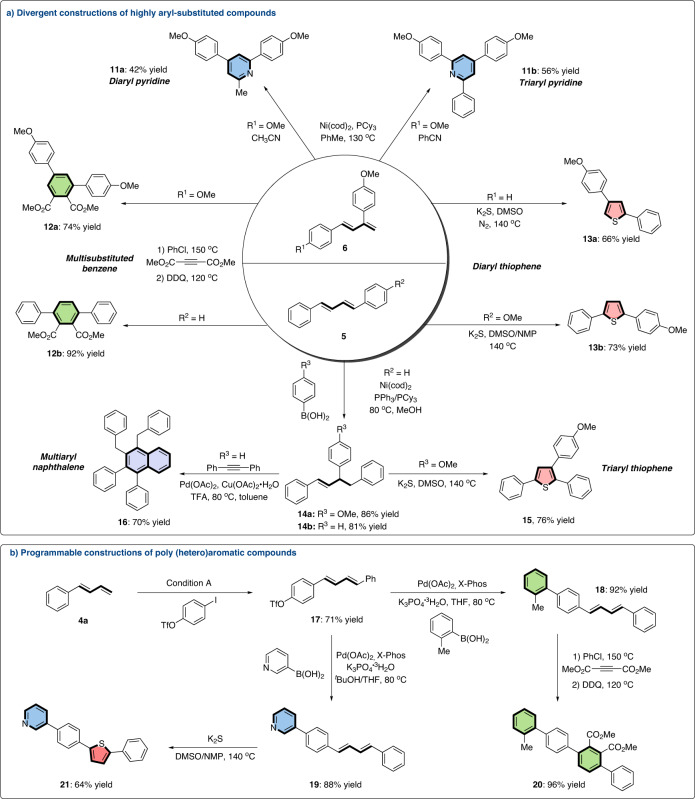
Divergent and programmable synthetic transformations of dienes. **a** Divergent constructions of highly aryl-substituted compounds. **b** Programmable constructions of poly (hetero)aromatic compounds.

To demonstrate the synthetic utility of the diarylation products, concise syntheses of highly aryl-substituted compounds were performed from linear and branched dienes **5** and **6** (Fig. 3a). Through a Ni-catalyzed dehydrogenative [4 + 2] cycloaddition between nitrile and branched arylated product **6** **m**, diaryl- and triaryl pyridines (**11a** and **11b**) could be accessed with 42 and 56% yields respectively^62^. Multisubstituted benzenes (**12a** and **12b**) were easily synthesized from **5** **s** and **6** **m** in 92 and 74% yields via Diels-Alder reaction and oxidation in one pot^63^. Meanwhile, diaryl thiophenes (**13a** and **13b**), which were potential pesticides, could also be accessed by an oxidative cyclization from inorganic sulfurating reagents K_2_S and diene **5a** or **6a**. Under nickel catalysis^64^, the hydroarylations of linear diene **5** **s** smoothly delivered diarylated products **14a** and **14b** which could be further converted to polyaryl thiophene **15** and naphthalene **16**^64,65^.

On the other hand, the linear poly (hetero)aromatic compounds, as important components of quasi-one-dimensional conducting polymers^66^, could be constructed programmatically from simple starting materials. The chemoselective coupling of bifunctional reagent 4-iodophenyl triflate with 1-phenyl-1,3-diene yielded the desired aryl triflate **17**. Subsequent Suzuki reactions of **17** with different (hetero)arylboronic acid gave ploy (hetero)aryl dienes **18** and **19**^67^. Through oxidative cyclizations, linear poly (hetero)aromatic compounds **20** and **21** were finally obtained in 96 and 64% yields (Fig. 3b)^63,64^.

### Mechanistic investigations

**Fig. 4:**
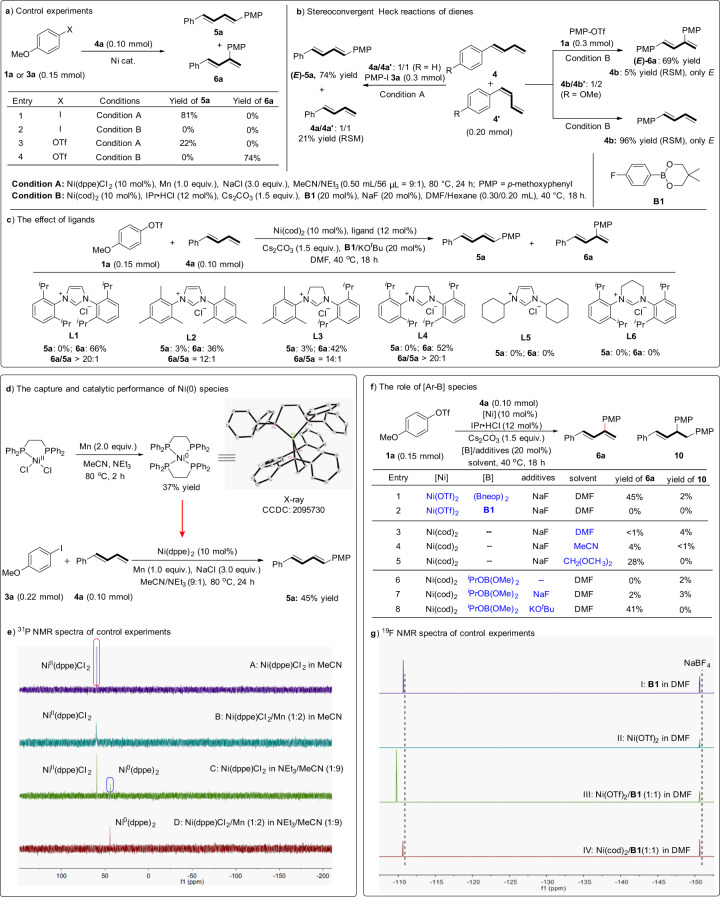
Mechanistic studies of Heck reactions. **a** Control experiments. **b** Stereoconvergent Heck reactions of dienes. **c** The effect of ligands. **d** The capture and catalytic performance of Ni(0) species. **e** ^31^P NMR spectra of control experiments. **f** The role of [Ar-B] species. **g** ^19^F NMR spectra of control experiments.

Corresponding control studies were carried out to shed light on the mechanistic insights. The chemoselectivities and regioselectivities of Ni catalysis on two leaving groups (I or OTf) were investigated under standard conditions A and B. Although good yield of **5a** was obtained for PMP-I **3a** in condition A, no expected product **5a** or **6a** was observed under condition B (Fig. 4a, entries 1 and 2). For aryl triflate **1a**, less Heck product **5a** was found in condition A than that of PMP-I **3a** (entries 3 *vs* 1). Moreover, branched product **6a** became the only product instead of **5a** in condition B (entries 4 *vs* 3). These control experiments suggest that, the reactivity of linear arylation is positively correlated with the leaving ability of substituted group (I and OTf). While for branched Heck reaction, this reaction is chemospecific to the leaving group. In addition, regioselectivity is regulated by ligand or additives other than leaving groups.

When submitting a *Z*/*E* mixture of diene isomers (**4a/4a′** = 1/1 and **4b/4b′** = 1/2) to the Heck reaction conditions, only *E* products **5a** and **6a** were deserved and the yields were similar to that of results using (*E*)-substrates (Fig. 4b). Through the analysis of the recovered starting materials, the *E/Z* ratio of diene **4a** did not change significantly under condition A, but only (*E*)-diene **4b** was recovered in 5% yield. In the absence of triflate **1a**, only (*E)*-**4b** was recovered under condition B. The strong stereoconvergent effect implies that an interconversion between *η*^1^-allyl nickel and *η*^3^-allyl nickel species might be involved in the catalytic cycle of linear Heck reaction. And it indicates a fast isomerization from *cis*- to *trans*-diene **4** could occur in branched Heck reaction. From the evaluation of NHC ligands (Fig. 4c), the less bulky ligand IMes displayed lower regioselectivity and reactivity (**L2** *vs* **L1**). But as the steric hindrance increases on one side of the ligand, the selectivity and yield would be slightly promoted (**L3** *vs* **L2**). When **L1** was replaced by **L4** which shows the only difference in donor properties, the yield of **6a** decreased a little bit but the selectivity could still be maintained. It indicated that the steric hindrance of ligand has a more significant effect on the regioselectivity than electronic effect. There was no obvious reaction with the addition of **L5** or **L6** and it seems that the skeleton of ligand has a decisive influence on the reactivity. These conclusions have been supported by previous theoretical study^68^. Possibly due to energy difference of insertion transition states^69^, the use of phosphine ligands and bulky NHC ligands displayed the opposite selectivities.

Next, further studies have been performed to capture possible intermediates and verify the effects of additives in this divergent Heck reaction. For linear Heck reaction of diene, treating stoichiometric Ni^II^(dppe)Cl_2_ with Mn powder in MeCN/NEt_3_ successfully provided Ni^0^(dppe)_2_ which structure was confirmed through X-ray analysis (Fig. 4d). With the aid of this prepared Ni^0^ precursor Ni(dppe)_2_, the Heck reaction coupling between **3a** and **4a** could also proceed smoothly to deliver product **5a** in 45% yield. Based on these observations, the linear Heck reaction might be initiated by the formation Ni(0) species in situ. Meanwhile, NEt_3_ has shown important effect on enhancing the reactivity of current Ni-catalyzed linear Heck reaction (Table 1). Thus, ^31^P NMR spectra of control experiments were then performed to probe the role of NEt_3_. As depicted in Fig. 4e, the chemical shift of Ni(dppe)Cl_2_ in MeCN was found at 59.44 ppm. No new signal or chemical shift was found after the addition of Mn to the Ni(dppe)Cl_2_ solution, indicating the difficulty of the Ni(II) precursor to be reduced to Ni(0) species by Mn powder alone in this case. However, a new peak at 43.87 ppm, which should be assigned to Ni^0^(dppe)_2_ complex, could be successfully detected when NEt_3_ was loaded. Therefore, this unexpected reduction indicates that NEt_3_ not only served as a base in this transformation, but also may facilitate reduction process of Ni^II^ precursor to Ni(0) species.

For branched Heck reaction, organoboron also plays a crucial role on reactivity. Diboron compounds have been previously suggested to reduce Ni^II^ to Ni^0^^51,54^. Using Ni(OTf)_2_ instead of Ni(cod)_2_ as catalyst precursor, aryl boron (**B1**) did not show the same positive effect as diboron (B_2_neop_2_) (Fig. 4f, entries 1 *vs* 2). It suggests that Ni^II^ could not be reduced effectively by aryl boron. In the absence of organoboron additive, chelated solvent dimethoxy methane (DMM) favored the formation of **6a** than DMF and MeCN (Fig. 4f, entries 3–5). Given the structural similarity between boron ester and DMM, it indicates that boron ester probably promote reaction through chelation on Ni catalyst. To figure out the role of aryl group on additive **B1**, nonreductive ^*i*^PrOB(OMe)_2_ was used instead of **B1** for branched dehydroarylation. The desired reaction occurred in the presence of the catalytic amount of NaF or KO^*t*^Bu and the latter gave a higher yield (Fig. 4f, entries 6–8)^70^. These results indicate that ionic ployalkoxyborate better promotes the branched Heck reaction than neutral organoborate. It also rules out the reductant role of arylboron **B1** for Ni catalyst. Meanwhile, ^19^F NMR studies of control experiments were performed to further confirm the chelation effect of borate (Fig. 4g). The addition of Ni(OTf)_2_ to **B1** in DMF led a clear change from −110.67 ppm to −109.76 ppm in ^19^F NMR spectra (Fig. 4g, I *vs* III). This downfield shift probably resulted from a decrease of electron density on fluorine atom of **B1**. However, there was no obvious change on chemical shift of ^19^F NMR signal by mixing Ni(cod)_2_ with **B1** in DMF. It suggests anionic organoborate preferentially coordinate with cationic Ni^II^ species rather than neutral Ni^0^ complex in the catalytic cycle.

**Fig. 5:**
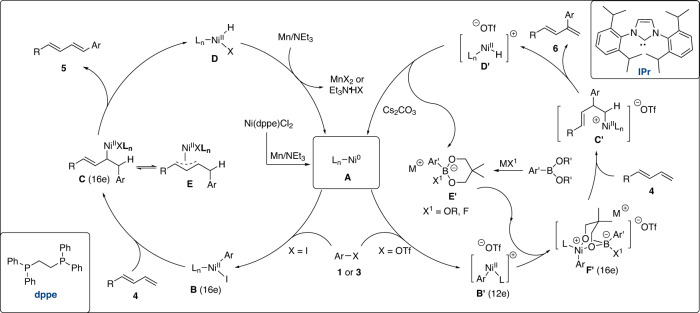
Proposed mechanism for divergent arylation via nickel catalysis. Through the regulation of dppe ligand, linear Heck product **5** is obtained in presence of Mn and NEt_3_. With the aid of bulky ligand IPr, the selectivity of reaction is switched to branched product **6** in presence of alkoxyborate.

On the basis of the above observations, a plausible mechanism for the divergent arylation via nickel catalysis was proposed (Fig. 5). For linear Heck reaction, catalyst precursor Ni(dppe)Cl_2_ is reduced to give active Ni(0) species **A** in situ by Mn powder and NEt_3_. Subsequently, an oxidative addition of iodoarene **3** with **A** yields aryl-Ni(II) complex **B**. Then, migratory insertion between complex **B** and 1,3-diene **4** gives allyl-nickel complex **C** or its ƞ^3^ coordinated form **E**. Next, final *β*-H elimination from complex **C** affords linear Heck product **5** and Ni^II^ species **D**. With the aid of Mn and NEt_3_, Ni^0^ catalyst **A** is regenerated from Ni^II^ species **D** to complete the catalytic cycle.

For branched Heck reaction promoted by IPr ligand (Fig. 5, right), cationic Ni(II) species **B′** is initially obtained from an oxidative addition of aryl triflate **1** with **A**. Meanwhile, the reaction of organoboron with base (NaF or KO^*t*^Bu) produces alkoxyborate **E**. Then a chelation of cationic Ni^II^ species **B′** with anionic organoborate **E**′ forms Ni(II) complex **F′**. A subsequent migratory insertion of diene **4** into Ni-Ar **F**′ yields an alkyl-nickel (II) species **C′**. The steric hindrance between bulky ligand IPr and allylic motif impedes the formation of isomer **C**. Afterwards, product **6** and Ni^II^ species **D′** can be obtained through *β*-H elimination from complex **C′**. With the help of Cs_2_CO_3_, a final reductive elimination from allyl-Ni^III^ species **D′** regenerates the Ni^0^ catalyst **A** and alkoxyborate **E′** for next catalytic cycle. It will be more challenging to realize the 1- and 2-arylation of dienes which probably requires special substrate or catalyst design.

## Discussion

In this work, a divergent Heck reaction of 1,3-dienes with Ar-X (X = I or OTf) is developed under Ni catalysis. Through the regulation of dppe ligand, linear Heck products are successfully obtained in presence Mn and NEt_3_. The selectivity of reaction is efficiently switched to branched products by the combination of Ni(cod)_2_/IPr and alkoxyborate. A series of highly aryl-substituted compounds are constructed programmatically and concisely from our protocol. Besides, preliminary mechanistic studies provide clues for possible catalytic pathways and the role of additives. Further studies and application on this divergent arylation of dienes are underway in our laboratory.

## Methods

### General procedure for Ni-catalyzed linear Heck reaction of 1,3-dienes

In a glove box, a sealed tube was charged with iodoarene **3** (0.40 mmol), Ni(dppe)Cl_2_ (0.02 mmol, 10 mol%), Mn (0.20 mmol, 1.0 equiv.), NaCl (0.60 mmol, 3.0 equiv.), 1,3-diene **4** (0.20 mmol), MeCN (0.5 mL), NEt_3_ (56 μL) at room temperature. The reaction tube was sealed with a Teflon screw cap, removed from the glove box. Then, the reaction mixture was stirred at 80 °C for 24 h. The ratio of **5** and **5’** was determined by GC-FID analysis. And the crude reaction mixture was purified by column chromatography on silica gel or recrystallization using petroleum ether and dichloromethane to afford the corresponding product **5**.

### General procedure for Ni-catalyzed branched Heck reaction of 1,3-dienes

In a glove box, Ni(cod)_2_ (0.02 mmol, 10 mol%), IPr·HCl (0.024 mmol, 12 mol%), Cs_2_CO_3_ (0.30 mmol, 1.5 equiv.), and **B1**/NaF (0.04 mmol, 20 mol%) were added to DMF (0.30 mL) in sequence and stirred at room temperature for 30 min. Then, the mixture of aryl triflate **1** (0.20 mmol), 1,3-diene **4** (0.30 mmol) in hexane (0.20 mL) was added into the reaction solvent. The reaction tube was sealed with a Teflon screw cap, removed from the glove box. And the reaction mixture was stirred at 40 °C for 18 h. The selectivity was determined by ^1^H NMR analysis. And the crude reaction mixture was purified by column chromatography on silica gel or recrystallization using petroleum ether and ethyl acetate to afford the corresponding product **6**.

## Supplementary information

Supplementary Information

## Data Availability

The X-ray crystallographic data for compound Ni(dppe)_2_ has been deposited in the Cambridge Crystallographic Data Centre (CCDC), under deposition number CCDC 2095730, [https://www.ccdc.cam.ac.uk/structures/]. Data relating to the characterization data of materials and products, general methods, optimization studies, experimental procedures, mechanistic studies and NMR spectra are available in the Supplementary Information. All data are also available from the corresponding author upon request.
