# Supplementary material for: Nickel-catalyzed divergent Mizoroki–Heck reaction of 1,3-dienes
Source: Nat Commun. 2023 Feb 6;14:651. doi: 10.1038/s41467-023-36237-1 (PMC9902549; doi:10.1038/s41467-023-36237-1)
Supplement: Supplementary file 1 — Supplementary Information [file 41467_2023_36237_MOESM1_ESM.pdf]

Supplementary Information for:

Nickel-Catalyzed Divergent Mizoroki–Heck Reaction of 1,3-Dienes

Wei-Song Zhang,[1,2] Ding-Wei Ji,[1] Ying Li,[1,2] Xiang-Xin Zhang,[1,2] Yong-Kang Mei,[1,2] Bing-Zhi Chen,[1,2] and Qing-An Chen[1,2,*]

[1]Dalian Institute of Chemical Physics, Chinese Academy of Sciences, Dalian 116023, People's Republic of China

[2]University of Chinese Academy of Sciences, Beijing 100049, People's Republic of China

*Corresponding author E-mail: qachen@dicp.ac.cn

Table of Contents

1. Supplementary Note 1 .... S2
2. Supplementary Note 2 .... S2
    2.1 Optimization for linear Heck reaction of 1,3-Dienes. S2
    2.2 Optimization for branched Heck reaction of 1,3-Dienes. S4
3. Supplementary Methods S7
    3.1 Procedure for the synthesis of substrates S7
    3.2 General procedure A: Ni-catalyzed linear Heck reaction of 1,3-dienes S8
    3.3 General procedure B: Ni-catalyzed branched Heck reaction of 1,3-dienes S16
    3.4 Unsuccessful substrates S22
    3.5 Scale-up reaction S22
    3.6 Cascade Heck reactions S23
    3.7 Construction of highly aryl-substituted pyridines S25
    3.8 Construction of highly aryl-substituted benzenes S26
    3.9 Construction of diaryl thiophenes S27
    3.10 Construction of highly aryl-substituted naphthalene and thiophene S28
    3.11 Programmable constructions of poly (hetero)aromatic compounds S30
    3.12 Further Heck reaction S32
4. Supplementary Note 3 .... S33
    4.1 Control experiments S33
    4.2 Stereoconvergent Heck reactions of dienes S34
    4.3 The effect of ligands S35
    4.4 The capture of Ni (0) species S36
    4.5 The catalytic performance of Ni species S37
    4.6 ^{31}P NMR spectra of control experiments S38
    4.7 The EPR spectrum S40
    4.8 The role [Ar-B] species S42
    4.9 The detection of Heck product from aryl boron S42
    4.10 ^{19}F NMR and ^{11}B NMR spectra of control experiments S43
    4.11 Copy of NMR spectra S48
5. Supplementary References S133

1. Supplementary Note 1

All the reagents were commercially available and were used without further purification unless otherwise stated. Solvents were treated prior to use according to the standard methods. Unless otherwise stated, all reactions were conducted under inert atmosphere using standard Schlenk techniques or in an argon-filled glove-box. ^{1}H NMR and ^{13}C NMR spectra were recorded at room temperature in $CDCl_3$ on 400 MHz or 700 MHz instrument with tetramethylsilane (TMS) as internal standard. Data are reported as follows: chemical shift in ppm (δ), multiplicity (s = singlet, d = doublet, t = triplet, q = quartet, brs = broad singlet, m = multiplet), coupling constant (Hz), and integration. Flash column chromatography was performed on commercially available silica gel (200-300 mesh). All reactions were monitored by TLC, GC-FID, GC-MS or NMR analysis. HRMS data was obtained with Micromass HPLC-Q-TOF mass spectrometer (ESI) or Agilent 6540 Accurate-MS spectrometer (Q-TOF).

2. Supplementary Note 2 (Yields and selectivities were determined by GC-FID analysis of the crude products mixture using mesitylene as internal standard).

2.1 Optimization for linear Heck reaction of 1,3-Dienes.

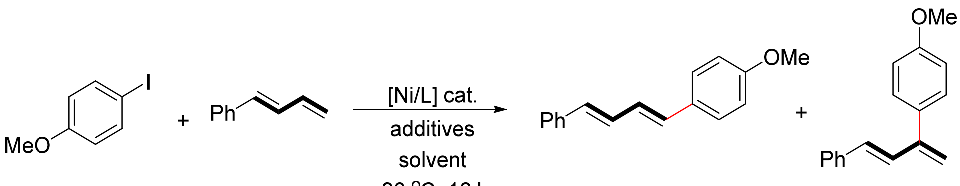

3a (0.22 mmol) **4a** (0.10 mmol) **5a** **6a**

Supplementary Table 1. Effect of ligands[a]

Entry	Ligand	Yield of **5a** (%)	Yield of **6a** (%)
1	dppm	--	--
2	dppe[b]	12	--
3	Xantphos	5	--
4	DPEphos	--	--
5	PCy_3[c]	--	--
6	PPh_3[c]	--	--
7	2,2'-bipyridine	trace	--
8	1,10-phenanthroline	--	--

[a]Conditions: **3a** (0.22 mmol), **4a** (0.10 mmol), $NiCl_2$ (10 mol%), ligand (12 mol%), Mn (0.15 mmol), MeCN (0.50 mL). [b]$Ni(dppe)Cl_2$ (10 mol%); [c]Ligand (24 mol%), 80 °C, 18 h.

Supplementary Table 2. Effect of reductants[a]

Entry	Reductant	Yield of **5a** (%)	Yield of **6a** (%)
1	Zn	10	--
2	In	9	--
3	Mn	12	--
4	Fe	trace	--
5	BEt_3	--	--
6	$HMeSi(OEt)_2$	6	--

[a]Conditions: **3a** (0.22 mmol), **4a** (0.10 mmol), Ni(dppe)Cl_2 (10 mol%), reductant (0.15 mmol.), MeCN (0.50 mL), 80 °C, 18 h.

Supplementary Table 3. Effect of solvents[a]

Entry	Solvent	Yield of **5a** (%)	Yield of **6a** (%)
1	MeCN	10	--
2	THF	9	--
3	PhMe	12	--
4	DMF	trace	--
5	1,4-dioxane	--	--

[a]Conditions: **3a** (0.22 mmol), **4a** (0.10 mmol), Ni(dppe)Cl_2 (10 mol%), Mn (0.15 mmol.), solvent (0.50 mL), 80 °C, 18 h.

Supplementary Table 4. Effect of the amount of NEt_3[a]

Entry	NEt_3	Yield of **5a** (%)	Yield of **6a** (%)
1	0.5 equiv.	15	--
2	1.0 equiv.	36	--
3	2.0 equiv.	42	--
4	0.056 mL (NEt_3/MeCN = 1/9)	60	--
5	0.10 mL (NEt_3/MeCN = 1/5)	31	--

[a]Conditions: **3a** (0.22 mmol), **4a** (0.10 mmol), Ni(dppe)Cl_2 (10 mol%), Mn (0.15 mmol.), MeCN (0.50 mL), 80 °C, 18 h.

Supplementary Table 5. Effect of additives[a]

Entry	Additive	Yield of **5a** (%)	Yield of **6a** (%)
1	NaCl	68	--
2	KCl	63	--
3	$MgCl_2$	37	--
4	$CaCl_2$	21	--
5	$CrCl_2$	47	--

[a]Conditions: **3a** (0.22 mmol), **4a** (0.10 mmol), Ni(dppe)Cl_2 (10 mol%), Mn (0.15 mmol.), additive (1.0 equiv.), MeCN/NEt_3 (0.50 mL/56 µL = 9:1), 80 °C, 18 h.

Supplementary Table 6. Effect of the amount of Mn[a]

Entry	Mn	Yield of **5a** (%)	Yield of **6a** (%)
1	0.5 equiv.	50	--
2	1.0 equiv.	69	--
3	1.5 equiv.	68	--

[a]Conditions: **3a** (0.22 mmol), **4a** (0.10 mmol), Ni(dppe)Cl_2 (10 mol%), NaCl (1.0 equiv.), MeCN/NEt_3 (0.50 mL/56 µL = 9:1), 80 °C, 18 h.

2.2 Optimization for branched Heck reaction of 1,3-Dienes.

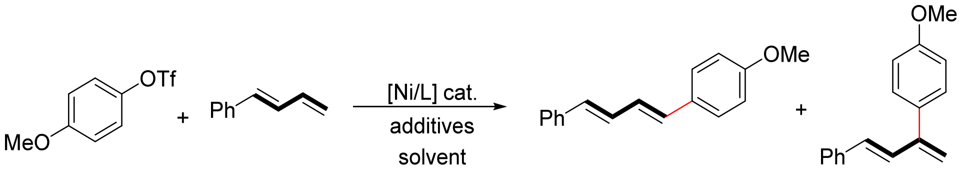

1a (0.15 mmol) **4a** (0.10 mmol) **5a** **6a**

Supplementary Table 7. Effect of bases[a]

Entry	Base	Yield of **5a** (%)	Yield of **6a** (%)
1	NaOMe	7	7
2	NaOEt	7	10
3	^{t}BuOK	6	11
4	^{t}BuOLi	45	18
5	Cs_2CO_3	5	11
6	NaOAc	--	--
7	K_3PO_4	3	4
8	DBU	2	2

[a]Conditions: **1a** (0.15 mmol), **4a** (0.10 mmol), Ni$(cod)_2$ (10 mol%), IMes·HCl (12 mol%), base (0.15 mmol), 1,4-dioxane (0.50 mL), 80 °C, 18 h.

Supplementary Table 8. Effect of ligands[a]

Entry	Ligand	Yield of **5a** (%)	Yield of **6a** (%)
1	PPh_3[b]	8	--
2	PCy_3[b]	10	5
3	dppf	5	2
4	dppp	trace	trace
5	bipyridine	--	--
6	IPr·HCl	1	16
7	SIPr·HCl	trace	3

[a]Conditions: **1a** (0.15 mmol), **4a** (0.10 mmol), $Ni(cod)_2$ (10 mol%), ligand (12 mol%), Cs_2CO_3 (0.15 mmol), 1,4-dioxane (0.50 mL), 80 °C, 18 h. [b]ligand (24 mol%).

Supplementary Table 9. Effect of additives[a]

Entry	Additive	Yield of **5a** (%)	Yield of **6a** (%)
1	TMSOTf[b]	--	4
2	TMSCl[b]	--	--
3	BEt_3	5	25
4	$AlMe_3$	3	8
5	BCF	trace	4
6	$(Bpin)_2$	7	26

[a]Conditions: **1a** (0.15 mmol), **4a** (0.10 mmol), $Ni(cod)_2$ (10 mol%), IPr·HCl (12 mol%), Cs_2CO_3 (0.15 mmol), additive (20 mol%), 1,4-dioxane (0.50 mL), 80 °C, 18 h. [b]additive (50 mol%).

Supplementary Table 10. Effect of temperature[a]

Entry	T/°C	Yield of **5a** (%)	Yield of **6a** (%)
1	100	5	23
2	80	7	26
3	60	5	26
4	40	4	23

[a]Conditions: **1a** (0.15 mmol), **4a** (0.10 mmol), $Ni(cod)_2$ (10 mol%), IPr·HCl (12 mol%), Cs_2CO_3 (0.15 mmol), $(Bpin)_2$ (20 mol%), 1,4-dioxane (0.50 mL), T °C, 18 h.

Supplementary Table 11. Effect of solvents[a]

Entry	Solvent	Yield of **5a** (%)	Yield of **6a** (%)
1	THF	3	19
2	PhMe	2	7
3	DMF	--	31
4	DMSO	--	18
5	MeCN	trace	31
6	NMP	--	--

[a]Conditions: **1a** (0.15 mmol), **4a** (0.10 mmol), Ni(cod)$_2$ (10 mol%), IPr·HCl (12 mol%), Cs_2CO_3 (0.15 mmol), (Bpin)$_2$ (20 mol%), solvent (0.50 mL), 40 °C, 18 h.

Supplementary Table 12. Effect of [B][a]

Entry	[B]	Yield of **5a** (%)	Yield of **6a** (%)
1	AllylBpin	--	34
2	(Bpin)$_2$	--	41
3	PhBpin	--	52
4	PhB(OH)$_2$	--	39
5	PhBneop	--	60

[a]Conditions: **1a** (0.15 mmol), **4a** (0.10 mmol), Ni(cod)$_2$ (10 mol%), IPr·HCl (12 mol%), Cs_2CO_3 (0.15 mmol), [B]/^{t}BuOK (20 mol%), DMF (0.50 mL), 40 °C, 18 h.

Supplementary Table 13. Effect of ArBneop[a]

Entry	[B]	Yield of **5a** (%)	Yield of **6a** (%)
1	**B1**	--	66
2	**B2**	--	63
3	**B3**	--	57
4	**B4**	--	58
5	**B5**	--	55
6	**B6**	--	45

[a]Conditions: **1a** (0.15 mmol), **4a** (0.10 mmol), Ni(cod)$_2$ (10 mol%), IPr·HCl (12 mol%), Cs_2CO_3 (0.15 mmol), [B]/^{t}BuOK (20 mol%), DMF (0.50 mL), 40 °C, 18 h.

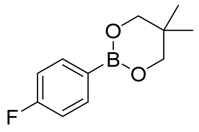

B1

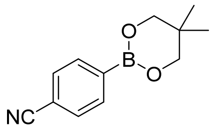

B2

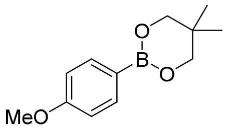

B3

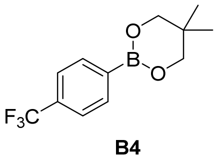

B4

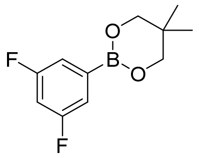

B5

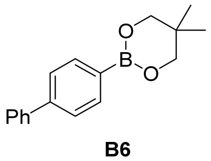

B6

3. Supplementary Methods

3.1 Procedure for the synthesis of substrates

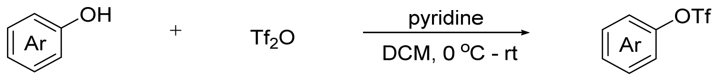

1.2 equiv. **1a**

To a flame-dried round-bottom flask, the solution of the phenol (20 mmol) and pyridine (24 mmol) in CH_2Cl_2 (20 mL) was added Tf_2O (24 mmol) slowly at 0 °C under N_2. After stirring for overnight, the reaction mixture was quenched with sat. NH_4Cl aq. (15 mL) and extracted with diethyl ether (20 mL × 3). The combined organic layers were dried over Na_2SO_4, concentrated in vacuo and purified by flash chromatography on silica gel with *n*-pentene or *n*-hexane to afford the aryl triflates.

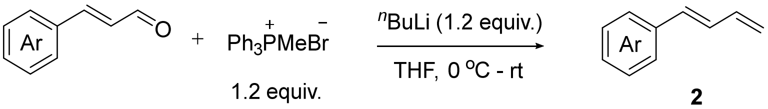

1.2 equiv. **2**

To a flame-dried round-bottom flask, methyltriphenylphosphonium bromide (6.0 mmol) in THF (40 mL) was added ^{n}BuLi (2.4 mL, 2.5 M in hexane, 6.0 mmol) slowly at 0 °C under N_2. After stirring for 20 min, a cinnamaldehyde (5.0 mmol) was added. The reaction mixture was then warmed to room temperature and stirred for another 5-10 hours. After the starting material was consumed completely which was detected by TLC, the reaction mixture was quenched with sat. NH_4Cl aq. (15 mL) and extracted with diethyl ether (20 mL × 3). The combined organic layers were dried over Na_2SO_4, concentrated in vacuo and purified by flash chromatography on silica gel with *n*-pentene or *n*-hexane to afford the diene products.

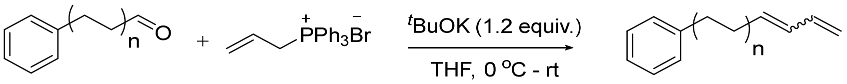

n=0 or 2 1.2 equiv. **2** *E/Z* = 1:1

To a flame-dried round-bottom flask, allyltriphenylphosphonium bromide (6.0 mmol) in THF (40 mL) was added potassium *tert*-butoxide (6.0 mmol) at 0 °C under N_2. After stirring for 20 min, an aldehyde (5.0 mmol) was added. The reaction mixture was then warmed to room temperature and stirred for another 10-18 hours. After the starting material was consumed completely which was detected by TLC, the reaction mixture was quenched with sat. NH_4Cl aq. (15 mL) and extracted with diethyl ether (20 mL × 3). The combined organic layers were dried over Na_2SO_4, concentrated in vacuo and purified by flash chromatography on silica gel with n-hexane to afford the diene products.

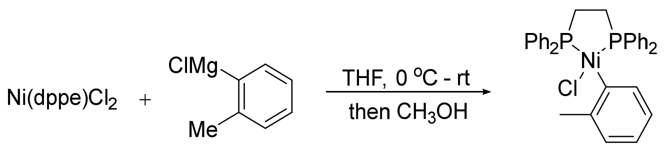

Ni(2-MeC_6H_4)Cl(dppe): 68% yield

Ni(dppe)Cl_2 (1.0 mmol, 528 mg) was placed in an oven-dried, 125 mL round-bottom flask containing a magnetic stir bar. THF (50 mL) was added, the mixture was cooled to 0 °C with an ice bath, and o-tolylmagnesium chloride (2.0 mmol, 1.0 M in THF, 2.0 mL) was added dropwise with vigorous stirring. Partway through the addition, the solution became completely homogeneous and began to change color

to yellow. After complete addition of the Grignard reagent, the solution was stirred for 30 min at 0 °C, after which the stir bar was removed and the solution was evaporated to dryness under reduced pressure. MeOH (10 mL) was added and the mixture was sonicated until a uniform suspension was obtained (approximately 5 min). After the suspension was cooled to 0 °C, the yellow precipitate was collected by vacuum filtration, washed with two portions of cold MeOH (5 mL), and dried under high vacuum to yield Ni(2-MeC_6H_4)Cl(dppe) (397 mg, 68 %) as a fine, bright yellow powder.[1]

3.2 General procedure A: Ni-catalyzed linear Heck reaction of 1,3-dienes

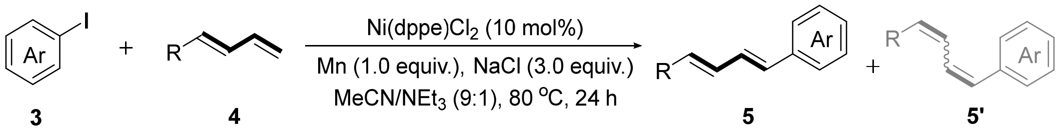

3 **4** **5** **5'**

In a glove box, a sealed tube was charged with iodoarene **3** (0.40 mmol), Ni(dppe)Cl_2 (0.02 mmol, 10 mol%), Mn (0.20 mmol), NaCl (0.60 mmol), 1,3-diene **4** (0.20 mmol), MeCN (0.5 mL), NEt_3 (56 uL) at room temperature. The reaction tube was sealed with a Teflon screw cap, removed from the glove box. Then, the reaction mixture was stirred at 80 ºC for 24 hours. The ratio of **5** and **5'** was determined by GC-FID analysis. And the crude reaction mixture was purified by column chromatography on silica gel or recrystallization using petroleum ether and dichloromethane to afford the corresponding product **5.**

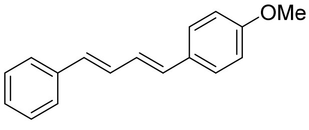

1-Methoxy-4-((1*E*,3*E*)-4-phenylbuta-1,3-dien-1-yl)benzene (5a): Prepared according to the general procedure A, known compound,[2] white solid, 36.9 mg, 78% yield, R_f = 0.4 (PE/DCM = 4/1). **^{1}H NMR** (400 MHz, Chloroform-*d*) δ 7.44 (d, *J* = 7.6 Hz, 2H), 7.40 (d, *J* = 8.5 Hz, 2H), 7.34 (t, *J* = 7.5 Hz, 2H), 7.23 (t, *J* = 7.4 Hz, 1H), 7.01-6.78 (m, 4H), 6.64 (d, *J* = 15.2 Hz, 2H), 3.83 (s, 3H); **^{13}C NMR** (100 MHz, Chloroform-*d*) δ 159.41, 137.69, 132.58, 131.80, 130.34, 129.65, 128.76, 127.75, 127.44, 127.39, 126.38, 114.28, 55.44. **HRMS** calculated for $C_{17}H_{16}O$ $[M]^+$ 236.1196, found 236.1200.

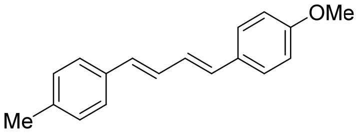

1-Methoxy-4-((1*E*,3*E*)-4-(p-tolyl)buta-1,3-dien-1-yl)benzene (5b): Prepared according to the general procedure A, known compound,[3] white solid, 34.0 mg, 74% yield, R_f = 0.5 (PE/DCM = 4/1), **^{1}H NMR** (400 MHz, Chloroform-*d*) δ 7.39 (d, *J* = 8.7 Hz, 2H), 7.34 (d, *J* = 8.0 Hz, 2H), 7.15 (d, *J* = 7.9 Hz, 2H), 6.95-6.78 (m, 4H), 6.61 (d, *J* = 14.9 Hz, 2H), 3.83 (s, 3H), 2.36 (s, 3H); **^{13}C NMR** (100 MHz, Chloroform-*d*) δ 159.31, 137.34, 134.92, 131.99, 131.82, 130.46, 129.48, 128.72, 127.67, 127.58, 126.31, 114.26, 55.44, 21.38. **HRMS** calculated for $C_{18}H_{18}O$ $[M]^+$ 250.1352, found 250.1352.

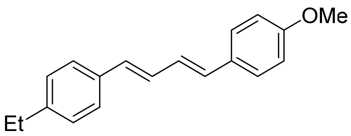

1-Ethyl-4-((1*E*,3*E*)-4-(4-methoxyphenyl)buta-1,3-dien-1-yl)benzene (5c): Prepared according to the general procedure A, white solid, melting point: 153-155 ºC, 41.4 mg, 78% yield, R_f = 0.5 (PE/DCM = 4/1), **^{1}H NMR** (400 MHz, Chloroform-*d*) δ 7.48-7.34 (m, 4H), 7.19 (d, *J* = 7.9 Hz, 2H), 6.99-6.79 (m, 4H), 6.72-6.55 (m, 2H), 3.83 (s, 3H), 2.67 (q, *J* = 7.5 Hz, 2H), 1.27 (t, *J* = 7.6 Hz, 3H); **^{13}C NMR** (100 MHz, Chloroform-*d*) δ 159.30, 143.74, 135.18,

132.00, 131.83, 130.45, 128.78, 128.28, 127.67, 127.59, 126.39, 114.25, 55.41, 28.77, 15.65. **HRMS** calculated for $C_{19}H_{20}O$ $[M]^+$ 264.1509, found 264.1513.

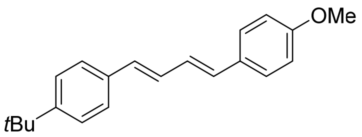

1-(*tert*-Butyl)-4-((1*E*,3*E*)-4-(4-methoxyphenyl)buta-1,3-dien-1-yl)benzene (5d): Prepared according to the general procedure A, white solid, melting point: 199-201 °C, 30.6 mg, 52% yield, R_f = 0.5 (PE/DCM = 4/1), **^{1}H NMR** (400 MHz, Chloroform-*d*) δ 7.44-7.33 (m, 6H), 6.96-6.80 (m, 4H), 6.62 (d, *J* = 16.3 Hz, 2H), 3.83 (s, 3H), 1.33 (s, 9H); **^{13}C NMR** (101 MHz, Chloroform-*d*) δ 159.33, 150.63, 134.95, 132.03, 131.70, 130.47, 128.95, 127.68, 127.64, 126.14, 125.71, 114.27, 55.45, 34.75, 31.43. **HRMS** calculated for $C_{21}H_{24}O$ $[M]^+$ 292.1822, found 292.1821.

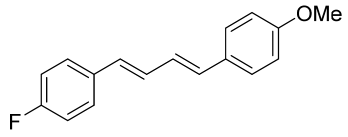

1-Fluoro-4-((1*E*,3*E*)-4-(4-methoxyphenyl)buta-1,3-dien-1-yl)benzene (5e): Prepared according to the general procedure A, white solid, 35.1 mg (**5e**+**5e'**), 69% yield (**5e**/**5e'** = 88/12), R_f = 0.5 (PE/DCM = 4/1, recrystallization), **^{1}H NMR** (400 MHz, Chloroform-*d*) δ 7.44-7.35 (m, 4H), 7.02 (t, *J* = 8.7 Hz, 2H), 6.91-6.77 (m, 4H), 6.67-6.54 (m, 2H), 3.83 (s, 3H); **^{13}C NMR** (100 MHz, Chloroform-*d*) δ 162.27 (d, *J* = 247.0 Hz), 159.45, 133.90 (d, *J* = 3.4 Hz), 132.62, 130.49, 130.27, 129.43 (d, *J* = 2.5 Hz), 127.80 (d, *J* = 8.1 Hz), 127.75, 127.18, 115.71 (d, *J* = 21.7 Hz), 114.29, 55.45; **^{19}F NMR** (376 MHz, Chloroform-*d*) δ -114.54. **HRMS** calculated for $C_{17}H_{15}FO$ $[M]^+$ 254.1101, found 254.1102.

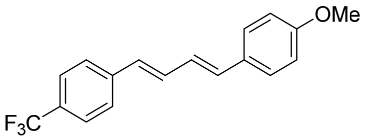

1-Methoxy-4-((1*E*,3*E*)-4-(4-(trifluoromethyl)phenyl)buta-1,3-dien-1-yl)benzene (5f): Prepared according to the general procedure A, white solid, 48.0 mg (**5f**+**5f'**), 79% yield (**5f**/**5f'** = 87/13), R_f = 0.6 (PE/DCM = 3/1, recrystallization), **^{1}H NMR** (400 MHz, Chloroform-*d*) δ 7.57 (d, *J* = 6.8 Hz, 2H), 7.50 (d, *J* = 6.9 Hz, 2H), 7.40 (d, *J* = 7.6 Hz, 2H), 7.09-6.96 (m, 1H), 6.96-6.77 (m, 3H), 6.69 (d, *J* = 15.4 Hz, 1H), 6.62 (d, *J* = 15.3 Hz, 1H), 3.83 (s, 3H); **^{13}C NMR** (100 MHz, Chloroform-*d*) δ 159.75, 141.19, 134.35, 132.08, 129.98, 128.95 (q, *J* = 32.9 Hz), 128.00, 126.73, 126.37, 125.69 (q, *J* = 3.5 Hz), 124.37 (q, *J* = 272.2 Hz), 114.35, 55.46; **^{19}F NMR** (376 MHz, Chloroform-*d*) δ -62.41. **HRMS** calculated for $C_{18}H_{15}F_3O$ $[M]^+$ 304.1070, found 304.1073.

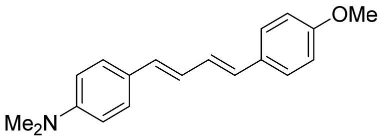

4-((1*E*,3*E*)-4-(4-Methoxyphenyl)buta-1,3-dien-1-yl)-*N,N*-dimethylaniline (5g): Prepared according to the general procedure A, green solid, melting point: 194-196 °C, 46.1 mg, 83% yield, R_f = 0.5 (PE/DCM = 3/1), **^{1}H NMR** (400 MHz, Chloroform-*d*) δ 7.41-7.29 (m, 4H), 6.87 (d, *J* = 8.6 Hz, 2H), 6.84-6.73 (m, 2H), 6.70 (d, *J* = 8.6 Hz, 2H), 6.62-6.49 (m, 2H), 3.82 (s, 3H), 2.98 (s, 6H); **^{13}C NMR** (100 MHz, Chloroform-*d*) δ 159.00, 150.05, 132.25, 130.89, 130.08, 128.22, 127.50, 127.41, 125.66, 114.23, 112.64, 55.45, 40.63. **HRMS** calculated for $C_{19}H_{22}NO$ $[M+H]^+$ 280.1696, found 280.1693.

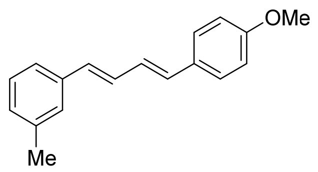

1-((1*E*,3*E*)-4-(4-Methoxyphenyl)buta-1,3-dien-1-yl)-3-methylbenzene (5h): Prepared according to the general procedure A, white solid, melting point: 105-107 °C, 40.5 mg, 81% yield, R_f = 0.5 (PE/DCM = 4/1), **^{1}H NMR** (400 MHz, Chloroform-*d*) δ 7.40 (d, *J* = 8.7 Hz, 2H), 7.29-7.21 (m, 3H), 7.07 (d, *J* = 6.5 Hz, 1H), 7.00-6.81 (m, 4H), 6.69-6.56 (m, 2H), 3.83 (s, 3H), 2.38 (s, 3H); **^{13}C NMR** (100 MHz, Chloroform-*d*) δ 159.37, 138.26, 137.63, 132.38, 131.94, 130.38, 129.48, 128.65, 128.30, 127.73, 127.49, 127.10, 123.57, 114.27, 55.42, 21.56. **HRMS** calculated for $C_{18}H_{18}O$ $[M]^+$ 250.1352, found 250.1356.

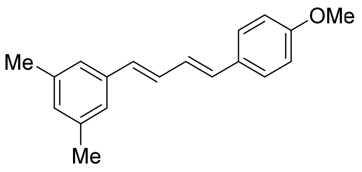

1-((1*E*,3*E*)-4-(4-Methoxyphenyl)buta-1,3-dien-1-yl)-3,5-dimethylbenzene (5i): Prepared according to the general procedure A, white solid, melting point: 133-134 °C, 37.9 mg, 72% yield, R_f = 0.5 (PE/DCM = 4/1), **^{1}H NMR** (400 MHz, Chloroform-*d*) δ 7.40 (d, *J* = 8.4 Hz, 2H), 7.08 (s, 2H), 7.00-6.78 (m, 5H), 6.70-6.53 (m, 2H), 3.83 (s, 3H), 2.34 (s, 6H); **^{13}C NMR** (100 MHz, Chloroform-*d*) δ 159.34, 138.15, 137.60, 132.19, 132.06, 130.43, 129.33, 129.30, 127.70, 127.59, 124.31, 114.26, 55.42, 21.43. **HRMS** calculated for $C_{19}H_{20}O$ $[M]^+$ 264.1509, found 264.1513.

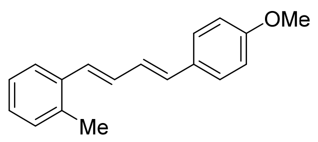

1-((1*E*,3*E*)-4-(4-Methoxyphenyl)buta-1,3-dien-1-yl)-2-methylbenzene (5j): Prepared according to the general procedure A, white solid, melting point: 91-93 °C, 40.5 mg, 81% yield, R_f = 0.5 (PE/DCM = 4/1), **^{1}H NMR** (400 MHz, Chloroform-*d*) δ 7.56 (d, *J* = 7.5 Hz, 1H), 7.40 (d, *J* = 8.6 Hz, 2H), 7.24-7.13 (m, 3H), 6.97-6.79 (m, 5H), 6.70-6.60 (m, 1H), 3.83 (s, 3H), 2.41 (s, 3H); **^{13}C NMR** (100 MHz, Chloroform-*d*) δ 159.37, 136.46, 135.54, 132.44, 130.69, 130.56, 130.34, 129.35, 127.75, 127.73, 127.35, 126.24, 125.03, 114.25, 55.39, 20.01. **HRMS** calculated for $C_{18}H_{18}O$ $[M]^+$ 250.1352, found 250.1357.

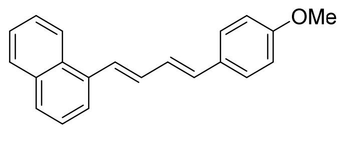

1-((1*E*,3*E*)-4-(4-Methoxyphenyl)buta-1,3-dien-1-yl)naphthalene (5k): Prepared according to the general procedure A, yellow solid, melting point: 141-143 °C, 40.1 mg, 70% yield, R_f = 0.5 (PE/DCM = 4/1), **^{1}H NMR** (400 MHz, Chloroform-*d*) δ 8.22 (d, *J* = 8.1 Hz, 1H), 7.87 (d, *J* = 7.9 Hz, 1H), 7.78 (d, *J* = 8.1 Hz, 1H), 7.73 (d, *J* = 7.2 Hz, 1H), 7.60-7.38 (m, 6H), 7.08-6.96 (m, 2H), 6.91 (d, *J* = 8.6 Hz, 2H), 6.75-6.63 (m, 1H), 3.84 (s, 3H); **^{13}C NMR** (101 MHz, Chloroform-*d*) δ 159.49, 134.97, 133.93, 132.90, 132.38, 131.23, 130.31, 128.74, 128.36, 127.88, 127.83, 127.68, 126.14, 125.89, 125.79, 123.74, 123.21, 114.31, 55.46. **HRMS** calculated for $C_{21}H_{19}O$ $[M+H]^+$ 287.1430, found 287.1423.

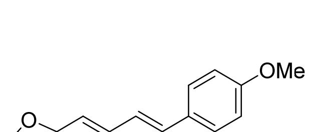

2-((1*E*,3*E*)-4-(4-Methoxyphenyl)buta-1,3-dien-1-yl)furan (5l): Prepared according to the general procedure A, yellow solid, 32.3 mg (**5l**+**5l'**), 72% yield (**5l**/**5l'** = 83/17), R_f = 0.6 (PE/DCM = 3/1, recrystallization), **^{1}H NMR** (400 MHz, Chloroform-*d*) δ 7.44-7.31 (m, 3H), 6.91-6.68 (m, 4H), 6.60 (d, *J* = 15.0 Hz, 1H), 6.50-6.33 (m, 2H), 6.26 (d, *J* = 2.9 Hz, 1H), 3.81 (s, 3H); **^{13}C NMR** (100 MHz, Chloroform-*d*) δ 159.40, 153.61, 142.13, 132.65, 130.33, 128.34, 127.74,

126.93, 119.31, 114.27, 111.80, 108.15, 55.43. **HRMS** calculated for $C_{15}H_{15}O_2$ $[M+H]^+$ 227.1067, found 227.1065.

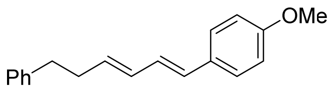

1-Methoxy-4-((1*E*,3*E*)-6-phenylhexa-1,3-dien-1-yl)benzene (5m): Prepared according to the general procedure A, white solid, 35.0 mg (**5m+5m'**), 66% yield (**5m/5m'** = 90/10), R_f = 0.5 (PE/DCM = 3/1, recrystallization), **^{1}H NMR** (400 MHz, Chloroform-*d*) δ 7.41-7.27 (m, 4H), 7.24-7.14 (m, 3H), 6.85 (d, *J* = 8.2 Hz, 2H), 6.62 (dd, *J* = 15.5, 10.3 Hz, 1H), 6.40 (d, *J* = 15.6 Hz, 1H), 6.22 (dd, *J* = 14.8, 10.8 Hz, 1H), 5.80 (dt, *J* = 14.4, 6.8 Hz, 1H), 3.81 (s, 3H), 2.75 (t, *J* = 7.7 Hz, 2H), 2.47 (q, *J* = 7.3 Hz, 2H); **^{13}C NMR** (100 MHz, Chloroform-*d*) δ 159.10, 141.93, 133.49, 131.36, 130.55, 130.13, 128.59, 128.47, 127.47, 127.40, 125.99, 114.18, 55.45, 36.00, 34.82. **HRMS** calculated for $C_{18}H_{18}O$ $[M]^+$ 264.1509, found 264.1509.

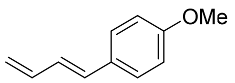

(*E*)-1-(Buta-1,3-dien-1-yl)-4-methoxybenzene (5n): Prepared according to the general procedure A (**3a** (0.20 mmol), 1,3-butadiene (0.60 mmol)), known compound,[4] white solid, 10.3 mg, 32% yield, R_f = 0.7 (PE/DCM = 3/1), **^{1}H NMR** (400 MHz, Chloroform-*d*) δ 7.34 (d, *J* = 8.7 Hz, 2H), 6.86 (d, *J* = 8.8 Hz, 2H), 6.67 (dd, *J* = 15.4, 10.5 Hz, 1H), 6.62-6.36 (m, 2H), 5.28 (d, *J* = 15.4 Hz, 1H), 5.11 (d, *J* = 8.6 Hz, 1H), 3.81 (s, 3H); **^{13}C NMR** (100 MHz, Chloroform-*d*) δ 159.43, 137.51, 132.54, 130.08, 127.79, 116.58, 114.22, 55.45. **HRMS** calculated for $C_{11}H_{12}O$ $[M]^+$ 160.0883, found 160.0883.

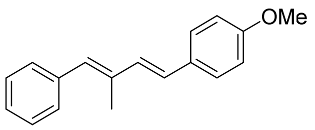

1-Methoxy-4-((1*E*,3*E*)-3-methyl-4-phenylbuta-1,3-dien-1-yl)benzene (5o): Prepared according to the general procedure A, known compound,[5] white solid, 33.4 mg, 67% yield, R_f = 0.5 (PE/EA = 50/1), **^{1}H NMR** (400 MHz, Chloroform-*d*) δ 7.42 (d, *J* = 8.7 Hz, 2H), 7.39-7.32 (m, 4H), 7.25-7.21 (m, 1H), 6.96-6.83 (m, 3H), 6.72-6.52 (m, 2H), 3.83 (s, 3H), 2.14 (d, *J* = 1.1 Hz, 3H); **^{13}C NMR** (100 MHz, Chloroform-*d*) δ 159.21, 138.16, 136.14, 132.35, 131.39, 130.59, 129.36, 128.27, 127.71, 127.65, 126.59, 114.26, 55.45, 14.11.

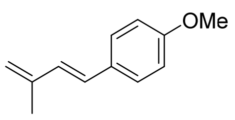

(*E*)-1-Methoxy-4-(3-methylbuta-1,3-dien-1-yl)benzene (5p): Prepared according to the general procedure A (**3a** (0.20 mmol), isoprene (0.60 mmol)), known compound,[6] an inseparable mixture of **5p** and **other isomers** (**5p/other isomers** = 3:1), 14.8 mg, 42% yield, R_f = 0.4 (PE/EA = 50/1), **^{1}H NMR** (400 MHz, Chloroform-*d*) δ 7.42 (d, *J* = 8.7 Hz, 2H), 7.39-7.32 (m, 4H), 7.25-7.21 (m, 1H), 6.96-6.83 (m, 3H), 6.72-6.52 (m, 2H), 3.83 (s, 3H), 2.14 (d, *J* = 1.1 Hz, 3H); **^{13}C NMR** (100 MHz, Chloroform-*d*) δ 159.25, 142.31, 130.29, 129.83, 128.30, 127.76, 116.44, 114.19, 55.44, 18.78.

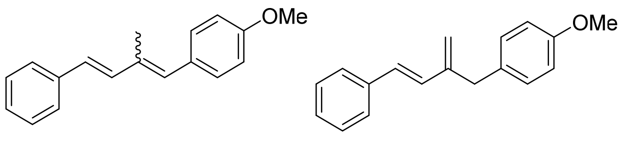

5q and **5q''**
(**5q:5q''** = 1:1)

1-Methoxy-4-((3*E*)-2-methyl-4-phenylbuta-1,3-dien-1-yl)benzene (5q) and (*E*)-1-methoxy-4-(2-methylene-4-phenylbut-3-en-1-yl)benzene (5q''): Prepared according to the general procedure A, known compounds,[5] an inseparable mixture of **5q** and **5q''** (**5q/5q''** = 1:1), 9.1 mg, 18% yield, R_f = 0.5 (PE/EA = 50/1), **^{1}H**

NMR (400 MHz, Chloroform-*d*) δ 7.47 (d, *J* = 7.4 Hz, 2H), 7.41-7.29 (m, 8H), 7.24-7.15 (m, 5H), 6.99 (d, *J* = 16.0 Hz, 1H), 6.93-6.89 (m, 2H), 6.87-6.83 (m, 2H), 6.70-6.58 (m, 3H), 5.28 (s, 1H), 4.97 (s, 1H), δ 3.84 (s, 1H), 3.80 (s, 1H). 3.63 (s, 2H), 2.14 (d, *J* = 0.9 Hz, 3H); **^{13}C NMR** (100 MHz, Chloroform-*d*) δ 158.50, 145.50, 137.95, 137.42, 134.59, 134.51, 132.09, 131.56, 130.75, 130.67, 129.92, 129.11, 128.77, 128.70, 127.61, 127.37, 126.59, 126.45, 118.55, 113.93, 113.81, 55.43, 37.92, 14.09.

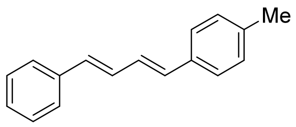

Methyl-4-((1*E*,3*E*)-4-phenylbuta-1,3-dien-1-yl)benzene (5r): Prepared according to the general procedure A, known compound,[7] white solid, 35.8 mg, 81% yield, R_f = 0.5 (PE), **^{1}H NMR** (400 MHz, Chloroform-*d*) δ 7.48 (d, *J* = 7.5 Hz, 2H), 7.42-7.31 (m, 4H), 7.27 (t, *J* = 7.3 Hz, 1H), 7.18 (d, *J* = 7.9 Hz, 2H), 7.04-6.90 (m, 2H), 6.78-6.58 (m, 2H), 2.39 (s, 3H); **^{13}C NMR** (100 MHz, Chloroform-*d*) δ 137.60, 134.71, 132.97, 132.36, 129.56, 129.51, 128.76, 128.44, 127.54, 126.45, 21.40. **HRMS** calculated for $C_{17}H_{16}$ $[M]^+$ 220.1247, found 220.1241.

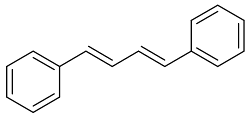

(1*E*,3*E*)-1,4-Diphenylbuta-1,3-diene (5s): Prepared according to the general procedure A, known compound,[2] white solid, 37.8 mg, 92% yield, R_f = 0.5 (PE), **^{1}H NMR** (400 MHz, Chloroform-*d*) δ 7.43 (d, *J* = 7.6 Hz, 4H), 7.32 (t, *J* = 7.6 Hz, 4H), 7.22 (t, *J* = 7.2 Hz, 2H), 7.02-6.88 (m, 2H), 6.77-6.56 (m, 2H); **^{13}C NMR** (100 MHz, Chloroform-*d*) δ 137.49, 132.95, 129.38, 128.79, 127.69, 126.52. **HRMS** calculated for $C_{16}H_{14}$ $[M]^+$ 206.1090, found 206.1097.

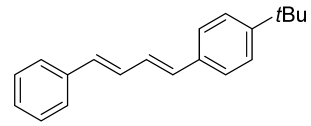

1-(*tert*-Butyl)-4-((1*E*,3*E*)-4-phenylbuta-1,3-dien-1-yl)benzene (5t): Prepared according to the general procedure A, known compound,[8] white solid, 45.3mg, 87% yield, R_f = 0.5 (PE), **^{1}H NMR** (400 MHz, Chloroform-*d*) δ 7.45 (d, *J* = 7.5 Hz, 2H), 7.43-7.36 (m, 4H), 7.33 (d, *J* = 7.8 Hz, 2H), 7.24 (t, *J* = 7.3 Hz, 1H), 7.04-6.89 (m, 2H), 7.71-6.64 (m, 2H), 1.35 (s, 9H); **^{13}C NMR** (100 MHz, Chloroform-*d*) δ 150.87, 137.60, 134.73, 132.85, 132.40, 129.60, 128.77, 128.66, 127.55, 126.45, 126.28, 125.73, 34.76, 31.42. **HRMS** calculated for $C_{20}H_{22}$ $[M]^+$ 262.1716, found 262.1721.

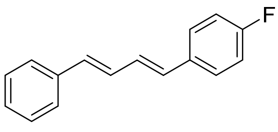

1-Fluoro-4-((1*E*,3*E*)-4-phenylbuta-1,3-dien-1-yl)benzene (5u): Prepared according to the general procedure A, known compound,[9] white solid, 40.2 mg, 90% yield, R_f = 0.5 (PE), **^{1}H NMR** (400 MHz, Chloroform-*d*) δ 7.47 (d, *J* = 7.4 Hz, 2H), 7.45-7.39 (m, 2H), 7.36 (t, *J* = 7.6 Hz, 2H), 7.27 (t, *J* = 7.3 Hz, 1H), 7.05 (t, *J* = 8.7 Hz, 2H), 6.96 (dd, *J* = 15.0, 10.4 Hz, 1H), 6.89 (dd, *J* = 14.9, 10.5 Hz, 1H), 6.81-6.55 (m, 2H); **^{13}C NMR** (100 MHz, Chloroform-*d*) δ 162.39 (d, *J* = 247.4 Hz), 137.41, 133.68 (d, *J* = 3.4 Hz), 132.99, 131.63, 129.15, 129.12, 128.80, 127.96 (d, *J* = 7.9 Hz), 127.73, 126.51, 115.74 (d, *J* = 21.7 Hz); **^{19}F NMR** (376 MHz, Chloroform-*d*) δ -114.06. **HRMS** calculated for $C_{16}H_{13}F$ $[M]^+$ 224.0996, found 224.1001.

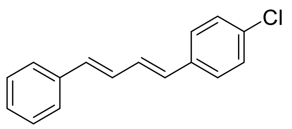

1-Chloro-4-((1*E*,3*E*)-4-phenylbuta-1,3-dien-1-yl)benzene (5v): Prepared according to the general procedure A, known compound,[2]. white solid, 39.6 mg, 83% yield, R_f = 0.5 (PE), **^{1}H NMR** (400 MHz, Chloroform-*d*) δ 7.43 (d, *J* = 7.5 Hz, 2H), 7.38-7.30 (m, 4H), 7.29-7.27 (m, 2H), 7.25-7.20 (m, 1H), 6.98-6.84 (m, 2H), 6.72-6.64 (m, 1H), 6.64-6.54 (m, 1H); **^{13}C NMR** (100

MHz, Chloroform-*d*) δ 137.31, 135.99, 133.53, 133.17, 131.48, 129.95, 129.01, 128.94, 128.81, 127.85, 127.62, 126.57. **HRMS** calculated for $C_{16}H_{13}Cl^{35}$ $[M]^+$ 240.0700, found 240.0708; for $C_{16}H_{13}Cl^{37}$ $[M]^+$ 242.0671, found 242.0685.

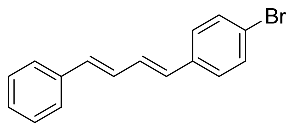

1-Bromo-4-((1*E*,3E)-4-phenylbuta-1,3-dien-1-yl)benzene (5w): Prepared according to the general procedure A, known compound,[10] white solid, 37.1 mg, 63% yield (Accompanied by a small amount of inseparable 4,4'-dibromo-1,1'-biphenyl, the yield of the **3g** has been adjusted according to ^{1}H NMR analysis), R_f = 0.5 (PE), **^{1}H NMR** (400 MHz, Chloroform-*d*) δ 7.50-7.41 (m, 4H), 7.35 (t, *J* = 7.6 Hz, 2H), 7.30 (d, *J* = 8.5 Hz, 2H), 7.26 (t, *J* = 7.4 Hz, 1H), 6.99-6.89 (m, 2H), 6.74-6.66 (m, 1H), 6.65-6.53 (m, 1H); **^{13}C NMR** (100 MHz, Chloroform-*d*) δ 137.30, 136.42, 133.63, 131.88, 131.52, 130.06, 129.00, 128.82, 127.93, 127.87, 126.58, 121.34. **HRMS** calculated for $C_{16}H_{13}Br^{79}$ $[M]^+$ 284.0195, found 284.0195; for $C_{16}H_{13}Br^{81}$ $[M]^+$ 286.0175, found 284.0186.

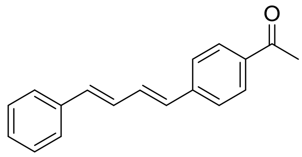

(4-((1*E*,3*E*)-4-Phenylbuta-1,3-dien-1-yl)phenyl)ethan-1-one (5x): Prepared according to the general procedure A, yellow solid, melting point: 146-148 °C, 34.0 mg, 69% yield, R_f = 0.2 (PE/DCM = 2/1), **^{1}H NMR** (400 MHz, Chloroform-*d*) δ 7.92 (d, *J* = 8.3 Hz, 2H), 7.50 (d, *J* = 8.3 Hz, 2H), 7.46 (d, *J* = 7.4 Hz, 2H), 7.35 (t, *J* = 7.5 Hz, 2H), 7.27 (t, *J* = 7.3 Hz, 1H), 7.06 (dd, *J* = 15.2, 10.6 Hz, 1H), 6.96 (dd, *J* = 15.2, 10.6 Hz, 1H), 6.75 (d, *J* = 15.2 Hz, 1H), 6.68 (d, *J* = 15.2 Hz, 1H), 2.59 (s, 3H); **^{13}C NMR** (100 MHz, Chloroform-*d*) δ 197.48, 142.13, 137.08, 135.89, 134.81, 132.08, 131.51, 128.95, 128.83, 128.11, 126.69, 126.42, 26.66. **HRMS** calculated for $C_{18}H_{17}O$ $[M+H]^+$ 249.1274, found 249.1276.

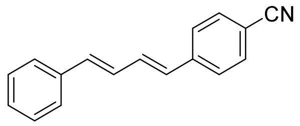

4-((1*E*,3*E*)-4-Phenylbuta-1,3-dien-1-yl)benzonitrile (5y): Prepared according to the general procedure A, known compound,[11] white solid, 35.1 mg, 76% yield, R_f = 0.3 (PE/DCM = 2/1), **^{1}H NMR** (400 MHz, Chloroform-*d*) δ 7.57 (d, *J* = 8.2 Hz, 2H), 7.47 (d, *J* = 8.7 Hz, 2H), 7.44 (d, J = 7.4 Hz, 2H), 7.34 (t, *J* = 7.5 Hz, 2H), 7.26 (t, J = 7.3 Hz, 1H), 7.03 (dd, *J* = 15.1, 10.6 Hz, 1H), 6.94 (dd, *J* = 15.0, 10.6 Hz, 1H), 6.75 (d, *J* = 15.1 Hz, 1H), 6.62 (d, *J* = 15.1 Hz, 1H); **^{13}C NMR** (100 MHz, Chloroform-*d*) δ 141.94, 136.86, 135.57, 132.92, 132.52, 130.63, 128.86, 128.41, 128.32, 126.77, 126.74, 119.17, 110.42. **HRMS** calculated for $C_{17}H_{13}N$ $[M]^+$ 231.1043, found 231.1034.

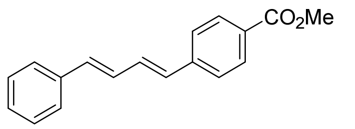

Methyl 4-((1*E*,3*E*)-4-phenylbuta-1,3-dien-1-yl)benzoate (5z): Prepared according to the general procedure A, white solid, 44.1 mg (**5x**+**5x'**), 84% yield (**5x**/**5x'** = 84/16), R_f = 0.3 (PE/DCM = 2/1), **^{1}H NMR** (400 MHz, Chloroform-*d*) δ 8.00 (d, *J* = 8.4 Hz, 2H), 7.48 (d, *J* = 8.5 Hz, 2H), 7.46 (d, *J* = 7.6 Hz, 2H), 7.35 (t, *J* = 7.5 Hz, 2H), 7.26 (t, *J* = 7.3 Hz, 1H), 7.06 (dd, *J* = 15.2, 10.6 Hz, 1H), 6.97 (dd, *J* = 15.0, 10.6 Hz, 1H), 6.74 (d, *J* = 15.1 Hz, 1H), 6.68 (d, *J* = 15.1 Hz, 1H), 3.92 (s, 3H); **^{13}C NMR** (100 MHz, Chloroform-*d*) δ 166.97, 141.97, 137.14, 134.63, 131.87, 131.64, 130.11, 128.87, 128.83, 128.07, 126.69, 126.26, 52.17. **HRMS** calculated for $C_{18}H_{17}O_2$ $[M+H]^+$ 265.1223, found 265.1223.

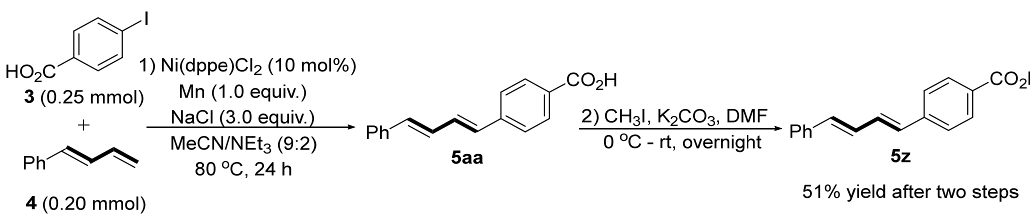

In a glove box, a sealed tube was charged with iodoarene **3** (0.25 mmol), Ni(dppe)Cl_2 (0.02 mmol, 10 mol%), Mn (0.20 mmol), NaCl (0.60 mmol), 1,3-diene **4** (0.20 mmol), MeCN (0.5 mL), NEt_3 (112 uL) at room temperature. The reaction tube was sealed with a Teflon screw cap, removed from the glove box. Then, the reaction mixture was stirred at 80 ºC for 24 hours. And the reaction mixture was quenched with 1M HCl aq. (2 mL) and extracted with DCM (5 mL × 3). The combined organic layers were dried over Na_2SO_4, concentrated in vacuo and dissolved in DMF (5 mL). Without further purification, K_2CO_3 (0.60 mmol) and CH_3I (0.50 mmol) were added into the solution at 0 ºC. The reaction mixture was then warmed to room temperature and stirred for overnight.[12] And the crude reaction mixture was purified by column chromatography on silica gel to afford the corresponding product **5x.**

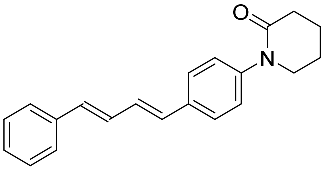

1-(4-((1*E*,3*E*)-4-Phenylbuta-1,3-dien-1-yl)phenyl)piperidin-2-one (5bb): Prepared according to the general procedure A, white solid, melting point: 198-200 ºC, 48.3 mg, 80% yield, R_f = 0.3 (MeOH/DCM = 1/40), **^{1}H NMR** (400 MHz, Chloroform-*d*) δ 7.49-7.38 (m, 4H), 7.32 (t, *J* = 7.6 Hz, 2H), 7.26-7.17 (m, 3H), 6.99-6.84 (m, 2H), 6.71-6.56 (m, 2H), 3.67-3.54 (m, 2H), 2.55 (t, *J* = 5.8 Hz, 2H), 1.97-1.85 (m, 4H); **^{13}C NMR** (100 MHz, Chloroform-*d*) δ 170.03, 142.56, 137.31, 135.74, 132.98, 132.05, 129.50, 129.18, 128.68, 127.61, 126.99, 126.41, 126.24, 51.46, 32.95, 23.54, 21.44. **HRMS** calculated for $C_{21}H_{22}NO$ $[M+H]^+$ 304.1696, found 304.1698.

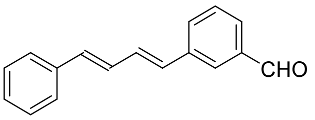

3-((1*E*,3*E*)-4-Phenylbuta-1,3-dien-1-yl)benzaldehyde (5cc): Prepared according to the general procedure A, yellow solid, melting point: 128-130 ºC, 31.1 mg, 67% yield, R_f = 0.3 (PE/DCM = 2/1), **^{1}H NMR** (400 MHz, Chloroform-*d*) δ 10.01 (s, 1H), 7.92 (s, 1H), 7.72 (d, *J* = 7.5 Hz, 1H), 7.66 (d, *J* = 7.8 Hz, 1H), 7.49 (d, *J* = 7.6 Hz, 1H), 7.45 (d, *J* = 8.6 Hz, 2H), 7.34 (t, *J* = 7.6 Hz, 2H), 7.25 (t, *J* = 7.6 Hz, 1H), 7.03 (dd, *J* = 15.1, 10.5 Hz, 1H), 6.95 (dd, *J* = 15.0, 10.5 Hz, 1H), 6.78-6.63 (m, 2H); **^{13}C NMR** (100 MHz, Chloroform-*d*) δ 192.39, 138.49, 137.14, 136.91, 134.30, 132.17, 131.10, 129.44, 128.82, 128.80, 128.75, 128.02, 127.21, 126.65. **HRMS** calculated for $C_{17}H_{15}O$ $[M+H]^+$ 235.1117, found 235.1114.

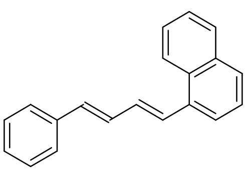

1-((1*E*,3*E*)-4-Phenylbuta-1,3-dien-1-yl)naphthalene (5dd): Prepared according to the general procedure A, known compound,[13] white solid, 39.5 mg, 77% yield, R_f = 0.3 (PE), **^{1}H NMR** (400 MHz, Chloroform-*d*) δ 8.25 (d, *J* = 8.3 Hz, 1H), 7.91 (d, *J* = 7.6 Hz, 1H), 7.83 (d, *J* = 8.2 Hz, 1H), 7.78 (d, *J* = 7.2 Hz, 1H), 7.63-7.46 (m, 6H), 7.42 (t, *J* = 7.6 Hz, 2H), 7.31 (t, *J* = 7.3 Hz, 1H), 7.17 (dd, *J* = 15.1, 10.6 Hz, 1H), 7.08 (dd, *J* = 14.9, 10.6 Hz, 1H), 6.77 (d, *J* = 15.1 Hz, 1H); **^{13}C NMR** (100 MHz, Chloroform-*d*) δ 137.45, 134.74, 133.91, 133.23, 132.07, 131.23, 129.63, 129.52,

128.81, 128.75, 128.10, 127.76, 126.57, 126.20, 125.92, 125.76, 123.69, 123.37. **HRMS** calculated for $C_{20}H_{16}$ $[M]^+$ 256.1247, found 256.1238.

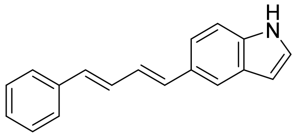

5-((1*E*,3*E*)-4-Phenylbuta-1,3-dien-1-yl)-1H-indole (5ee): Prepared according to the general procedure A, white solid, melting point: 147-149 °C, 29.4 mg, 60% yield, R_f = 0.3 (PE/DCM = 2/1, recrystallization), **^{1}H NMR** (400 MHz, Chloroform-*d*) δ 8.09 (s, 1H), 7.68 (s, 1H), 7.44 (d, *J* = 7.5 Hz, 2H), 7.40-7.27 (m, 4H), 7.27-7.13 (m, 3H), 7.05-6.88 (m, 2H), 6.80 (d, *J* = 14.5 Hz, 1H), 6.62 (d, *J* = 14.5 Hz, 1H), 6.54 (s, 1H); **^{13}C NMR** (100 MHz, Chloroform-*d*) δ 137.89, 135.71, 134.60, 131.17, 130.05, 129.73, 128.75, 128.38, 127.28, 126.91, 126.34, 124.89, 120.77, 119.56, 111.43, 103.17. **HRMS** calculated for $C_{18}H_{16}N$ $[M+H]^+$ 246.1277, found 246.1286.

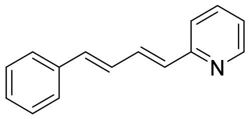

2-((1*E*,3*E*)-4-Phenylbuta-1,3-dien-1-yl)pyridine (5ff): Prepared according to the general procedure A, known compound,[14] white solid, 22.1 mg, 53% yield, R_f = 0.7 (PE/EA = 5/1), **^{1}H NMR** (400 MHz, Chloroform-*d*) δ 8.58 (d, *J* = 3.9 Hz, 1H), 7.63 (td, *J* = 7.7, 1.8 Hz, 1H), 7.49-7.40 (m, 3H), 7.37-7.28 (m, 3H), 7.26 (t, *J* = 7.3 Hz, 1H), 7.14-7.07 (m, 1H), 7.00 (dd, *J* = 15.3, 10.9 Hz, 1H), 6.79 (d, *J* = 15.6 Hz, 1H), 6.73 (d, *J* = 15.3 Hz, 1H); **^{13}C NMR** (100 MHz, Chloroform-*d*) δ 155.80, 149.82, 137.19, 136.56, 135.47, 133.37, 132.00, 128.81, 128.65, 128.09, 126.78, 122.09, 122.01.

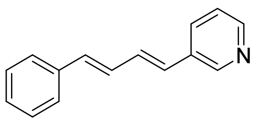

3-((1*E*,3*E*)-4-Phenylbuta-1,3-dien-1-yl)pyridine (5gg): Prepared according to the general procedure A, known compound,[15] white solid, 26.7 mg, 64% yield, R_f = 0.2 (PE/EA = 5/1), **^{1}H NMR** (400 MHz, Chloroform-*d*) δ 8.66 (d, *J* = 2.0 Hz, 1H), 8.46 (dd, *J* = 4.7, 1.3 Hz, 1H), 7.76 (d, *J* = 8.0 Hz, 1H), 7.46 (d, *J* = 7.4 Hz, 2H), 7.35 (t, *J* = 7.6 Hz, 2H), 7.26 (t, *J* = 7.6 Hz, 2H), 7.06 -6.92 (m, 2H), 6.73 (d, *J* = 14.7 Hz, 1H), 6.64 (d, *J* = 14.9 Hz, 1H); **^{13}C NMR** (100 MHz, Chloroform-*d*) δ 148.50, 137.09, 134.39, 133.20, 132.63, 131.39, 128.85, 128.82, 128.72, 128.09, 126.68, 123.68.

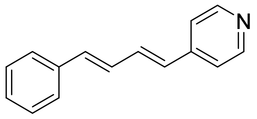

4-((1*E*,3*E*)-4-Phenylbuta-1,3-dien-1-yl)pyridine (5hh): Prepared according to the general procedure A, known compound,[16] white solid, 24.0 mg, 58% yield, R_f = 0.2 (PE/EA = 4/1), **^{1}H NMR** (400 MHz, Chloroform-*d*) δ 8.54 (d, *J* = 5.3 Hz, 2H), 7.46 (d, *J* = 7.6 Hz, 2H), 7.35 (t, *J* = 7.5 Hz, 2H), 7.32-7.21 (m, 3H), 7.12 (dd, *J* = 15.4, 10.5 Hz, 1H), 6.95 (dd, *J* = 15.4, 10.6 Hz, 1H), 6.77 (d, *J* = 15.5 Hz, 1H), 6.57 (d, *J* = 15.5 Hz, 1H); **^{13}C NMR** (100 MHz, Chloroform-*d*) δ 150.22, 144.73, 136.83, 135.94, 133.71, 129.84, 128.88, 128.39, 128.25, 126.83, 120.74.

3.3 General procedure B: Ni-catalyzed branched Heck reaction of 1,3-dienes

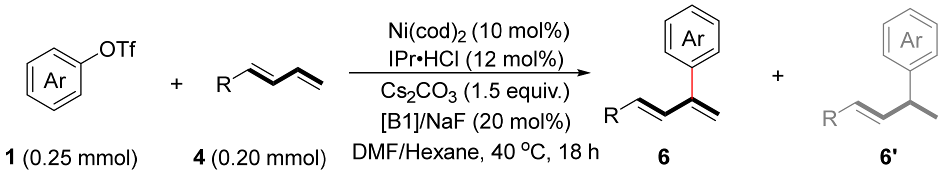

In a glove box, $Ni(cod)_2$ (0.02 mmol, 10 mol%), IPr·HCl (0.024 mmol, 12 mol%), Cs_2CO_3 (0.30 mmol, 1.5 equiv.) and **B1**/NaF (0.04 mmol, 20 mol%) were added to DMF (0.30 mL) in sequence and stirred at room temperature for 30 min. Then, the mixture of aryl triflate **1** (0.30 mmol), 1,3-diene **4** (0.20 mmol) in hexane (0.20 mL) was added into the reaction solvent. The reaction tube was sealed with a Teflon screw cap, removed from the glove box. And the reaction mixture was stirred at 40 ºC for 18 hours. The selectivity was determined by ^{1}H NMR analysis. And the crude reaction mixture was purified by column chromatography on silica gel or recrystallization using petroleum ether and ethyl acetate to afford the corresponding product **6**.

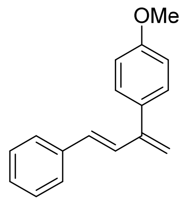

(*E*)-1-Methoxy-4-(4-phenylbuta-1,3-dien-2-yl)benzene (6a): Prepared according to the general procedure B (**1** (0.20 mmol), **4** (0.25 mmol), **B2** was used instead of **B1**), white solid, melting point: 81-83 ºC, 33.8 mg, 72% yield, R_f = 0.5 (PE/EA = 50/1), **^{1}H NMR** (400 MHz, Chloroform-*d*) δ 7.41 (d, *J* = 7.5 Hz, 2H), 7.36-7.28 (m, 4H), 7.23 (t, *J* = 7.3 Hz, 1H), 7.04 (d, *J* = 16.1 Hz, 1H), 6.93 (d, *J* = 8.6 Hz, 2H), 6.52 (d, *J* = 16.1 Hz, 1H), 5.37 (s, 1H), 5.21 (d, *J* = 1.4 Hz, 1H), 3.85 (s, 3H);c **^{13}C NMR** (100 MHz, Chloroform-*d*) δ 159.35, 147.78, 137.46, 132.80, 131.99, 130.84, 129.78, 128.83, 127.84, 126.78, 116.89, 113.83, 55.55. **HRMS** calculated for $C_{17}H_{17}O$ $[M+H]^+$ 237.1724, found 237.1723.

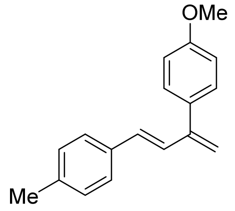

(*E*)-1-Methoxy-4-(4-(*p*-tolyl)buta-1,3-dien-2-yl)benzene (6b): Prepared according to the general procedure B (**1** (0.20 mmol), **4** (0.25 mmol), **B2** was used instead of **B1**), white solid, melting point: 92-93 ºC, 35.1 mg, 70% yield, R_f = 0.4 (PE/EA = 50/1), **^{1}H NMR** (400 MHz, Chloroform-*d*) δ 7.36-7.28 (m, 4H), 7.13 (d, *J* = 8.0 Hz, 2H), 7.00 (d, *J* = 16.1 Hz, 1H), 6.93 (d, *J* = 8.7 Hz, 2H), 6.51 (d, *J* = 16.1 Hz, 1H), 5.35 (s, 1H), 5.19 (d, *J* = 1.6 Hz, 1H), 3.86 (s, 3H), 2.35 (s, 3H); **^{13}C NMR** (100 MHz, Chloroform-*d*) δ 159.25, 147.81, 137.63, 134.60, 132.86, 131.83, 129.78, 129.67, 129.45, 126.61, 116.21, 113.72, 55.43, 21.37. **HRMS** calculated for $C_{18}H_{19}O$ $[M+H]^+$ 251.1430, found 251.1431.

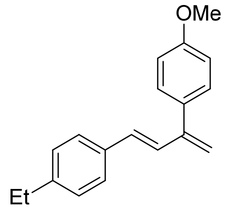

(*E*)-1-Ethyl-4-(3-(4-methoxyphenyl)buta-1,3-dien-1-yl)benzene (6c): Prepared according to the general B (**1** (0.20 mmol), **4** (0.25 mmol), **B2** was used instead of **B1**), white solid, melting point: 87-89 ºC, 36.1 mg, 68% yield, R_f = 0.4 (PE/EA = 50/1), **^{1}H NMR** (400 MHz, Chloroform-*d*) δ 7.37-7.29 (m, 4H), 7.15 (d, *J* = 8.1 Hz, 2H), 7.00 (d, *J* = 16.1 Hz, 1H), 6.93 (d, *J* = 8.7 Hz, 2H), 6.50 (d, *J* = 16.1 Hz, 1H), 5.34 (s, 1H), 5.18 (d, *J* = 1.5 Hz, 1H), 3.85 (s, 3H), 2.64 (q, *J* = 7.6 Hz, 2H), 1.23 (t, *J* = 7.6 Hz, 3H); **^{13}C NMR** (100 MHz, Chloroform-*d*) δ 159.25, 147.82, 144.06, 132.88, 131.86, 129.85, 129.67, 128.26, 126.69, 116.20, 113.72, 55.44, 28.77, 15.66. **HRMS** calculated for $C_{19}H_{21}O$ $[M+H]^+$ 265.1587, found 265.1585.

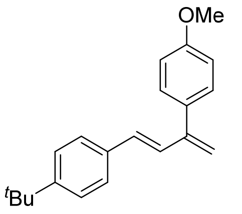

(*E*)-1-(*tert*-Butyl)-4-(3-(4-methoxyphenyl)buta-1,3-dien-1-yl)benzene (6d): Prepared according to the general procedure B (**1** (0.20 mmol), **4** (0.25 mmol), **B2** was used instead of **B1**), white solid, melting point: 121-123 °C, 47.4 mg, 81% yield, R_f = 0.4 (PE/EA = 50/1), **^{1}H NMR** (400 MHz, Chloroform-*d*) 7.40-7.34 (m, 4H), 7.33 (d, *J* = 8.7 Hz, 2H), 7.02 (d, *J* = 16.1 Hz, 1H), 6.94 (d, *J* = 8.7 Hz, 2H), 6.53 (d, *J* = 16.1 Hz, 1H), 5.36 (s, 1H), 5.19 (d, *J* = 1.6 Hz, 1H), 3.86 (s, 3H), 1.33 (s, 9H); **^{13}C NMR** (100 MHz, Chloroform-*d*) δ 159.25, 150.91, 147.81, 134.61, 132.88, 131.73, 129.99, 129.67, 126.43, 125.67, 116.19, 113.72, 55.43, 34.74, 31.42. **HRMS** calculated for $C_{21}H_{25}O$ $[M+H]^+$ 293.1900, found 293.1899.

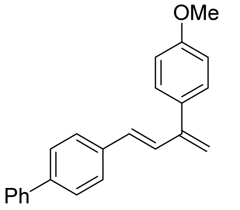

(*E*)-4-(3-(4-Methoxyphenyl)buta-1,3-dien-1-yl)-1,1'-biphenyl (6e): Prepared according to the general procedure B (**1** (0.20 mmol), **4** (0.25 mmol), **B2** was used instead of **B1**), white solid, melting point: 148-150 °C, 36.7 mg, 59% yield, R_f = 0.4 (PE/DCM = 10/1), **^{1}H NMR** (400 MHz, Chloroform-*d*) δ 7.62 (d, *J* = 7.4 Hz, 2H), 7.58 (d, *J* = 8.0 Hz, 2H), 7.51-7.40 (m, 4H), 7.40-7.30 (m, 3H), 7.10 (d, *J* = 16.1 Hz, 1H), 6.96 (d, *J* = 8.2 Hz, 2H), 6.57 (d, *J* = 16.0 Hz, 1H), 5.40 (s, 1H), 5.24 (s, 1H), 3.87 (s, 3H); **^{13}C NMR** (100 MHz, Chloroform-*d*) δ 159.30, 147.74, 140.78, 140.43, 136.45, 132.72, 131.43, 130.83, 129.70, 128.92, 127.45, 127.40, 127.13, 127.03, 116.85, 113.77, 55.45. **HRMS** calculated for $C_{23}H_{21}O$ $[M+H]^+$ 313.1587, found 313.1586.

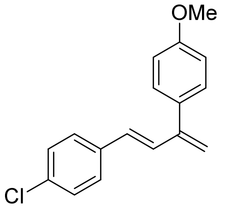

(*E*)-1-Chloro-4-(3-(4-methoxyphenyl)buta-1,3-dien-1-yl)benzene (6f): Prepared according to the general procedure B (**1** (0.20 mmol), **4** (0.25 mmol), **B2** was used instead of **B1**), colorless oil, 35.9 mg, 66% yield, R_f = 0.4 (PE/EA = 50/1), **^{1}H NMR** (400 MHz, Chloroform-*d*) δ 7.38-7.26 (m, 6H), 7.00 (d, *J* = 16.1 Hz, 1H), 6.93 (d, *J* = 8.6 Hz, 2H), 6.46 (d, *J* = 16.1 Hz, 1H), 5.37 (s, 1H), 5.23 (s, 1H), 3.85 (s, 3H); **^{13}C NMR** (100 MHz, Chloroform-*d*) δ 159.34, 147.51, 135.91, 132.49, 131.40, 130.58, 129.66, 128.89, 127.84, 117.32, 113.80, 55.45. **HRMS** calculated for $C_{17}H_{16}Cl^{35}O$ $[M+H]^+$ 271.0884, found 271.0891; for $C_{17}H_{16}Cl^{37}O$ $[M+H]^+$ 273.0855, found 273.0861.

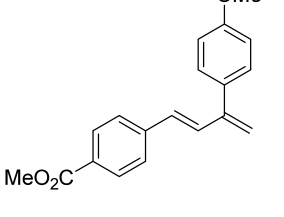

Methyl (*E*)-4-(3-(4-methoxyphenyl)buta-1,3-dien-1-yl)benzoate (6g): Prepared according to the general procedure B, an inseparable mixture of **6g+6g"** (**6g/6g"** = 11:1), 27.4 mg, 47% yield, R_f = 0.3 (PE/EA = 20/1), **^{1}H NMR** (400 MHz, Chloroform-*d*) δ 7.97 (d, *J* = 8.2 Hz, 2H), 7.44 (d, *J* = 8.2 Hz, 2H), 7.30 (d, *J* = 8.5 Hz, 2H), 7.13 (d, *J* = 16.1 Hz, 1H), 6.93 (d, *J* = 8.6 Hz, 2H), 6.53 (d, *J* = 16.1 Hz, 1H), 5.42 (s, 1H), 5.27 (s, 1H), 3.91 (s, 3H), 3.85 (s, 3H); **^{13}C NMR** (100 MHz, Chloroform-*d*) δ 166.88, 159.26, 147.34, 141.79, 133.16, 132.15, 130.69, 129.93, 129.54, 128.90, 126.38, 118.18, 113.70, 55.32, 52.06. **HRMS** calculated for $C_{19}H_{19}O_3$ $[M+H]^+$ 295.1329, found 295.1330.

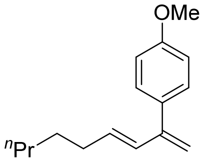

(*E*)-1-Methoxy-4-(nona-1,3-dien-2-yl)benzene (6h): Prepared according to the general procedure B, colorless oil, 21.8 mg, 47% yield, R_f = 0.5 (PE/EA = 50/1), **^{1}H NMR** (400 MHz, Chloroform-*d*) δ 7.27 (d, *J* = 8.8 Hz, 2H), 6.89 (d, *J* = 8.8 Hz, 2H), 6.29 (d, *J* = 15.6 Hz, 1H), 5.68 (dt, *J* = 15.5, 7.0 Hz, 1H), 5.13 (d, *J* = 1.4 Hz,

1H), 5.02 (d, J = 1.8 Hz, 1H), 3.83 (s, 3H), 2.12 (q, J = 6.9 Hz, 2H), 1.41-1.27 (m, 6H), 0.89 (t, J = 6.9 Hz, 3H); **^{13}C NMR** (100 MHz, Chloroform-*d*) δ 159.05, 147.66, 134.61, 133.27, 131.56, 129.43, 113.79, 113.53, 55.39, 33.00, 31.62, 29.06, 22.67, 14.21. **HRMS** calculated for $C_{16}H_{23}O$ $[M+H]^+$ 231.1743, found 231.1737.

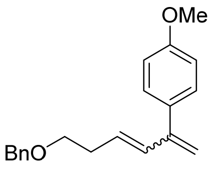

1-(6-(Benzyloxy)hexa-1,3-dien-2-yl)-4-methoxybenzene (6i): Prepared according to the general procedure B, colorless oil, 26.1 mg, 44% yield (*E/Z* = 3:1), R_f = 0.4 (PE/EA = 50/1), **^{1}H NMR** (400 MHz, Chloroform-*d*) δ 7.36–7.22 (m, 7H), 6.92–6.80 (m, 2H), 6.36 (d, J = 15.7 Hz, 0.7H), 6.20 (d, J = 11.5 Hz, 0.3H), 5.77 (dt, J = 11.6, 7.2 Hz, 0.3H), 5.68 (dt, J = 15.5, 7.0 Hz, 0.7H), 5.46 (d, J = 1.6 Hz, 0.3H), 5.14 (s, 0.7H), 5.08 (s, 0.3H), 5.05 (d, J = 1.7 Hz, 0.7H), 4.54-4.45 (s, 2H), 3.85-3.78 (s, 3H), 3.57–3.45 (t, J = 6.84 Hz, 2H), 2.54–2.36 (m, 2H); **^{13}C NMR** (100 MHz, Chloroform-*d*) δ 159.34, 159.09, 147.41, 143.67, 138.55, 133.52, 133.23, 132.98, 130.26, 130.21, 129.45, 128.50, 128.47, 127.79, 127.69, 114.56, 113.72, 113.57, 113.47, 73.02, 70.11, 69.75, 55.40, 33.45. **HRMS** calculated for $C_{20}H_{23}O_2$ $[M+H]^+$ 295.1693, found 295.1702.

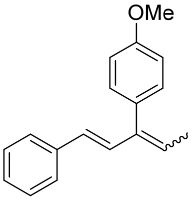

1-Methoxy-4-((1*E*)-1-phenylpenta-1,3-dien-3-yl)benzene (6j): Prepared according to the general procedure B (**1** (0.20 mmol), **4** (0.25 mmol), **B2** was used instead of **B1)**, colorless oil, 13.0 mg, 26% yield (*E/Z* or *Z/E* = 2:1), R_f = 0.6 (PE/EA = 50/1), **^{1}H NMR** (400 MHz, Chloroform-*d*) δ 7.44-7.36 (m, 2H), 7.34-7.28 (m, 3H), 7.24-7.09 (m, 3H), 6.97 (d, J = 8.7 Hz, 0.7H), 6.90 (d, J = 8.8 Hz, 1.5H), 6.44-6.39 (m, 0.7H), 6.05 (d, J = 16.0 Hz, 0.4H), 5.94 (q, J = 7.1 Hz, 0.3H), 5.65 (q, J = 7.2 Hz, 0.7H), 3.89-3.82 (m, 3H), 1.99 (d, J = 7.2 Hz, 2H), 1.66 (d, J = 7.1 Hz, 1H); **^{13}C NMR** (100 MHz, Chloroform-*d*) δ 140.10, 137.96, 134.76, 133.66, 131.74, 130.11, 128.71, 128.61, 127.51, 127.20, 126.56, 126.34, 125.79, 113.81, 113.60, 55.42, 15.34, 14.23. **HRMS** calculated for $C_{18}H_{19}O$ $[M+H]^+$ 251.1430, found 251.1432.

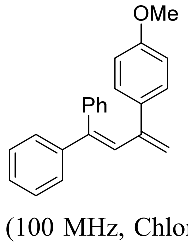

(3-(4-Methoxyphenyl)buta-1,3-diene-1,1-diyl)dibenzene (6k): Prepared according to the general procedure B(**1** (0.20 mmol), **4** (0.25 mmol), **B2** was used instead of **B1)**, known compound,[17] yellow solid, 51.8 mg, 83% yield, R_f = 0.4 (PE/EA = 50/1), **^{1}H NMR** (400 MHz, Chloroform-*d*) δ 7.42-7.30 (m, 7H), 7.26-7.16 (m, 5H), 6.82 (d, J = 8.8 Hz, 2H), 6.76 (s, 1H), 5.35 (d, J = 1.0, 1H), 4.97 (s, 1H), 3.81 (s, 3H); **^{13}C NMR** (100 MHz, Chloroform-*d*) δ 159.22, 144.64, 144.61, 143.27, 140.24, 133.35, 130.20, 128.75, 128.28, 128.07, 128.02, 127.88, 127.67, 127.11, 115.69, 113.61, 55.41. **HRMS** calculated for $C_{23}H_{21}O$ $[M+H]^+$ 313.1587, found 313.1587.

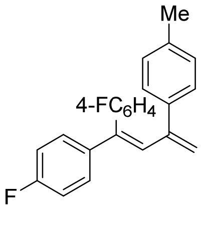

4,4'-(3-(*p*-Tolyl)buta-1,3-diene-1,1-diyl)bis(fluorobenzene) (6l): Prepared according to the general procedure B (**1** (0.20 mmol), **4** (0.25 mmol), **B2** was used instead of **B1)**, colorless oil, 57.4 mg, 86% yield, R_f = 0.5 (PE), **^{1}H NMR** (400 MHz, Chloroform-*d*) δ 7.31-7.22 (m, 4H), 7.11-7.03 (m, 4H), 7.00 (t, J = 8.7 Hz, 2H), 6.86 (t, J = 8.8 Hz, 2H), 6.66 (s, 1H), 5.38 (d, J = 1.2 Hz, 1H), 4.98 (t, J = 1.3 Hz, 1H), 2.31 (s, 3H); **^{13}C NMR** (100 MHz, Chloroform-*d*) δ 162.60 (d, J = 246.4 Hz), 162.13 (d, J = 246.4 Hz), 145.03, 142.54, 139.23 (d, J = 3.2 Hz), 137.58, 137.52, 135.92 (d, J = 3.4 Hz), 131.81 (d, J = 8.0 Hz), 129.66 (d, J = 8.0 Hz), 128.98, 128.85, 126.61, 115.24 (d, J = 21.5 Hz), 115.04

(d, J = 21.5 Hz), 21.24; **^{19}F NMR** (376 MHz, Chloroform-*d*) δ -114.53, -114.91. **HRMS** calculated for $C_{23}H_{29}F_2$ $[M]^+$ 332.1371, found 332.1370.

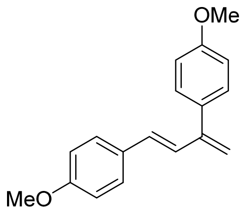

(*E*)-4,4'-(Buta-1,3-diene-1,3-diyl)bis(methoxybenzene) (6m): Prepared according to the general procedure B, white solid, melting point: 105-106 °C , 39.4 mg, 74% yield, R_f = 0.4 (PE/EA = 20/1), **^{1}H NMR** (400 MHz, Chloroform-*d*) δ 7.42-7.29 (m, 4H), 6.98-6.90 (m, 3H), 6.86 (d, J = 8.7 Hz, 2H), 6.47 (d, J = 16.1 Hz, 1H), 5.33 (s, 1H), 5.16 (d, J = 1.5 Hz, 1H), 3.85 (s, 3H), 3.82 (s, 3H); **^{13}C NMR** (100 MHz, Chloroform-*d*) δ 159.36, 159.17, 147.82, 132.89, 131.37, 130.13, 129.68, 128.70, 127.88, 115.81, 114.15, 113.67, 55.42. **HRMS** calculated for $C_{18}H_{19}O_2$ $[M+H]^+$ 267.1380, found 267.1381.

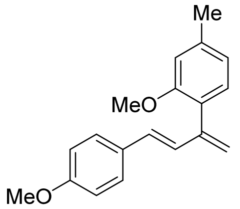

(*E*)-2-Methoxy-1-(4-(4-methoxyphenyl)buta-1,3-dien-2-yl)-4-methylbenzene (6n): Prepared according to the general procedure B, colorless oil, 43.8 mg, 78% yield, R_f = 0.3 (PE/EA = 50/1), **^{1}H NMR** (400 MHz, Chloroform-*d*) δ 7.31 (d, J = 8.5 Hz, 2H), 7.08 (d, J = 7.4 Hz, 1H), 6.94 (d, J = 16.0 Hz, 1H), 6.89-6.76 (m, 4H), 6.14 (d, J = 16.0 Hz, 1H), 5.49 (s, 1H), 5.15 (s, 1H), 3.85-3.77 (m, 6H), 2.43 (s, 3H); **^{13}C NMR** (100 MHz, Chloroform-*d*) δ 159.21, 156.80, 145.83, 138.89, 130.83, 130.69, 130.38, 128.70, 127.87, 126.60, 121.28, 117.87, 114.06, 112.07, 55.82, 55.38, 21.76. **HRMS** calculated for $C_{19}H_{21}O_2$ $[M+H]^+$ 281.1536, found 281.1542.

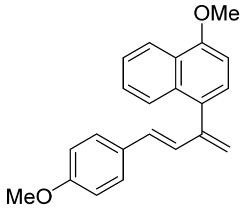

(*E*)-1-Methoxy-4-(4-(4-methoxyphenyl)buta-1,3-dien-2-yl)naphthalene (6o): Prepared according to the general procedure B, colorless oil, 35.0 mg, 55% yield, R_f = 0.3 (PE/EA = 50/1), **^{1}H NMR** (400 MHz, Chloroform-*d*) δ 8.33 (d, J = 7.7 Hz, 1H), 7.89 (d, J = 8.0 Hz, 1H), 7.51-7.41 (m, 2H), 7.29 (d, J = 7.6 Hz, 1H), 7.23 (d, J = 8.0 Hz, 2H), 7.09 (d, J = 15.9 Hz, 1H), 6.86 (d, J = 7.7 Hz, 1H), 6.80 (d, J = 7.9 Hz, 2H), 6.01 (d, J = 15.9 Hz, 1H), 5.64 (s, 1H), 5.23 (s, 1H), 4.05 (s, 3H), 3.78 (s, 3H); **^{13}C NMR** (100 MHz, Chloroform-*d*) δ 159.33, 155.09, 147.46, 132.95, 131.89, 130.69, 130.09, 129.89, 127.87, 126.68, 126.48, 126.35, 125.16, 122.07, 119.06, 114.08, 103.44, 55.66, 55.40. **HRMS** calculated for $C_{22}H_{21}O_2$ $[M+H]^+$ 317.1536, found 317.1536.

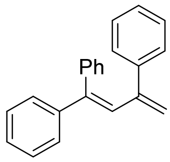

Buta-1,3-diene-1,1,3-triyltribenzene (6p): Prepared according to the general procedure B (**1** (0.25 mmol)), colorless oil, 37.9 mg, 67% yield, R_f = 0.5, (PE), **^{1}H NMR** (400 MHz, Chloroform-*d*) δ 7.43-7.38 (m, 2H), 7.36-7.27 (m, 5H), 7.25-7.14 (m, 8H), 6.74 (s, 1H), 5.39 (s, 1H), 5.03 (s, 1H); **^{13}C NMR** (100 MHz, Chloroform-*d*) δ 145.29, 144.70, 143.12, 140.73, 140.07, 130.15, 128.34, 128.19, 128.11, 127.98, 127.93, 127.62, 127.42, 127.04, 126.70, 117.39. **HRMS** calculated for $C_{22}H_{18}$ $[M]^+$ 282.1403, found 282.1402.

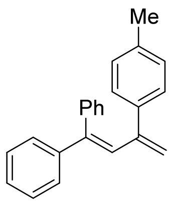

(3-(*p*-Tolyl)buta-1,3-diene-1,1-diyl)dibenzene (6q): Prepared according to the general procedure B (**1** (0.25 mmol)), colorless oil, 56.7 mg, 96% yield, R_f = 0.5, (PE), **^{1}H NMR** (400 MHz, Chloroform-*d*) δ 7.40-7.30 (m, 7H), 7.26-7.18 (m, 5H), 7.10 (d, J = 8.0 Hz, 2H), 6.75 (s, 1H), 5.40 (d, J = 1.0 Hz, 1H), 4.98 (s, 1H), 2.35 (s, 3H); **^{13}C NMR** (100 MHz, Chloroform-*d*) δ 144.99, 144.68, 143.26, 140.28, 137.96, 137.38, 130.21, 128.97, 128.65, 128.29, 128.07, 127.68, 127.09, 126.59, 116.43, 21.25. **HRMS** calculated for

$C_{23}H_{20}$ $[M]^+$ 296.1560, found 296.1557.

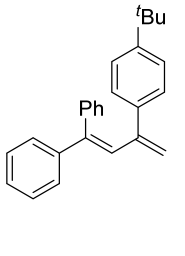

(3-(4-(*tert*-Butyl)phenyl)buta-1,3-diene-1,1-diyl)dibenzene (6r): Prepared according to the general procedure B (**1** (0.25 mmol)), an inseparable mixture of **6r+6r'** (**6r/6r'** = 9:1), 60.6 mg, 90% yield, R_f = 0.5 (PE), **^{1}H NMR** (700 MHz, Chloroform-*d*) δ 7.39-7.28 (m, 9H), 7.22-7.15 (m, 5H), 6.76 (s, 1H), 5.40 (s, 1H), 5.01 (s, 1H), 1.33 (s, 9H); **^{13}C NMR** (100 MHz, Chloroform-*d*) δ 150.52, 145.05, 144.58, 143.31, 140.25, 137.94, 130.27, 128.69, 128.28, 128.06, 128.02, 127.66, 127.08, 126.53, 125.14, 116.82, 34.62, 31.43. **HRMS** calculated for $C_{26}H_{26}$ $[M]^+$ 338.2029, found 338.2027.

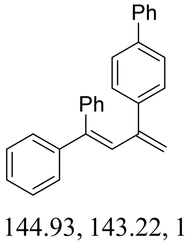

4-(4,4-Diphenylbuta-1,3-dien-2-yl)-1,1'-biphenyl (6s): Prepared according to the general procedure B (**1** (0.25 mmol), **4** (0.20 mmol), **B1** was used instead of **B2**), colorless oil, 40.0 mg, 56% yield, R_f = 0.4 (PE), **^{1}H NMR** (400 MHz, Chloroform-*d*) δ 7.58 (d, *J* = 7.7 Hz, 2H), 7.49-7.42 (m, 6H), 7.40-7.29 (m, 7H), 7.23-7.14 (m, 4H), 6.79 (s, 1H), 5.47 (s, 1H), 5.08 (s, 1H); **^{13}C NMR** (100 MHz, Chloroform-*d*) δ 145.00, 144.93, 143.22, 140.98, 140.34, 140.17, 139.78, 130.28, 128.88, 128.40, 128.33, 128.11, 128.06, 127.77, 127.37, 127.24, 127.17, 127.13, 126.95, 117.50. **HRMS** calculated for $C_{28}H_{22}$ $[M]^+$ 358.1716, found 358.1711.

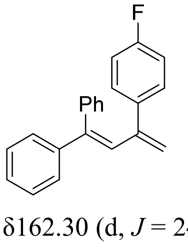

(3-(4-Fluorophenyl)buta-1,3-diene-1,1-diyl)dibenzene (6t): Prepared according to the general procedure B (**1** (0.25 mmol), **4** (0.20 mmol), **B1** was used instead of **B2**), colorless oil, 34.9 mg, 58% yield, R_f = 0.5 (PE), **^{1}H NMR** (400 MHz, Chloroform-*d*) δ 7.36-7.27 (m, 7H), 7.21-7.07 (m, 5H), 6.88 (t, *J* = 8.8 Hz, 1H), 6.74 (d, *J* = 1.0 Hz, 1H), 5.34 (d, *J* = 1.1 Hz, 1H), 5.08 (s, 1H); **^{13}C NMR** (100 MHz, Chloroform-*d*) δ162.30 (d, *J* = 246.2 Hz), 144.97, 144.70, 143.13, 140.00, 136.86 (d, *J* = 3.3 Hz), 130.25, 128.51 (d, *J* = 8.0 Hz), 128.32, 128.25, 128.11, 128.00, 127.83, 127.25, 117.67, 114.91 (d, *J* = 21.4 Hz). **^{19}F NMR** (376 MHz, Chloroform-*d*) δ -115.30. **HRMS** calculated for $C_{22}H_{17}F$ $[M]^+$ 300.1309, found 300.1307. [1]

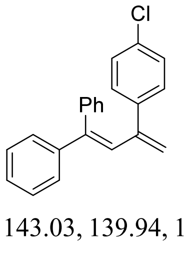

(3-(4-Chlorophenyl)buta-1,3-diene-1,1-diyl)dibenzene (6u): Prepared according to the general procedure B (**1** (0.25 mmol)), colorless oil, 39.5 mg, 62% yield, R_f = 0.5 (PE), **^{1}H NMR** (400 MHz, Chloroform-*d*) δ 7.32-7.27 (m, 5H), 7.25-7.22 (m, 2H), 7.18-7.12 (m, 5H), 7.10-7.05 (m, 2H), 6.70 (s, 1H), 5.35 (s, 1H), 5.07 (s, 1H); **^{13}C NMR** (100 MHz, Chloroform-*d*) ^{13}C NMR (101 MHz, Chloroform-*d*) δ 145.19, 144.61, 143.03, 139.94, 139.27, 133.17, 130.23, 128.34, 128.23, 128.19, 128.10, 128.05, 127.91, 127.88, 127.30, 118.15. **HRMS** calculated for $C_{22}H_{17}Cl^{35}$ $[M]^+$ 316.1013, found 316.1008; for $C_{22}H_{17}Cl^{37}$ $[M]^+$ 318.0984, found 318.0984.

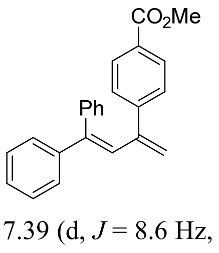

Methyl 4-(4,4-diphenylbuta-1,3-dien-2-yl)benzoate (6v): Prepared according to the general procedure B (**1** (0.20 mmol), **4** (0.25 mmol), KO^{t}Bu was used instead of NaF), an inseparable mixture of **6v+1v (6v/1v = 4:1)**, colorless oil, 39.9 mg, 48% yield (the yield of the **6v** has been adjusted according to ^{1}H NMR analysis), R_f = 0.5 (PE/EA = 20/1), **^{1}H NMR** (400 MHz, Chloroform-*d*) δ 7.86 (d, *J* = 8.6 Hz, 2H), 7.39 (d, *J* = 8.6 Hz, 2H), 7.36-7.29 (m, 5H), 7.17-7.12 (m, 3H), 7.12-7.07 (m, 2H), 6.77 (d, *J* = 1.0 Hz, 1H), 5.47 (m, d, *J* = 0.9 Hz, 1H), 5.20 (t, *J* = 1.2 Hz, 1H), 3.90 (s, 3H); **^{13}C NMR** (100 MHz, Chloroform-

d) δ 167.07, 145.46, 145.41, 145.04, 142.96, 139.88, 130.24, 129.46, 128.89, 128.34, 128.11, 128.05, 127.92, 127.62, 127.37, 126.84, 119.57, 115.90, 52.17. **HRMS** calculated for $C_{24}H_{21}O_2$ $[M+H]^+$ 341.1536, found 341.1537.

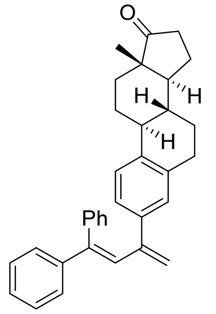

(8R,9S,13S,14S)-3-(4,4-Diphenylbuta-1,3-dien-2-yl)-13-methyl-6,7,8,9,11,12,13,14,15,16-decahydro-17H-cyclopenta[a]phenanthren-17-one (6w): Prepared according to the general procedure B (**1** (0.25 mmol)), an inseparable mixture of **6w+6w'** (**6w/6w'** = 13:1), 36.1 mg, 39% yield, R_f = 0.3 (PE/EA = 5/1), **^{1}H NMR** (400 MHz, Chloroform-*d*) δ 7.39-7.29 (m, 5H), 7.26-7.16 (m, 7H), 7.13 (s, 1H), 6.72 (s, 1H), 5.38 (d, *J* = 1.1 Hz, 1H), 4.97 (s, 1H), 2.93-2.82 (m, 2H), 2.57-2.47 (m, 1H), 2.47-2.40 (m, 1H), 2.35-2.25 (m, 1H), 2.21-1.95 (m, 5H), 1.71-1.54 (m, 4H), 1.51-1.36 (m, 2H), 0.93 (s, 3H); **^{13}C NMR** (100 MHz, Chloroform-*d*) δ 144.90, 144.58, 143.25, 140.27, 139.21, 138.27, 136.23, 130.18, 128.57, 128.28, 128.06, 128.00, 127.68, 127.42, 127.01, 125.36, 124.20, 116.86, 50.58, 48.10, 44.51, 38.26, 35.99, 31.69, 29.54, 26.63, 25.84, 21.71, 13.96. **HRMS** calculated for $C_{34}H_{35}O$ $[M+H]^+$ 459.2682, found 459.2697.

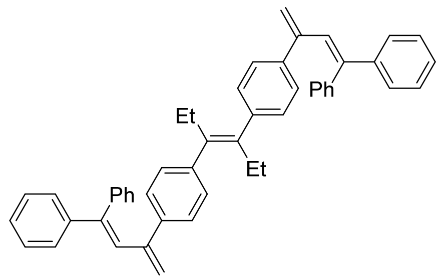

(*E*)-((Hex-3-ene-3,4-diylbis(4,1-phenylene))bis(buta-1,3-diene-3,1,1-triyl))tetrabenzene (6x): Prepared according to the general procedure B (**1** (0.10 mmol), **4** (0.25 mmol), , Ni(cod)$_2$ (20 mol%), IPr • Cl (24 mol%), Cs_2CO_3 (3.0 equiv.), **B1** (40 mol%), KO^{t}Bu (40 mol%)), colorless oil, 51.5 mg, 79% yield, R_f = 0.6 (PE/EA = 50/1), **^{1}H NMR** (400 MHz, Chloroform-*d*) δ 7.35-7.27 (m, 14H), 7.18-7.10 (m, 10H), 7.02-6.95 (m, 4H), 6.82 (s, 2H), 5.43 (s, 2H), 5.14 (s, 1H), 5.12 (s, 1H), 2.11-1.98 (m, 6H), 0.81-0.66 (m, 9H); **^{13}C NMR** (100 MHz, Chloroform-*d*) δ 148.17, 145.69, 144.75, 144.67, 143.40, 141.81, 141.02, 140.40, 140.24, 139.07, 138.68, 137.53, 130.65, 130.44, 128.73, 128.62, 128.45, 128.29, 128.25, 128.17, 127.88, 127.75, 127.71, 127.17, 126.80, 126.64, 121.06, 117.77, 117.50, 28.52, 13.53, 13.41, 13.35. **HRMS** calculated for $C_{50}H_{45}$ $[M+H]^+$ 645.3516, found 645.3500.

3.4 Unsuccessful substrates

Supplementary Table 14. Unsuccessful substrates

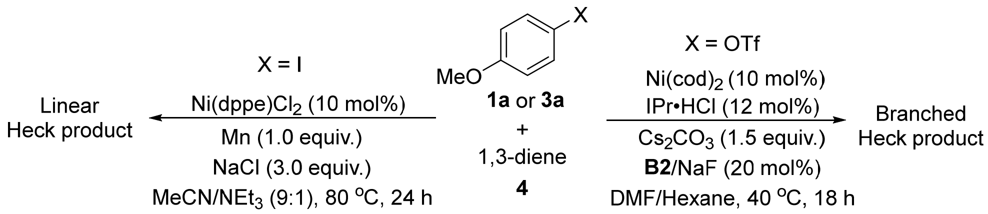

Linear Heck product	1,3-diene	Branched Heck product
N.D.	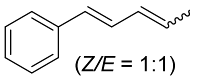 (*Z/E* = 1:1)	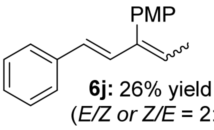 **6j:** 26% yield (*E/Z or Z/E* = 2:1)
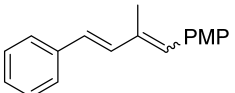 18% yield (**5q**:other isomers = 1:1)	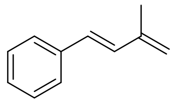	N.D.
--	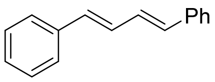	N.D.
N.D.	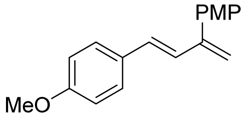	--
N.D.	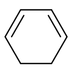	N.D.
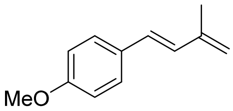 42% yield (**5p**:other isomers = 3:1)	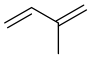	N.D.

3.5 Scale-up reaction

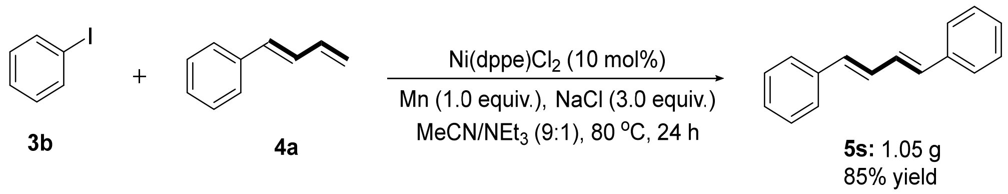

In a glove box, a sealed tube was charged with iodobenzene **3b** (1.0 mL, 6.0 mmol), Ni(dppe)Cl_2 (0.60 mmol, 10 mol%), Mn (0.60 mmol, 1.0 equiv.), NaCl (18 mmol, 3.0 equiv.), 1,3-diene **4a** (316.8 mg, 9.0 mmol), MeCN (15 mL), NEt_3 (1.7 mL). The reaction tube was sealed with a Teflon screw cap, removed from the glove box. Then, the reaction mixture was stirred at 80 °C for 24 hours. Upon completion, the mixture was filtered through a short pad of celite, concentrated in vacuo, and purified by silica chromatography (PE) to afford the product **5s** with 1.05 g, in 85% yield.

3.6 Cascade Heck reactions

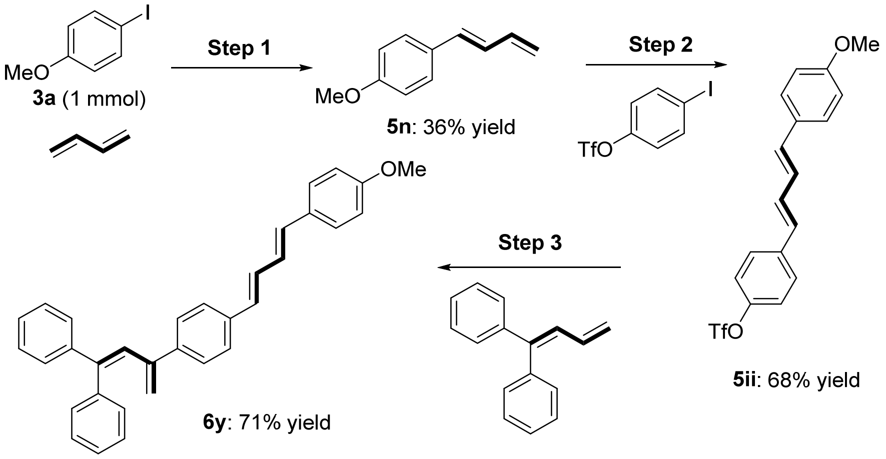

Step 1: In a glove box, a sealed tube was charged with 4-iodoanisole **3a** (1.0 mmol), Ni(dppe)Cl_2 (0.10 mmol, 10 mol%), Mn (1.0 mmol, 1.0 equiv.), NaCl (3.0 mmol, 3.0 equiv.), 1,3-butadiene **4** (3.0 mmol), MeCN (5.0 mL), NEt_3 (0.60 mL). The reaction tube was sealed with a Teflon screw cap, removed from the glove box. Then, the reaction mixture was stirred at 80 ºC for 24 hours. Upon completion, the mixture was filtered through a short pad of celite, concentrated in vacuo, and purified by silica chromatography (PE/EA = 50:1) to afford the product **5n.**

Step 2: In a glove box, a sealed tube was charged with 4-iodobenzene triflate (0.60 mmol), Ni(dppe)Cl_2 (0.04 mmol, 10 mol%), Mn (0.40 mmol, 1.0 equiv.), NaCl (1.2 mmol, 3.0 equiv.), diene **5n** (0.40 mmol), MeCN (1.0 mL), NEt_3 (112 uL). The reaction tube was sealed with a Teflon screw cap, removed from the glove box. Then, the reaction mixture was stirred at 80 ºC for 24 hours. Upon completion, the mixture was filtered through a short pad of celite, concentrated in vacuo, and purified by silica chromatography (PE/DCM = 4:1) to afford the product **5ii**.

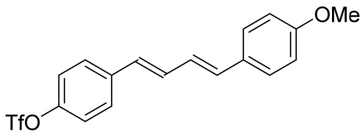

4-((1*E*,3*E*)-4-(4-Methoxyphenyl)buta-1,3-dien-1-yl)phenyl trifluoromethanesulfonate (5ii): Prepared according to the step 2, white solid, melting point: 123-125 ºC, 104.7 mg, 68% yield, R_f = 0.3 (PE/DCM = 4/1), **^{1}H NMR** (700 MHz, Chloroform-*d*) δ 7.46 (d, *J* = 8.7 Hz, 2H), 7.39 (d, *J* = 8.6 Hz, 2H), 7.22 (d, *J* = 8.7 Hz, 2H), 6.96-6.87 (m, 3H), 6.82 (dd, *J* = 15.4, 10.5 Hz, 1H), 6.68 (d, *J* = 15.4 Hz, 1H), 6.58 (d, *J* = 15.5 Hz, 1H), 3.83 (s, 3H); **^{13}C NMR** (125 MHz, Chloroform-*d*) δ 159.71, 148.43, 138.18, 134.12, 131.65, 129.93, 129.32, 127.96, 127.73, 126.64, 121.67, 118.90 (q, *J* = 321.7 Hz), 114.33, 55.43; **^{19}F NMR** (376 MHz, Chloroform-*d*) δ -72.78. **HRMS** calculated for $C_{18}H_{16}F_3O_4S$ $[M+H]^+$ 385.0716, found 385.0719.

Step 3: In a glove box, Ni(cod)$_2$ (0.02 mmol, 10 mol%), IPr·HCl (0.024 mmol, 12 mol%), Cs_2CO_3 (0.30 mmol, 1.5 equiv.) and **B1**/NaF (0.04 mmol, 20 mol%) were added to DMF (0.30 mL) in sequence and stirred at room temperature for 30 min. Then, the mixture of aryl triflate **5ii** (0.20 mmol), 1,1'-diaryl-1,3-diene **4** (0.25 mmol) in hexane (0.20 mL) was added into the reaction solvent. The reaction tube was sealed with a Teflon screw cap, removed from the glove box. And the reaction mixture was stirred at 40 ºC for 18 hours. Upon completion, the mixture was filtered through a short pad of celite, concentrated in vacuo, and purified by recrystallization to afford the corresponding product **6y.**

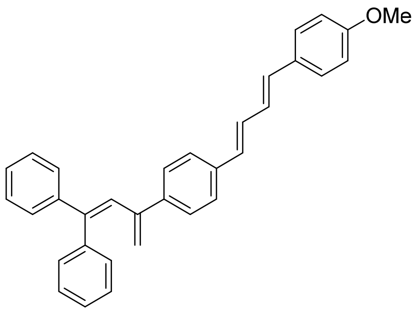

(3-(4-((1*E*,3*E*)-4-(4-Methoxyphenyl)buta-1,3-dien-1-yl)phenyl)buta-1,3-diene-1,1-diyl)dibenzene (6y): Prepared according to the step 3, yellow solid, melting point: 142-144 ºC, 62.2 mg, 71% yield, R_f = 0.4 (PE/EA = 20/1), **^{1}H NMR** (400 MHz, Chloroform-*d*) δ 7.47-7.28 (m, 11H), 7.25-7.11 (m, 5H), 6.96-6.80 (m, 4H), 6.74 (s, 1H), 6.67-6.52 (m, 2H), 5.42 (s, 1H), 5.02 (s, 1H), 3.83 (s, 3H); **^{13}C NMR** (100 MHz, Chloroform-*d*) δ 159.39, 144.90, 143.19, 140.17, 139.71, 136.90, 132.56, 131.45, 130.34, 130.23, 129.52, 128.36, 128.32, 128.10, 128.07, 127.75, 127.43, 127.20, 126.99, 126.13, 117.05, 114.27, 55.46. **HRMS** calculated for $C_{33}H_{29}O$ [M+H]$^+$ 441.2213, found 441.2210.

3.7 Construction of highly aryl-substituted pyridines

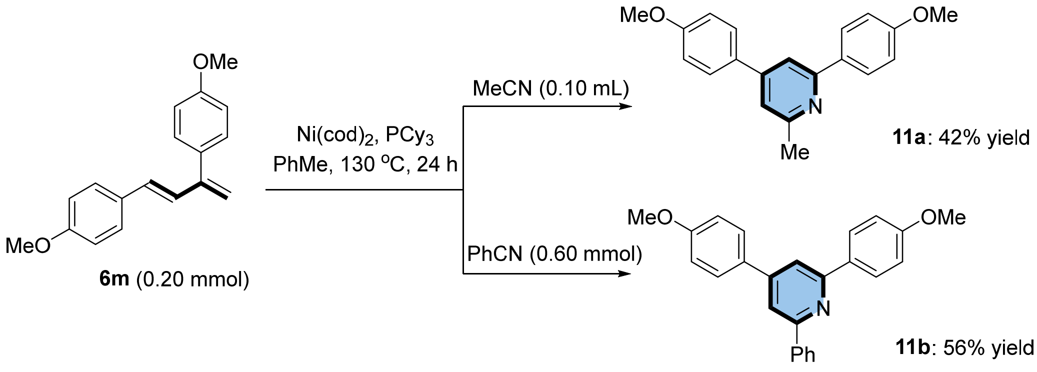

In a glove box, $Ni(cod)_2$ (0.02 mmol, 10 mol%), PCy_3 (0.08 mmol, 40 mol%), **6m** (0.20 mmol) and RCN (MeCN (0.1 mL) or PhCN (0.60 mmol)) were added to toluene (0.50 mL) in sequence. Then, the reaction tube was sealed with a Teflon screw cap, removed from the glove box. And the reaction mixture was stirred at 130 °C for 24 hours. Upon completion, the mixture was filtered through a short pad of celite, concentrated in vacuo, and purified by silica chromatography (PE/EA = 4:1) to afford the corresponding products **11a** and **11b**.[18]

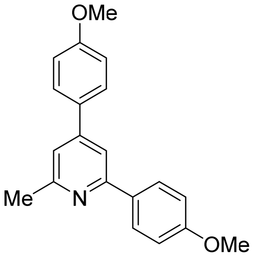

2,4-Bis(4-methoxyphenyl)-6-methylpyridine (11a): Prepared according to the procedure above, white solid, melting point: 98-100 °C, 25.9 mg, 42% yield, R_f = 0.3 (PE/EA = 4/1), **^{1}H NMR** (400 MHz, Chloroform-*d*) δ 7.98 (d, J = 8.8 Hz, 2H), 7.67-7.58 (m, 3H), 7.23 (s, 1H), 7.04-6.96 (m, 4H), 3.89-3.85 (m, 6H), 2.66 (s, 3H); **^{13}C NMR** (100 MHz, Chloroform-*d*) δ 160.40, 158.71, 157.35, 148.98, 132.77, 131.37, 128.49, 128.35, 118.78, 115.01, 114.54, 114.18, 55.54, 55.51, 25.00. **HRMS** calculated for $C_{20}H_{20}NO_2$ $[M+H]^+$ 306.1489, found 306.1487.

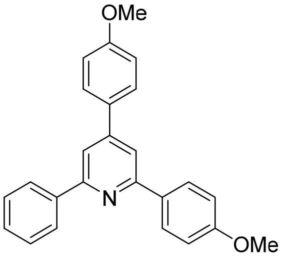

2,4-Bis(4-methoxyphenyl)-6-phenylpyridine (11b): Prepared according to the procedure above, white solid, melting point: 106-108 °C, 41.2 mg, 56% yield, R_f = 0.4 (PE/EA = 4/1), **^{1}H NMR** (400 MHz, Chloroform-*d*) δ 8.24-8.13 (m, 4H), 7.84-7.78 (m, 2H), 7.71 (d, J = 8.7 Hz, 2H), 7.52 (t, J = 7.4 Hz, 2H), 7.44 (t, J = 7.3 Hz, 1H), 7.11-6.99 (m, 4H), 3.89 (s, 6H); **^{13}C NMR** (100 MHz, Chloroform-*d*) δ 160.60, 160.52, 157.41, 157.19, 149.65, 139.95, 132.47, 131.56, 129.03, 128.79, 128.51, 128.44, 127.23, 116.13, 115.97, 114.62, 114.16, 55.57, 55.52. **HRMS** calculated for $C_{25}H_{22}NO_2$ $[M+H]^+$ 368.1645, found 368.1651.

3.8 Construction of highly aryl-substituted benzenes

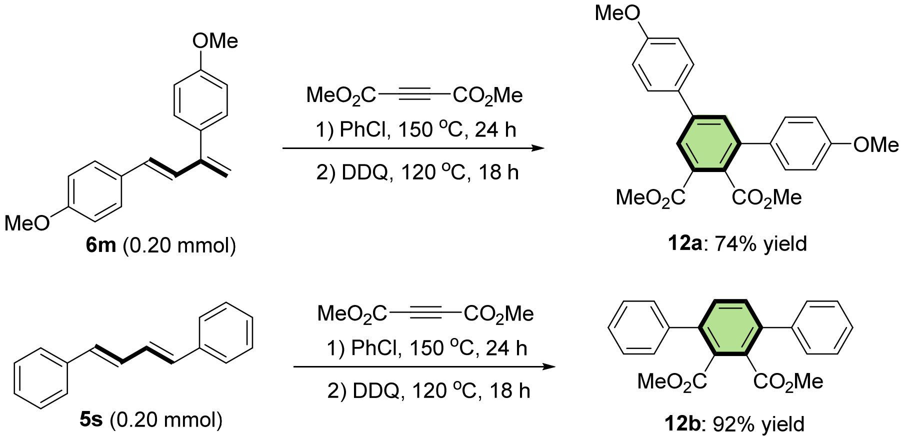

To the solution of 1,3-dienes **6m** or **5s** (0.20 mmol) in PhCl (1.0 mL) was added dimethyl acetylenedicarboxylate (0.60 mmol, 3.0 equiv.) and the reaction mixture was stirred in 150 °C under air atmosphere for 24 h. Then, DDQ was then added and the resulting solution was stirred for 18 h at 120 °C. Upon completion, the mixture was filtered through a short pad of celite, concentrated in vacuo, and purified by silica chromatography to afford the corresponding products **12a** and **12b**.[19]

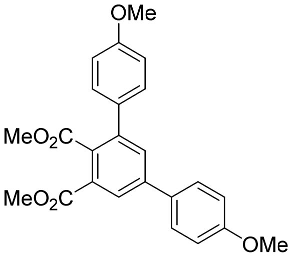

Dimethyl 4,4''-dimethoxy-[1,1':3',1''-terphenyl]-4',5'-dicarboxylate (12a): Prepared according to procedure above, colorless oil, 60.2 mg, 74% yield, R_f = 0.4 (PE/EA = 4/1), **^{1}H NMR** (400 MHz, Chloroform-*d*) δ 8.15 (d, *J* = 1.8 Hz, 1H), 7.70 (d, *J* = 1.8 Hz, 1H), 7.58 (d, *J* = 8.8 Hz, 2H), 7.35 (d, *J* = 8.8 Hz, 2H), 7.00 (d, *J* = 8.8 Hz, 2H), 6.95 (d, *J* = 8.8 Hz, 2H), 3.93 (s, 3H), 3.86 (s, 3H), 3.85 (s, 3H), 3.72 (s, 3H); **^{13}C NMR** (100 MHz, Chloroform-*d*) δ 169.69, 166.46, 159.99, 159.51, 141.84, 140.90, 132.86, 132.37, 131.86, 131.67, 129.91, 128.80, 128.44, 126.79, 114.55, 113.91, 55.53, 55.42, 52.79, 52.53. **HRMS** calculated for $C_{24}H_{22}O_6$ $[M+H]^+$ 407.1489, found 407.1492.

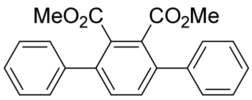

Dimethyl [1,1':4',1''-terphenyl]-2',3'-dicarboxylate (12b): Prepared according to procedure above, white solid, melting point: 188-190 °C, 63.9 mg, 92% yield, R_f = 0.4 (PE/DCM = 2/1), **^{1}H NMR** (400 MHz, Chloroform-*d*) δ 7.52 (s, 2H), 7.47-7.30 (m, 8H), 3.62 (s, 6H); **^{13}C NMR** (100 MHz, Chloroform-*d*) δ 168.91, 139.97, 139.79, 132.13, 131.79, 128.51, 128.32, 127.90, 52.51. **HRMS** calculated for $C_{22}H_{19}O_4$ $[M+H]^+$ 347.1278, found 347.1280.

3.9 Construction of diaryl thiophenes

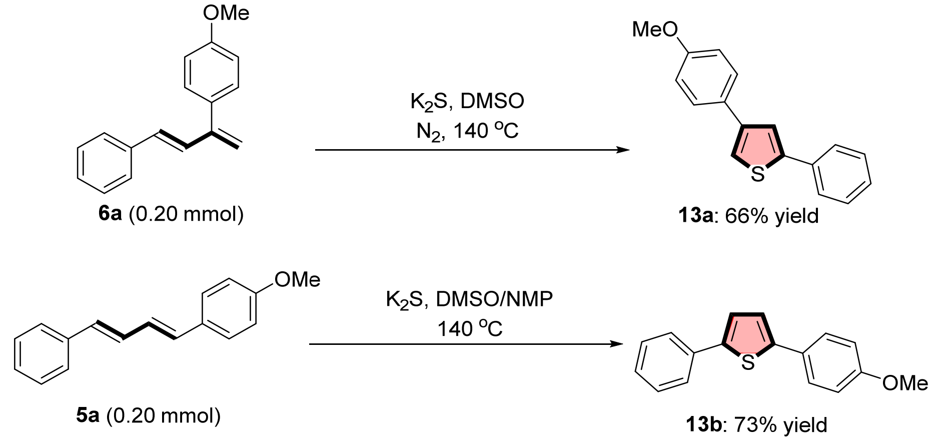

In N_2/air atmosphere, a sealed tube was charged with K_2S (0.60 mmol), **6a** or **5a** (0.20 mmol), DMSO or DMSO/NMP (1:1 *v/v*) (1.0 mL) at room temperature. Then, the reaction mixture was stirred at 140 ºC for 24 hours. The crude reaction mixture was purified by column chromatography on silica gel using petroleum ether and dichloromethane to afford the diaryl thiophenes **13a** and **13b**.[20]

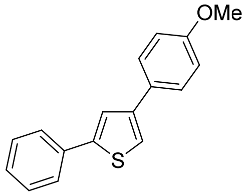

4-(4-Methoxyphenyl)-2-phenylthiophene (13a): Prepared according to procedure above, yellow solid, 151-153 ºC, 35.3 mg, 66% yield, R_f = 0.3 (PE/DCM = 4/1), **^{1}H NMR** (400 MHz, Chloroform-*d*) δ 7.67 (d, *J* = 7.6 Hz, 1H), 7.61-7.54 (m, 2H), 7.42 (t, *J* = 7.6 Hz, 1H), 7.37-7.27 (m, 1H), 6.97 (d, *J* = 8.7 Hz, 1H), 3.86 (s, 1H); **^{13}C NMR** (100 MHz, Chloroform-*d*) δ 159.07, 145.00, 142.89, 134.51, 129.04, 128.83, 127.75, 127.56, 125.95, 122.37, 118.53, 114.31, 55.47. **HRMS** calculated for $C_{17}H_{15}OS$ $[M+H]^+$ 267.0838, found 267.0840.

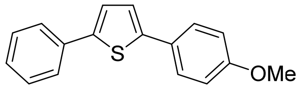

2-(4-Methoxyphenyl)-5-phenylthiophene (13b): Prepared according to procedure above, white solid, 166-168 ºC, 39.0 mg, 73% yield, R_f = 0.6 (PE/DCM = 2/1), **^{1}H NMR** (400 MHz, Chloroform-*d*) δ 7.63 (d, *J* = 7.2 Hz, 2H), 7.56 (d, *J* = 8.8 Hz, 2H), 7.39 (t, *J* = 7.7 Hz, 2H), 7.30-7.26 (m, 2H), 7.18 (s, 1H), 6.93 (d, *J* = 8.8 Hz, 2H), 3.85 (s, 3H); **^{13}C NMR** (100 MHz, Chloroform-*d*) δ 159.35, 143.72, 142.70, 134.54, 129.02, 127.45, 127.32, 127.06, 125.65, 124.07, 123.09, 114.44, 55.52. **HRMS** calculated for $C_{17}H_{15}OS$ $[M+H]^+$ 267.0838, found 267.0841.

3.10 Construction of highly aryl-substituted naphthalene and thiophene

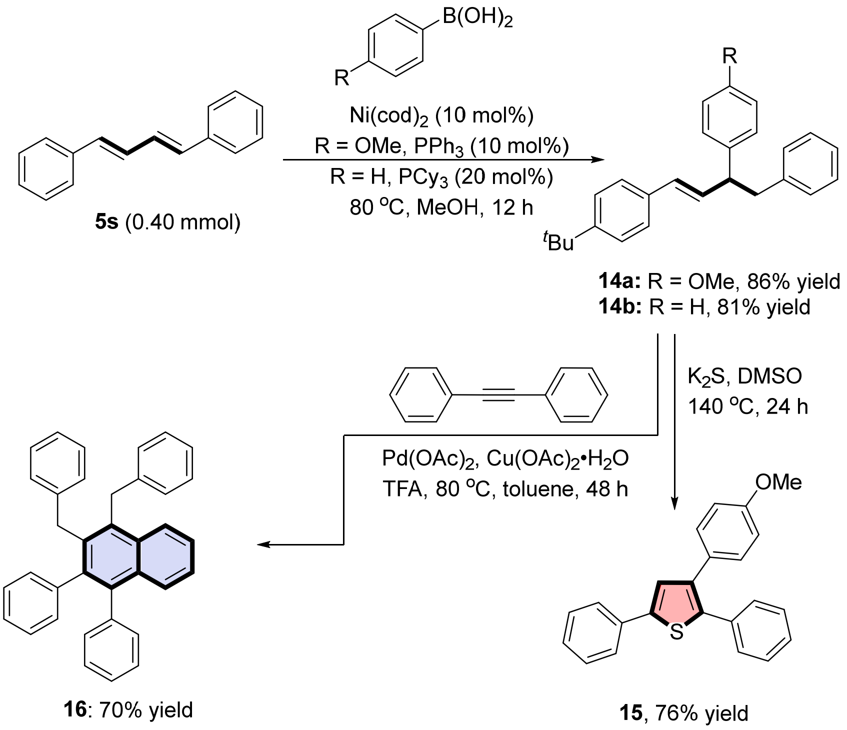

In a glove box, a sealed tube was charged with iodobenzene **5s** (0.40 mmol), Ni(cod)$_2$ (0.02 mmol, 5 mol%), liagnd (0.04 mmol, 10 mmol%), arylboronic acid (0.60 mmol), MeOH (1.0 mL) at room temperature. The reaction tube was sealed with a Teflon screw cap, removed from the glove box. Then, the reaction mixture was stirred at 80 ºC for 12 hours. The crude reaction mixture was purified by column chromatography on silica gel using petroleum ether and ethyl acetate to afford the corresponding products **14a** and **14b**.[21]

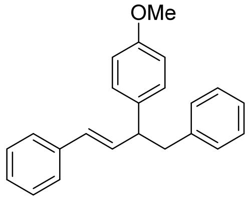

(*E*)-(3-(4-Methoxyphenyl)but-1-ene-1,4-diyl)dibenzene (14a): Prepared according to the procedure above, colorless oil, 108.1 mg, 86% yield, R_f = 0.5 (PE/EA = 50/1), **^{1}H NMR** (400 MHz, Chloroform-*d*) δ 7.32-7.05 (m, 12H), 6.82 (d, *J* = 8.7 Hz, 2H), 6.39 (dd, *J* = 15.9, 7.2 Hz, 1H), 6.26 (d, *J* = 16.0 Hz, 1H), 3.76 (s, 3H), 3.72-3.63 (m, 1H), 3.22-2.96 (m, 2H); **^{13}C NMR** (100 MHz, Chloroform-*d*) δ 158.16, 140.15, 137.62, 135.84, 133.75, 129.74, 129.39, 128.90, 128.55, 128.20, 127.15, 126.25, 126.03, 113.91, 55.33, 50.09, 42.85. **HRMS** calculated for $C_{23}H_{23}O$ $[M+H]^+$ 315.1743, found 315.1750.

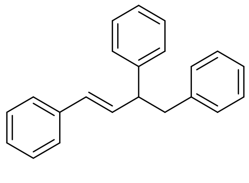

(*E*)-But-3-ene-1,2,4-triyltribenzene (14b): Prepared according to the procedure above, colorless oil, 92.4 mg, 81% yield, R_f = 0.4 (PE), **^{1}H NMR** (400 MHz, Chloroform-*d*) δ 7.31-7.11 (m, 13H), 7.11-7.03 (m, 2H), 6.41 (dd, *J* = 15.9, 7.4 Hz, 1H), 6.28 (d, *J* = 16.0 Hz, 1H), 3.72 (q, *J* = 7.4 Hz, 1H), 3.20-3.03 (m, 2H); **^{13}C NMR** (100 MHz, Chloroform-*d*) δ 143.85, 140.04, 137.57, 133.38, 130.08, 129.38, 128.56, 128.22, 127.98, 127.21, 126.50, 126.28, 126.08, 51.03, 42.78. **HRMS** calculated for $C_{22}H_{20}$ $[M]^+$ 284.1560, found 284.1561.

In air atmosphere, a sealed tube was charged with K_2S (0.60 mmol), **14a** (0.20 mmol), DMSO (1.0 mL) at room temperature. Then, the reaction mixture was stirred at 140 ºC for 24 hours. The crude reaction mixture was purified by column chromatography on silica gel using petroleum ether and ethyl acetate to afford the polyaryl thiophene **15** with 52.2 mg, in 76% yield.[20]

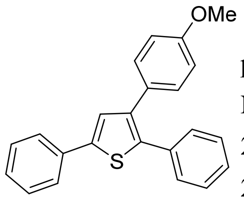

3-(4-Methoxyphenyl)-2,5-diphenylthiophene (15): Prepared according to procedure above, colorless oil, 52.2 mg, 76% yield, R_f = 0.5 (PE/EA = 20/1), **^{1}H NMR** (400 MHz, Chloroform-*d*) δ 7.64 (d, *J* = 7.2 Hz, 2H), 7.39 (t, *J* = 7.6 Hz, 2H), 7.36-7.32 (m, 3H), 7.32-7.26 (m, 3H), 7.26-7.20 (m, 3H), 6.84 (d, *J* = 8.8 Hz, 2H), 3.81 (s, 3H); **^{13}C NMR** (100 MHz, Chloroform-*d*) δ 158.77, 142.49, 138.73, 137.30, 134.51, 134.27, 130.28, 129.23, 129.14, 129.06, 128.60, 127.70, 127.46, 126.64, 125.71, 113.96, 55.36. **HRMS** calculated for $C_{23}H_{19}SO$ $[M+H]^+$ 343.1151, found 343.1152.

In a glove box, a sealed tube was charged with 1,2-bisphenyl ethyne (0.30 mmol), **14b** (0.20 mmol), $Pd(OAc)_2$ (0.02 mmol, 10 mol%), $Cu(OAc)_2\cdot H_2O$ (0.30 mmol), TFA (2.0 mmol), toluene (0.5 mL) at room temperature. The reaction tube was sealed with a Teflon screw cap, removed from the glove box. Then, the reaction mixture was stirred at 80 ºC for 18 hours.[22] The crude reaction mixture was purified by column chromatography on silica gel using petroleum ether and ethyl acetate to afford the polyaryl naphthalene **16**.[22]

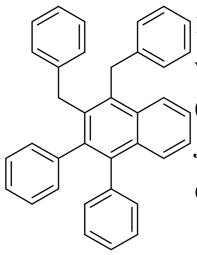

1,2-Dibenzyl-3,4-diphenylnaphthalene (16): Prepared according to procedure above, white solid, melting point: 123-125 ºC, 64.4 mg, 70% yield, R_f = 0.5 (PE), **^{1}H NMR** (400 MHz, Chloroform-*d*) δ 7.99 (d, *J* = 8.5 Hz, 1H), 7.49 (d, *J* = 8.4 Hz, 1H), 7.38 (t, *J* = 7.3 Hz, 1H), 7.29 (d, *J* = 7.7 Hz, 1H), 7.24-6.98 (m, 15H), 7.00-6.88 (m, 3H), 6.86-6.75 (m, 4H), 4.46 (s, 2H), 3.97 (s, 2H); **^{13}C NMR** (100 MHz, Chloroform-*d*) δ 140.93, 140.54, 139.92, 138.77, 135.37, 134.23, 132.57, 132.29, 131.27, 130.44, 128.61, 128.24, 128.21, 128.19, 127.75, 127.49, 127.15, 126.37, 126.16, 126.06, 125.67, 125.43, 124.80, 37.13, 35.12. **HRMS** calculated for $C_{36}H_{28}$ [M] 460.2191, found 460.2195.

3.11 Programmable constructions of poly (hetero)aromatic compounds

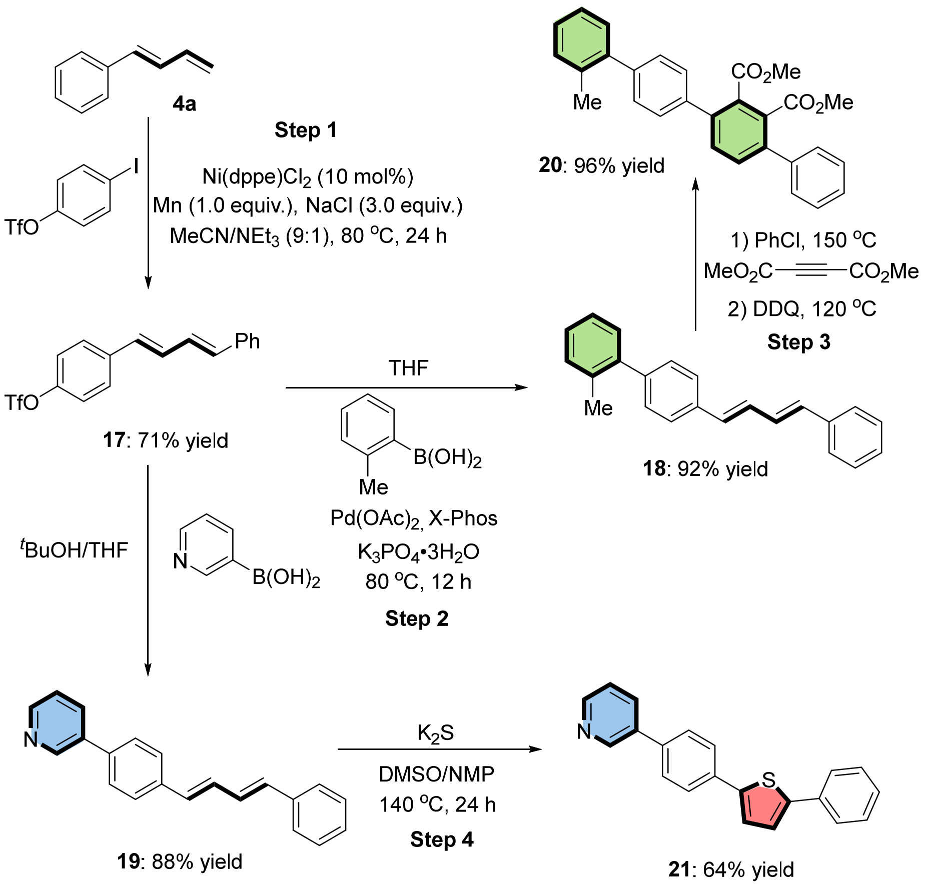

Step 1: In a glove box, a sealed tube was charged with 4-iodobenzene triflate (0.60 mmol), Ni(dppe)Cl_2 (0.04 mmol, 10 mol%), Mn (0.60 mmol, 1.0 equiv.), NaCl (1.2 mmol, 3.0 equiv.), diene **4a** (0.40 mmol), MeCN (1.0 mL) NEt_3 (112 uL). The reaction tube was sealed with a Teflon screw cap, removed from the glove box. Then, the reaction mixture was stirred at 80 ºC for 24 hours. Upon completion, the mixture was filtered through a short pad of celite, concentrated in vacuo, and purified by silica chromatography to afford the product **17**.

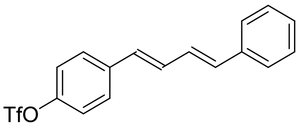

4-((1*E*,3*E*)-4-Phenylbuta-1,3-dien-1-yl)phenyl trifluoromethanesulfonate (17): Prepared according to the step 1, white solid, melting point: 90-91 ºC, 101.2 mg, 71% yield, R_f = 0.5 (PE), **^{1}H NMR** (400 MHz, Chloroform-*d*) δ 7.42 (d, *J* = 8.6 Hz, 4H), 7.32 (t, *J* = 7.5 Hz, 2H), 7.24 (t, *J* = 7.3 Hz, 1H), 7.19 (d, *J* = 8.8 Hz, 2H), 6.97-6.83 (m, 2H), 6.74-6.64 (m, 1H), 6.64-6.53 (m, 1H); **^{13}C NMR** (100 MHz, Chloroform-*d*) δ 148.56, 137.90, 137.06, 134.43, 131.30, 130.46, 128.84, 128.62, 128.07, 127.87, 126.65, 121.67, 118.91 (q, *J* = 324.9 Hz); **^{19}F NMR** (376 MHz, Chloroform-*d*) δ -72.78. **HRMS** calculated for $C_{17}H_{14}F_3O_3S$ $[M+H]^+$ 355.0610, found 355.0612.

Step 2: In glove box, a sealed tube was charged with **15** (0.20 mmol), $Pd(OAc)_2$ (0.01 mmol, 5 mol%), X-Phos (0.02 mmol, 10 mol%), $K_3PO_4{\cdot}H_2O$ (0.6 mmol, 3.0 equiv.), arylboronic acid (0.40 mmol), THF or ^{t}BuOH/THF (5:1 *v/v*) (0.5 mL). The reaction tube was sealed with a Teflon screw cap, removed from the glove box. Then, the reaction mixture was stirred at 80 ºC for 12 hours. Upon completion, the

mixture was filtered through a short pad of celite, concentrated in vacuo, and purified by silica chromatography to afford the products **18** and **19**.[23]

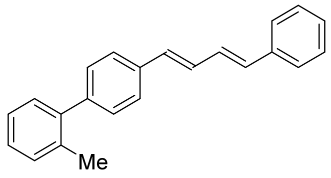

2-Methyl-4'-((1*E*,3*E*)-4-phenylbuta-1,3-dien-1-yl)-1,1'-biphenyl (18): Prepared according to the step 2, white solid, melting point: 115-117 °C, 54.6 mg, 92% yield, R_f = 0.7 (PE), **^{1}H NMR** (400 MHz, Chloroform-*d*) δ 7.48 (d, *J* = 8.2 Hz, 2H), 7.44 (d, *J* = 7.4 Hz, 2H), 7.37-7.28 (m, 4H), 7.25-7.19 (m, 4H), 7.04-6.93 (m, 2H), 6.77-6.59 (m, 2H), 2.29 (s, 3H); **^{13}C NMR** (100 MHz, Chloroform-*d*) δ 141.61, 141.35, 137.49, 136.02, 135.47, 132.93, 132.60, 130.54, 129.84, 129.69, 129.43, 129.39, 128.80, 127.70, 127.43, 126.52, 126.23, 125.96, 20.68. **HRMS** calculated for $C_{23}H_{20}$ $[M]^+$ 296.1560, found 296.1556.

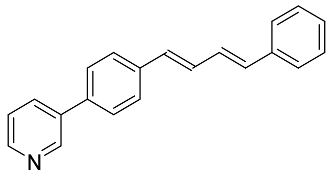

3-(4-((1*E*,3*E*)-4-Phenylbuta-1,3-dien-1-yl)phenyl)pyridine (19): Prepared according to the step 2, yellow solid, melting point: 162-163 °C, 49.7 mg, 88% yield, R_f = 0.2 (PE/EA = 4:1), **^{1}H NMR** (400 MHz, Chloroform-*d*) δ 8.88 (s, 1H), 8.59 (s, 1H), 7.89 (d, *J* = 7.3 Hz, 1H), 7.67-7.51 (m, 4H), 7.46 (d, *J* = 7.3 Hz, 2H), 7.39-7.30 (m, 3H), 7.25-7.19 (m, 1H), 7.08-6.92 (m, 2H), 6.72 (d, *J* = 14.2 Hz, 2H); **^{13}C NMR** (175 MHz, Chloroform-*d*) δ 148.55, 148.21, 137.45, 137.37, 136.84, 136.30, 134.20, 133.53, 132.05, 130.10, 129.22, 128.84, 127.86, 127.47, 127.20, 126.59, 123.75. **HRMS** calculated for $C_{21}H_{18}N$ $[M+H]^+$ 284.1434, found 284.1433.

Step 3: To the solution of 1,3-dienes **18** (0.10 mmol) in PhCl (0.50 mL) was added dimethyl acetylenedicarboxylate (0.30 mmol, 3.0 equiv.) and the reaction mixture was stirred in 150 °C under air atmosphere for 24 h. Then, DDQ was then added and the resulting solution was stirred for 18 h at 120 °C. Upon completion, the mixture was filtered through a short pad of celite, concentrated in vacuo, and purified by silica chromatography to afford the corresponding product **20**.[22]

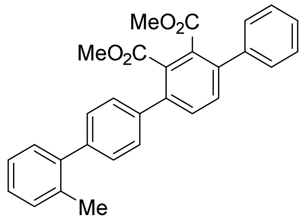

Dimethyl 2'''-methyl-[1,1':4',1'':4'',1'''-quaterphenyl]-2',3'-dicarboxylate (20): Prepared according to the step 3, white solid, melting point: 116-118 °C, 41.7 mg, 96% yield, R_f = 0.3 (PE/EA = 3:1), **^{1}H NMR** (400 MHz, Chloroform-*d*) δ 7.59 (d, *J* = 8.0 Hz, 1H), 7.54 (d, *J* = 8.0 Hz, 1H), 7.50-7.35 (m, 9H), 7.34-7.27 (m, 4H), 3.65 (s, 3H), 3.63 (s, 3H), 2.33 (s, 3H); **^{13}C NMR** (100 MHz, Chloroform-*d*) δ 169.04, 168.96, 141.55, 141.38, 140.03, 139.84, 139.76, 138.30, 135.44, 132.25, 132.22, 131.86, 131.80, 130.54, 129.88, 129.38, 128.55, 128.38, 128.09, 127.94, 127.57, 125.98, 52.56, 52.52, 20.63. **HRMS** calculated for $C_{29}H_{25}O_4$ $[M+H]^+$ 437.1747, found 437.1729.

Step 4: In air atmosphere, a sealed tube was charged with K_2S (0.30 mmol), **19** (0.10 mmol), DMSO (0.5 mL) at room temperature. Then, the reaction mixture was stirred at 140 °C for 24 hours. The crude reaction mixture purified by column chromatography on silica gel using petroleum ether and ethyl acetate to afford the corresponding product **21**.[20]

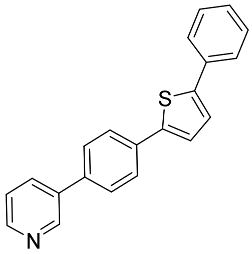

3-(4-(5-Phenylthiophen-2-yl)phenyl)pyridine (21): Prepared according to the step 3, white solid, melting point: 203-205 °C, 20.0 mg, 64% yield, R_f = 0.2 (PE/EA = 4:1), **^{1}H NMR** (400 MHz, Chloroform-*d*) δ 8.89 (d, *J* = 1.8 Hz, 1H), 8.66-8.55 (m, 1H), 7.91 (dt, *J* = 7.9, 1.9 Hz, 1H), 7.75 (d, *J* = 8.3 Hz, 2H), 7.69-7.57 (m, 4H), 7.45-7.35 (m, 4H), 7.34-7.27 (m, 2H); **^{13}C NMR** (100 MHz, Chloroform-*d*) δ 148.70, 148.23, 144.24, 142.85, 136.87, 136.10, 134.30, 134.20, 129.10, 127.80, 127.71, 126.33, 125.80, 124.53, 124.28, 123.76. **HRMS** calculated for $C_{21}H_{16}NS$ $[M+H]^+$ 314.0998, found 314.0998.

3.12 Further Heck reaction[a]

Supplementary Table 15. Further Heck reaction

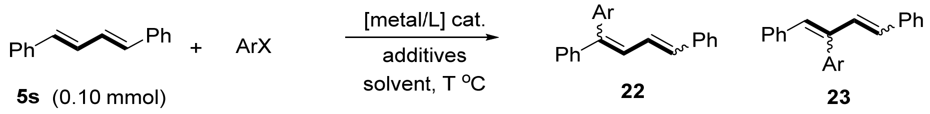

Entry	Ref.	ArX	Metal/ Ligand	Additives/ Solvent	T/° C	Time/ h	**22** (%)	**23** (%)
1[b]	Studer[24]	$PhB(OH)_2$	$Pd(OAc)_2$	TEMPO/KF $C_2H_5CO_2H$	r.t.	18	--	29
2[c]	Endo[25]	PMPI	$Pd(OAc)_2$ /PPh_3	Ag_2CO_3/ DMF	80	24	Mixture (45)	
3[d]	Cabri[26]	PhOTf	$Pd(OAc)_2$ /dppp	NEt_3/ DMF	80	18	N.D.	
4[e]	Zhou[27]	PhOTf	Pd_2dba_3/ dppf	Urotropine/ DMA	80	18	N.D.	
5[f]	Jamison[28]	PhOTf	$Ni(cod)_2$/ dppb	DABCO/ PhMe	60	24	N.D.	
6[g]	Watson[29]	PMPOPiv	$Ni(cod)_2$/ dppf	K_3PO_4/ PhMe	125	24	N.D.	

[a]Conditions: **5s** (0.10 mmol). [b]$Pd(OAc)_2$ (10 mol%), TEMPO (4.0 eq.), KF (4.0 eq.), $PhB(OH)_2$ (4.0 eq.), $C_2H_5CO_2H$ (0.50 mL); [c]$Pd(OAc)_2$ (10 mol%), PPh_3 (20 mol%), PhOTf (2.0 eq.), Ag_2CO_3 (2.0 equiv.), DMF (0.50 mL); [d]$Pd(OAc)_2$ (5.0 mol%), dppp (6.0 mol%), PhOTf (2.0 eq.), NEt_3 (2.0 eq.), DMF (0.50 mL); [e]Pd_2dba_3 (5.0 mol%), dppf (10 mol%), PhOTf (2.0 eq.), Urotropine (2.0 eq.), DMA (0.50 mL); [f]$Ni(cod)_2$ (15 mol%), dppb (18 mol%), PhOTf (2.0 eq.), DABCO (3.0 eq.), PhMe (0.50 mL); [f]$Ni(cod)_2$ (10 mol%), dppf (12 mol%), PMPOPiv (2.0 eq.), K_3PO_4 (2.0 eq.), PhMe (0.50 mL).

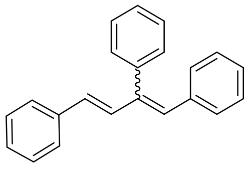

((3*E*)-Buta-1,3-diene-1,2,4-triyl)tribenzene (23): Prepared according to the procedure above, known compound,[30] colorless oil, 8.2 mg, 29% yield, R_f = 0.3 (PE), **^{1}H NMR** (400 MHz, Chloroform-*d*) δ 7.49-7.34 (m, 12H), 7.33-7.28 (m, 3H), 7.23 (t, *J* = 7.2 Hz, 1H), 6.65 (s, 1H), 6.56 (d, *J* = 16.2 Hz, 1H); **^{13}C NMR** (175 MHz, Chloroform-*d*) δ 142.35, 141.30, 137.74, 137.63, 134.29, 131.45, 129.85, 129.47, 128.79, 128.45, 128.37, 127.81, 127.68, 127.26, 127.23, 126.76.

4. Supplementary Note 3

4.1 Control experiments

Supplementary Table 16. Control experiments

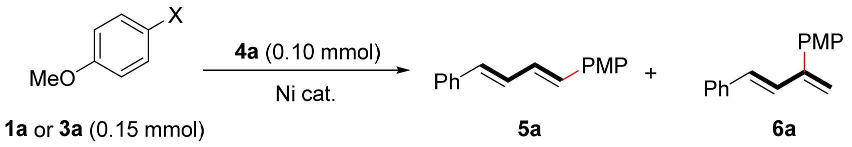

Entry	X	Conditions	Yield of **5a**	Yield of **6a**
1	I	Condition A	81%	0%
2	I	Condition B	0%	0%
3	OTf	Condition A	22%	0%
4	OTf	Condition B	0%	74%

Condition A: In a glove box, a sealed tube was charged with Ar-X (0.15 mmol), Ni(dppe)Cl_2 (0.01 mmol, 10 mol%), Mn (0.10 mmol), NaCl (0.30 mmol), 1,3-diene **4a** (0.10 mmol), MeCN (0.5 mL), NEt_3 (56 uL) at room temperature. The reaction tube was sealed with a Teflon screw cap, removed from the glove box. Then, the reaction mixture was stirred at 80 ºC for 24 hours.

Condition B: In a glove box, Ni(cod)$_2$ (0.01 mmol, 10 mol%), IPr·HCl (0.012 mmol, 12 mol%), Cs_2CO_3 (0.15 mmol, 1.5 equiv.) and [B1]/NaF (0.02 mmol, 20 mol%) were added to DMF (0.30 mL) in sequence and stirred at room temperature for 30 min. Then, the mixture of Ar-X (0.15 mmol), 1,3-diene **4a** (0.10 mmol) in Hexane (0.20 mL) was added into the reaction solvent. The reaction tube was sealed with a Teflon screw cap, removed from the glove box. And the reaction mixture was stirred at 40 ºC for 18 hours.

The yields of **5a** and **6a** were determined by GC-FID analysis of the crude products mixture using mesitylene as internal standard.

4.2 Stereoconvergent Heck reactions of dienes

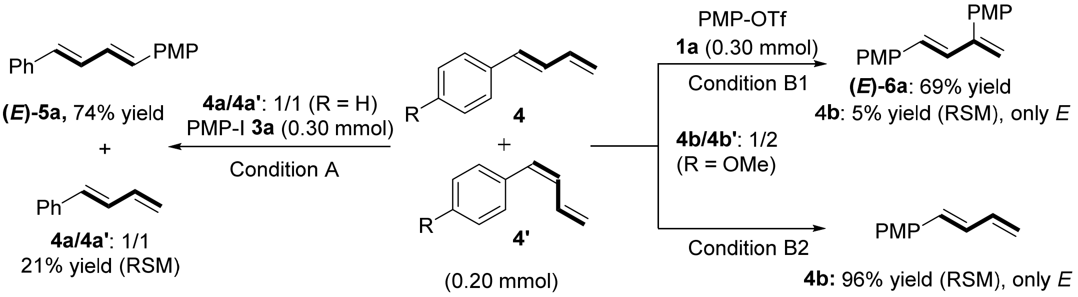

Condition A: In a glove box, a sealed tube was charged with PMP-I **3a** (0.30 mmol), Ni(dppe)Cl_2 (0.02 mmol, 10 mol%), Mn (0.20 mmol), NaCl (0.60 mmol), dienes **4a+4a'** (0.20 mmol), MeCN (0.5 mL), NEt_3 (56 uL) at room temperature. The reaction tube was sealed with a Teflon screw cap, removed from the glove box. Then, the reaction mixture was stirred at 80 °C for 24 hours. And the crude reaction mixture was purified by column chromatography on silica gel using petroleum ether and ethyl acetate to afford the corresponding product ***E*-5a** with 34.8 mg, in 74% yield. Dienes (**4a** and **4a'**) was recovered in 21% yield with 1:1 *E/Z.*

Condition B1: In a glove box, Ni(cod)$_2$ (0.02 mmol, 10 mol%), IPr·HCl (0.024 mmol, 12 mol%), Cs_2CO_3 (0.30 mmol, 1.5 equiv.) and [B1]/NaF (0.04 mmol, 20 mol%) were added to DMF (0.30 mL) in sequence and stirred at room temperature for 30 min. Then, the mixture of PMP-OTf **1a** (0.30 mmol), dienes **4b+4b'** (0.20 mmol) in Hexane (0.20 mL) was added into the reaction solvent. The reaction tube was sealed with a Teflon screw cap, removed from the glove box. And the reaction mixture was stirred at 40 °C for 18 hours. The crude reaction mixture was purified by column chromatography on silica gel using petroleum ether and ethyl acetate to afford the corresponding product ***E*-6a** with 36.9 mg, in 69% yield. And only *E*-diene **4b** was recovered in 5% yield.

Condition B2: In a glove box, Ni(cod)$_2$ (0.02 mmol, 10 mol%), IPr·HCl (0.024 mmol, 12 mol%), Cs_2CO_3 (0.30 mmol, 1.5 equiv.) and [B1]/NaF (0.04 mmol, 20 mol%) were added to DMF (0.30 mL) in sequence and stirred at room temperature for 30 min. Then, the mixture of dienes **4b+4b'** (0.20 mmol) in Hexane (0.20 mL) was added into the reaction solvent. The reaction tube was sealed with a Teflon screw cap, removed from the glove box. And the reaction mixture was stirred at 40 °C for 18 hours. The crude reaction mixture was purified by column chromatography on silica gel using petroleum ether and ethyl acetate to recover the starting material. And only *E*-diene **4b** was obtained in 96% yield.

4.3 The effect of ligands

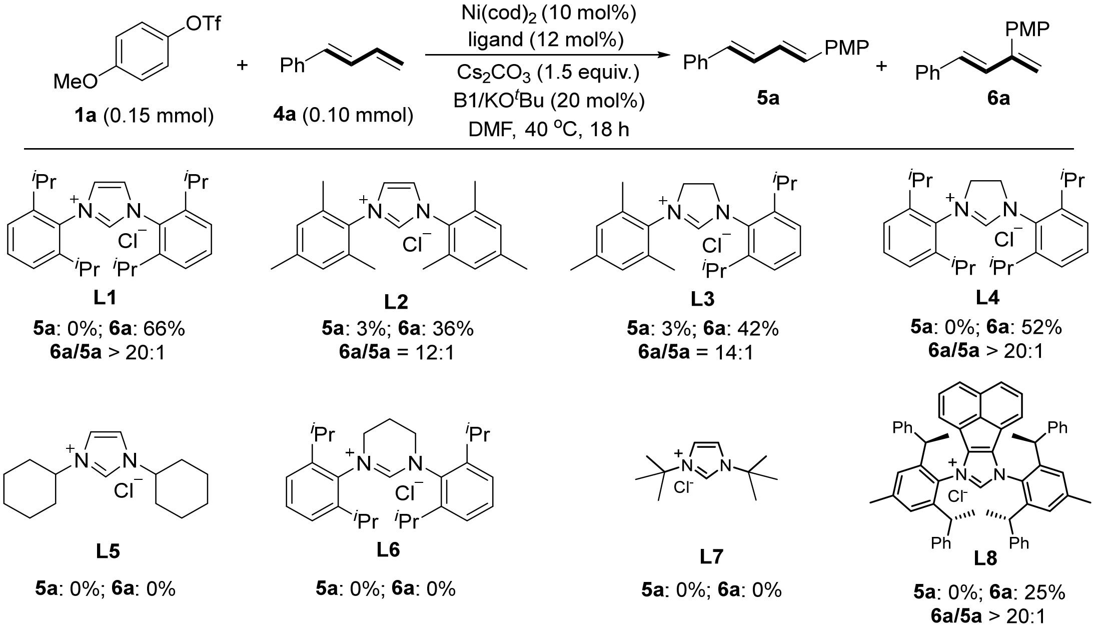

In glove box, $Ni(cod)_2$ (0.01 mmol, 10 mol%), NHC-ligand (0.012 mmol, 12 mol%), Cs_2CO_3 (0.15 mmol, 1.5 equiv.), [B1]/KO^{t}Bu (0.02 mmol, 20 mol%), **1a** (0.15 mmol) and **4a** (0.10 mmol) were added to DMF (0.30 mL) in sequence and stirred at 40 °C for 18 h. Then, yields and selectivities were determined by GC-FID analysis of the crude products mixture using mesitylene as internal standard.

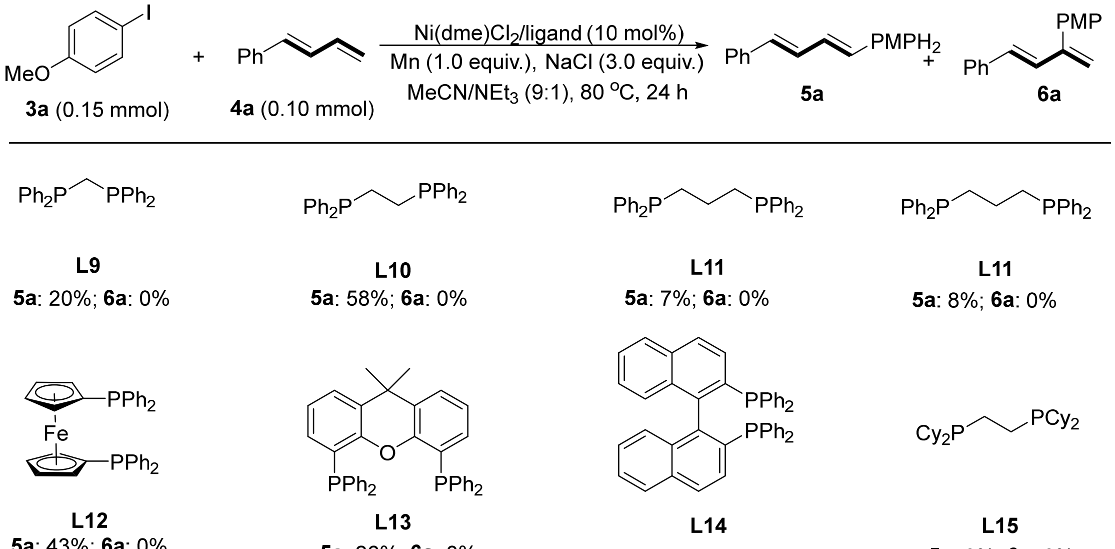

In glove box, a sealed tube was charged with $Ni(dme)Cl_2$ (0.01 mmol, 10 mol%), bisphosphine ligand (0.01 mmol, 10 mol%), Mn (0.10 mmol, 1.0 equiv.), NaCl (0.30 mmol, 3.0 equiv.), **3a** (0.15 mmol) and **4a** (0.10 mmol), MeCN (0.50 mL), NEt_3 (56 uL) at room temperature. The reaction tube was sealed with a Teflon screw cap, removed from the glove box. Then, the reaction mixture was stirred at 80 °C for 24 hours. Yields and selectivities were determined by GC-FID analysis of the crude products mixture using mesitylene as internal standard.

4.4 The capture of Ni (0) species

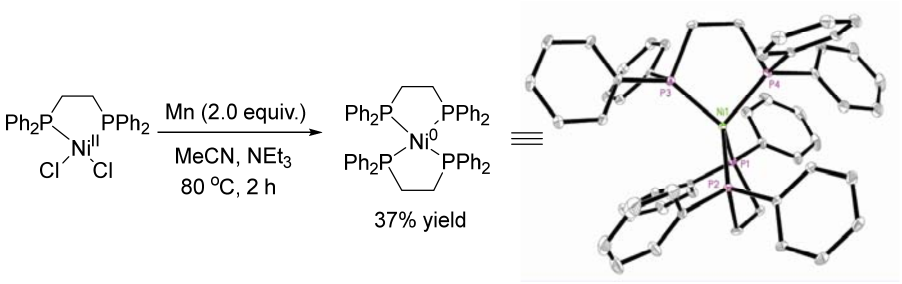

In a glove box, a sealed tube was charged with Ni(dppe)Cl_2 (0.02 mmol), Mn (0.04 mmol), MeCN (2.0 mL), NEt_3 (0.22 mL) at room temperature. The reaction tube was sealed with a Teflon screw cap and the reaction mixture was stirred at 80 ºC for 2 hours. Then, a clear yellow solution was obtained by a hot filtration. The yellow solution was slowly cooled to -18 ºC, upon which time small yellow crystals formed. Followed by decanting the solution and washing with cold MeCN, Ni$(dppe)_2$ (6.4 mg, 37%) was yielded. ^{1}H, ^{31}P NMR spectroscopy and crystal data were consistent with previous reports on this species.[31,32] **^{1}H NMR** (400 MHz, Benzene-d_6) δ 7.48 (s, 8H), 6.94 (s, 12H), 2.10 (s, 4H); **^{31}P NMR** (162 MHz, Benzene-d_6) δ 44.11.

Supplementary Table 17. The data of crystal

Identification code	2095730
Empirical formula	$C_{52}H_{48}NiP_4$
Formula weight	855.49
Temperature/K	170.0
Crystal system	monoclinic
Space group	$P2_1/n$
a/Å	9.754(2)
b/Å	20.953(11)
c/Å	21.285(6)
α/°	90.00
β/°	91.932(6)
γ/°	90.00
Volume/Å^3	4348(3)
Z	4
ρ_{calc}g/cm^3	1.307
μ/mm^{-1}	0.629
F(000)	1792.0
Crystal size/mm^3	0.25 × 0.2 × 0.2
Radiation	MoKα (λ = 0.71073)
2Θ range for data collection/°	4.6 to 55

Index ranges	$-12 \le h \le 12$, $-27 \le k \le 27$, $-27 \le l \le 27$
Reflections collected	57407
Independent reflections	9976 [R_{int} = 0.0827, R_{sigma} = 0.0538]
Data/restraints/parameters	9976/0/514
Goodness-of-fit on F^2	1.023
Final R indexes [I>=2σ (I)]	R_1 = 0.0366, wR_2 = 0.0805
Final R indexes [all data]	R_1 = 0.0645, wR_2 = 0.0893
Largest diff. peak/hole / e Å^{-3}	0.32/-0.26

4.5 The catalytic performance of Ni species

In glove box, a sealed tube was charged with iodobenzene **3a**, [Ni], Mn, NaCl (0.30 mmol, 3.0 equiv.), 1,3-diene **4a** (0.10 mmol), MeCN (0.50 mL), NEt_3 (56 uL) at room temperature. The reaction tube was sealed with a Teflon screw cap, removed from the glove box. Then, the reaction mixture was stirred at 80 °C for 24 hours. The yields were determined by GC-FID analysis of the crude product mixture using mesitylene as internal standard.

Supplementary Table 18. The catalytic performance of Ni species

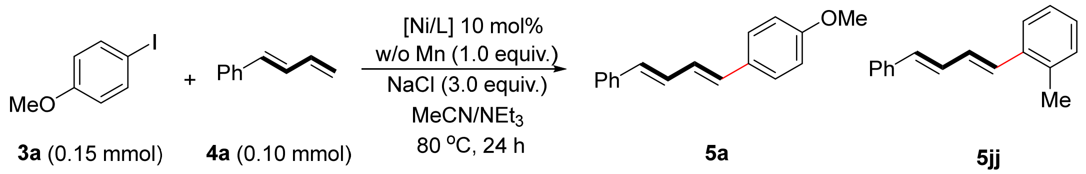

Entry	[Ni/L]	Mn	Yield of **5a** (%)	Yield of **5jj** (%)
1	Ni(dppe)$_2$	1.0 equiv.	43	--
2	Ni(dppe)Cl_2/dppe	1.0 equiv.	48	--
3	Ni(cod)$_2$/dppe	--	22	--
4	Ni(cod)$_2$/dppe	1.0 equiv.	49	--
5	Ni(2-MeC_6H_4)Cl(dppe)	--	6	4
6	Ni(2-MeC_6H_4)Cl(dppe)	1.0 equiv.	57	7
7^a	Ni(2-MeC_6H_4)Cl(dppe)	1.0 equiv.	--	75^b

^{a}Ni(2-MeC_6H_4)Cl(dppe) (1.0 equiv.) was used without **3a**; ^{b}Isolated yield.

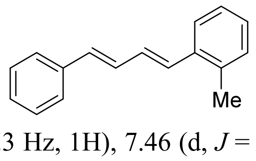

1-Methyl-2-((1*E*,3*E*)-4-phenylbuta-1,3-dien-1-yl)benzene (5jj): Prepared according to the procedure above, known compound,[9] white solid, 35.1 mg, 75% yield, R_f = 0.6 (PE), **^{1}H NMR** (400 MHz, Chloroform-*d*) δ 7.57 (d, *J* = 7.3 Hz, 1H), 7.46 (d, *J* = 7.5 Hz, 2H), 7.35 (t, *J* = 7.6 Hz, 2H), 7.25-7.12 (m, 4H), 7.02 (ddd, *J* = 15.5, 7.7, 2.0 Hz, 1H), 6.95-6.82 (m, 2H), 6.69 (d, *J* = 15.3 Hz, 1H), 2.41 (s, 3H); **^{13}C NMR** (100 MHz, Chloroform-*d*) δ 137.53, 136.30, 135.74, 132.82, 130.62, 130.55, 130.45, 129.76, 128.79, 127.67, 127.62, 126.52, 126.29, 125.16, 20.03.

4.6 ^{31}P NMR spectra of control experiments

The resulting MeCN solutions after filtration (Supplementary Figure 1-4) were transferred into silica tubes with a D_2O tube respectively, sealed with high vacuum grease and removed out of dry box. These samples were measured at room temperature and ^{31}P NMR spectrum were shown in Supplementary Figure 1-4.

In glove box, Ni(dppe)Cl_2 (5.3 mg, 0.01 mmol), and dry MeCN (0.5 mL) were added to a 4-mL screw-capped vial and stirred at 80 ºC for 1 hour. The ^{31}P NMR was shown in Supplementary Figure 1 and a chemical shift at 59.44 ppm was observed.

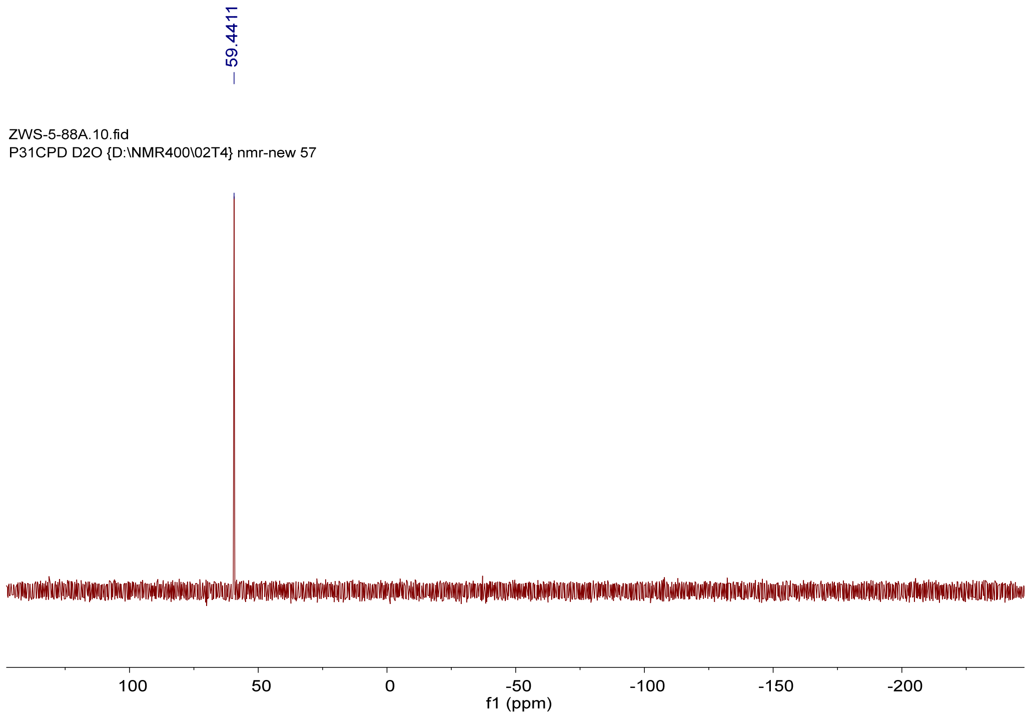

Supplementary Figure 1: ^{31}P NMR of Ni(dppe)Cl_2 in MeCN

In glove box, Ni(dppe)Cl_2 (5.3 mg, 0.01 mmol), and dry NEt_3/MeCN (56 uL/0.5 mL) were added to a 4-mL screw-capped vial and stirred at 80 ºC for 1 hour. The ^{31}P NMR was shown in Supplementary Figure 2 and a chemical shift at 59.35 ppm and a chemical shift at 43.84 ppm were observed.

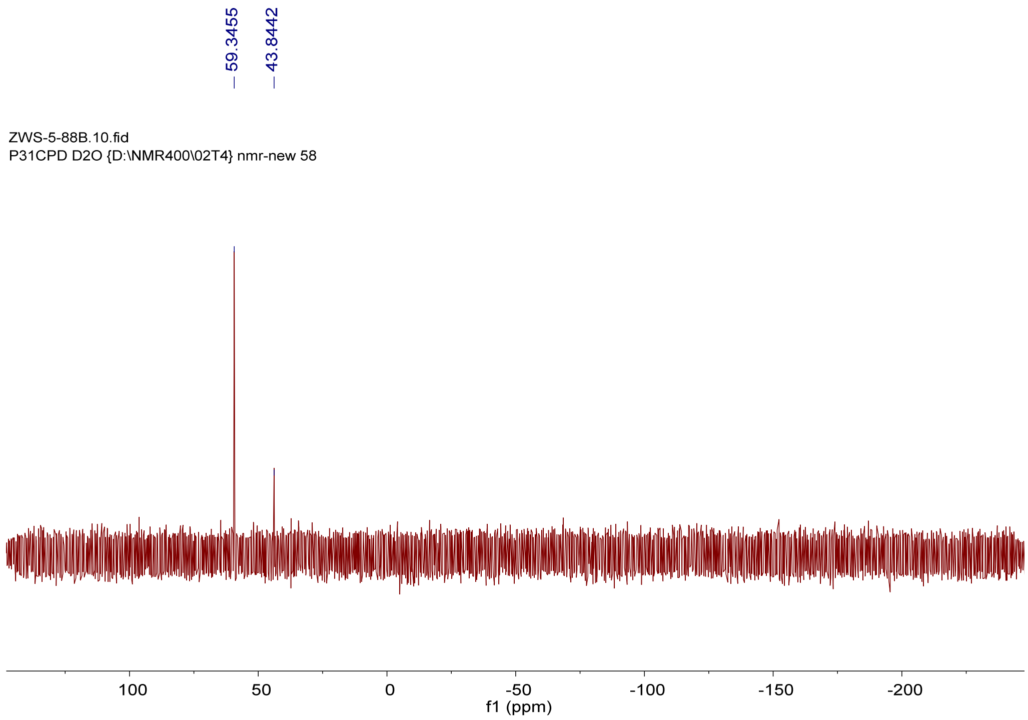

Supplementary Figure 2: ^{31}P NMR of $Ni(dppe)Cl_2$ in NEt_3/MeCN (1/9)

In glove box, $Ni(dppe)Cl_2$ (5.3 mg, 0.01 mmol), Mn (1.1 mg 0.02 mmol) and dry MeCN (0.5 mL) were added to a 4-mL screw-capped vial and stirred at 80 ºC for 1 hour. The ^{31}P NMR was shown in Supplementary Figure 3 and a chemical shift at 60.04 ppm was observed.

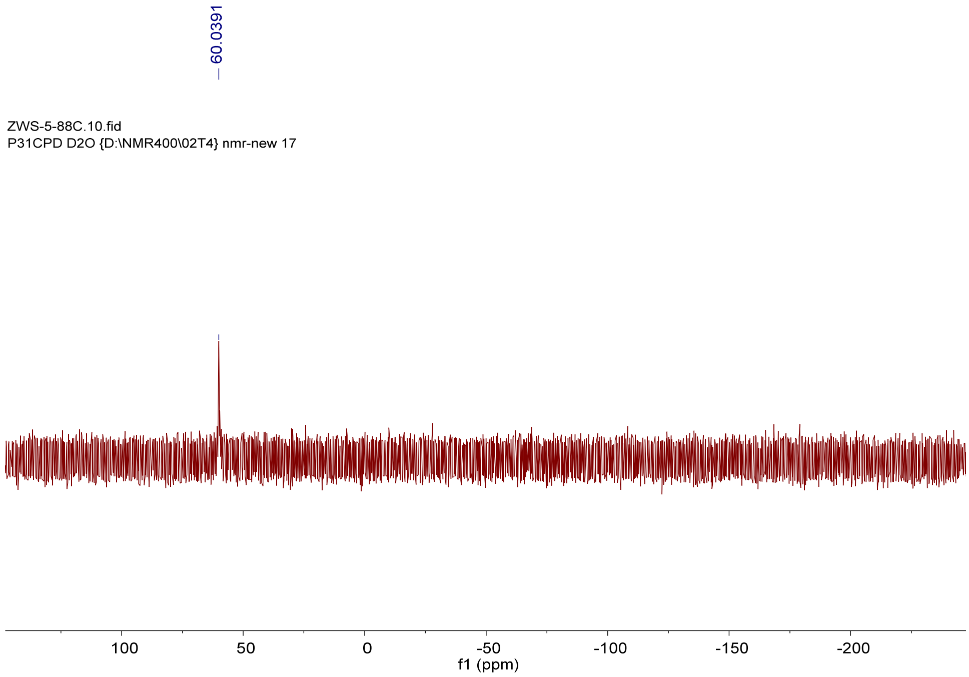

Supplementary Figure 3: ^{31}P NMR of $Ni(dppe)Cl_2$/Mn (1/2) in MeCN

In glove box, $Ni(dppe)Cl_2$ (5.3 mg, 0.01 mmol), Mn (1.1 mg 0.02 mmol) and dry NEt_3/MeCN (56 uL/0.5 mL) were added to a 4-mL screw-capped vial and stirred at 80 ºC for 1 hour. The ^{31}P NMR was shown in Supplementary Figure 4 and a chemical shift at 44.25 ppm was observed.

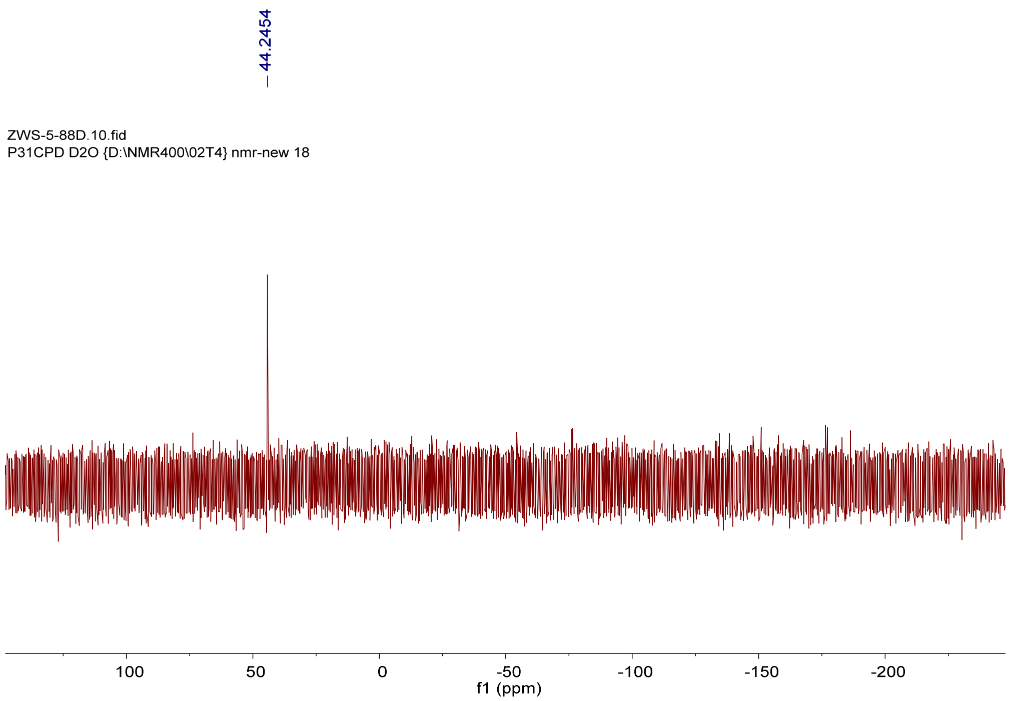

Supplementary Figure 4: ^{31}P NMR of Ni(dppe)Cl_2/Mn (1/2) in NEt_3/MeCN (1/9)

4.7 The EPR spectrum

The resulting MeCN/NEt_3 (9:1) solutions (Supplementary Figure 5-6) were partly transferred into a capillary column (5 cm), respectively, sealed with high vacuum grease and removed out of dry box. The sample was then put into a quartz tube and measured at 292 K (Frequency = 9.325813 GHz, Power = 10.01 mW, Field Center = 3350.000 G, Width +/- = 5000.000 G, Sweep Time = 167.94 sec, Modulation Frequency = 100.00 kHz, Amplitude 2.00 G, Time Constant 40.96 msec, Phase 0.00 deg. The EPR spectra were shown in Supplementary Figure 5-6.

In glove box, Ni(dppe)Cl_2 (5.3 mg, 0.01 mmol) and dry MeCN/NEt_3 (2.0/0.22 mL) were added to a 4-mL screw-capped vial and stirred at 80 ºC for 1 hour. The EPR spectrum was shown in Supplementary Figure 5 and no obvious resonance signal was observed.

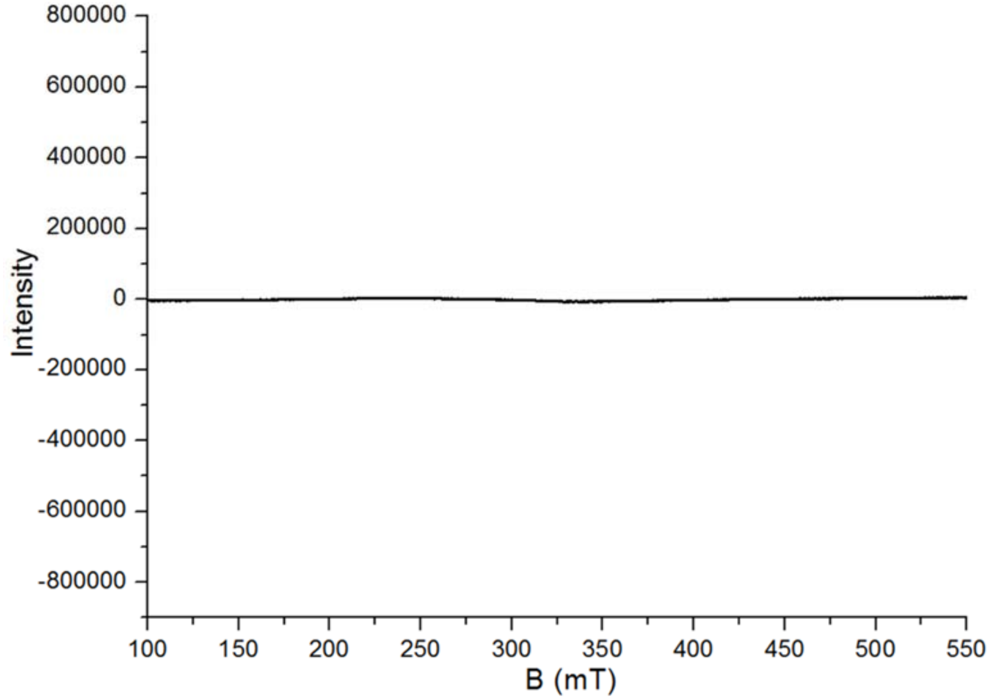

Supplementary Figure 5: EPR spectrum of Ni(dppe)Cl_2 in MeCN/NEt_3 (9:1) (292 K)

In glove box, Ni(dppe)Cl_2 (5.3 mg, 0.01 mmol), Mn (2.2 mg, 0.04 mmol) and dry MeCN/NEt_3 (2.0/0.22 mL) were added to a 4-mL screw-capped vial and stirred at 80 ºC for 1 hour. The EPR spectrum was shown in Supplementary Figure 6 and only symmetric signal of Mn^{2+} was observed.

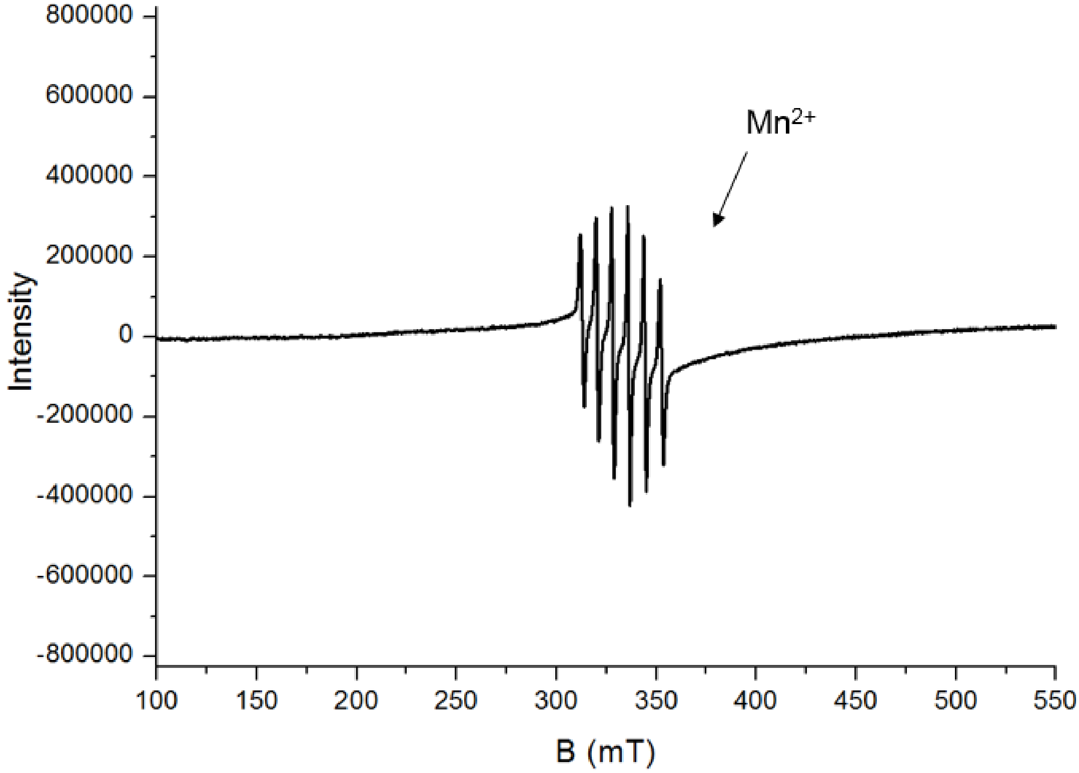

Supplementary Figure 6: EPR spectrum of Ni(dppe)Cl_2/Mn in MeCN/NEt_3 (9:1) (292 K)

4.8 The role [Ar-B] species

Supplementary Table 19. The role [Ar-B] species

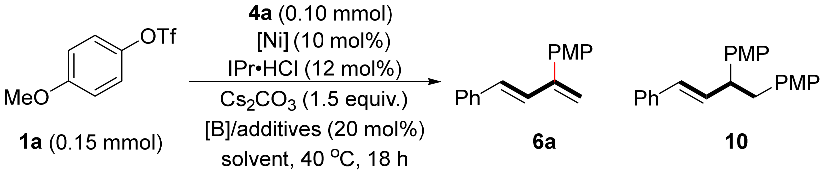

Entry	[Ni]	[B]	additives	solvent	yield of **6a**	yield of **10**
1	$Ni(OTf)_2$	$(Bneop)_2$	NaF	DMF	45%	2%
2	$Ni(OTf)_2$	**B1**	NaF	DMF	0%	0%
3	$Ni(cod)_2$	--	NaF	DMF	<1%	4%
4	$Ni(cod)_2$	--	NaF	MeCN	4%	<1%
5	$Ni(cod)_2$	--	NaF	THF	3%	0%
6	$Ni(cod)_2$	--	NaF	PhMe	5%	0%
7	$Ni(cod)_2$	--	NaF	1,4-dioxane	5%	0%
8	$Ni(cod)_2$	--	NaF	$CH_2(OCH_3)_2$	28%	0%
9[a]	$Ni(cod)_2$	**B1**	NaF	$CH_2(OCH_3)_2$	51%	0%
10	$Ni(cod)_2$	--	NaF	DME	30%	<1%
11	$Ni(cod)_2$	$^{i}PrOB(OMe)_2$	--	DMF	0%	2%
12	$Ni(cod)_2$	$^{i}PrOB(OMe)_2$	NaF	DMF	2%	3%
13	$Ni(cod)_2$	$^{i}PrOB(OMe)_2$	$KO^{t}Bu$	DMF	41%	0%

[a]About 8% yield of hydroarylation product **8** was detected.

In a glove box, $Ni(cod)_2$ (0.01 mmol, 10 mol%), IPr·HCl (0.012 mmol, 12 mol%), Cs_2CO_3 (0.15 mmol, 1.5 equiv.), [B]/additives (0.02 mmol, 20 mol%), Ar-OTf **1a** (0.15 mmol) and dienes **4a** (0.10 mmol) were added to solvent (0.50 mL) in sequence. The reaction tube was sealed with a Teflon screw cap, removed from the glove box. Then, the reaction mixture was stirred at 40 ºC for 18 hours. The yields of **6a** and **10** were determined by GC-FID analysis of the crude product mixture using mesitylene as internal standard.

4.9 The detection of Heck product from aryl boron

Supplementary Table 20. The detection of Heck product from aryl boron

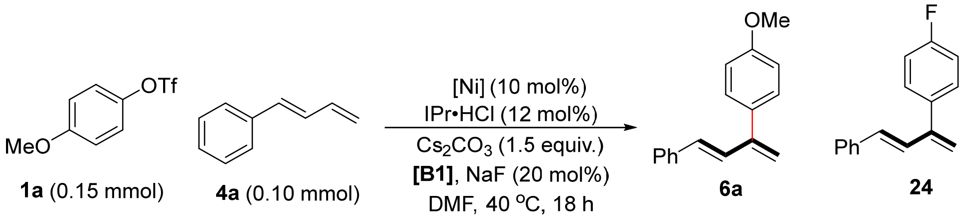

Entry	**[B1]**	yield of **6a**	yield of **24**
1	20 mol%	67%	0%
2	50 mol%	54%	0%
3	100 mol%	46%	0%

In a glove box, $Ni(cod)_2$ (0.01 mmol, 10 mol%), IPr·HCl (0.012 mmol, 12 mol%), Cs_2CO_3 (0.15 mmol, 1.5 equiv.), [B1], NaF (0.02 mmol, 20 mol%), Ar-OTf **1a** (0.15 mmol) and dienes **4a** (0.10 mmol) were added to DMF (0.50 mL) in sequence. The reaction tube was sealed with a Teflon screw cap, removed

from the glove box. Then, the reaction mixture was stirred at 40 ºC for 18 hours. The yields of **6a** and **24** were determined by GC-FID analysis of the crude product mixture using mesitylene as internal standard. With the increasing of **B1**, there was no any Heck product **24** was detected.

4.10 ^{19}F NMR and ^{11}B NMR spectra of control experiments

The resulting DMF solutions were transferred into silica tubes with a D_2O tube respectively, sealed with high vacuum grease and removed out of dry box. These samples were measured at room temperature. The ^{19}F NMR spectrum and ^{11}B NMR spectrum were shown in Supplementary Figure 7-14.

In glove box, **B1** (10.4 mg, 0.05 mmol), and dry DMF (0.5 mL) were added to a 4-mL screw-capped vial and stirred at 40 ºC for 4 hours. The ^{19}F NMR was shown in Supplementary Figure 7 and a chemical shift at -110.67 ppm was observed. The ^{11}B NMR was shown in Supplementary Figure 8 and a chemical shift at -27.04 ppm was observed.

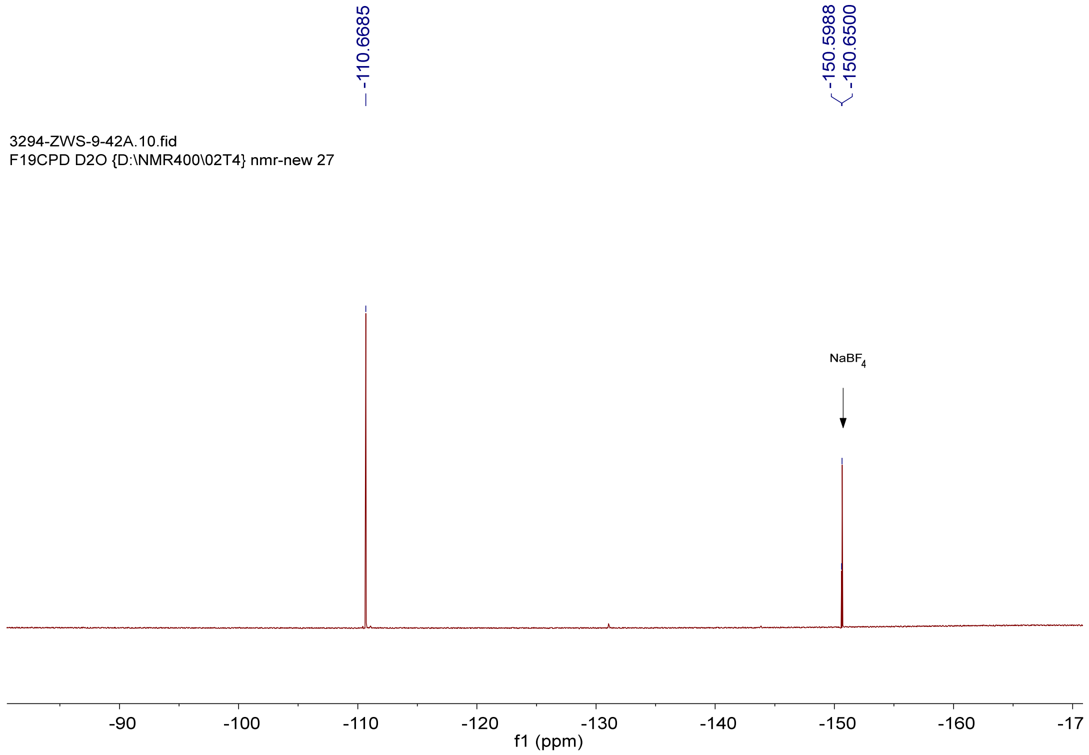

Supplementary Figure 7: ^{19}F NMR of **B1** in DMF

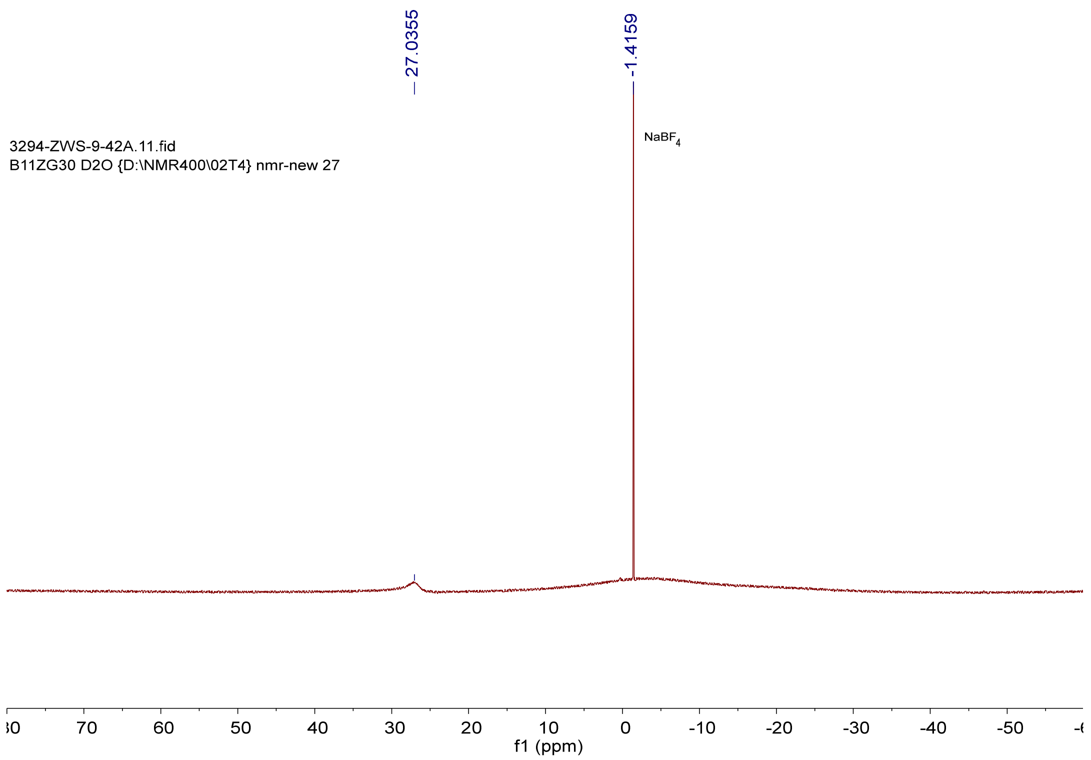

Supplementary Figure 8: ^{11}B NMR of **B1** in DMF

In glove box, Ni(OTf)$_2$ (17.8 mg, 0.05 mmol), and dry DMF (0.5 mL) were added to a 4-mL screw-capped vial and stirred at 40 ºC for 4 hours. The ^{19}F NMR was shown in Supplementary Figure 9 and a chemical shift at -77.40 ppm was observed. There was no other signal except for the $NaBF_4$ in ^{11}B NMR in Supplementary Figure 10.

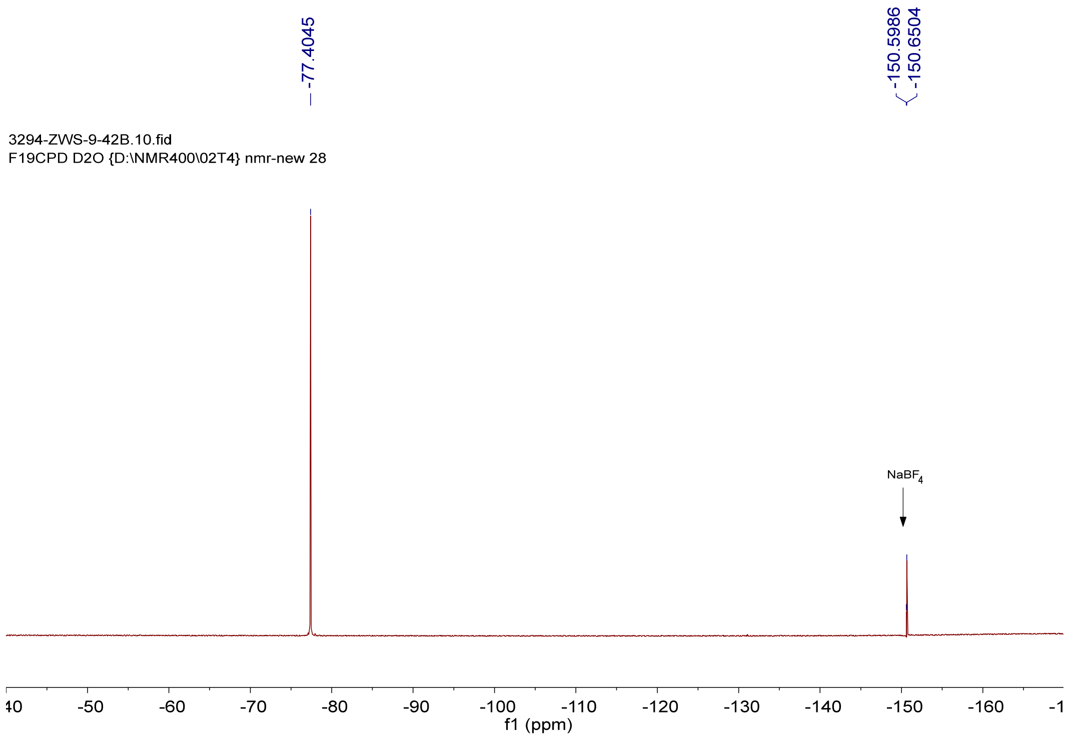

Supplementary Figure 9: ^{19}F NMR of Ni(OTf)$_2$ in DMF

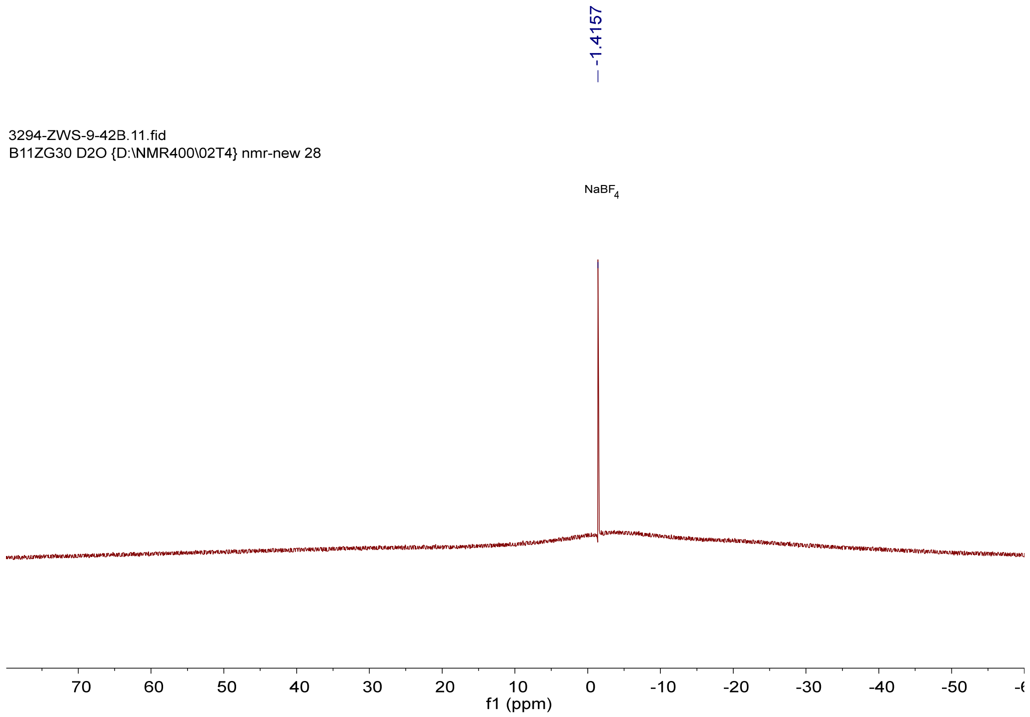

Supplementary Figure 10: ^{11}B NMR of $Ni(OTf)_2$ in DMF

In glove box, $Ni(OTf)_2$ (17.8 mg, 0.05 mmol), **B1** (10.4 mg, 0.05 mmol) and dry DMF (0.5 mL) were added to a 4-mL screw-capped vial and stirred at 40 ºC for 4 hours. The ^{19}F NMR was shown in Supplementary Figure 11 and chemical shifts at -77.24 ppm and -109.76 ppm were observed. The ^{11}B NMR was shown in Supplementary Figure 12 and chemical shifts at 27.85 ppm was observed.

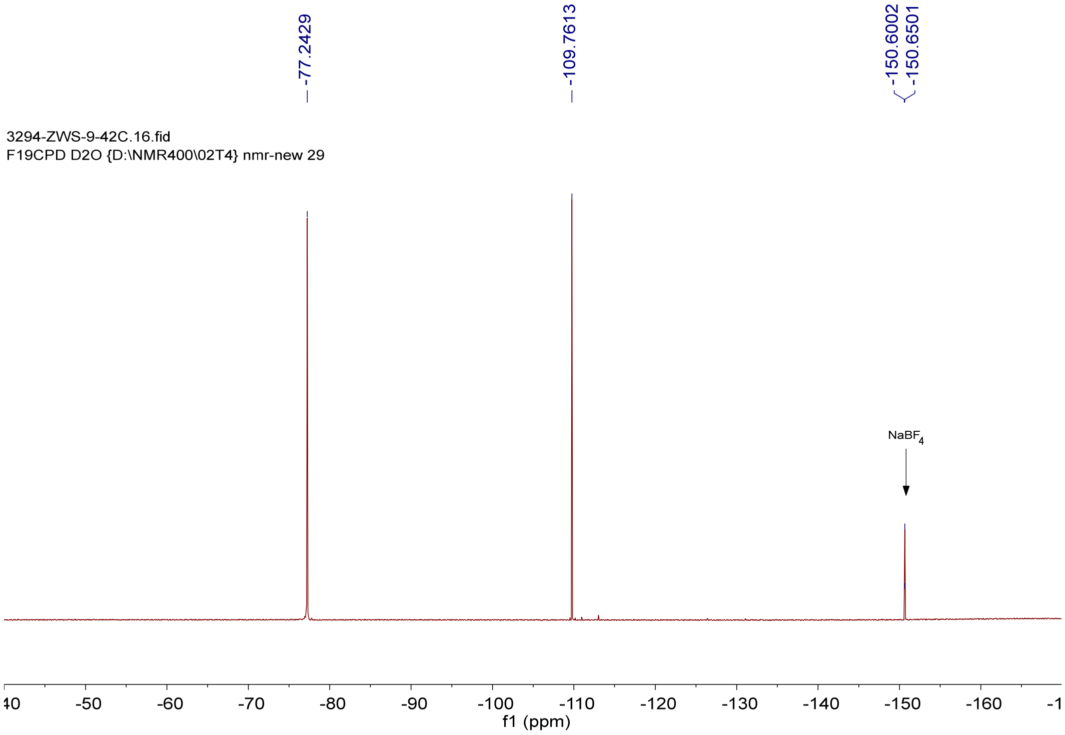

Supplementary Figure 11: ^{19}F NMR of $Ni(OTf)_2$/**B1** (1:1) in DMF

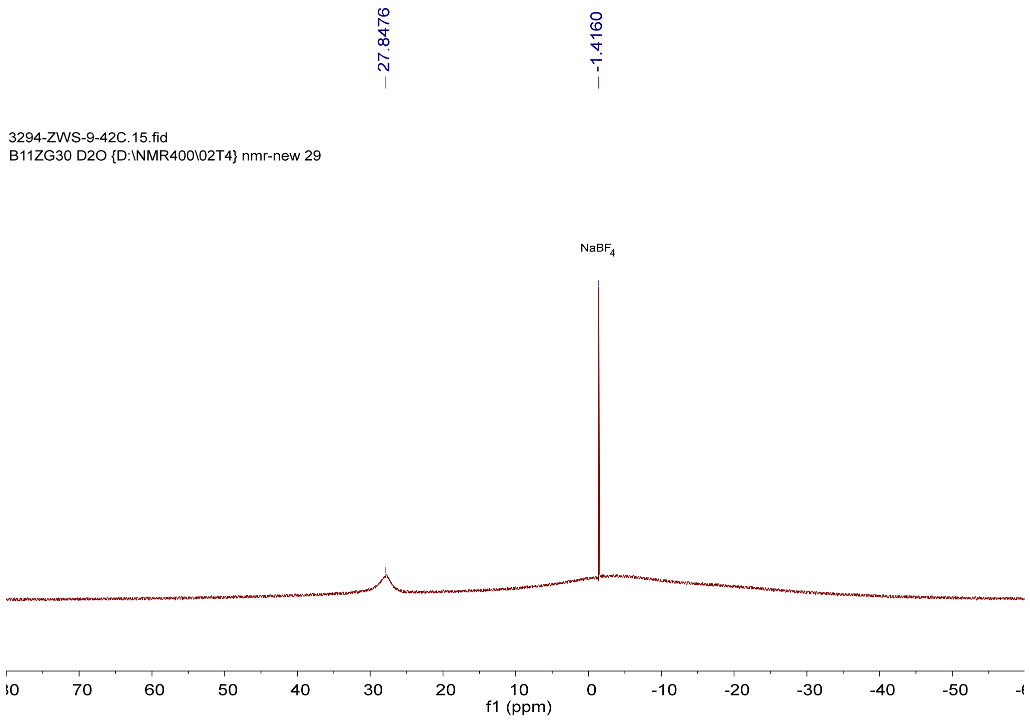

Supplementary Figure 12: ^{11}B NMR of Ni(OTf)$_2$/**B1** (1:1) in DMF

In glove box, Ni(cod)$_2$ (13.8 mg, 0.05 mmol), **B1** (10.4 mg, 0.05 mmol) and dry DMF (0.5 mL) were added to a 4-mL screw-capped vial and stirred at 40 ºC for 4 hours. The ^{19}F NMR was shown in Supplementary Figure 13 and a chemical shift at -110.62 ppm was observed. The ^{11}B NMR was shown in Supplementary Figure 14 and chemical shifts at 27.03 ppm was observed.

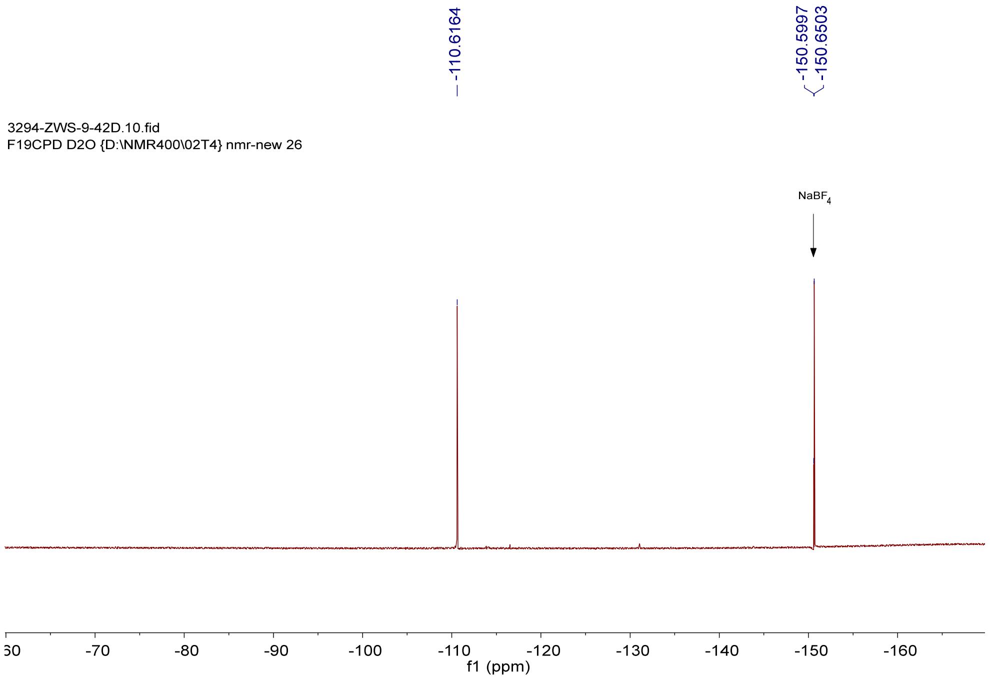

Supplementary Figure 13: ^{19}F NMR of Ni(cod)$_2$/**B1** (1:1) in DMF

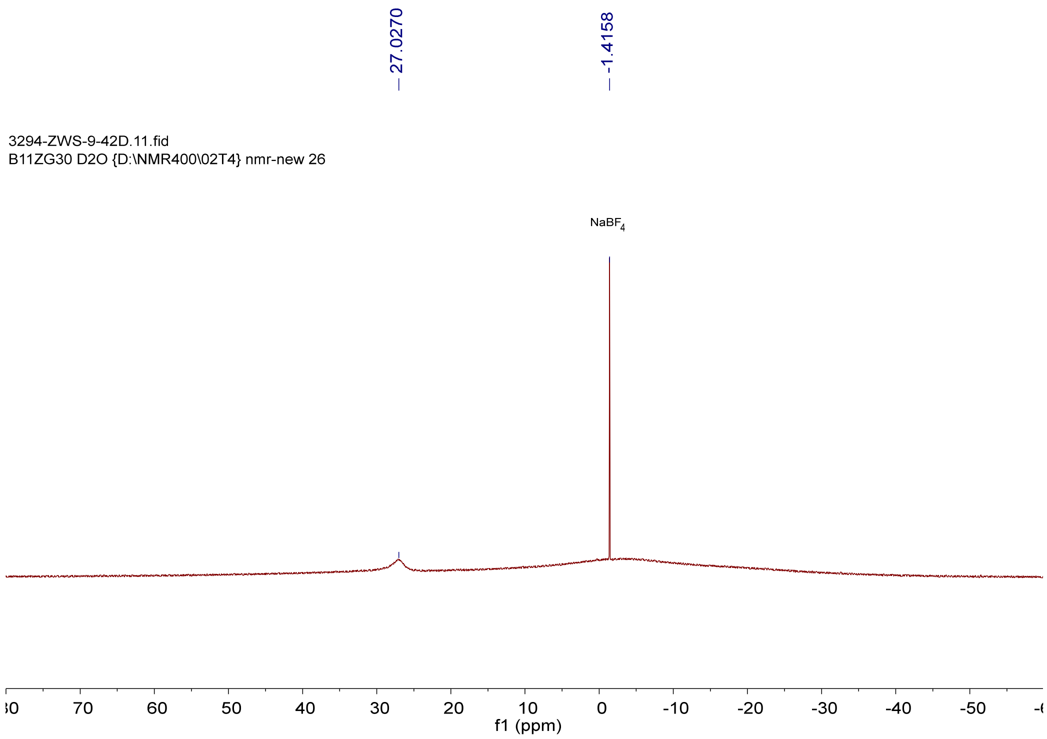

Supplementary Figure 14: ^{11}B NMR of Ni(cod)$_2$/**B1** (1:1) in DMF

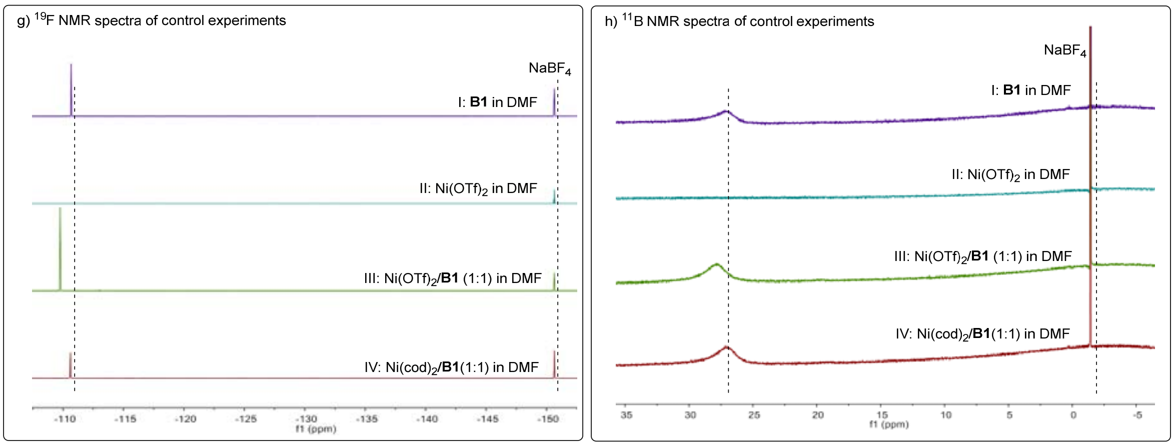

Supplementary Figure 15: The ^{19}F NMR and ^{11}B NMR of control experiments

4.11 Copy of NMR spectra

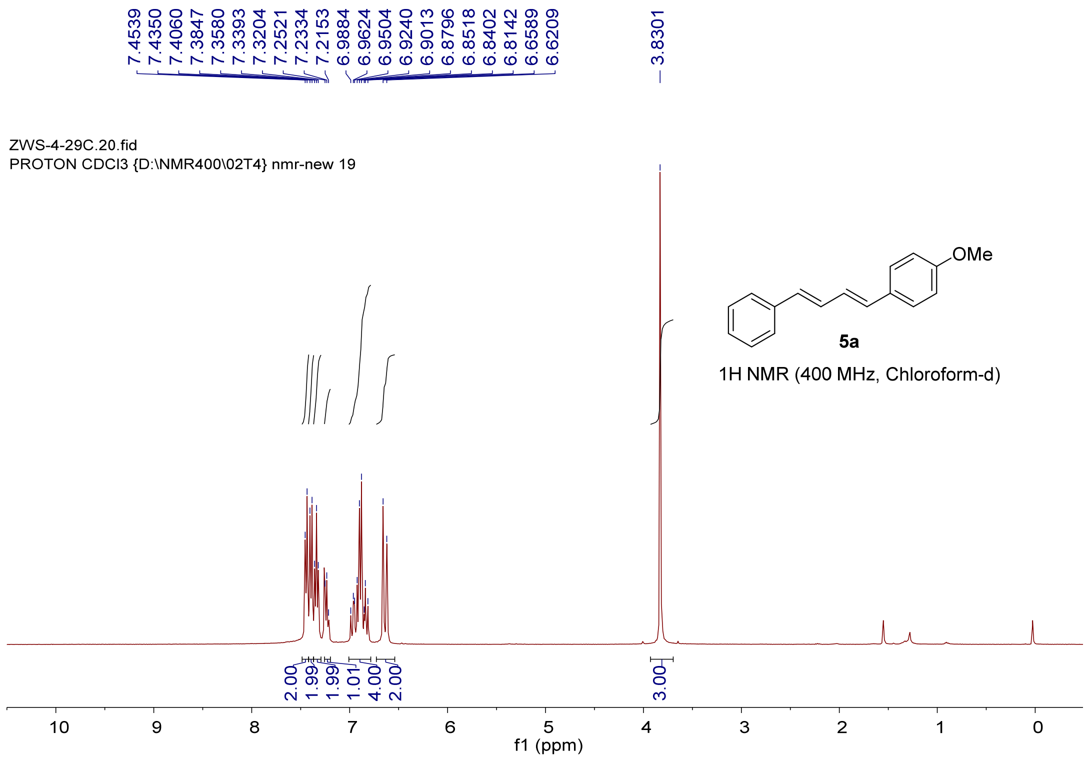

Supplementary Figure 16. ^{1}**H NMR** of compound **5a**

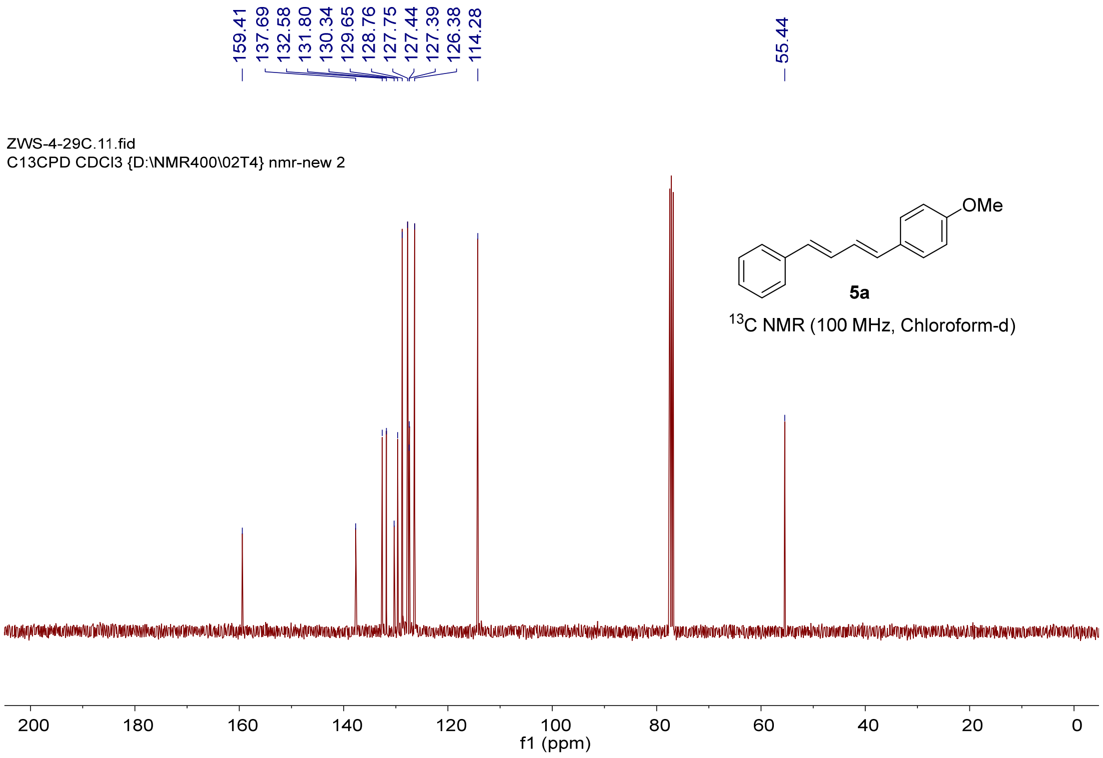

Supplementary Figure 17. ^{13}**C NMR** of compound **5a**

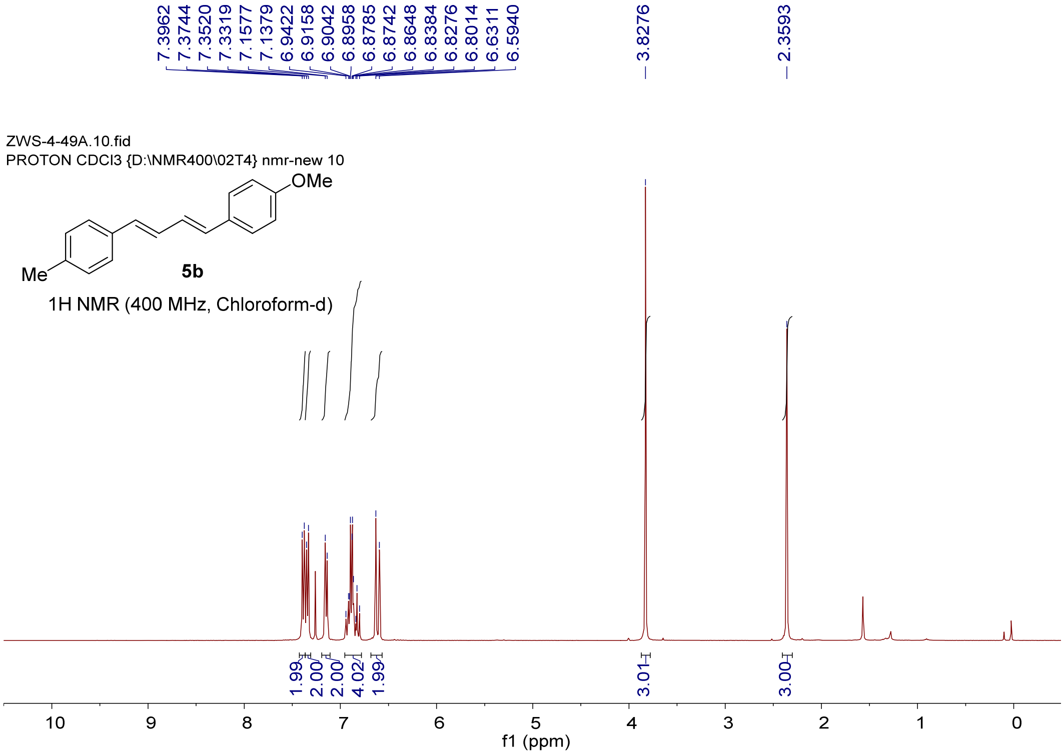

Supplementary Figure 18. **^{1}H NMR** of compound **5b**

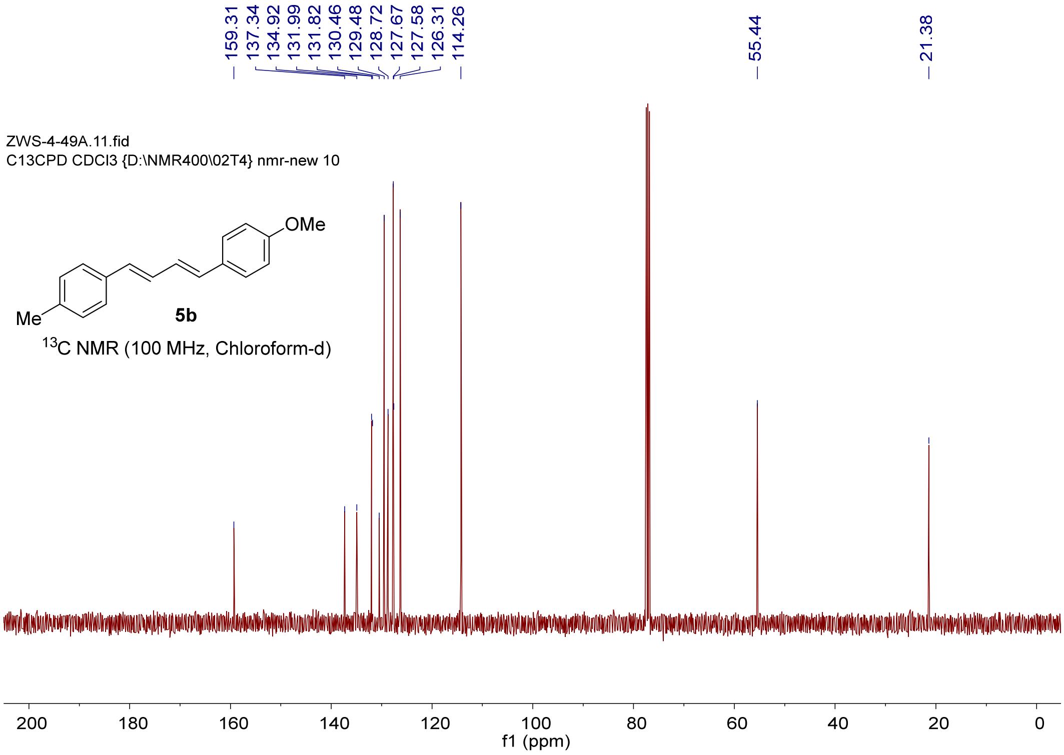

Supplementary Figure 19. **^{13}C NMR** of compound **5b**

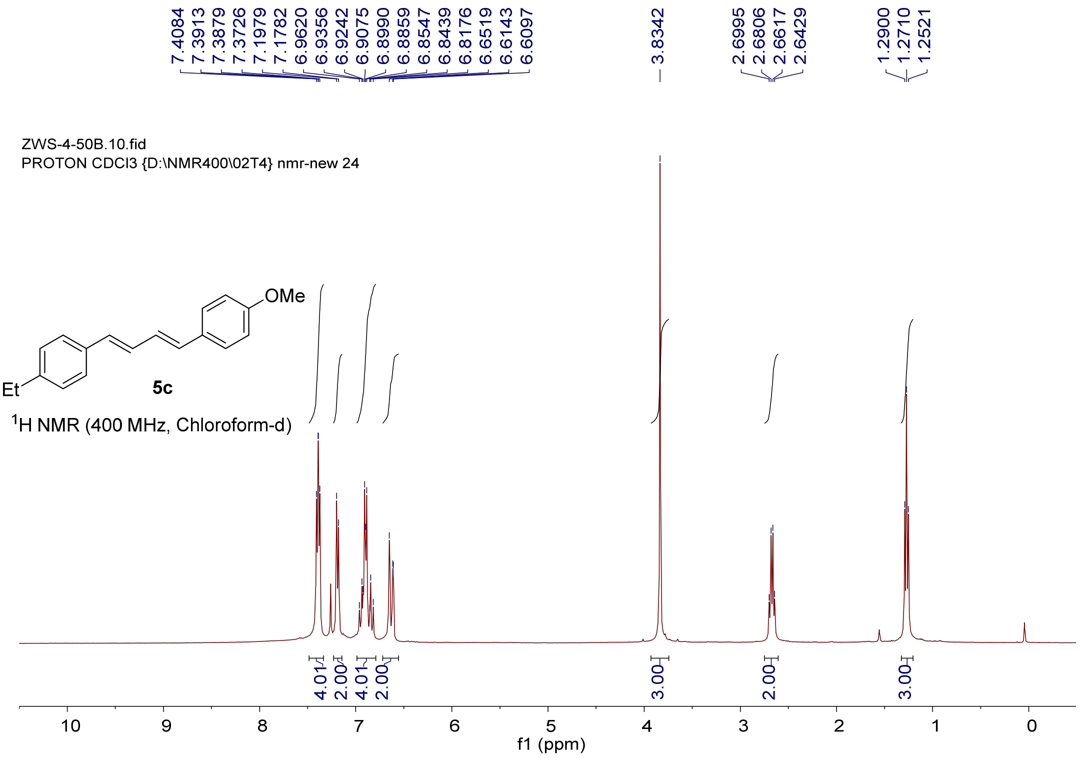

Supplementary Figure 20. ^{1}**H NMR** of compound **5c**

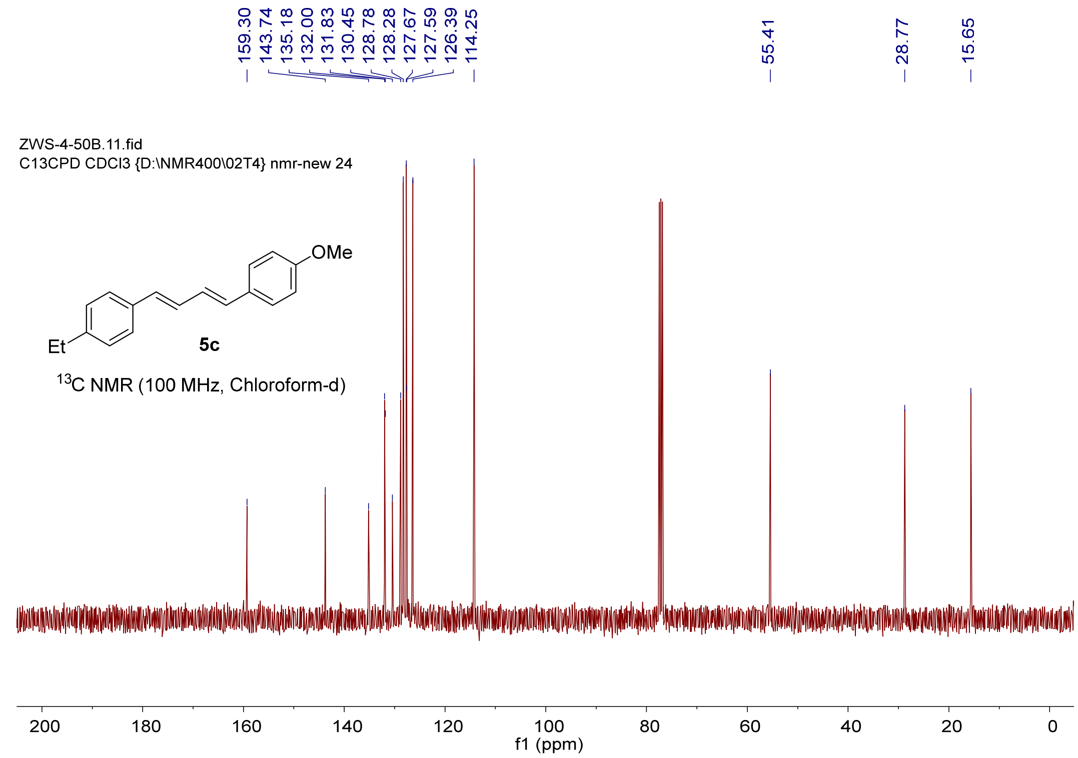

Supplementary Figure 21. ^{13}**C NMR** of compound **5c**

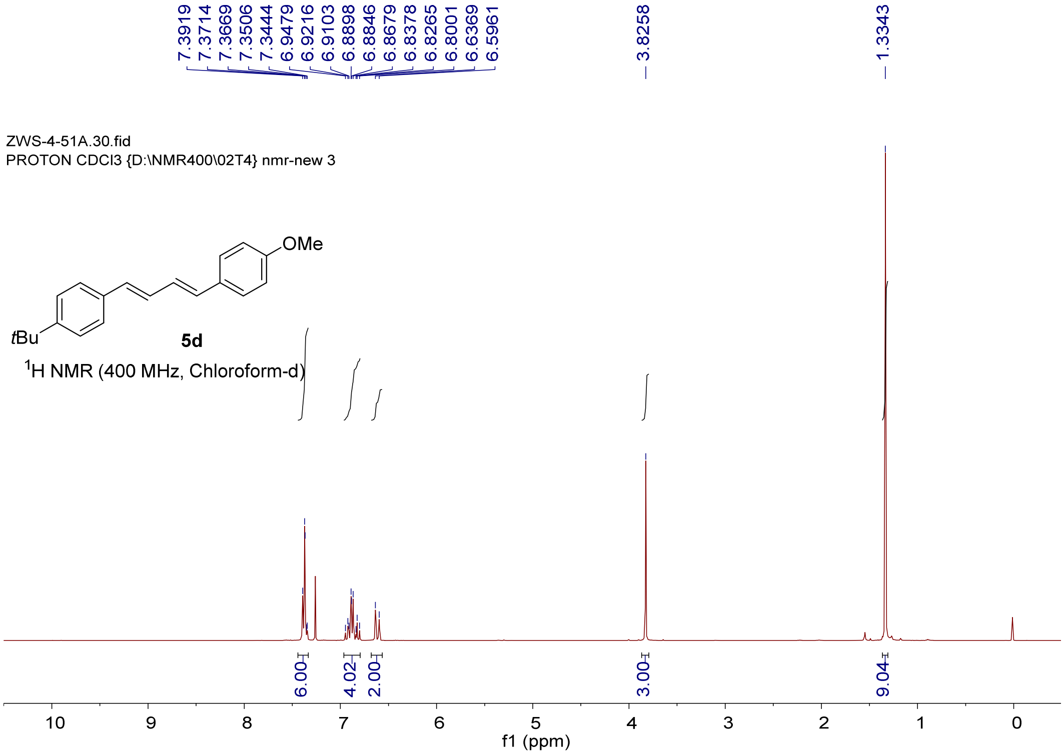

Supplementary Figure 22. ^{1}H NMR of compound **5d**

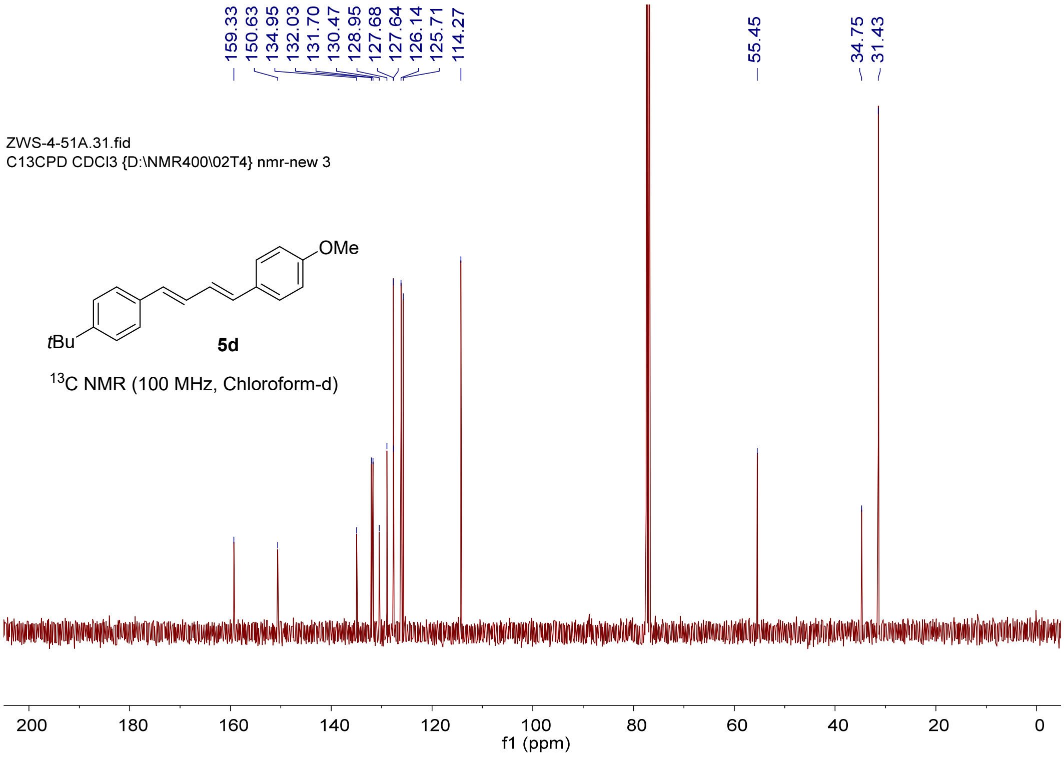

Supplementary Figure 23. ^{13}C NMR of compound **5d**

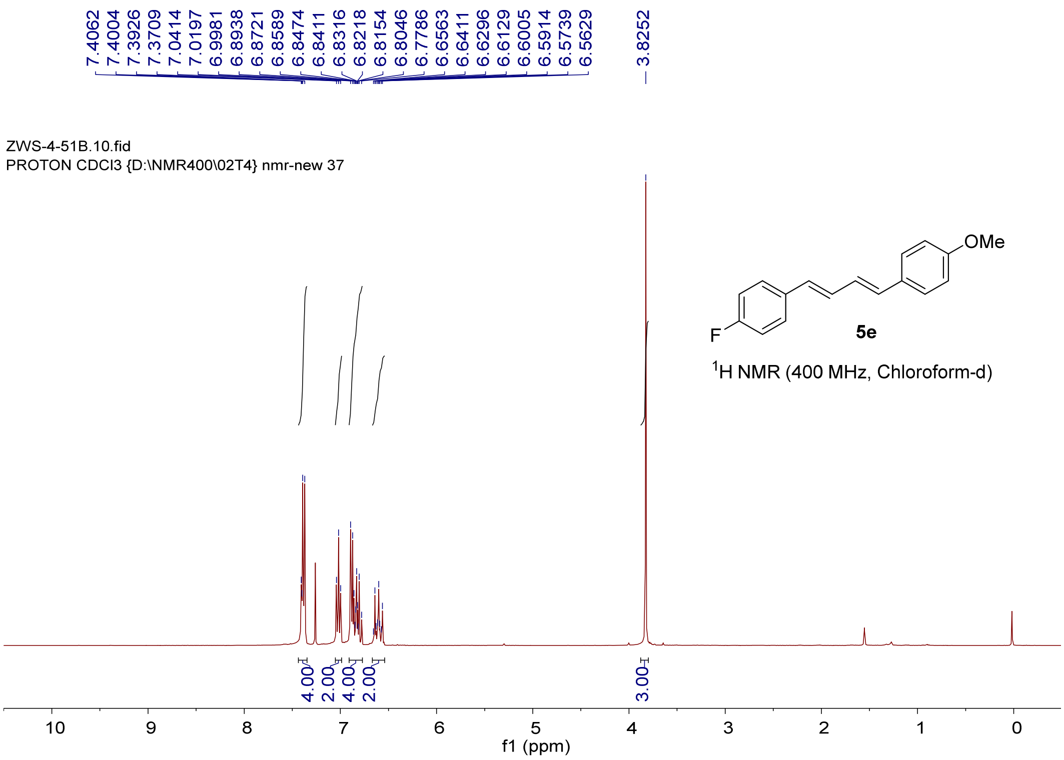

Supplementary Figure 24. ^{1}**H NMR** of compound **5e**

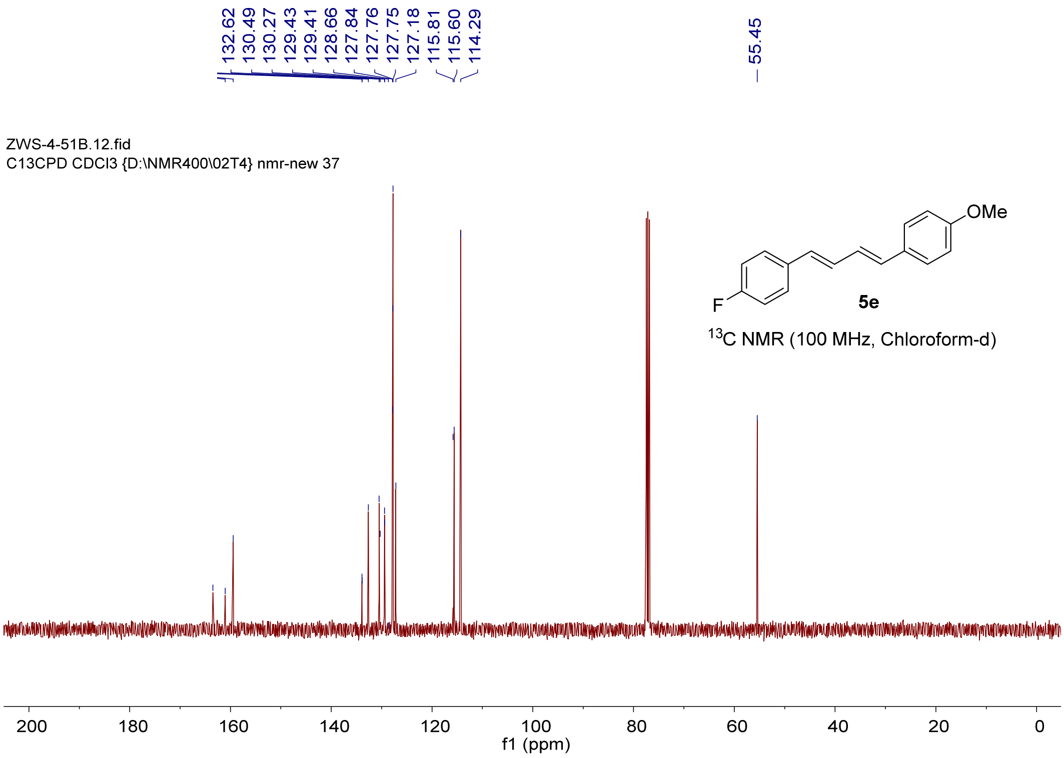

Supplementary Figure 25. ^{13}**C NMR** of compound **5e**

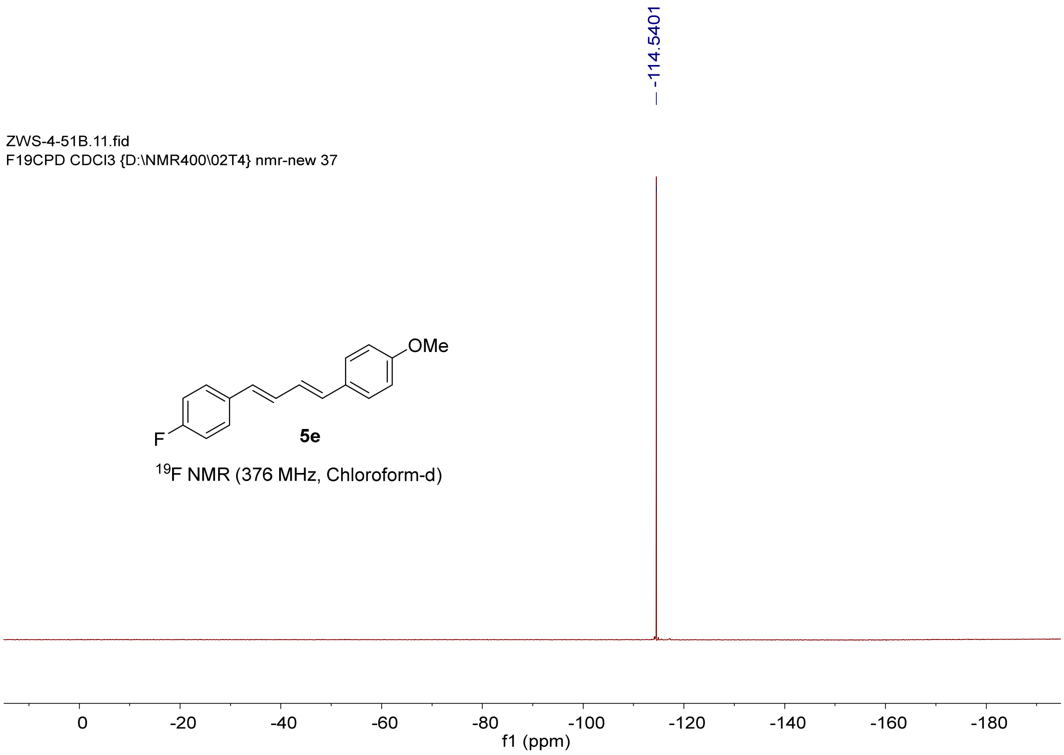

Supplementary Figure 26. ^{19}**F NMR** of compound **5e**

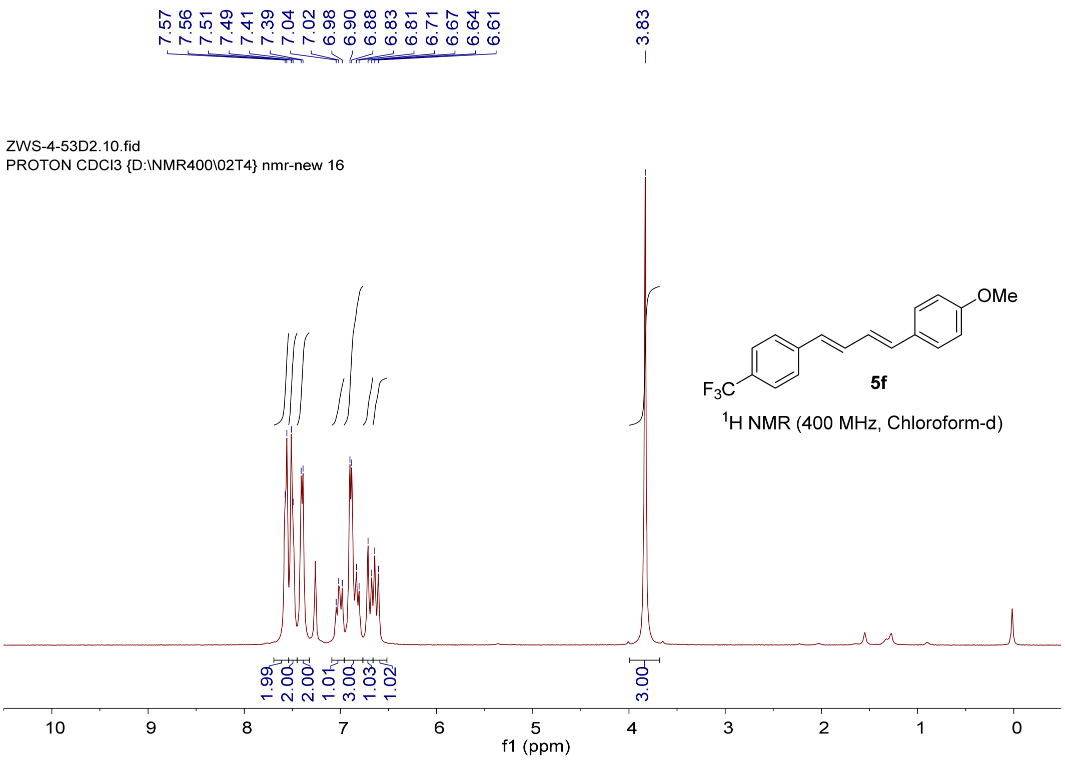

Supplementary Figure 27. **^{1}H NMR** of compound **5f**

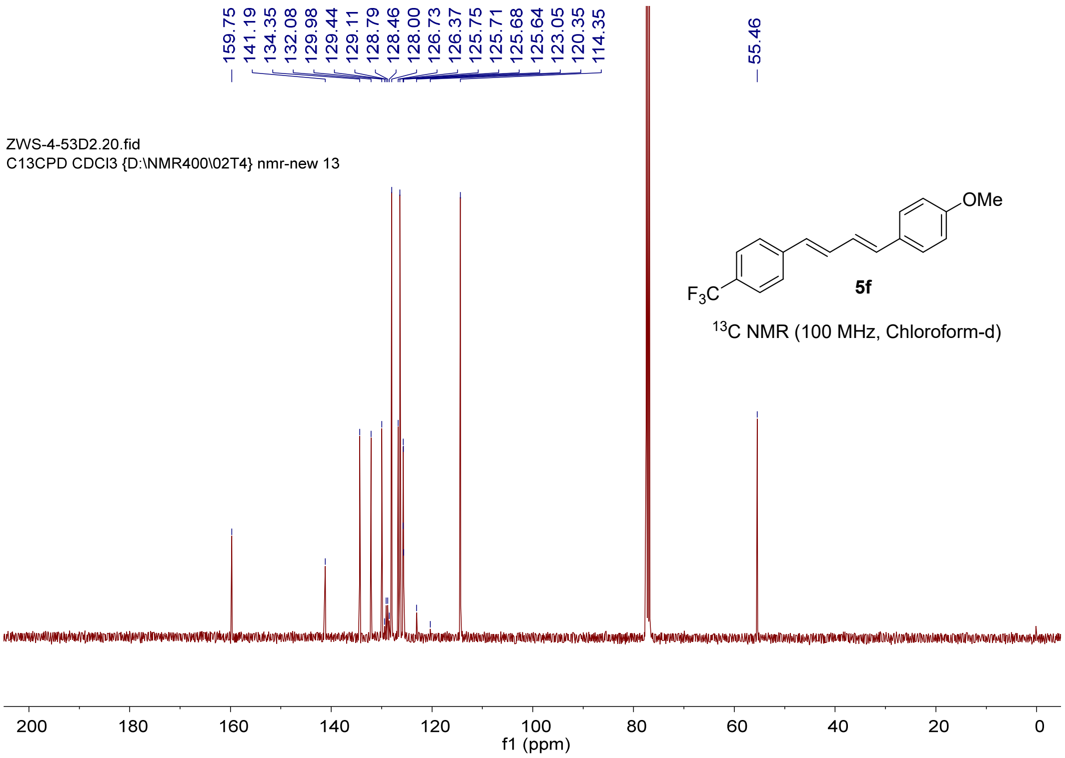

Supplementary Figure 28. **^{13}C NMR** of compound **5f**

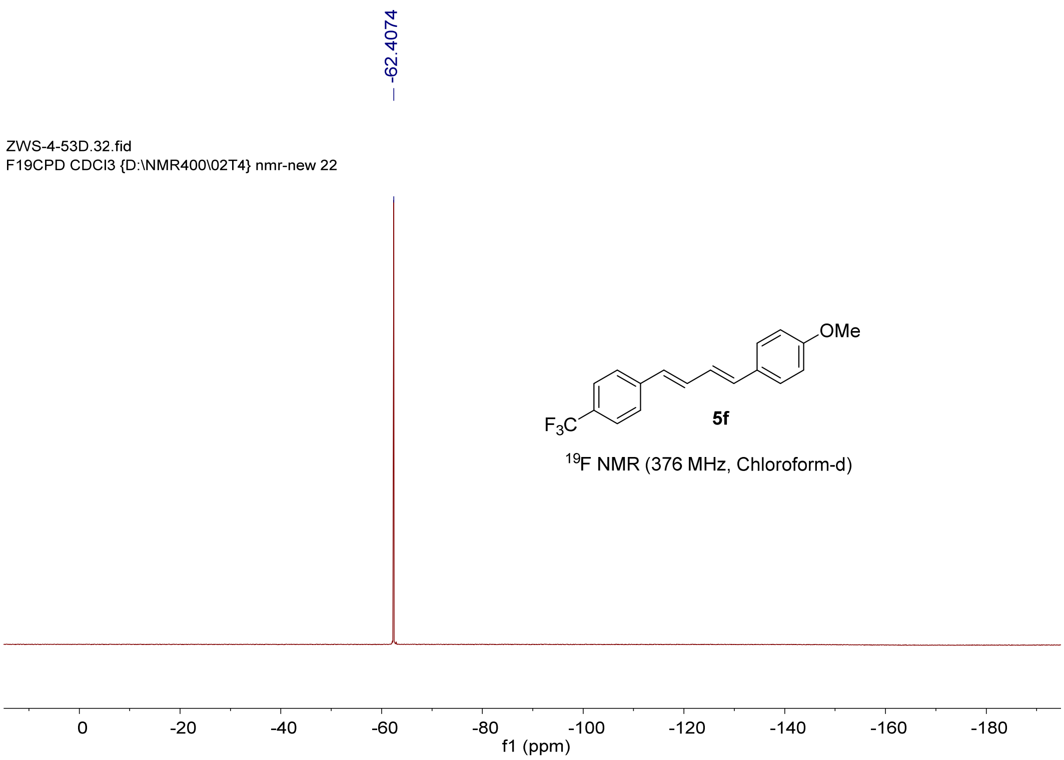

Supplementary Figure 29. ^{19}F NMR of compound **5f**

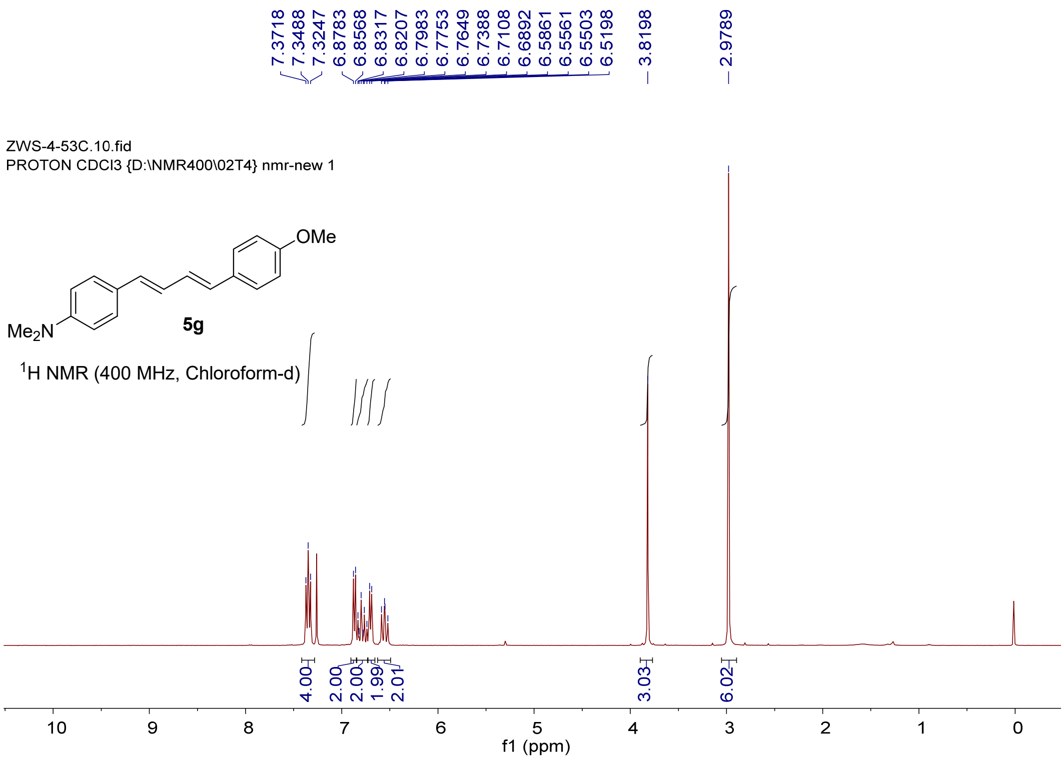

Supplementary Figure 30. **^{1}H NMR** of compound **5g**

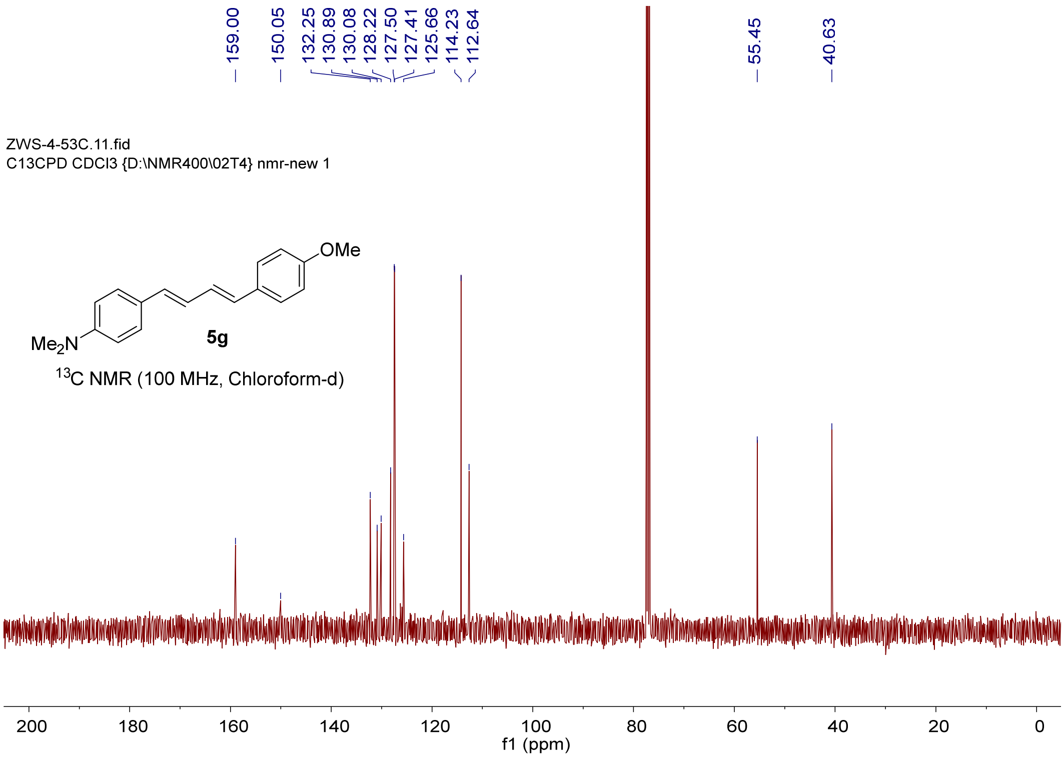

Supplementary Figure 31. **^{13}C NMR** of compound **5g**

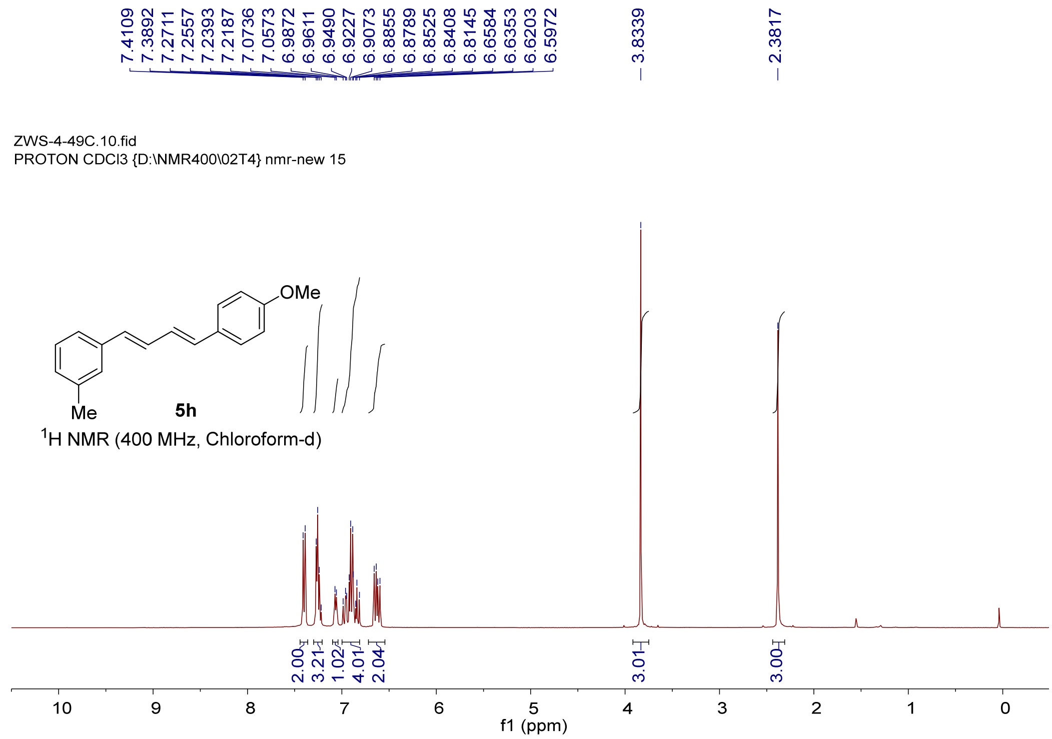

Supplementary Figure 32. **^{1}H NMR** of compound **5h**

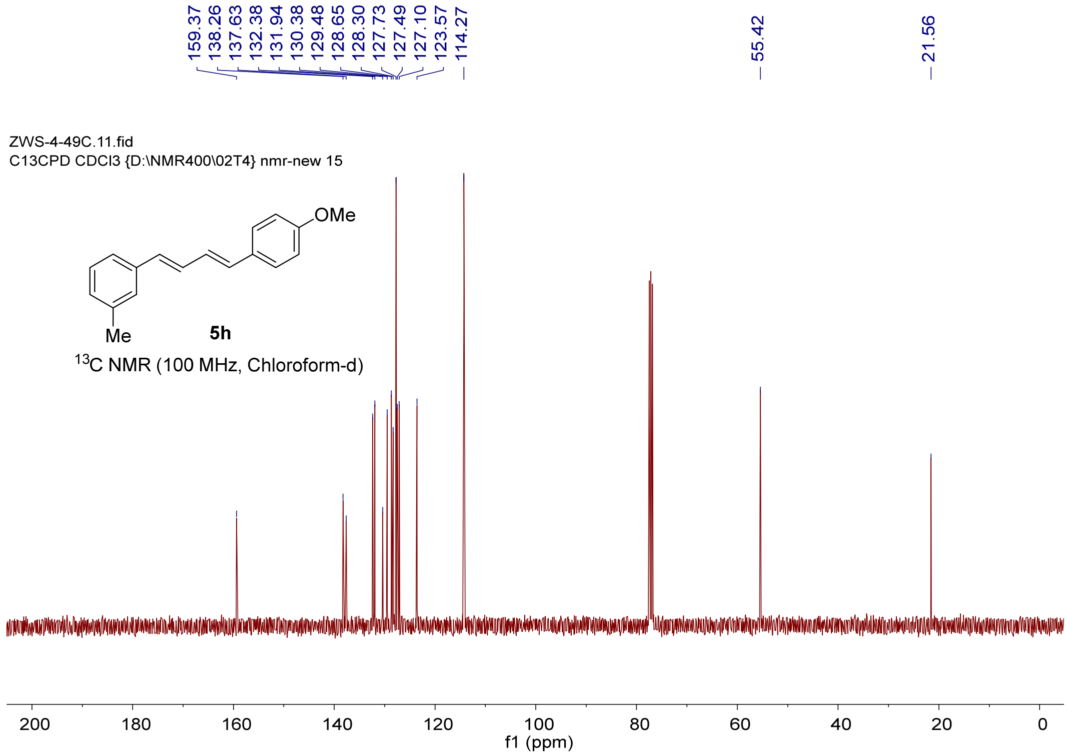

Supplementary Figure 33. **^{13}C NMR** of compound **5h**

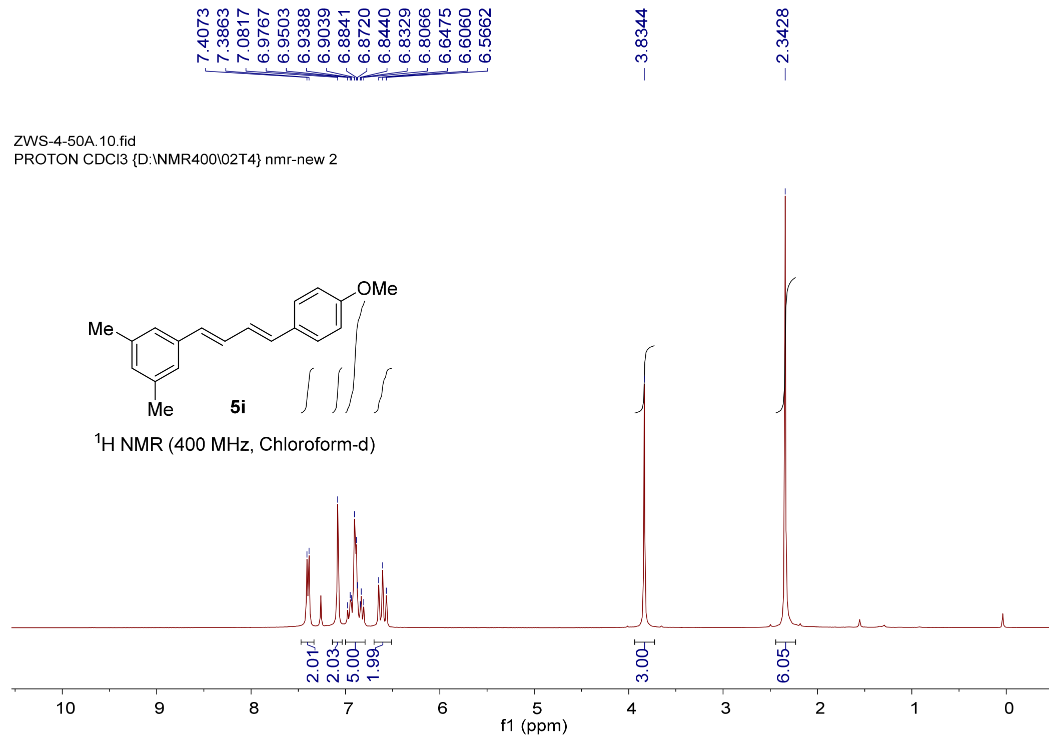

Supplementary Figure 34. ^{1}**H NMR** of compound **5i**

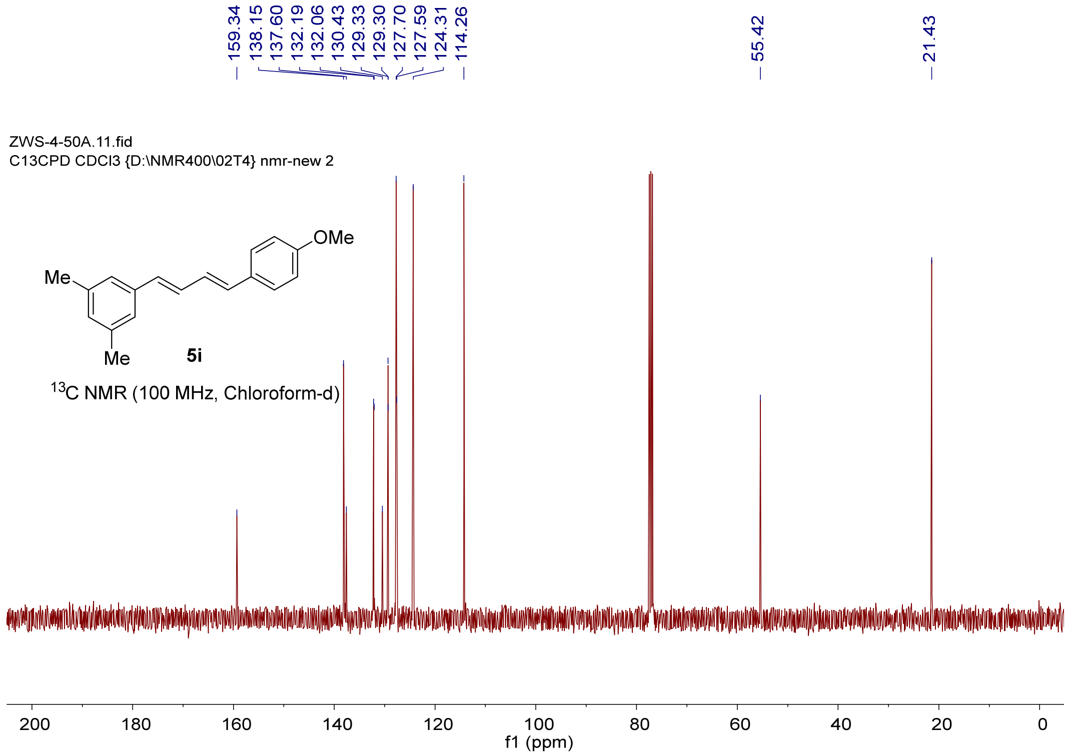

Supplementary Figure 35. ^{13}**C NMR** of compound **5i**

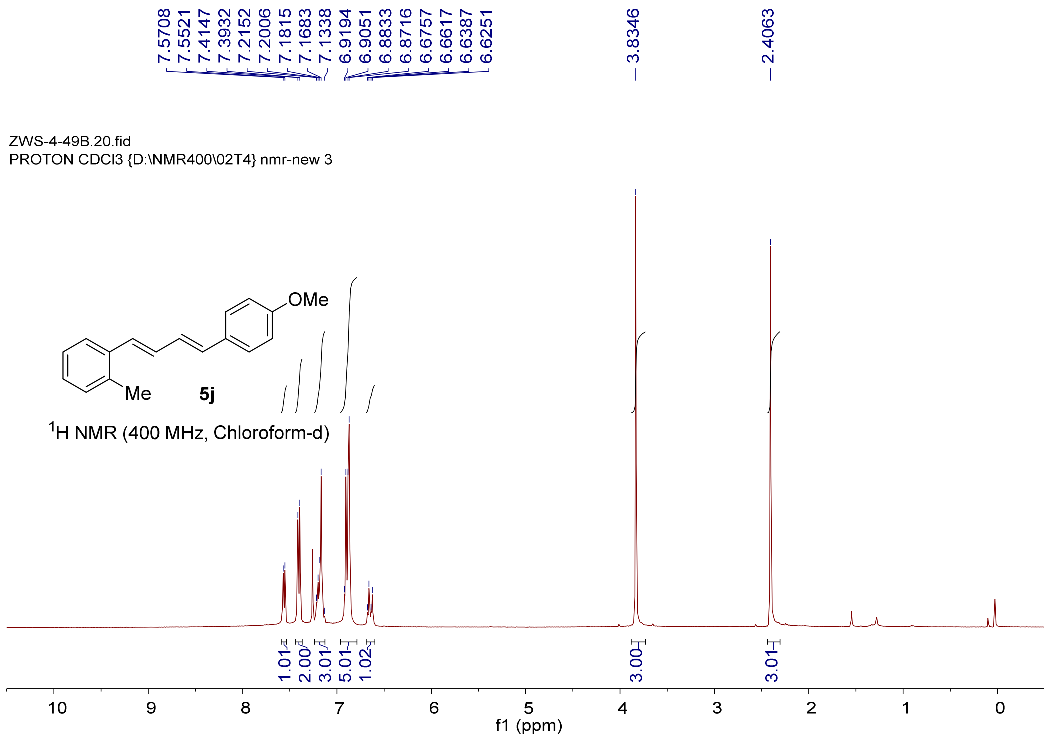

Supplementary Figure 36. ^{1}H NMR of compound **5j**

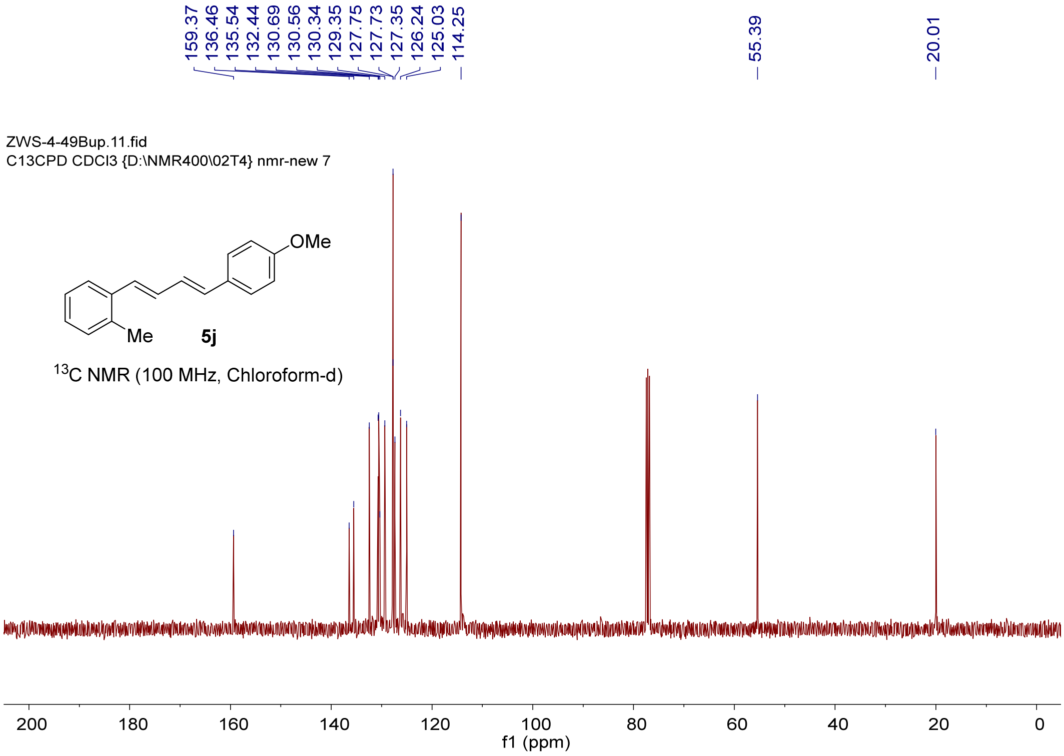

Supplementary Figure 37. ^{13}C NMR of compound **5j**

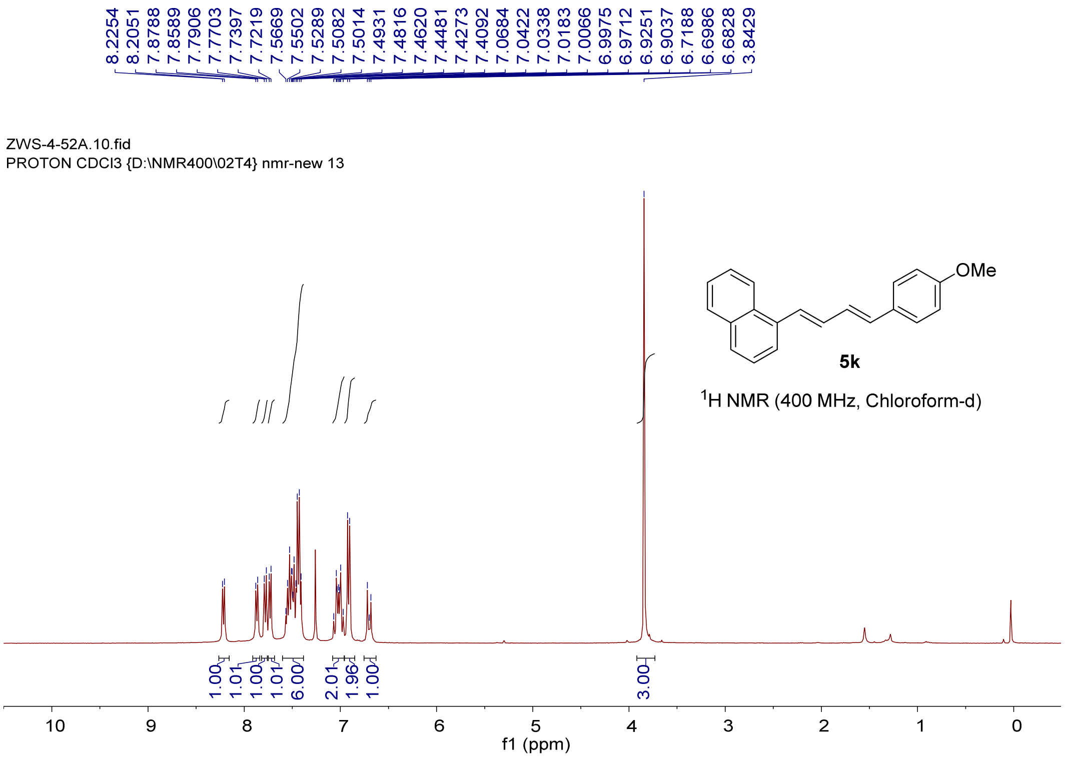

Supplementary Figure 38. ^{1}**H NMR** of compound **5k**

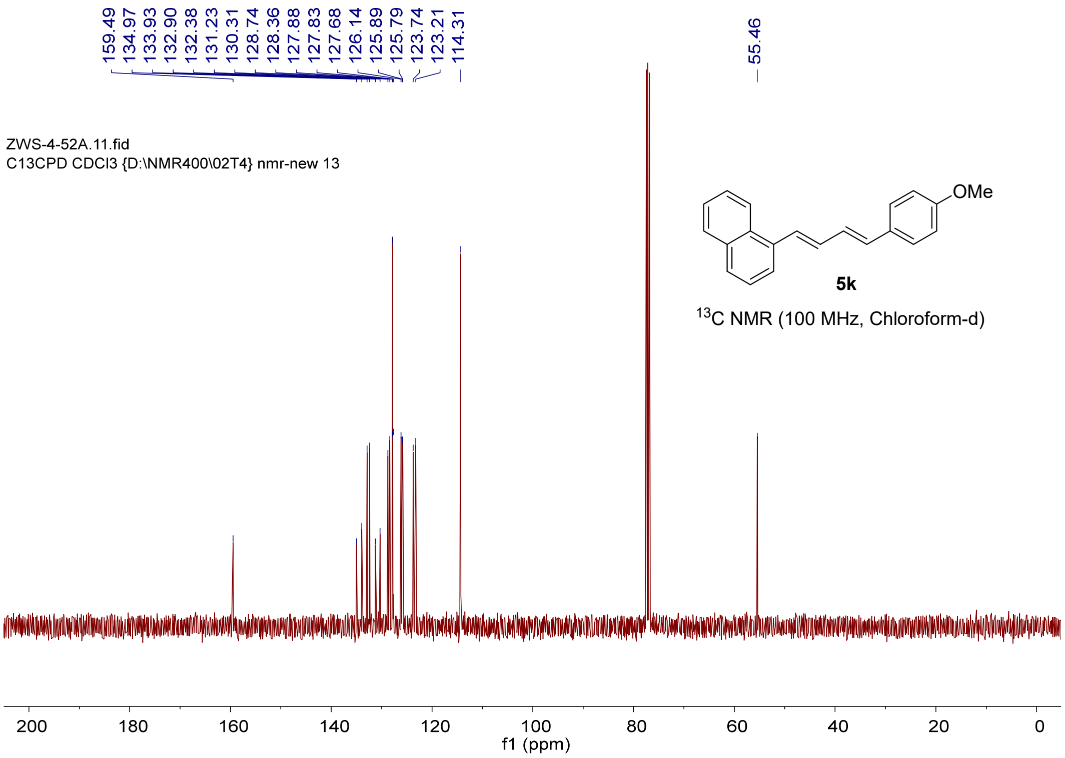

Supplementary Figure 39. ^{13}**C NMR** of compound **5k**

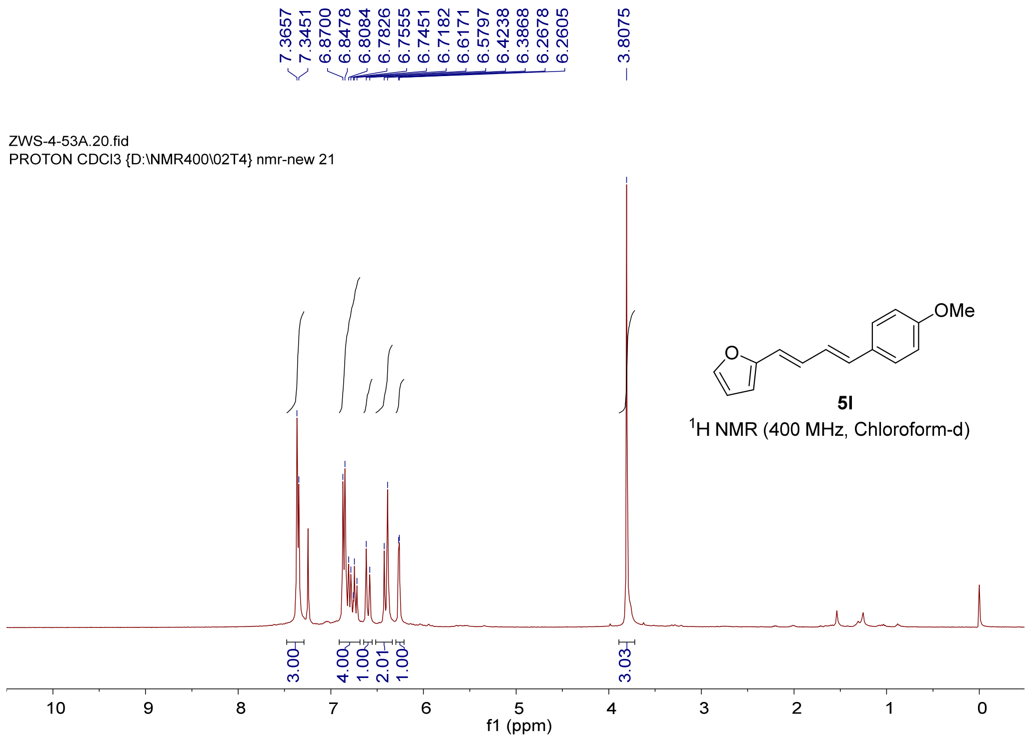

Supplementary Figure 40. ^{1}**H NMR** of compound **5l**

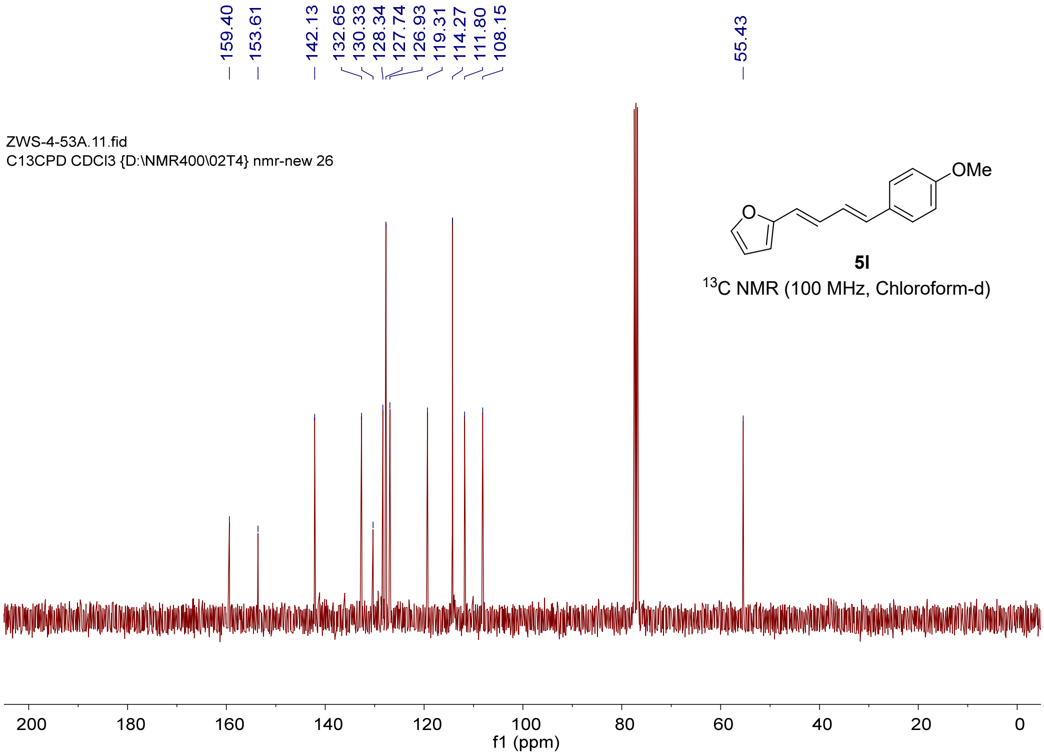

Supplementary Figure 41. ^{13}**C NMR** of compound **5l**

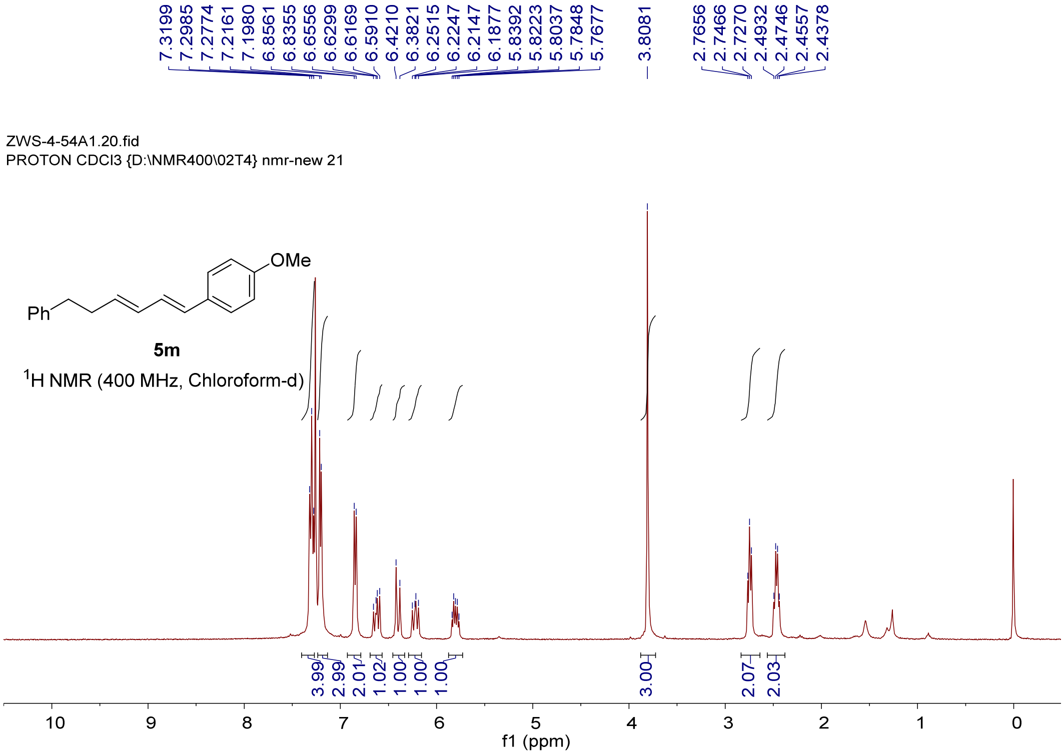

Supplementary Figure 42. ^{1}H NMR of compound **5m**

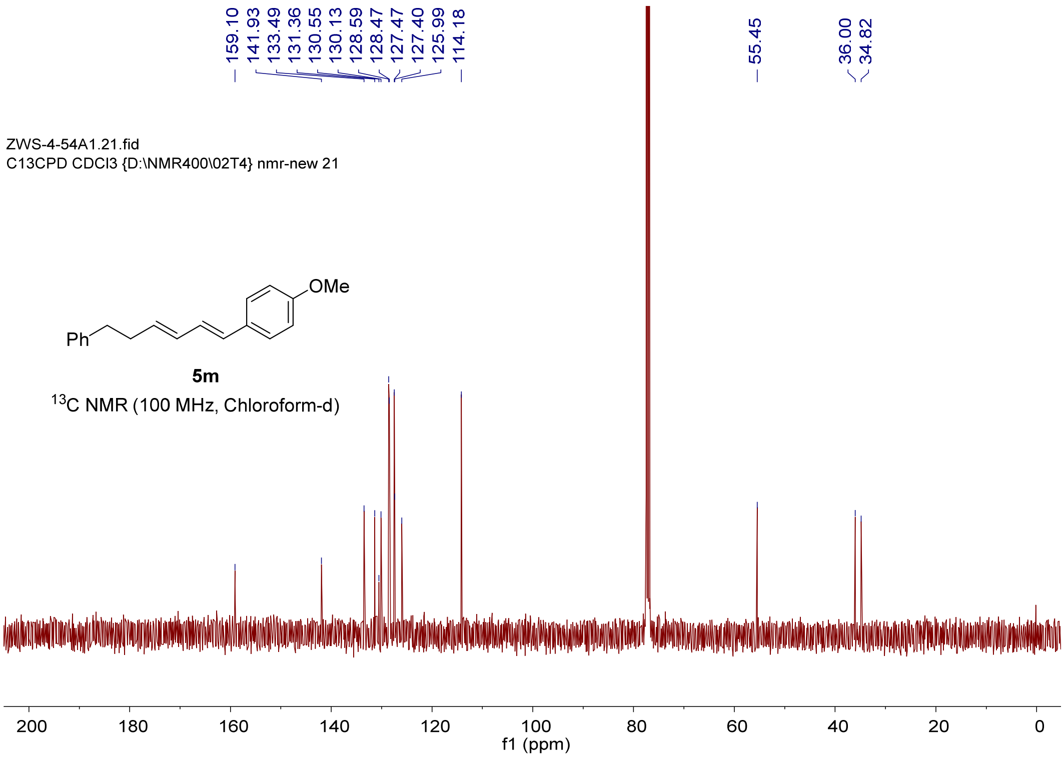

Supplementary Figure 43. ^{13}C NMR of compound **5m**

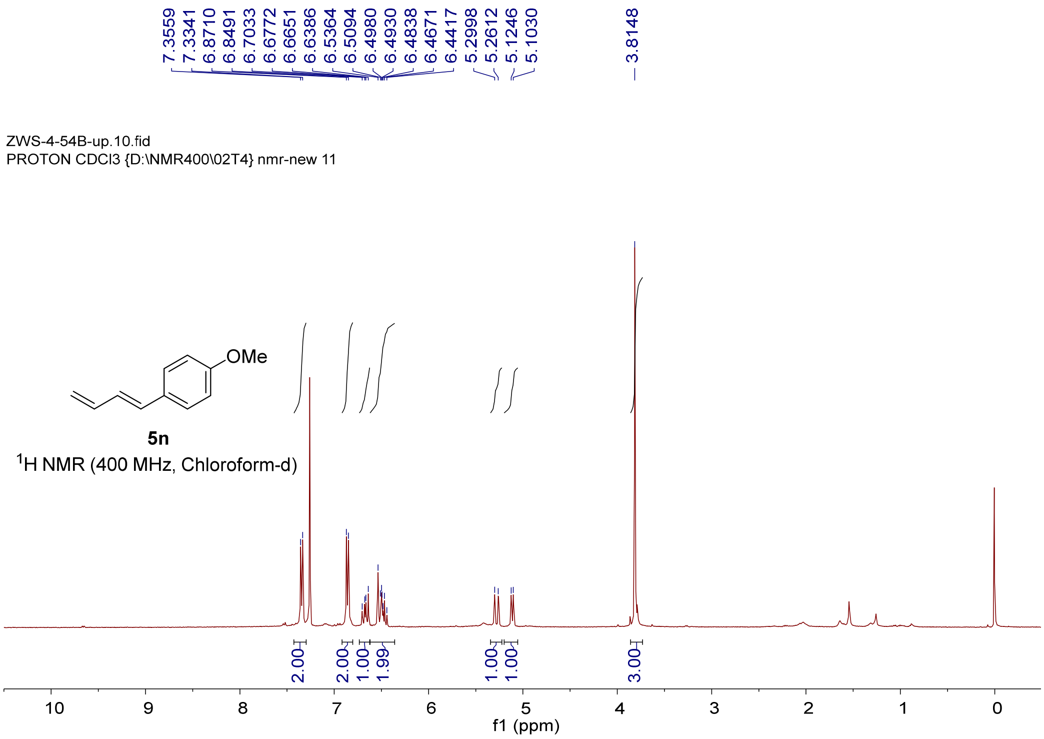

Supplementary Figure 44. **^{1}H NMR** of compound **5n**

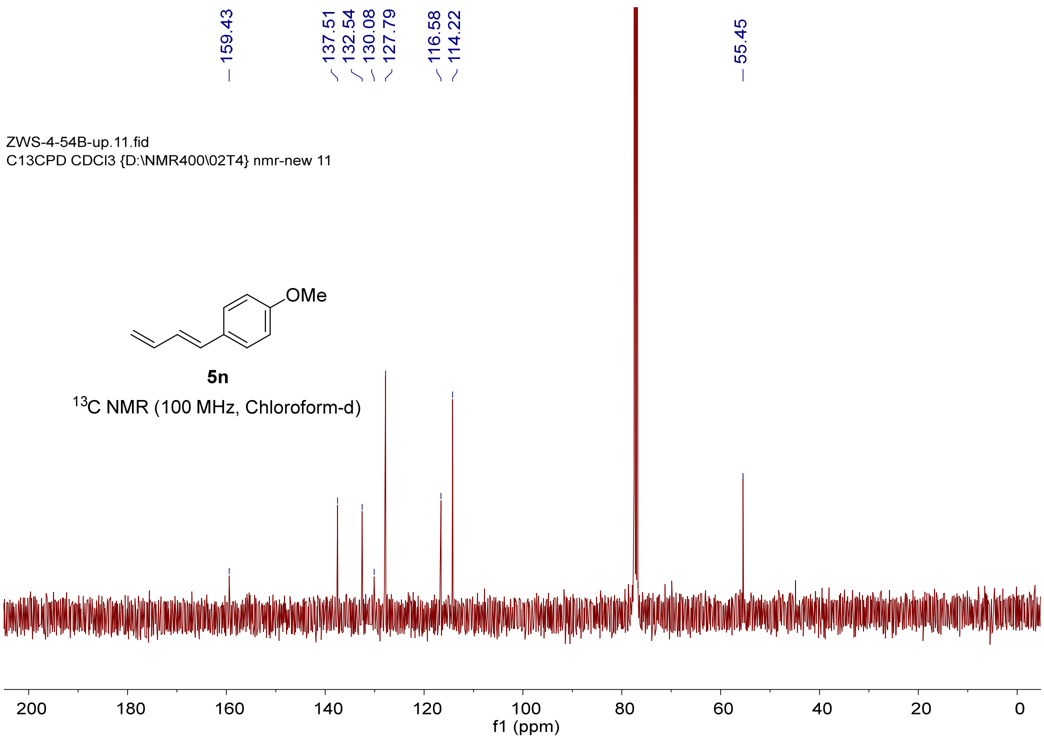

Supplementary Figure 45. **^{13}C NMR** of compound **5n**

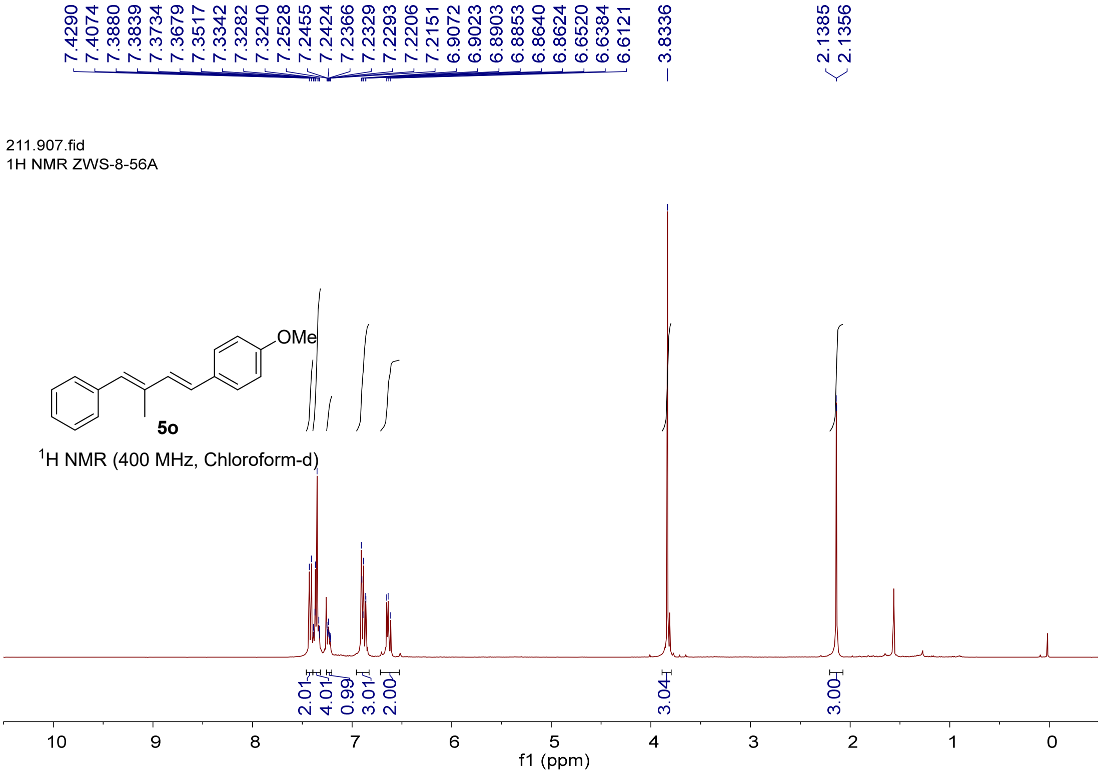

Supplementary Figure 46. **^{1}H NMR** of compound **5o**

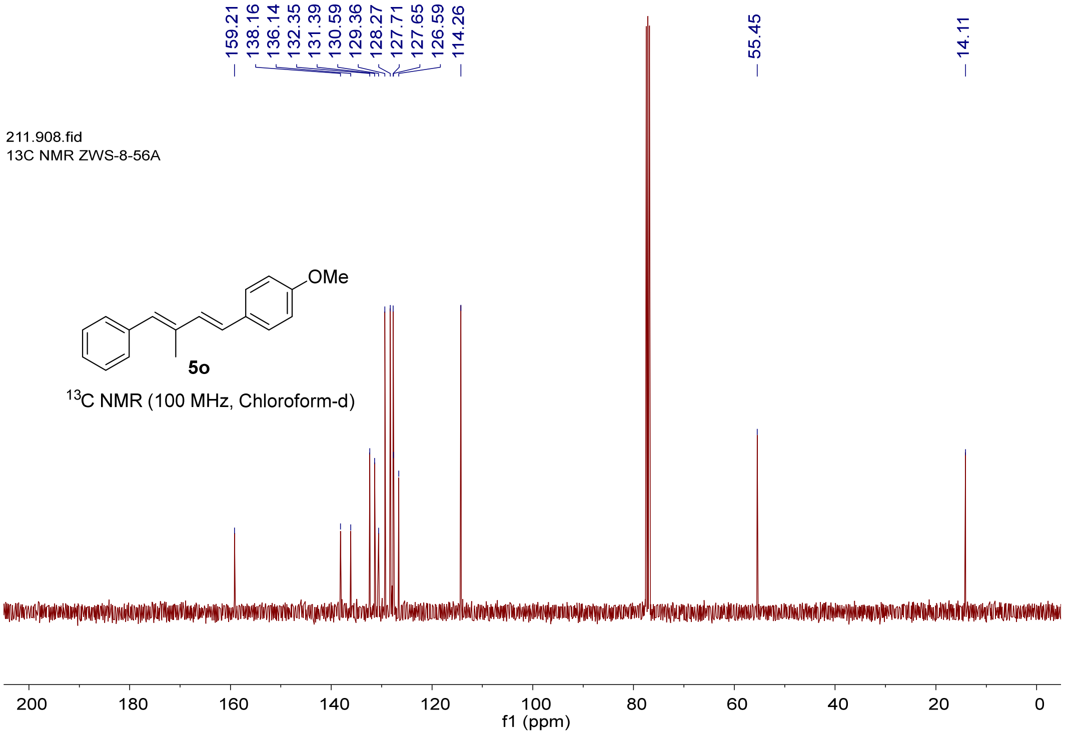

Supplementary Figure 47. **^{13}C NMR** of compound **5o**

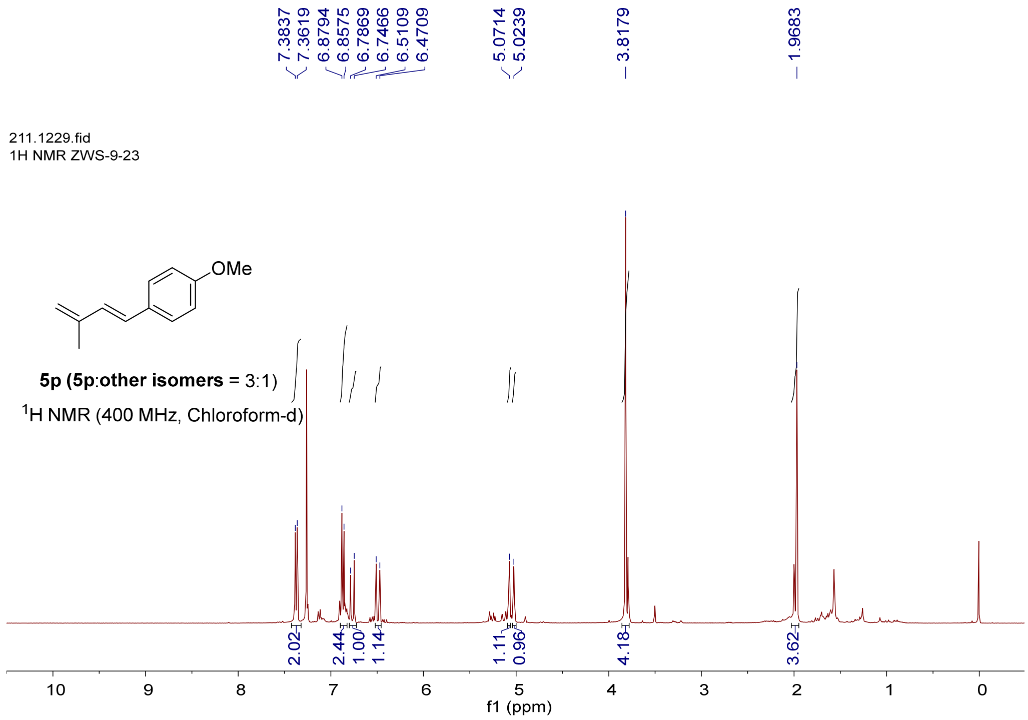

Supplementary Figure 48. ^{1}**H NMR** of compound **5p (5p:other isomers = 3:1)**

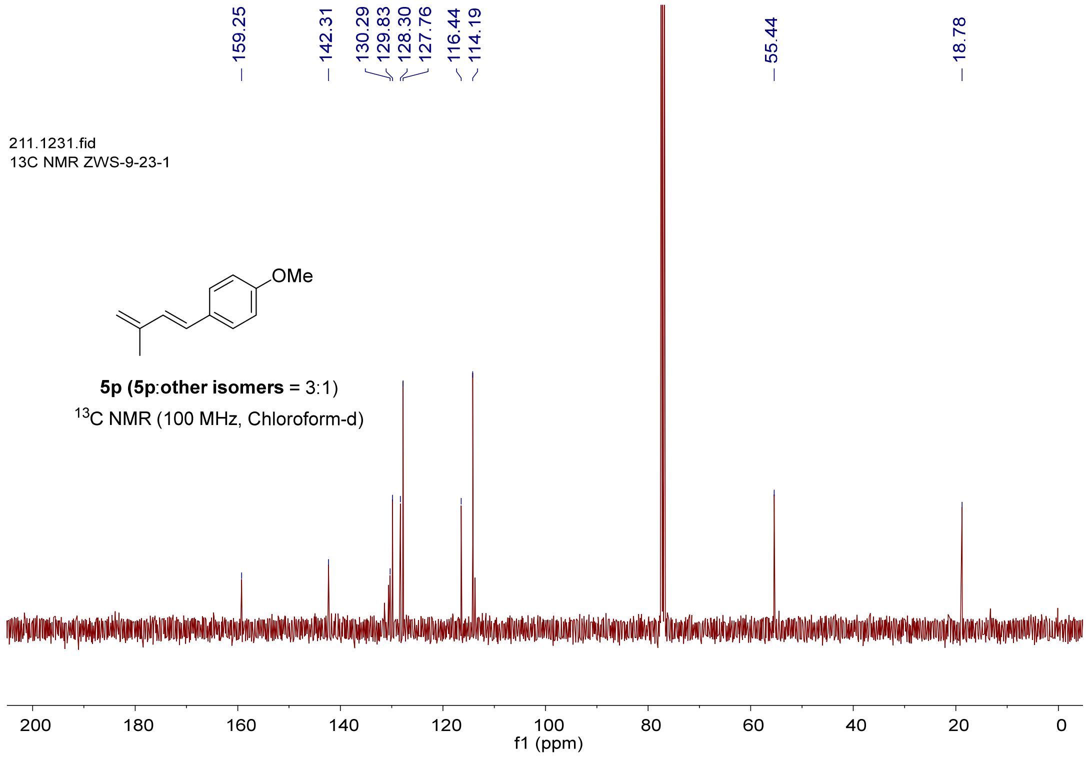

Supplementary Figure 49. ^{13}**C NMR** of compound **5p (5p:other isomers = 3:1)**

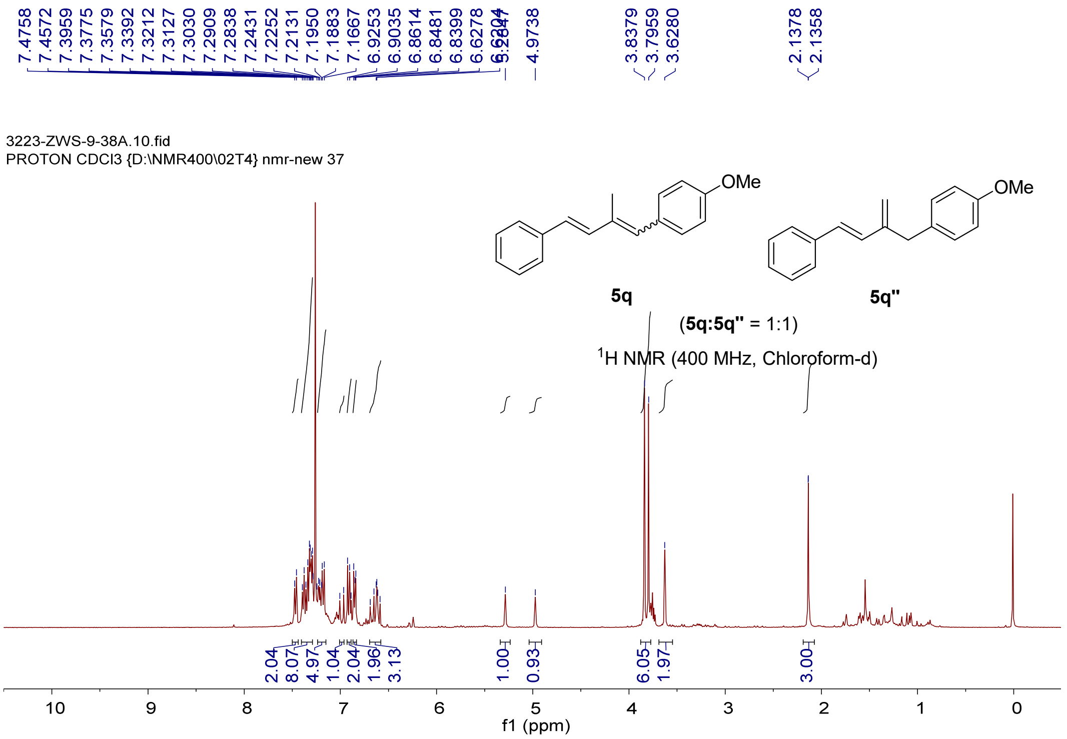

Supplementary Figure 50. **^{1}H NMR** of compound **5q** and **5q"** **(5q:5q" = 1:1)**

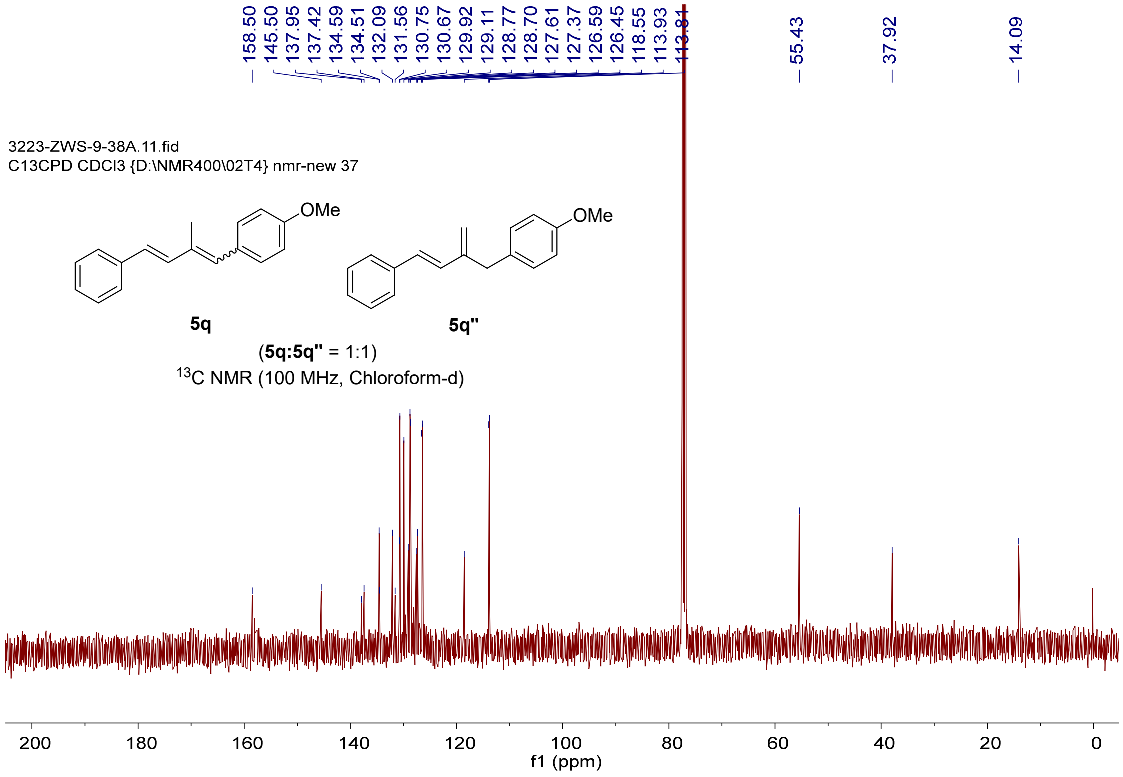

Supplementary Figure 51. **^{13}C NMR** of compound **5q** and **5q"** **(5q:5q" = 1:1)**

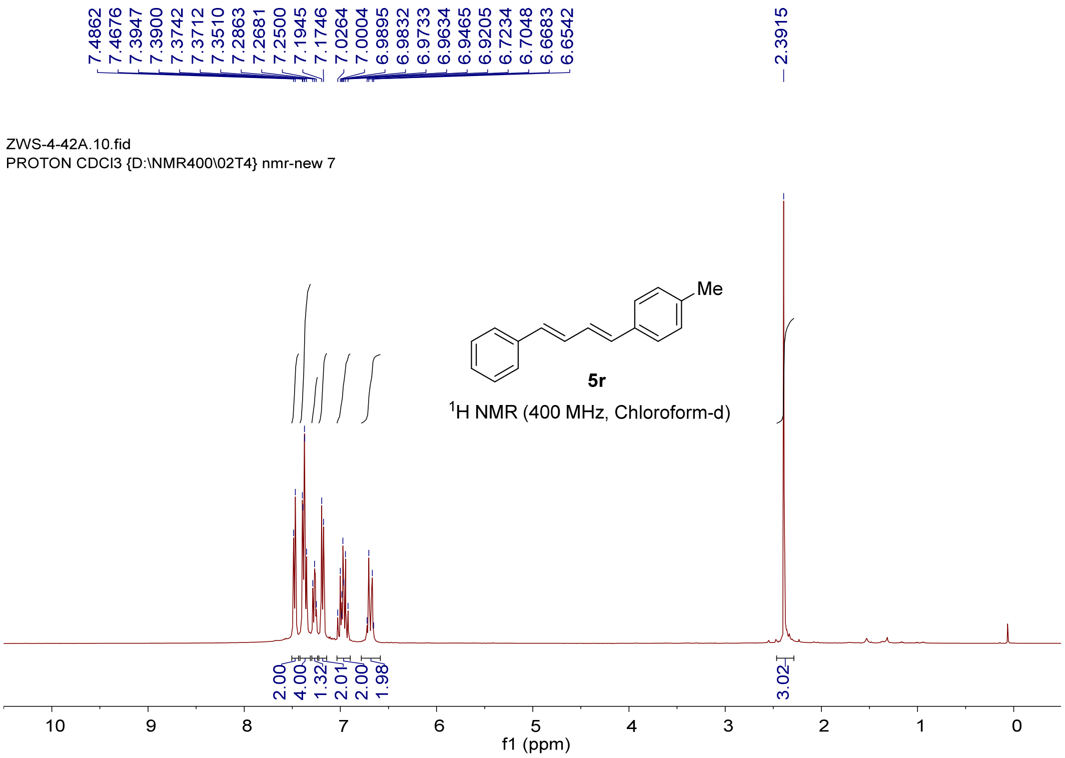

Supplementary Figure 52. ^{1}**H NMR** of compound **5r**

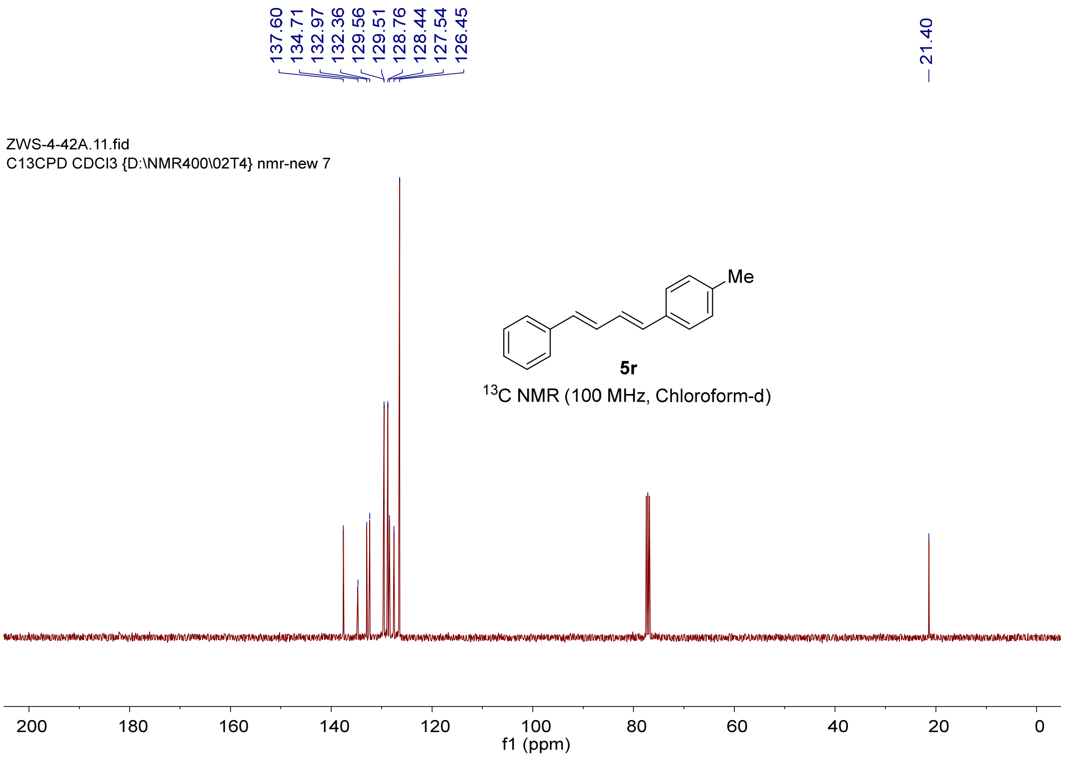

Supplementary Figure 53. ^{13}**C NMR** of compound **5r**

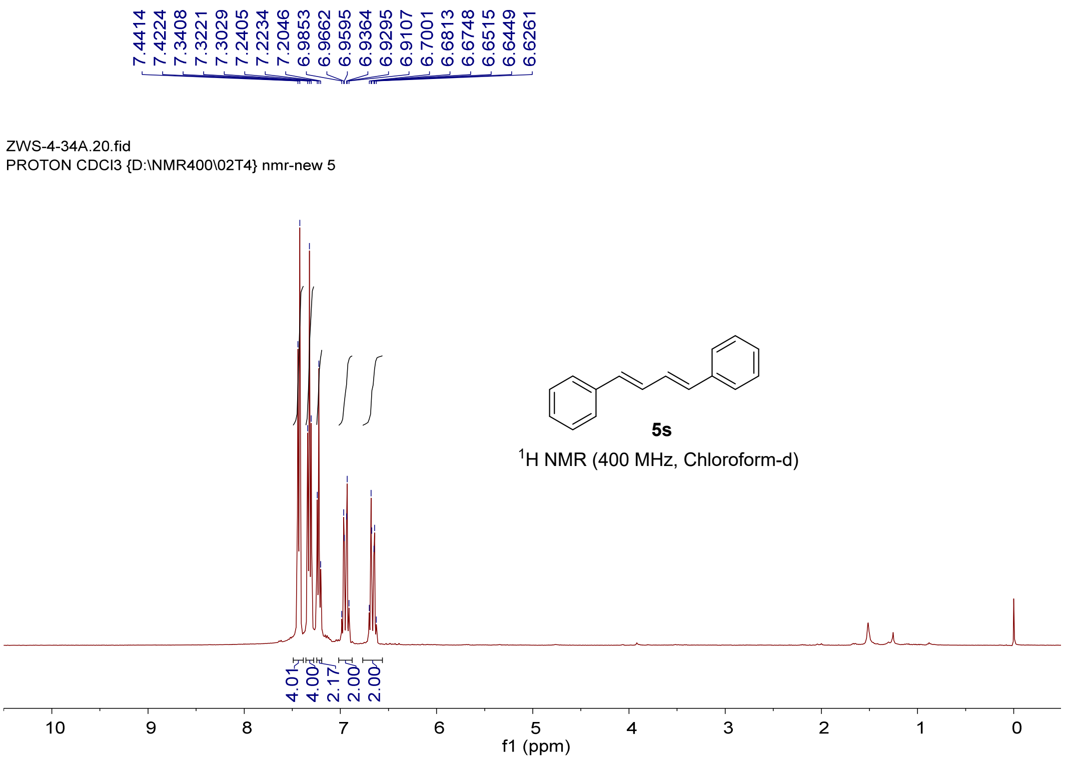

Supplementary Figure 54. ^{1}H NMR of compound **5s**

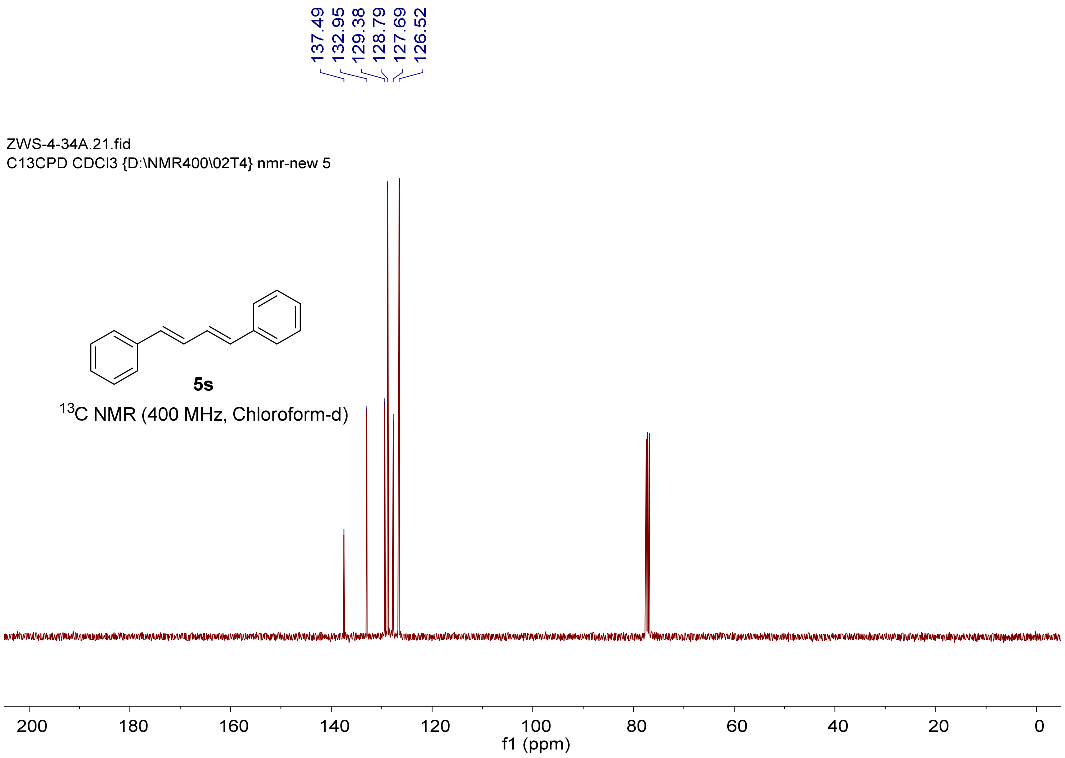

Supplementary Figure 55. ^{13}C NMR of compound **5s**

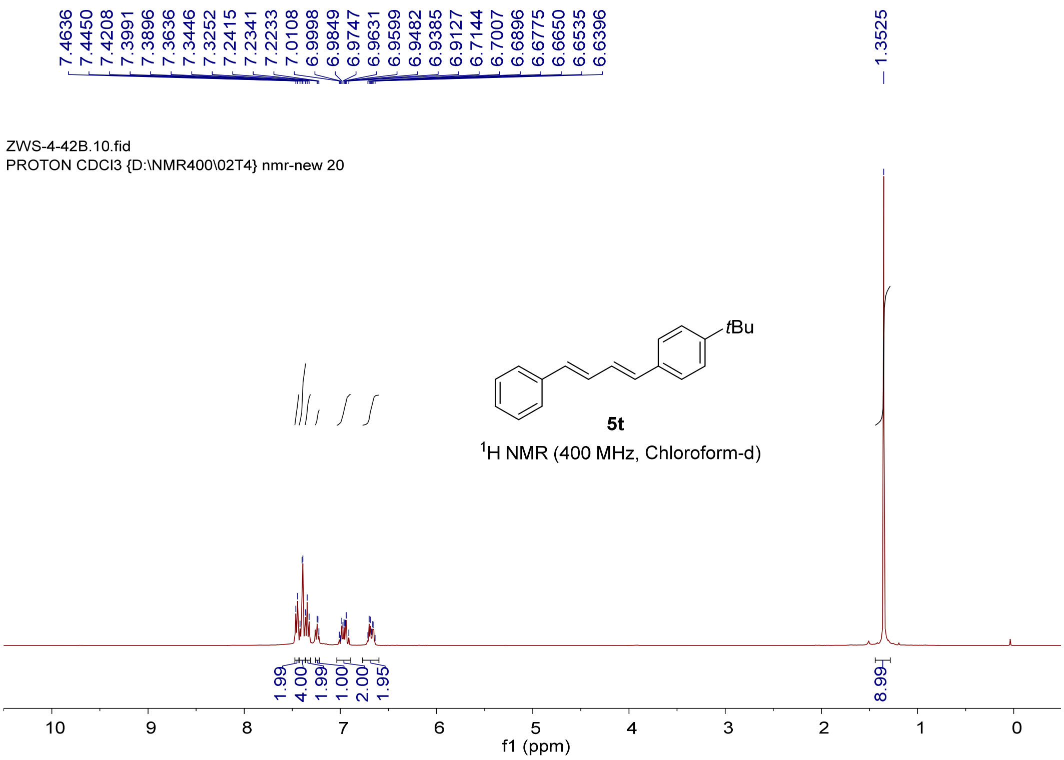

Supplementary Figure 56. ^{1}**H NMR** of compound **5t**

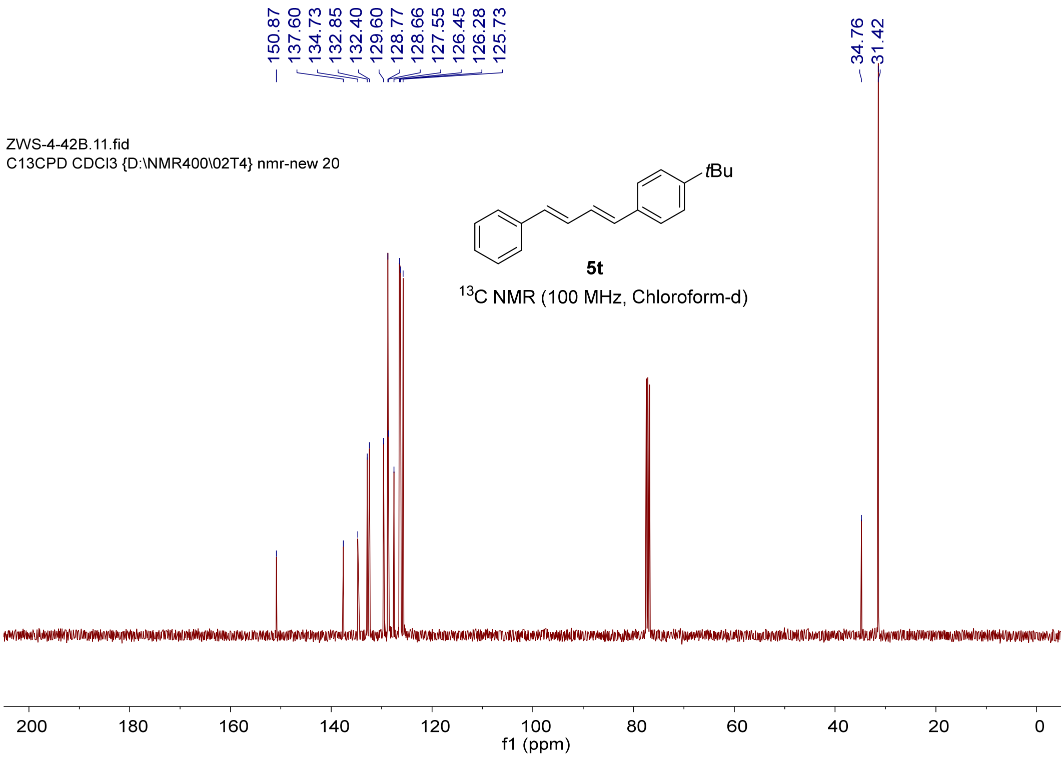

Supplementary Figure 57. ^{13}**C NMR** of compound **5t**

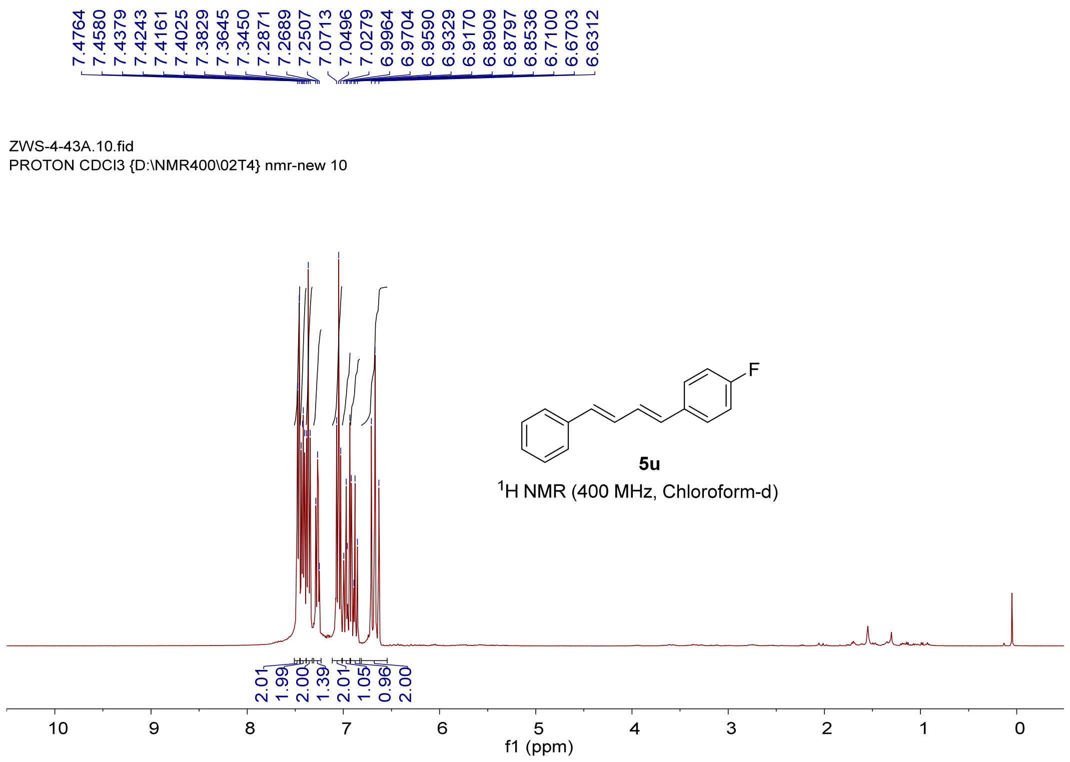

Supplementary Figure 58. ^{1}**H NMR** of compound **5u**

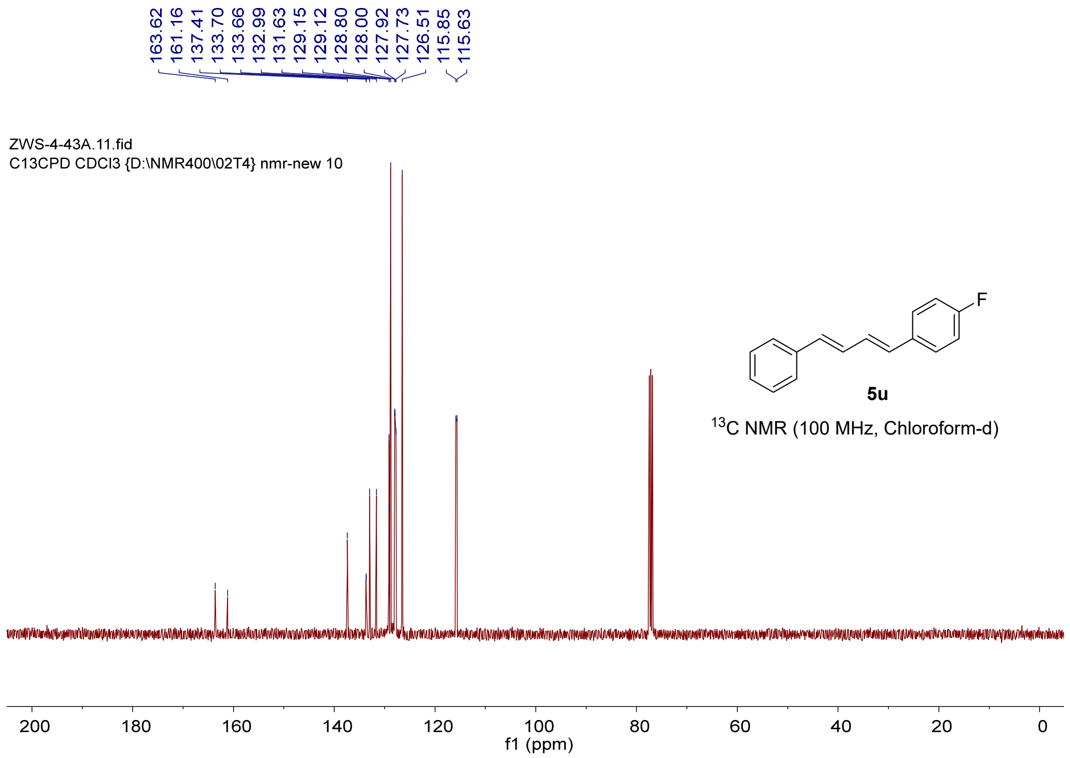

Supplementary Figure 59. ^{13}**C NMR** of compound **5u**

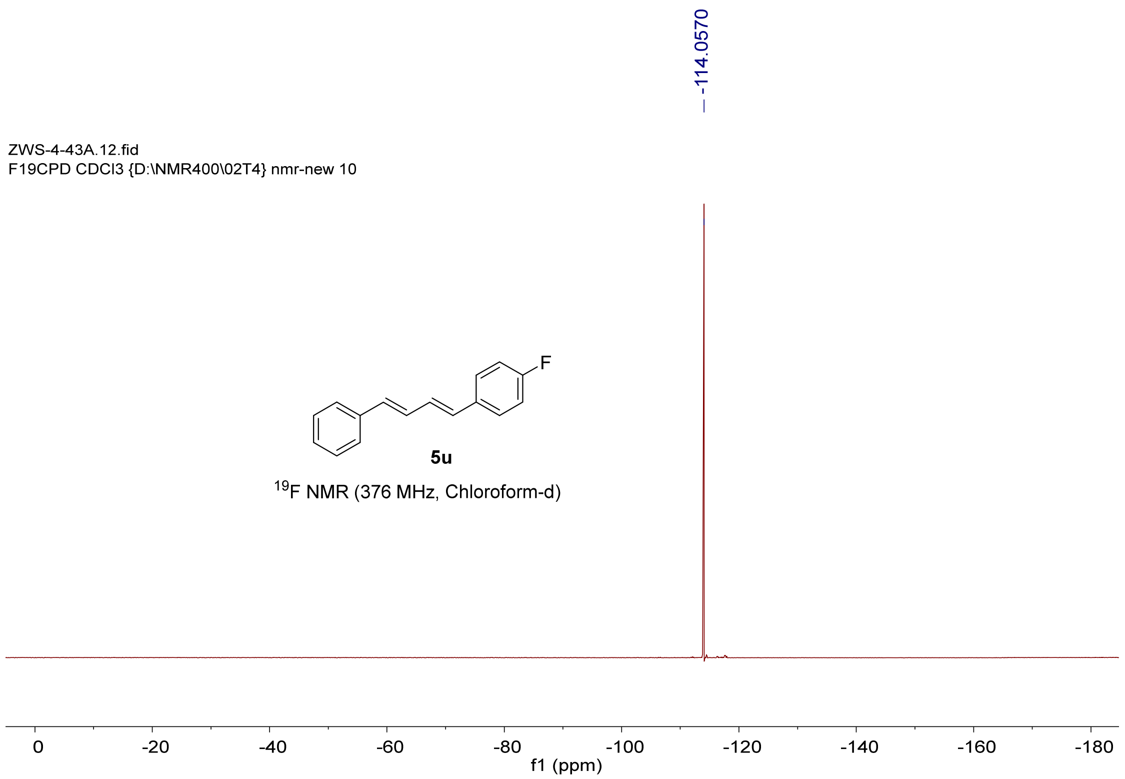

Supplementary Figure 60. ^{19}F NMR of compound **5u**

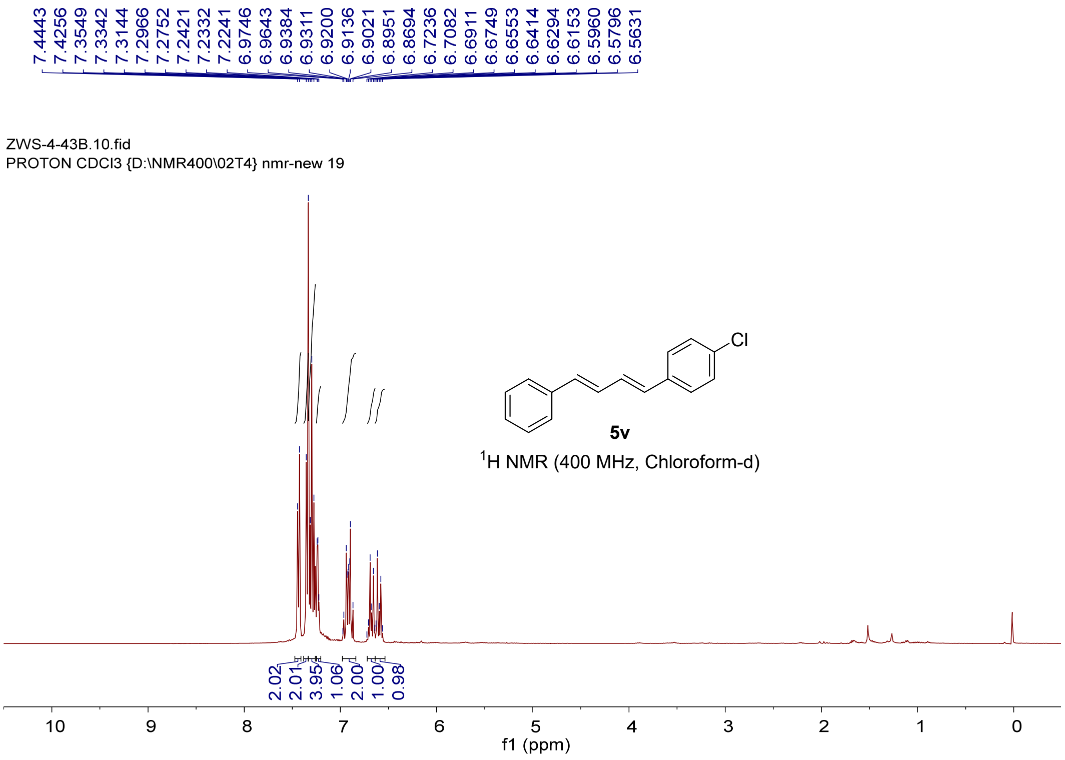

Supplementary Figure 61. ^{1}H NMR of compound **5v**

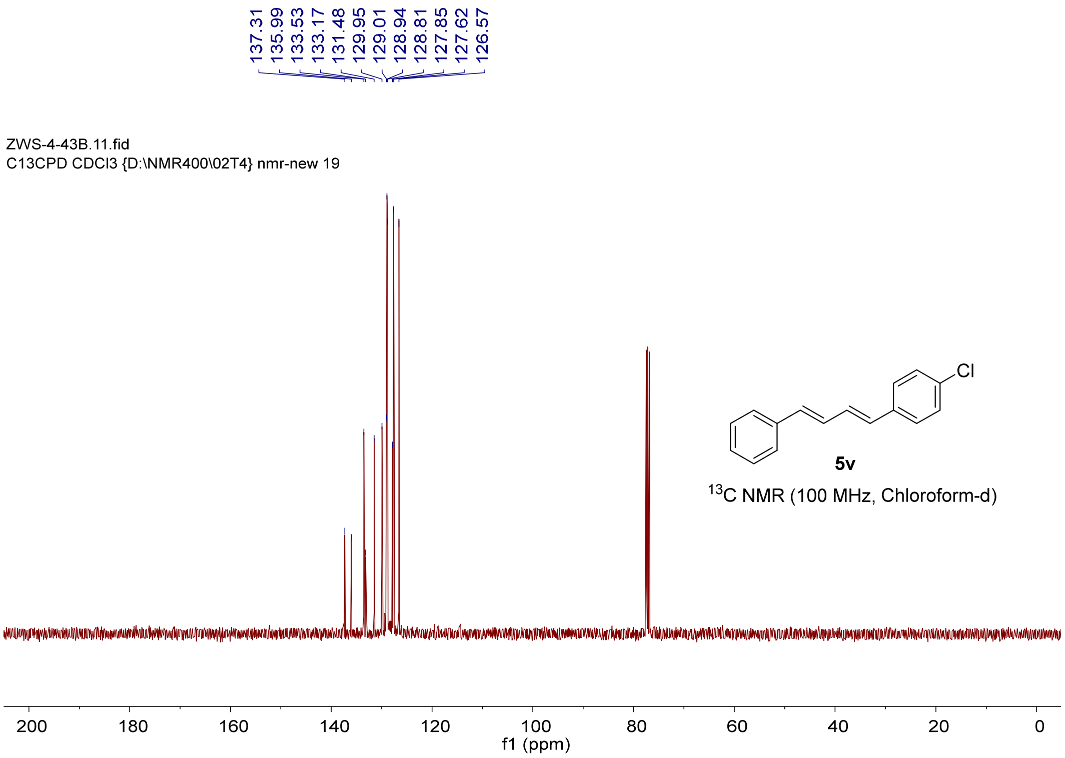

Supplementary Figure 62. ^{13}C NMR of compound **5v**

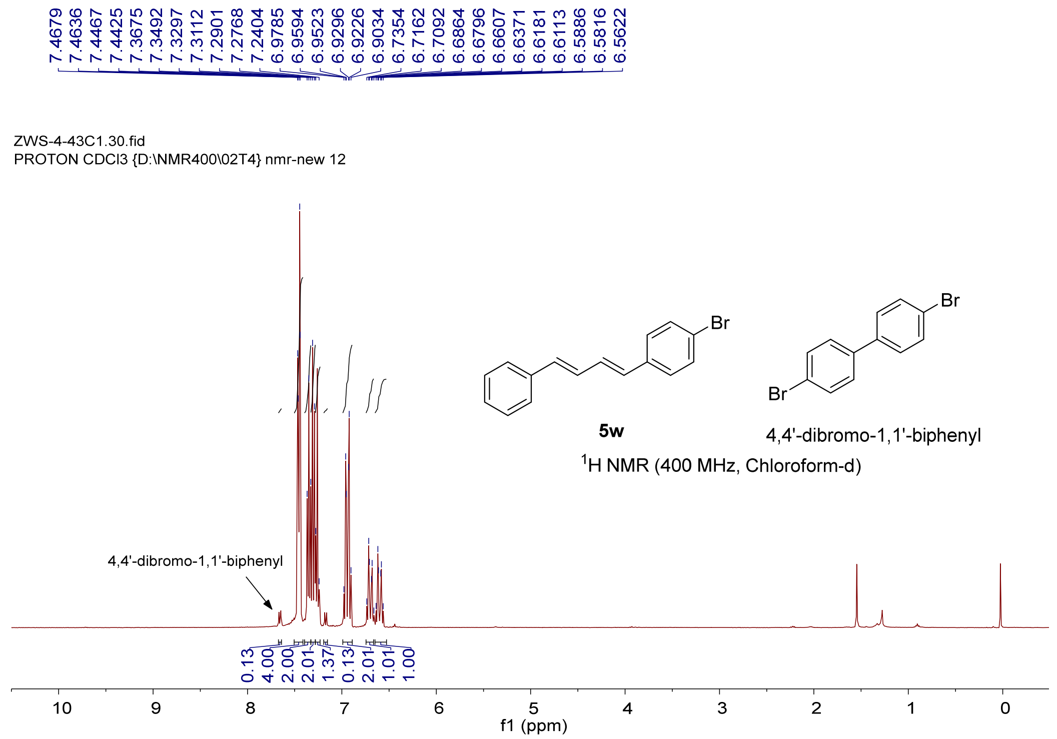

Supplementary Figure 63. ^{1}H NMR of compound **5w**

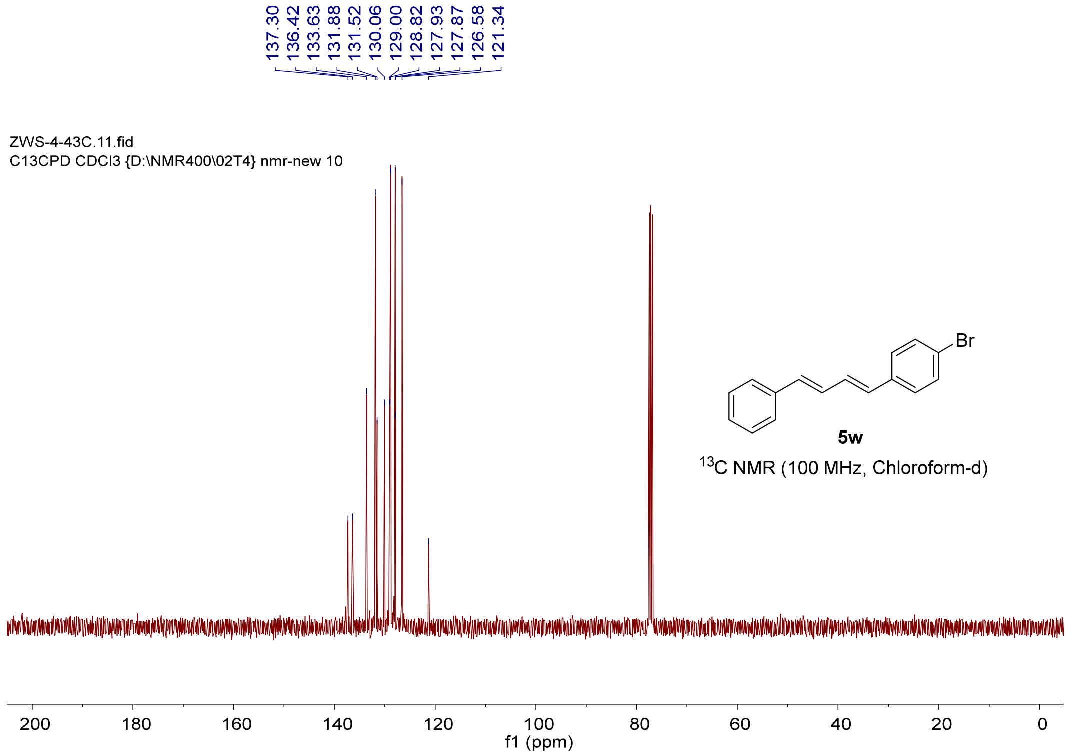

Supplementary Figure 64. ^{13}C NMR of compound **5w**

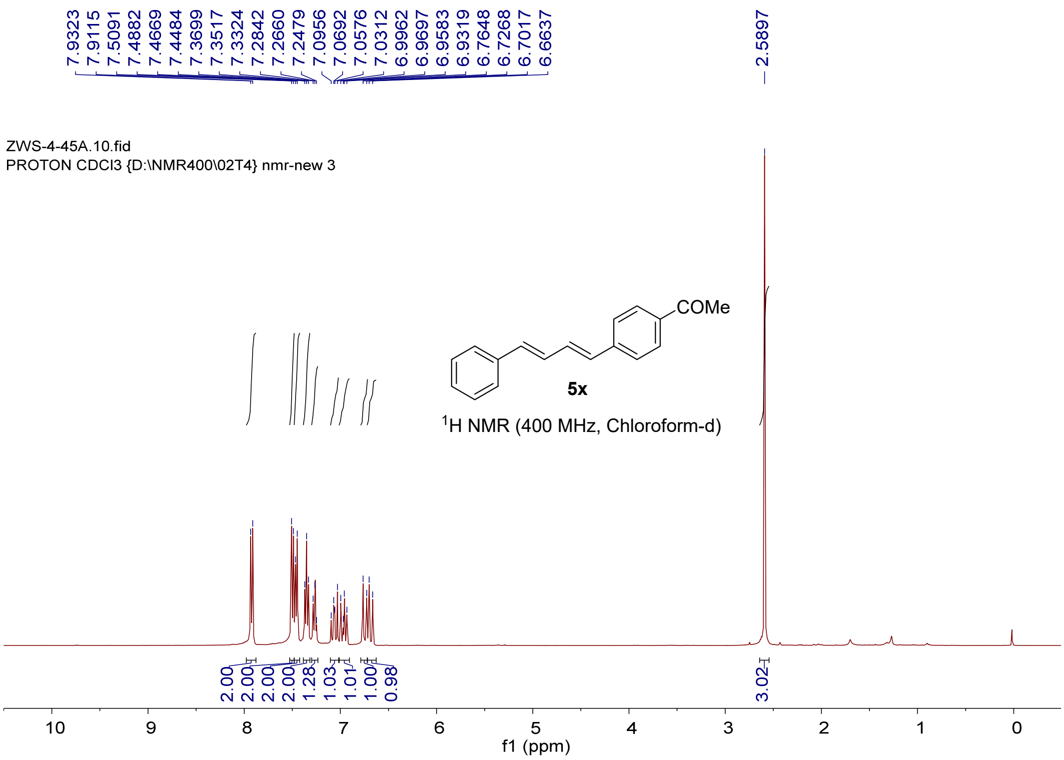

Supplementary Figure 65. ^{1}H NMR of compound **5x**

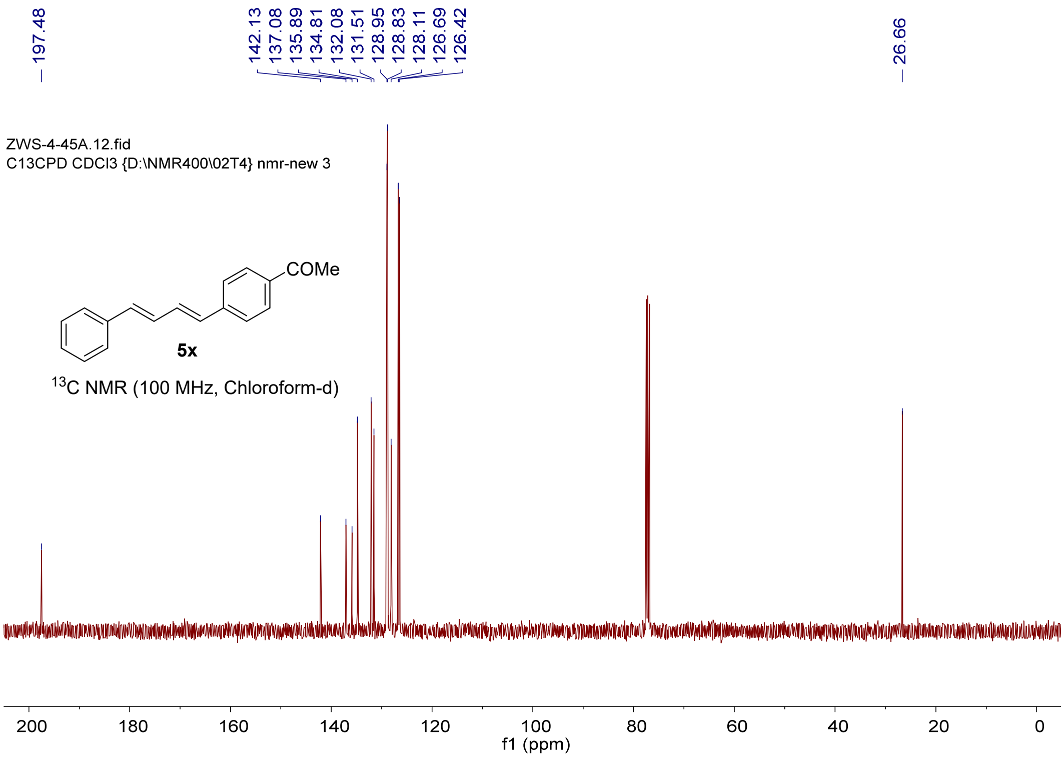

Supplementary Figure 66. ^{13}C NMR of compound **5x**

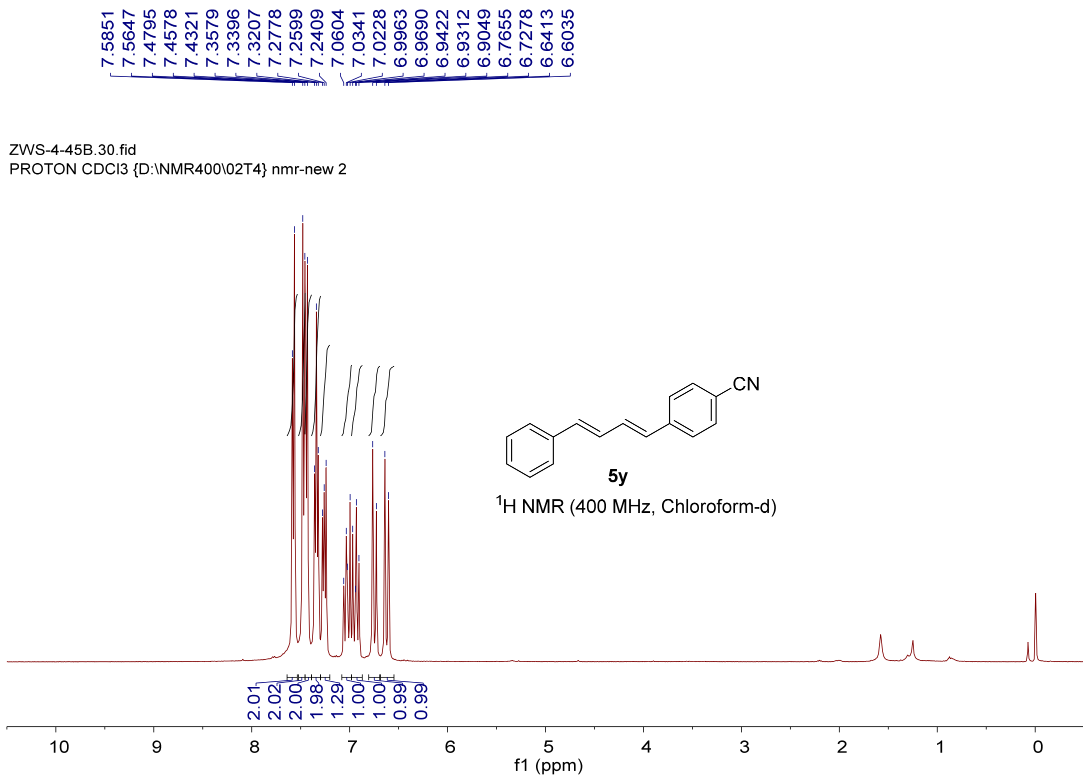

Supplementary Figure 67. ^{1}**H NMR** of compound **5y**

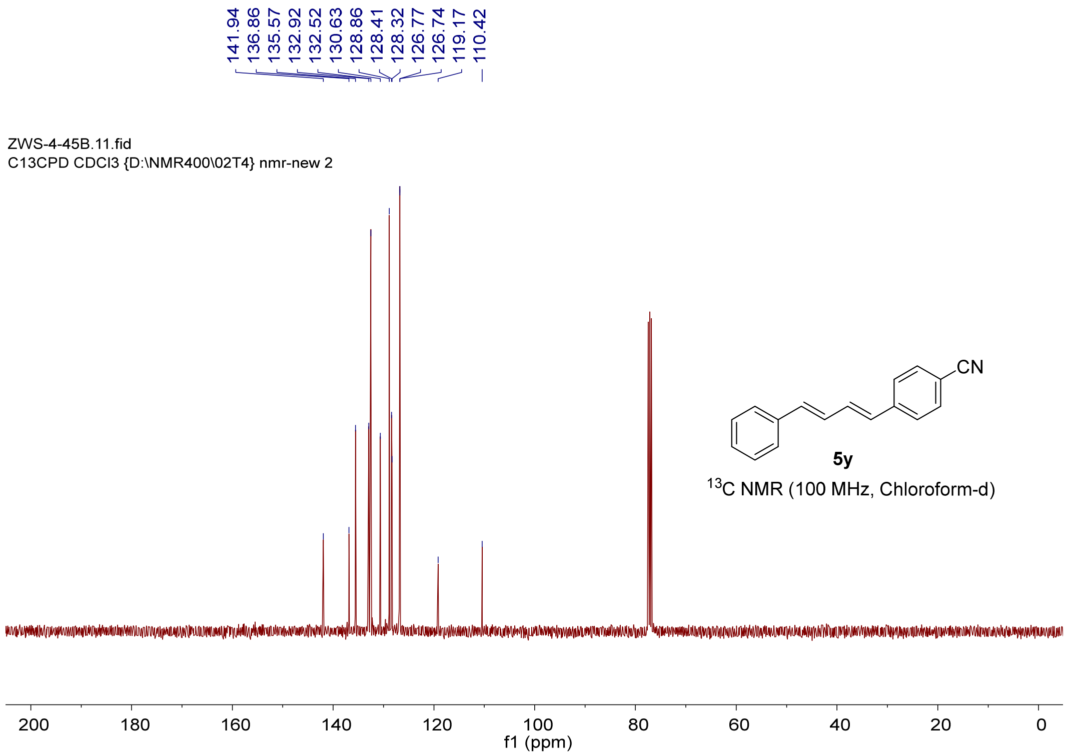

Supplementary Figure 68. ^{13}**C NMR** of compound **5y**

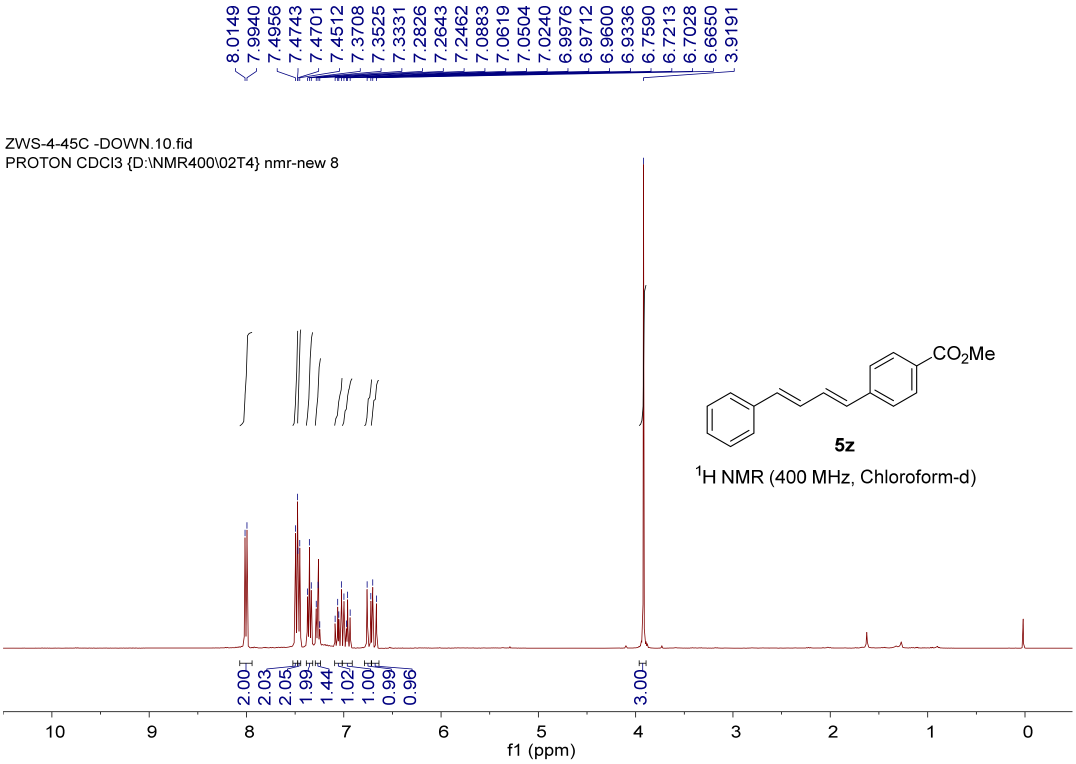

Supplementary Figure 69. **^{1}H NMR** of compound **5z**

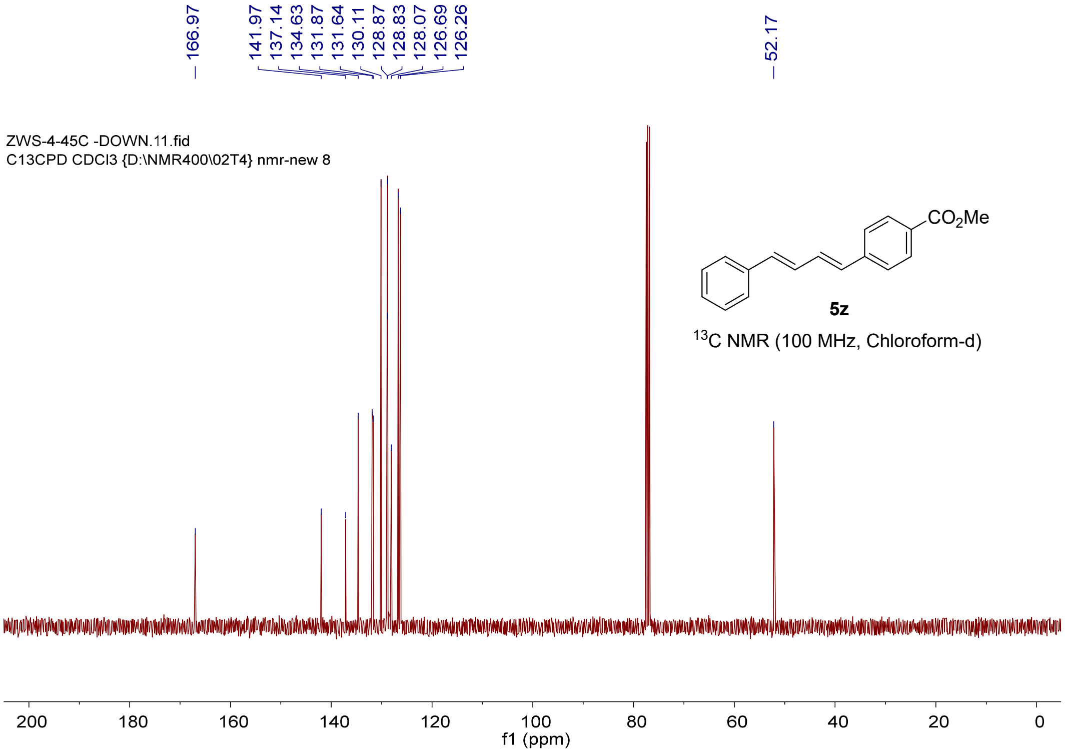

Supplementary Figure 70. **^{13}C NMR** of compound **5z**

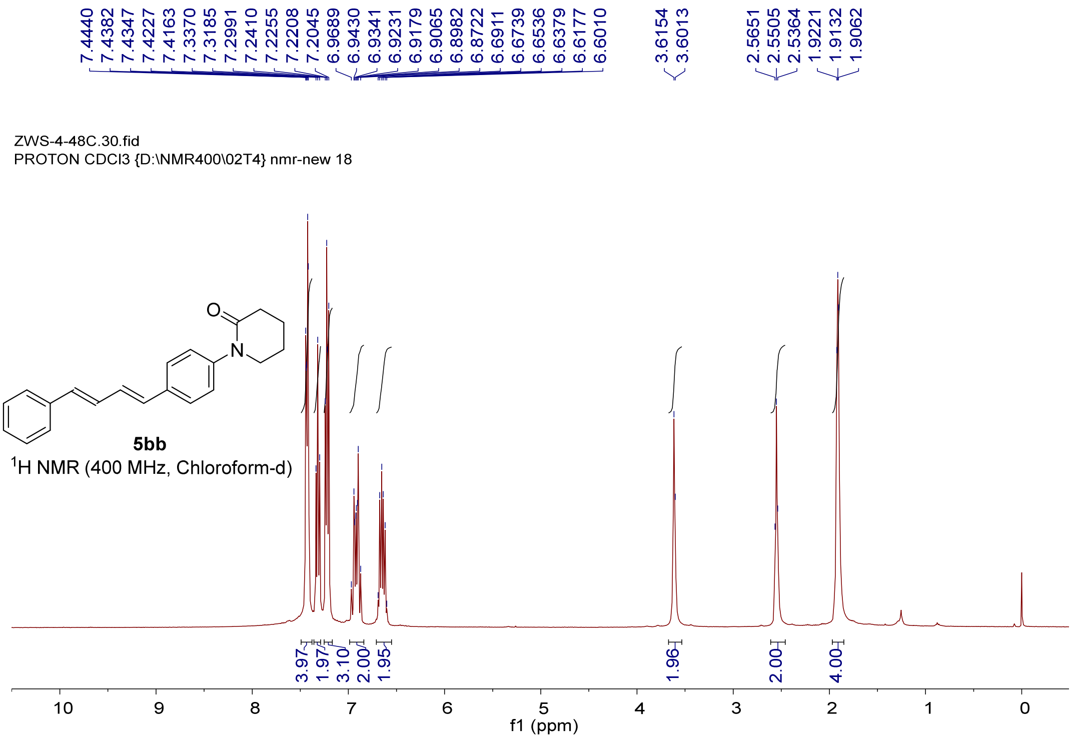

Supplementary Figure 71. ^{1}**H NMR** of compound **5bb**

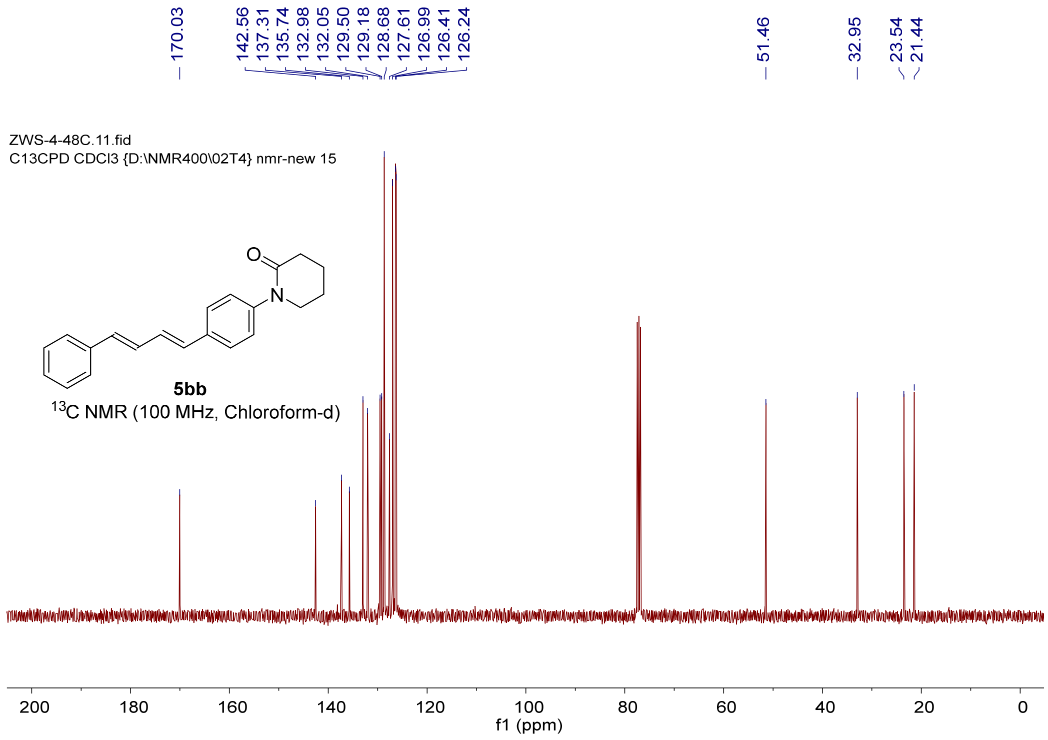

Supplementary Figure 72. ^{13}**C NMR** of compound **5bb**

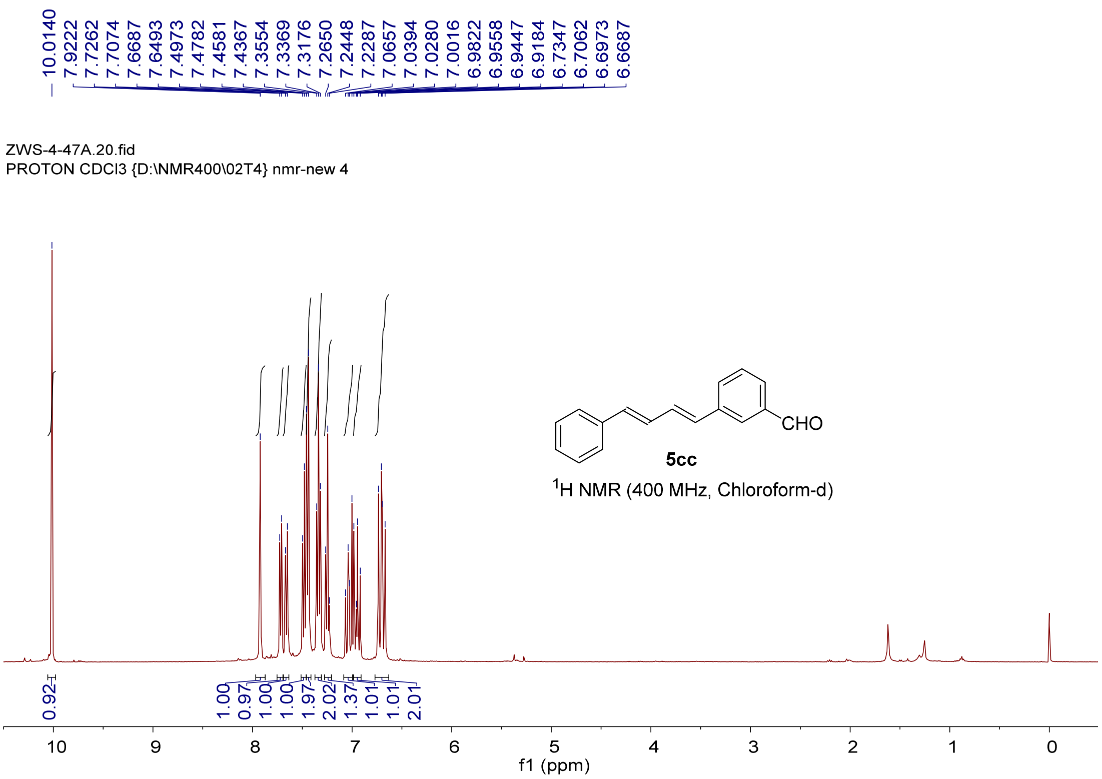

Supplementary Figure 73. ^{1}H NMR of compound **5cc**

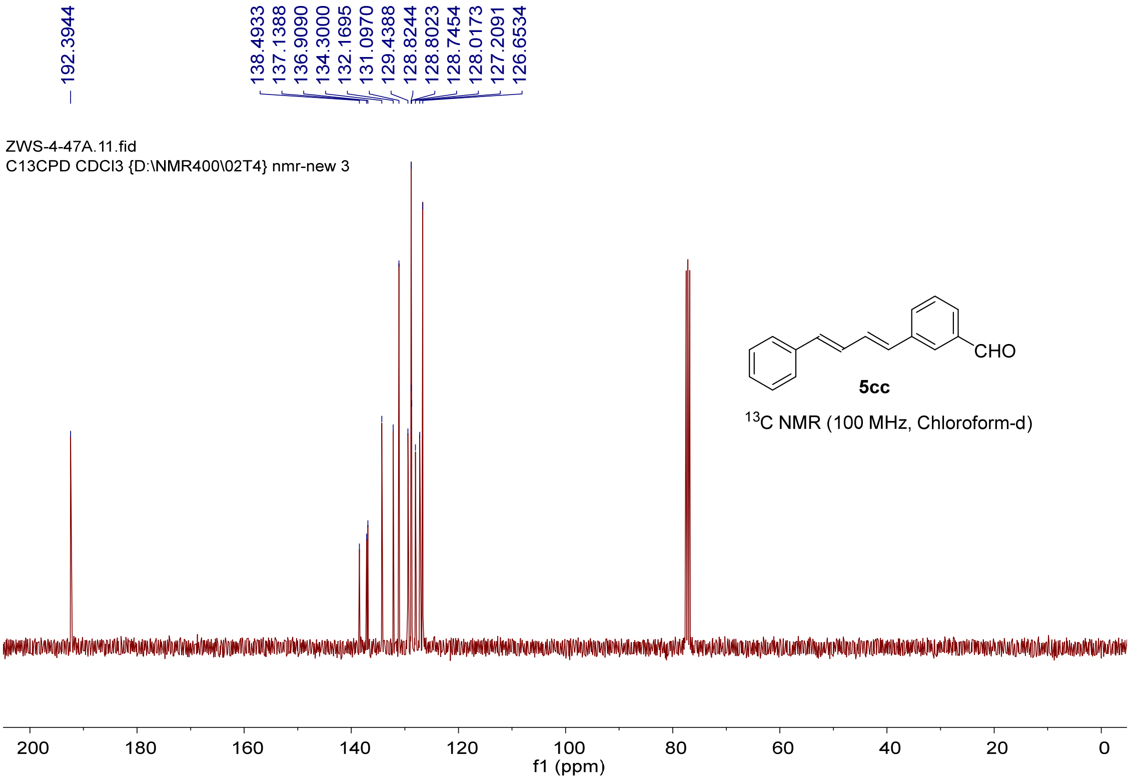

Supplementary Figure 74. ^{13}C NMR of compound **5cc**

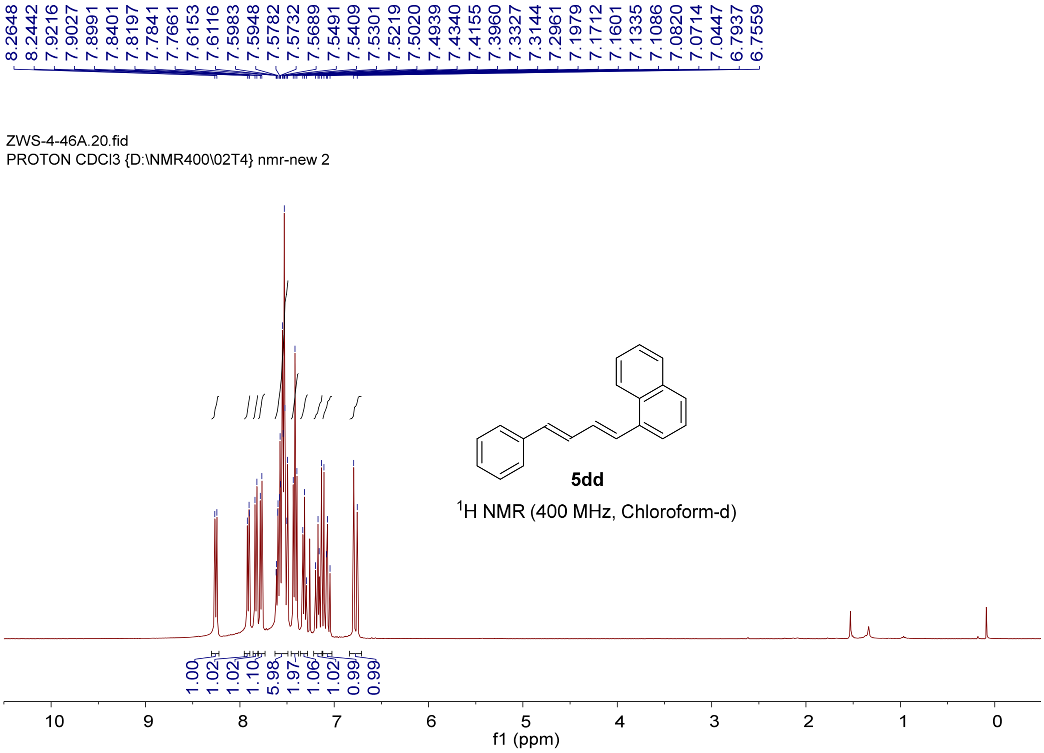

Supplementary Figure 75. ^{1}**H NMR** of compound **5dd**

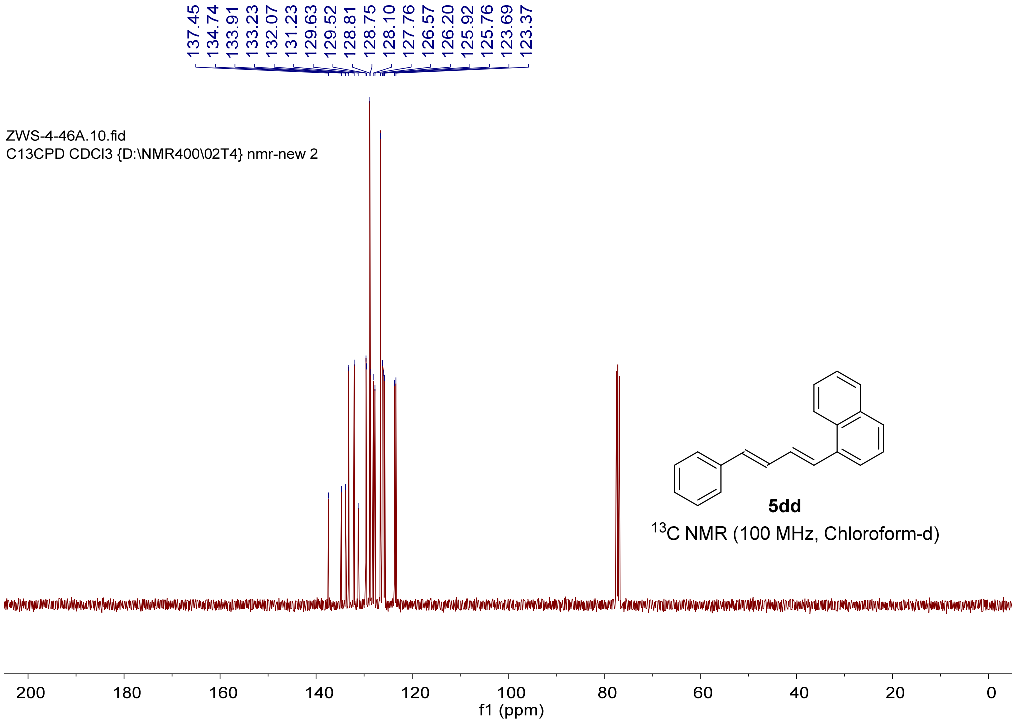

Supplementary Figure 76. ^{13}**C NMR** of compound **5dd**

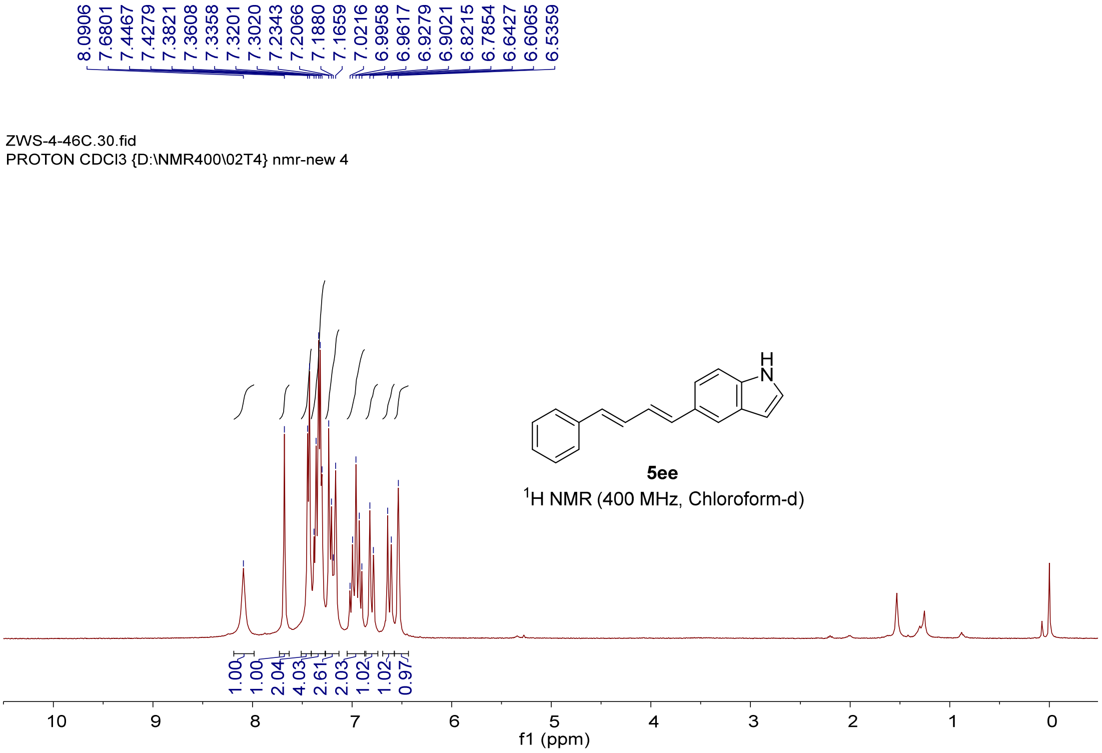

Supplementary Figure 77. ^{1}H NMR of compound **5ee**

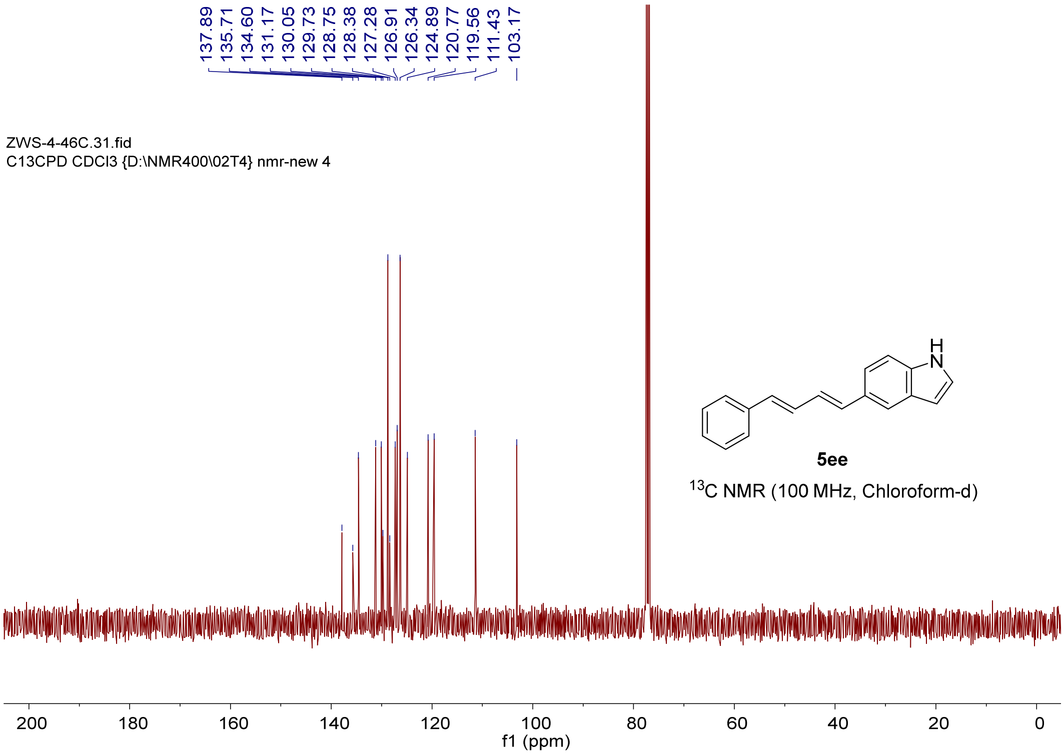

Supplementary Figure 78. ^{13}C NMR of compound **5ee**

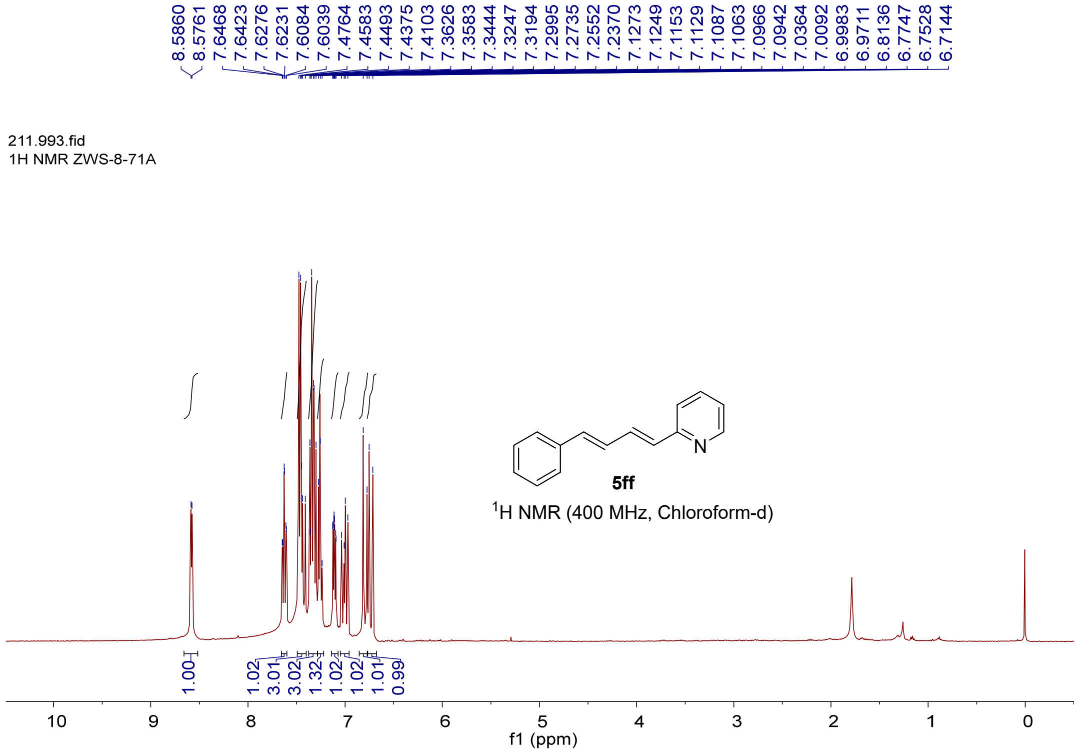

Supplementary Figure 79. **^{1}H NMR** of compound **5ff**

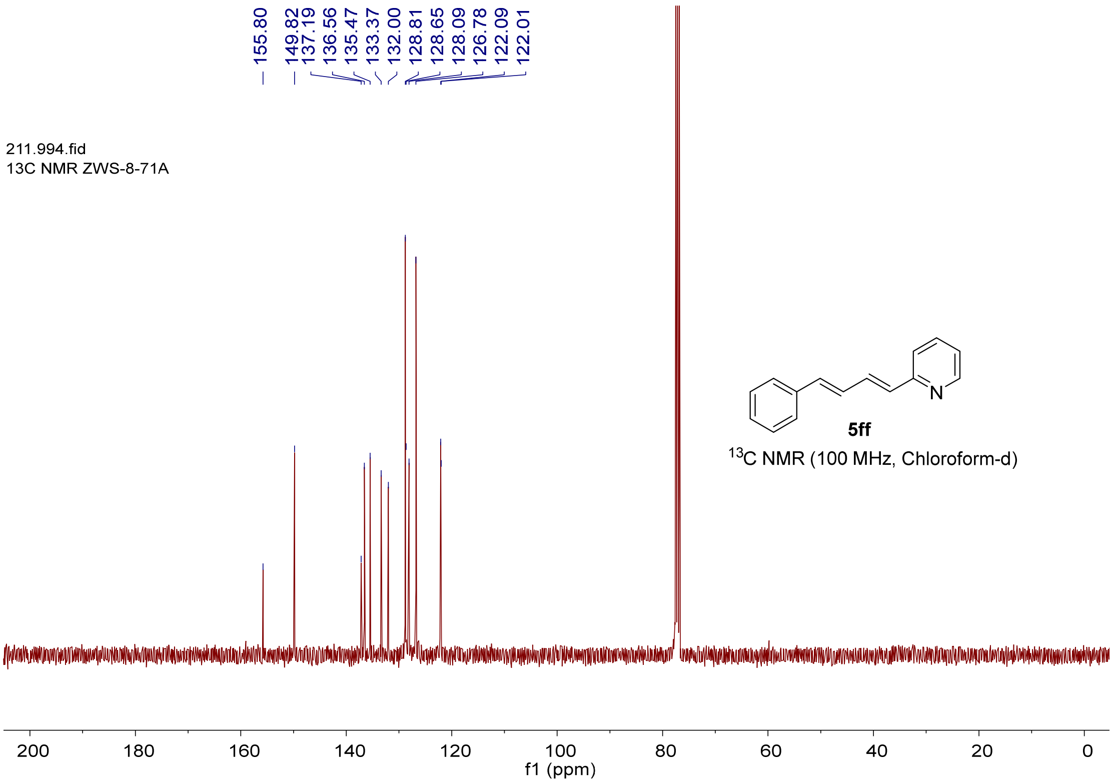

Supplementary Figure 80. **^{13}C NMR** of compound **5ff**

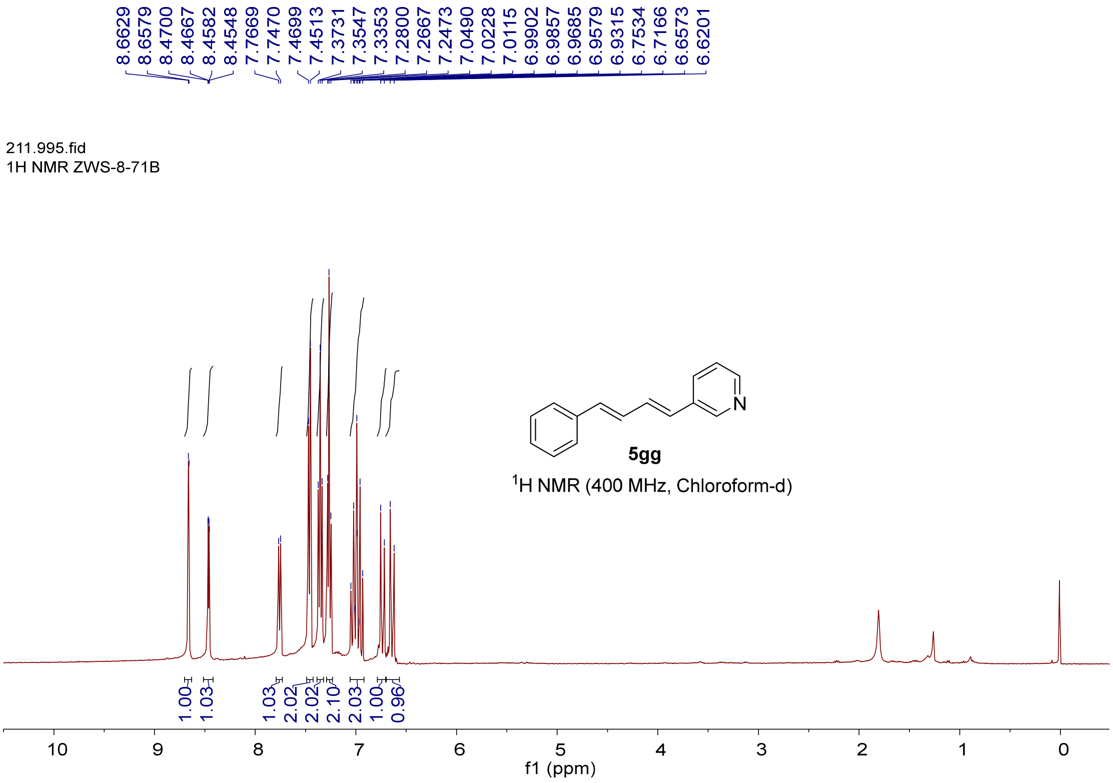

Supplementary Figure 81. **^{1}H NMR** of compound **5gg**

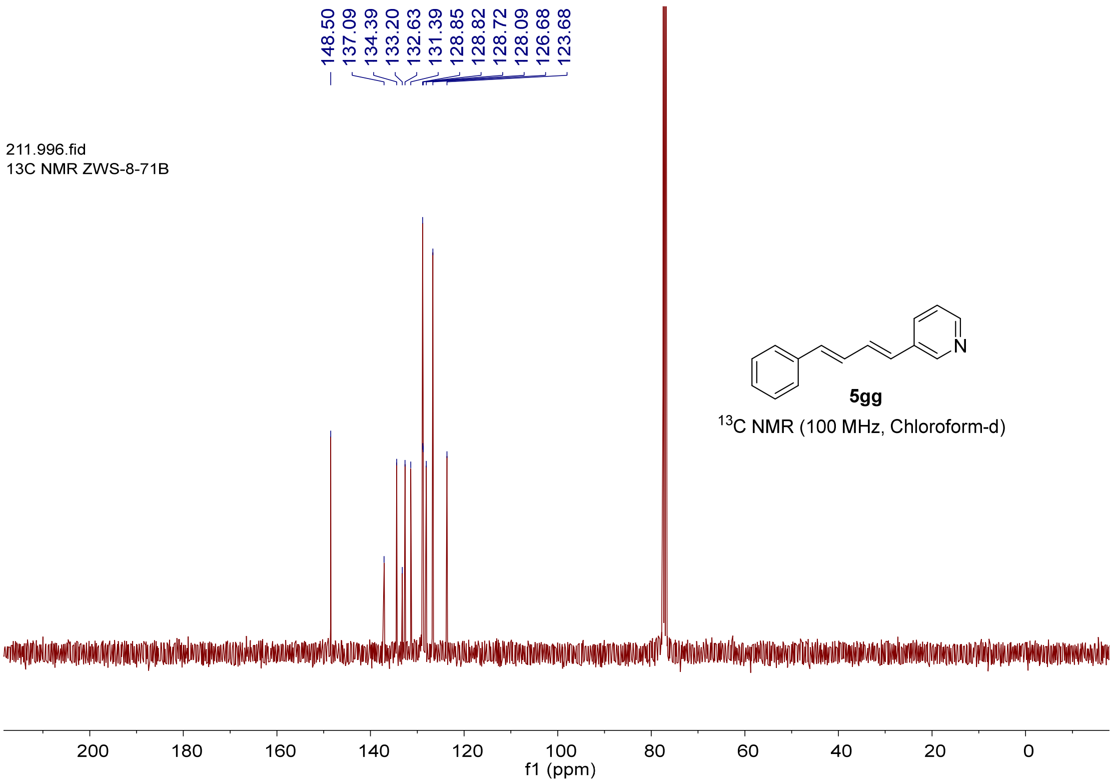

Supplementary Figure 82. **^{13}C NMR** of compound **5gg**

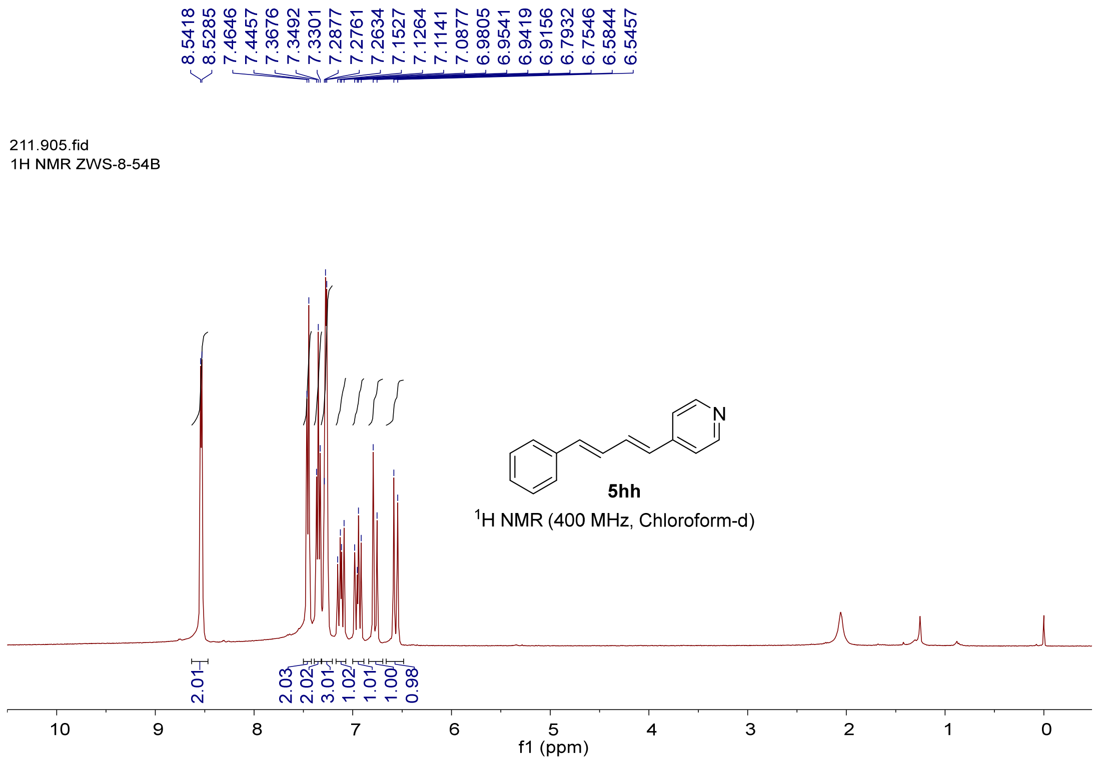

Supplementary Figure 83. **^{1}H NMR** of compound **5hh**

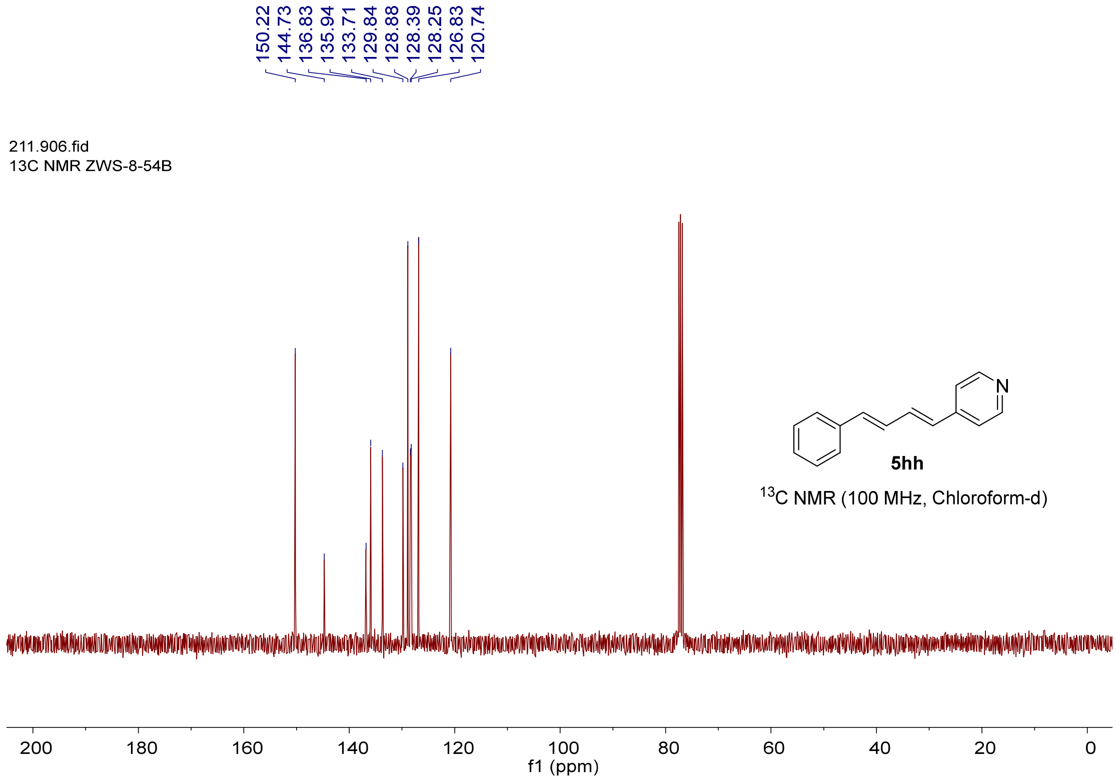

Supplementary Figure 84. **^{13}C NMR** of compound **5hh**

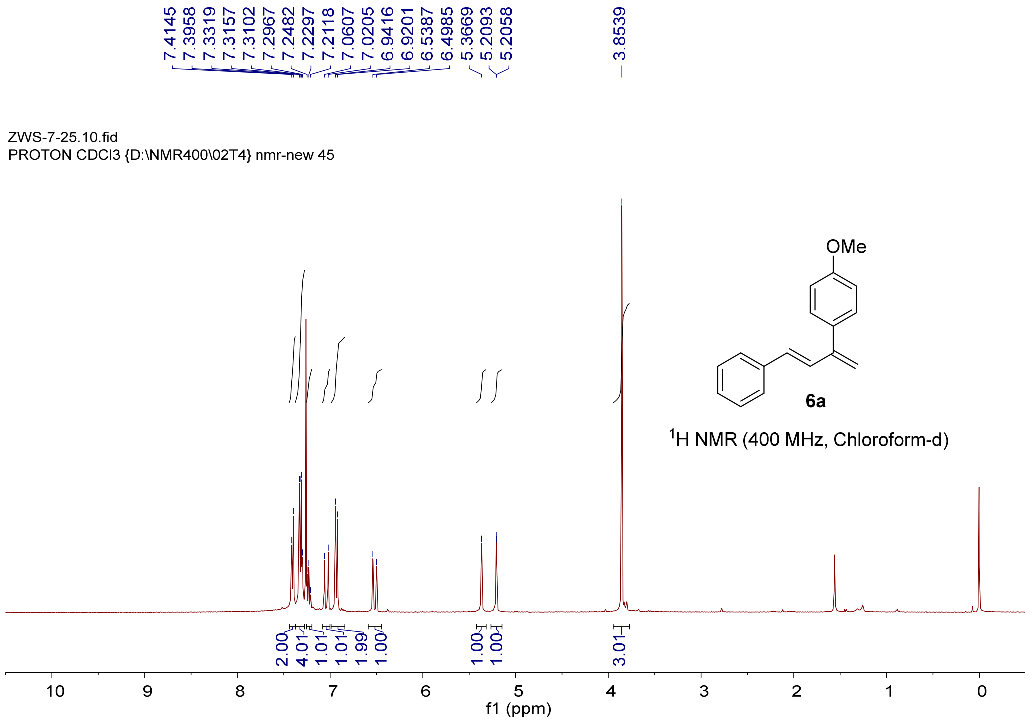

Supplementary Figure 85. ^{1}**H NMR** of compound **6a**

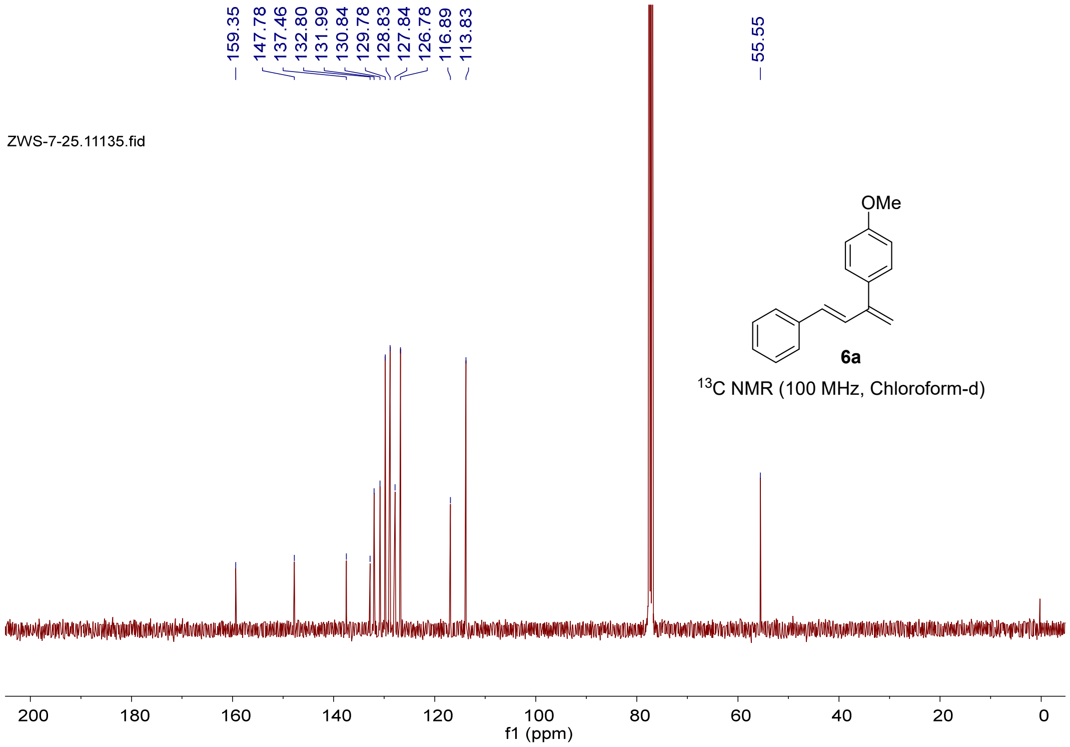

Supplementary Figure 86. ^{13}**C NMR** of compound **6a**

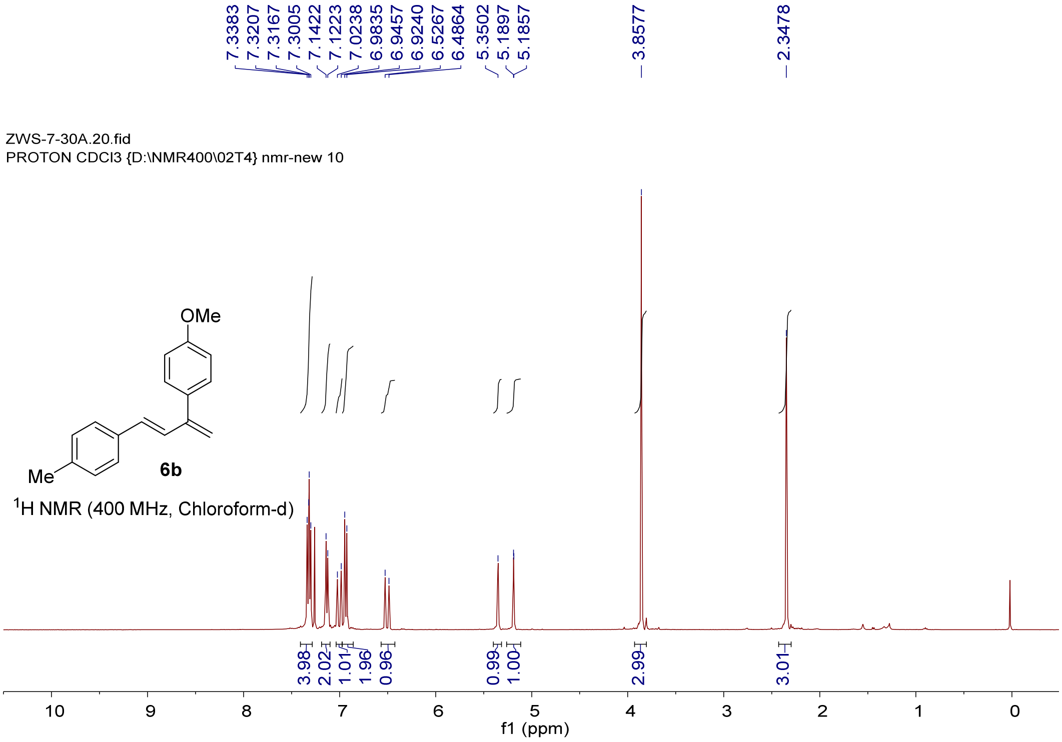

Supplementary Figure 87. ^{1}**H NMR** of compound **6b**

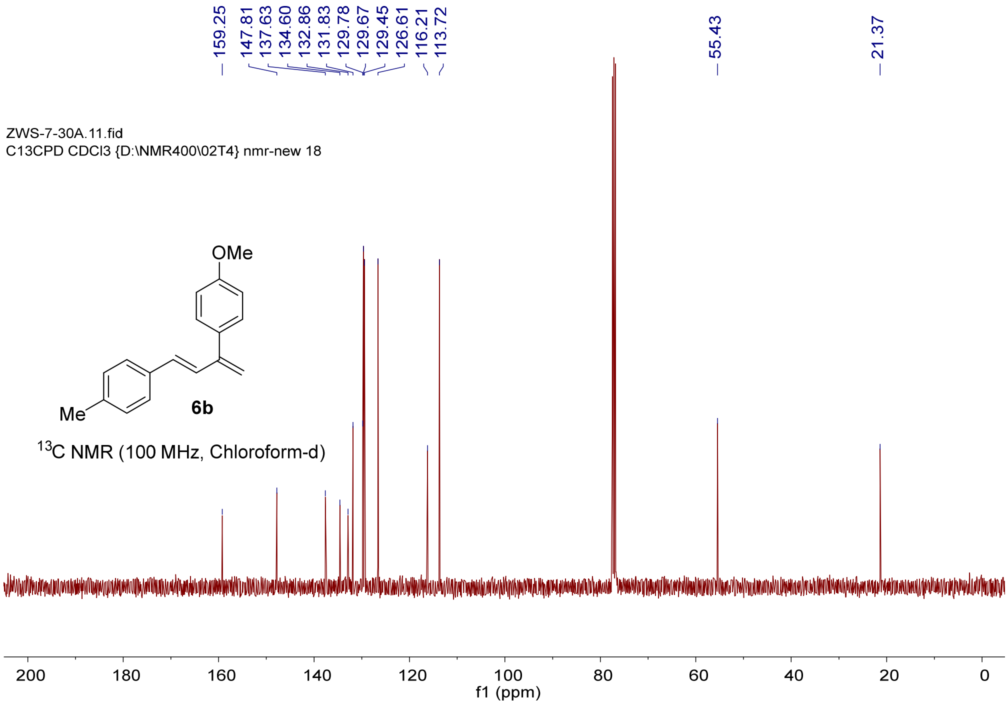

Supplementary Figure 88. ^{13}**C NMR** of compound **6b**

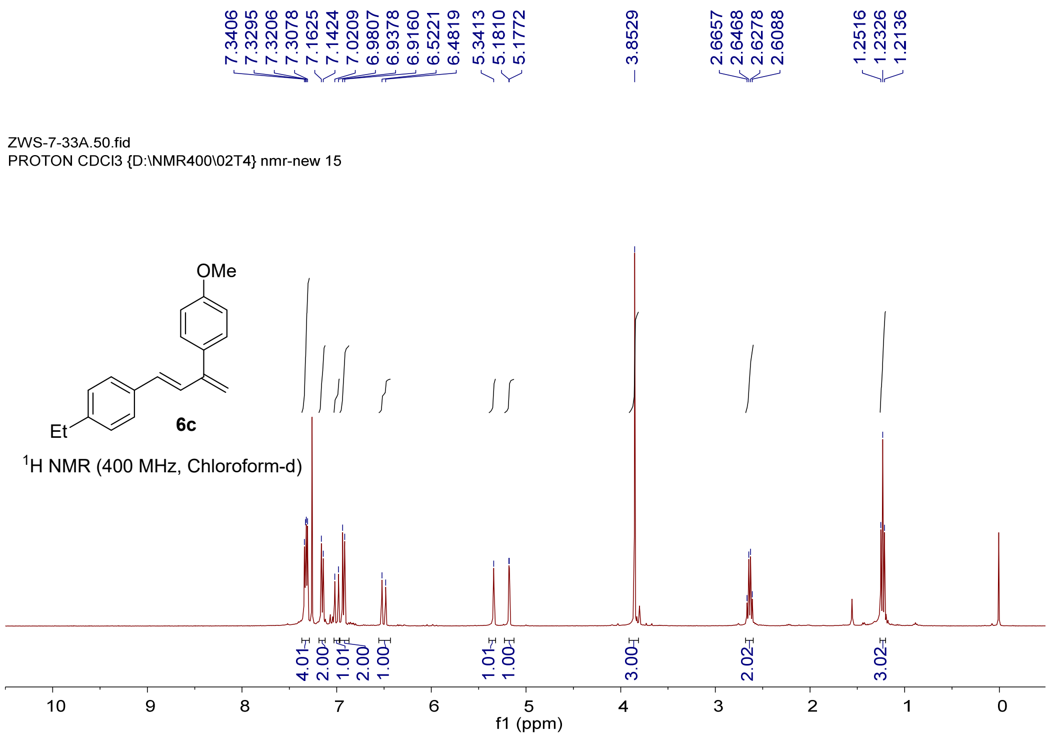

Supplementary Figure 89. ^{1}**H NMR** of compound **6c**

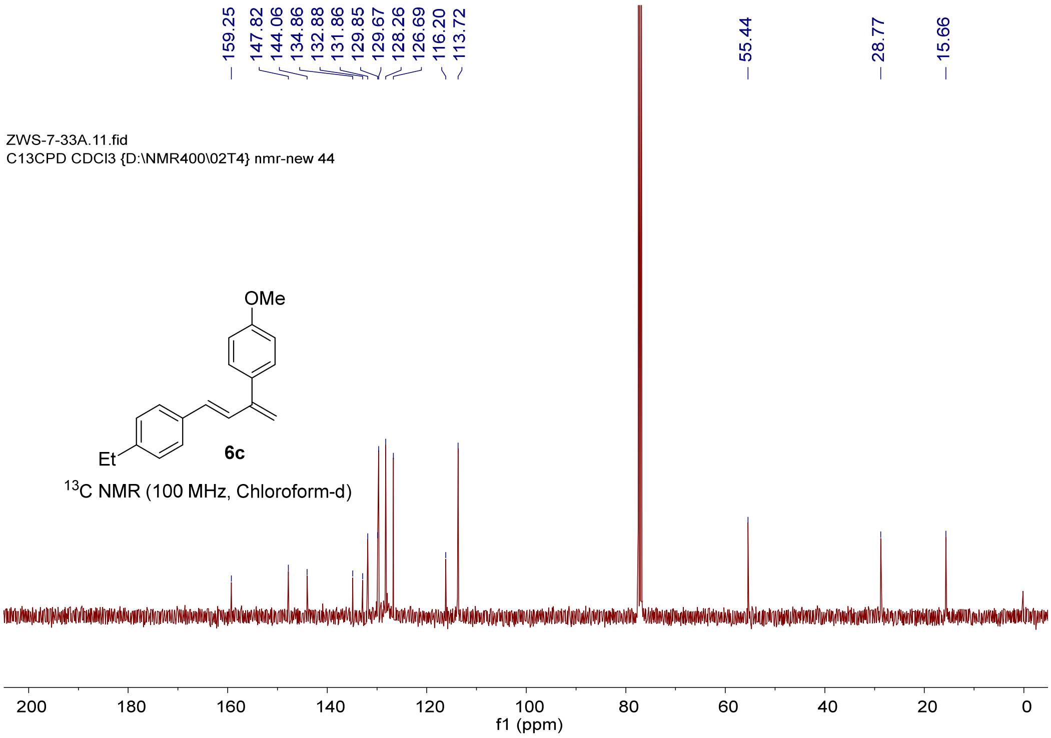

Supplementary Figure 90. ^{13}**C NMR** of compound **6c**

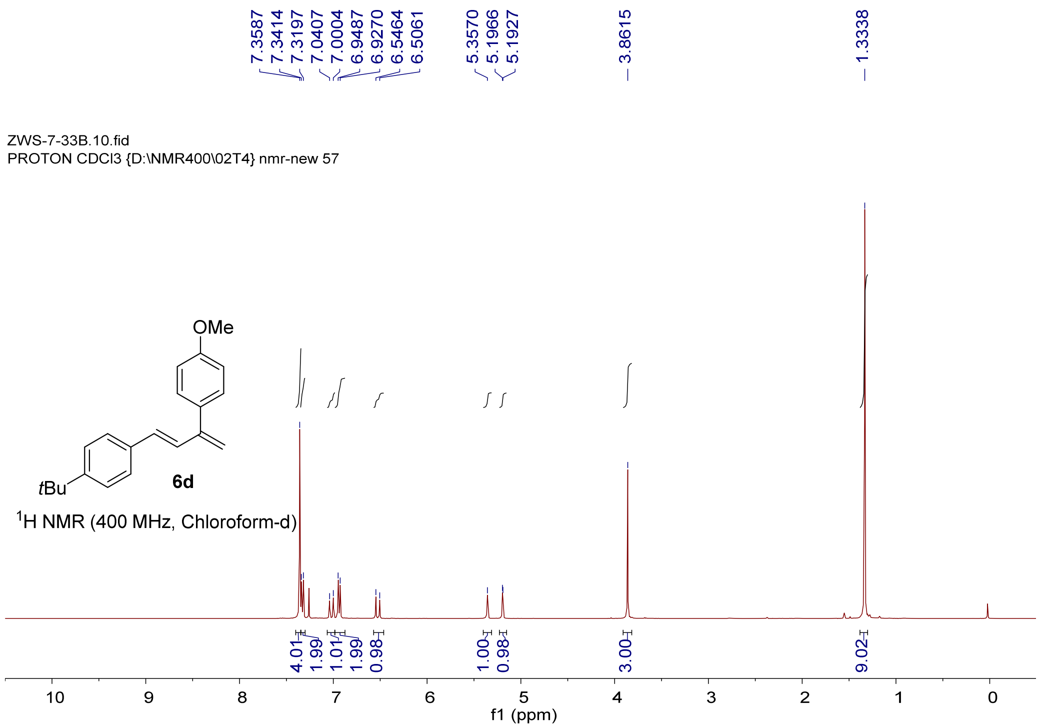

Supplementary Figure 91. ^{1}H NMR of compound **6d**

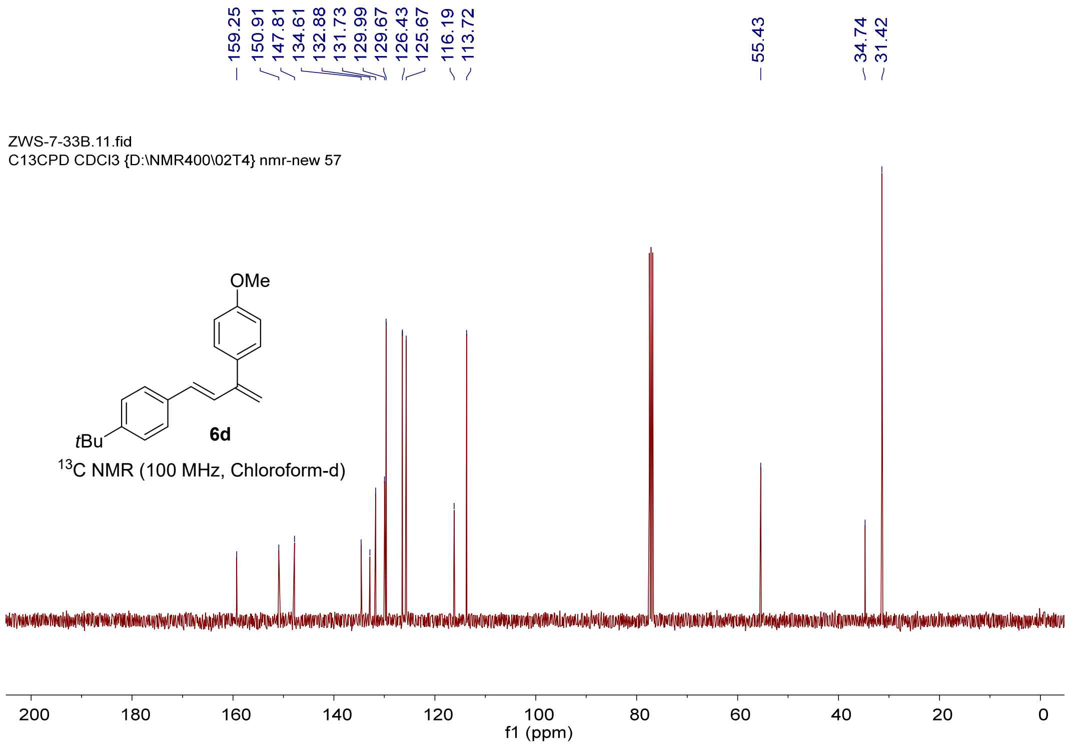

Supplementary Figure 92. ^{13}C NMR of compound **6d**

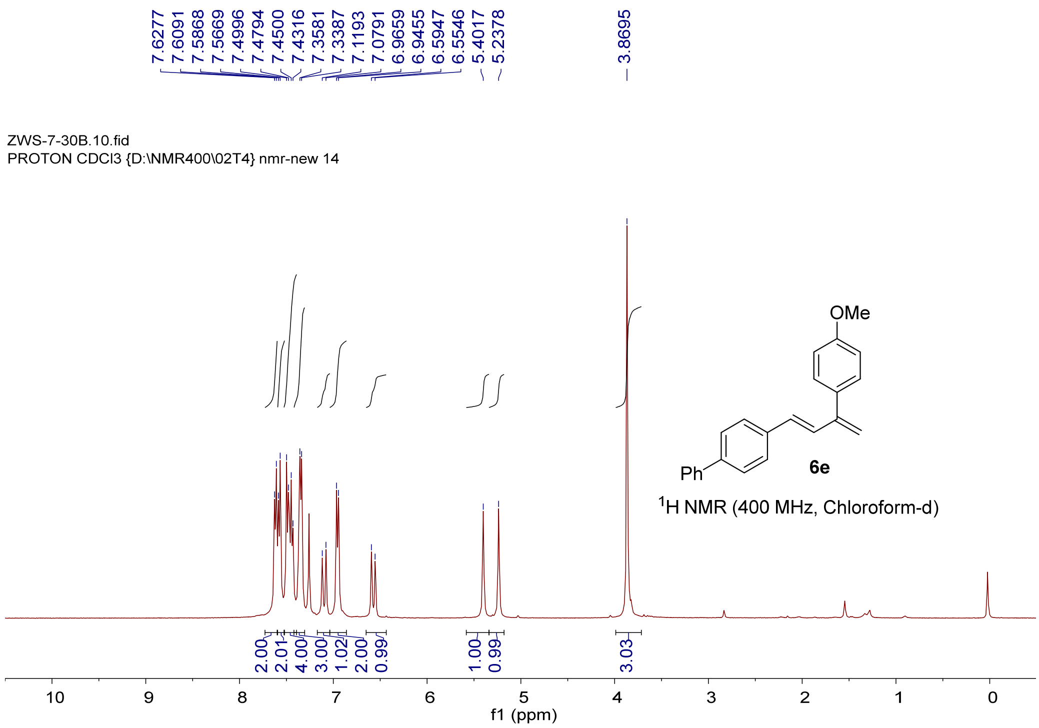

Supplementary Figure 93. **^{1}H NMR** of compound **6e**

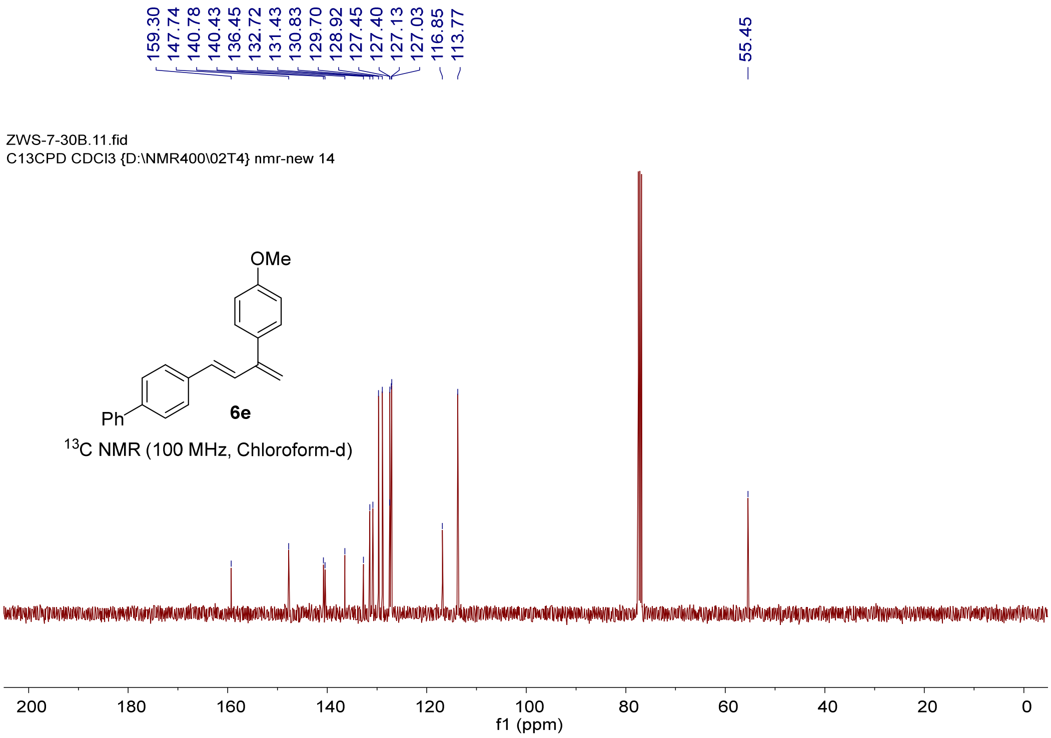

Supplementary Figure 94. **^{13}C NMR** of compound **6e**

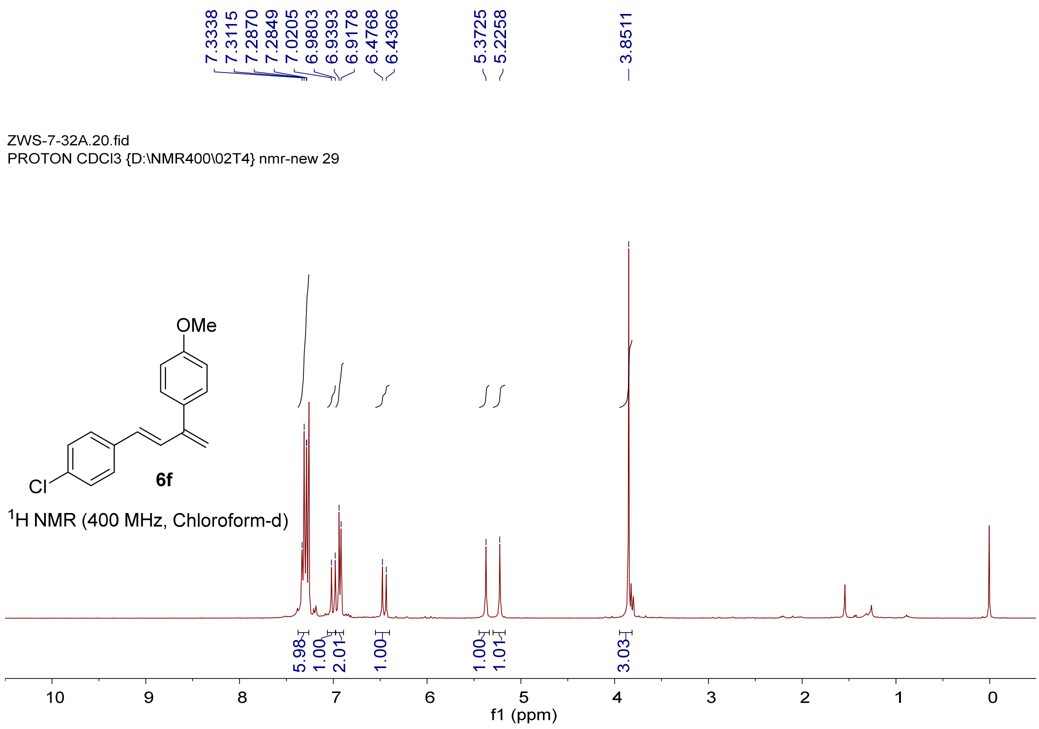

Supplementary Figure 95. ^{1}**H NMR** of compound **6f**

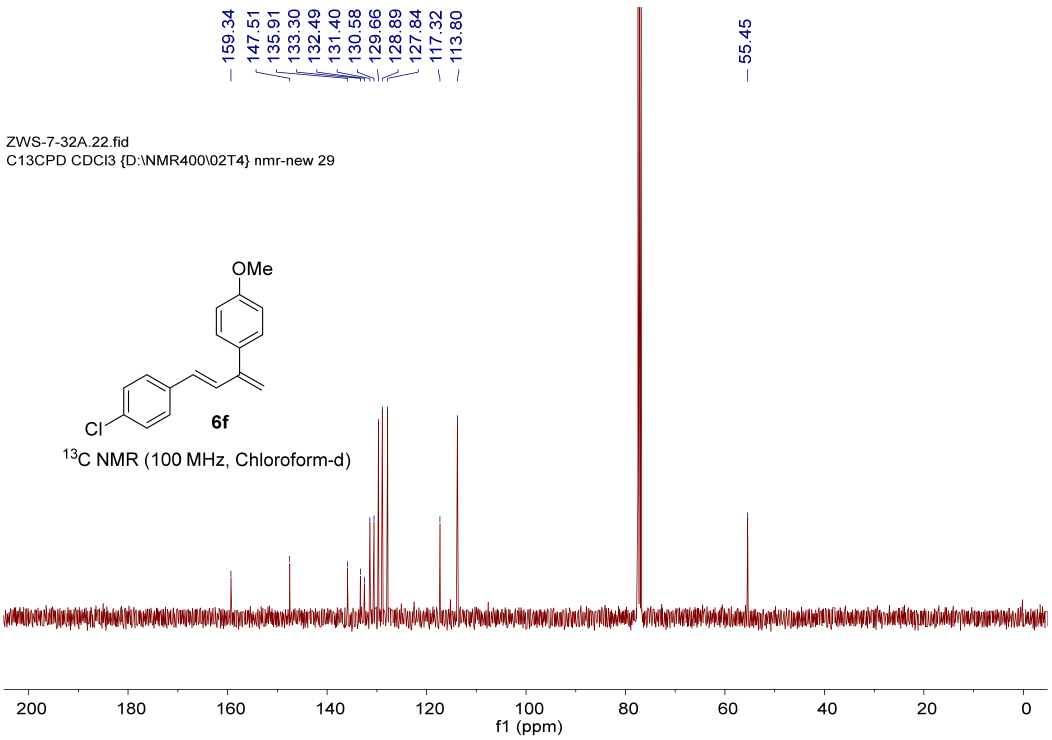

Supplementary Figure 96. ^{13}**C NMR** of compound **6f**

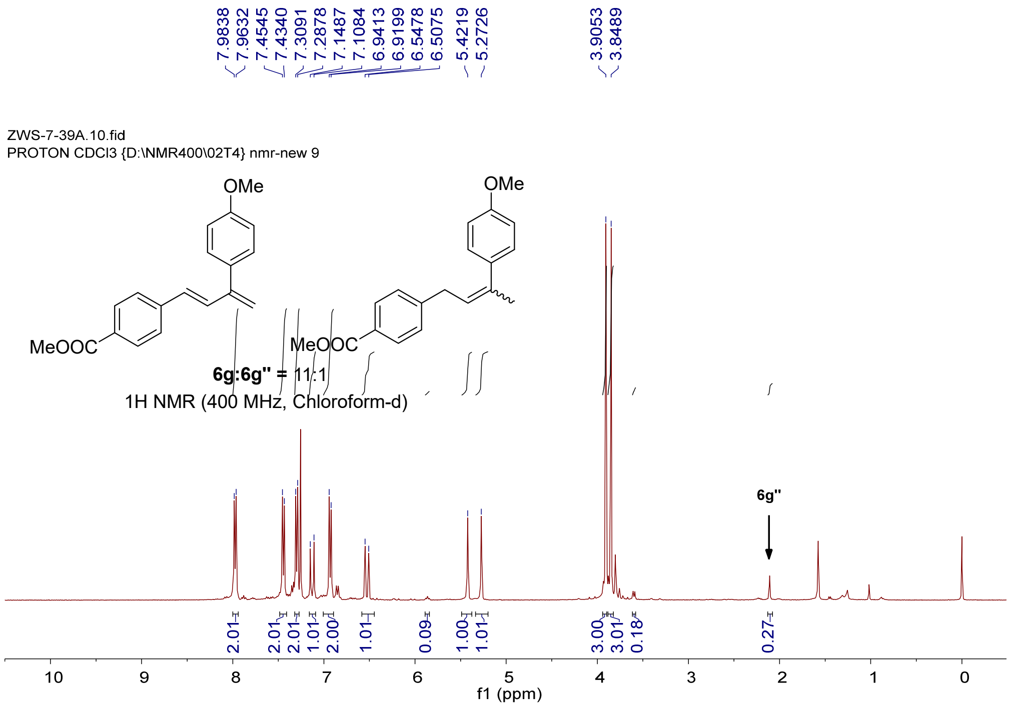

Supplementary Figure 97. ^{1}**H NMR** of compound **6g** and **6g''** **(6g:6g'' = 11:1)**

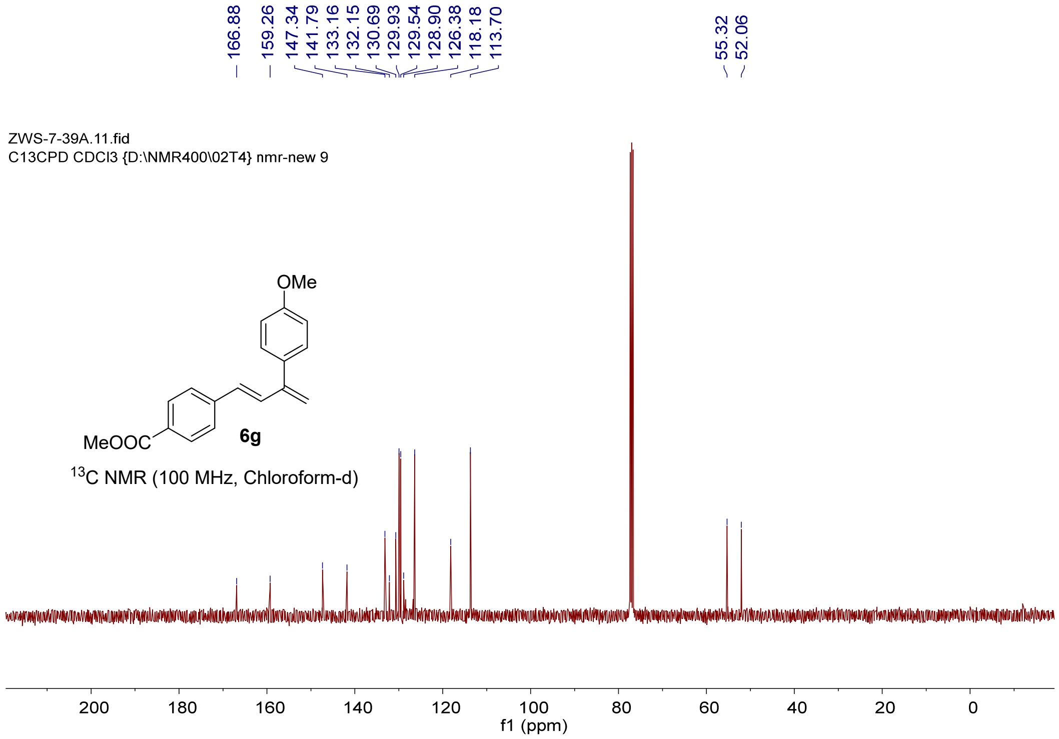

Supplementary Figure 98. ^{13}**C NMR** of compound **6g**

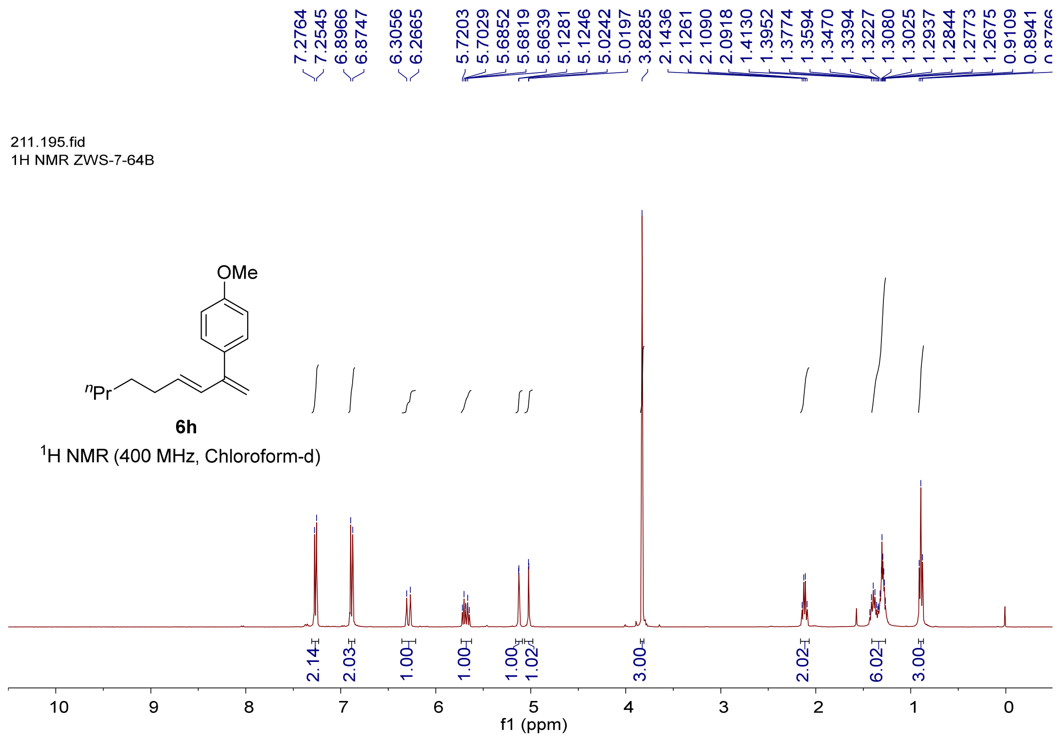

Supplementary Figure 99. **^{1}H NMR** of compound **6h**

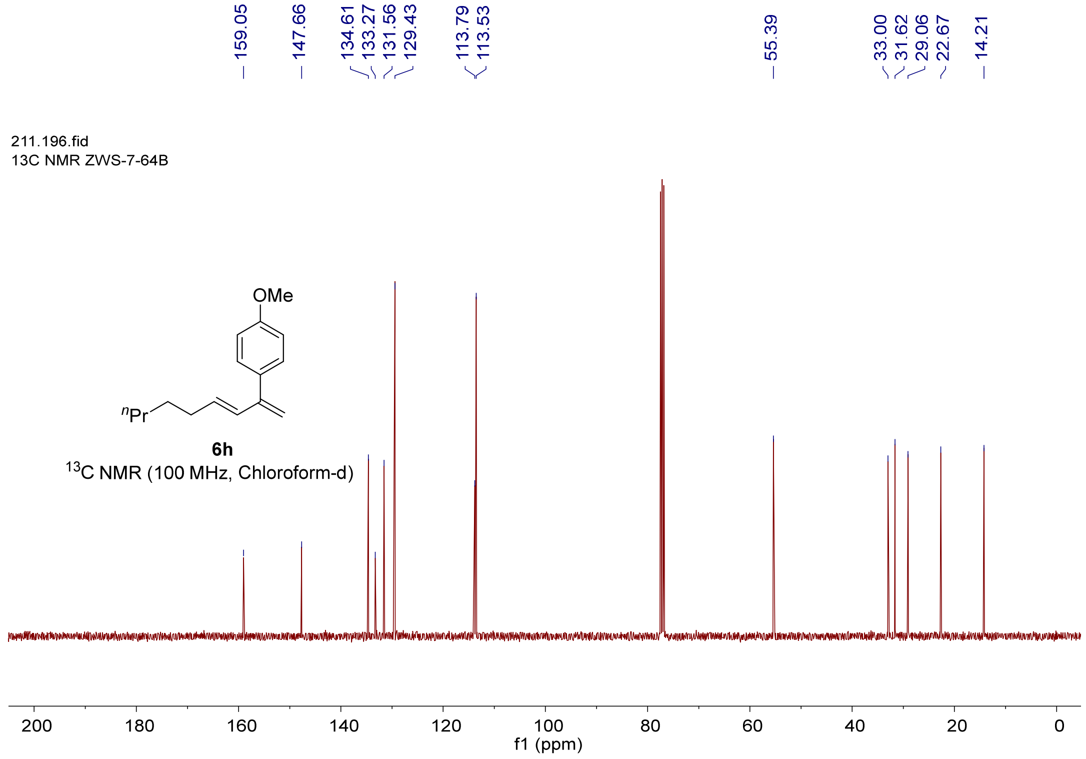

Supplementary Figure 100. **^{13}C NMR** of compound **6h**

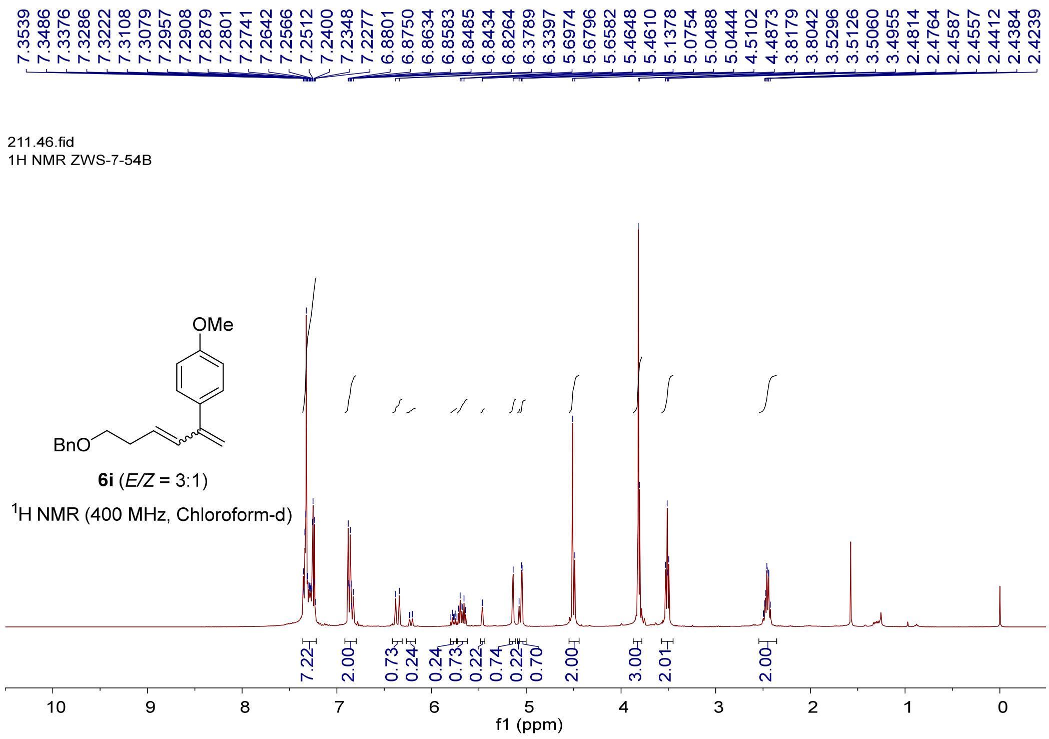

Supplementary Figure 101. ¹H NMR of compound **6i**

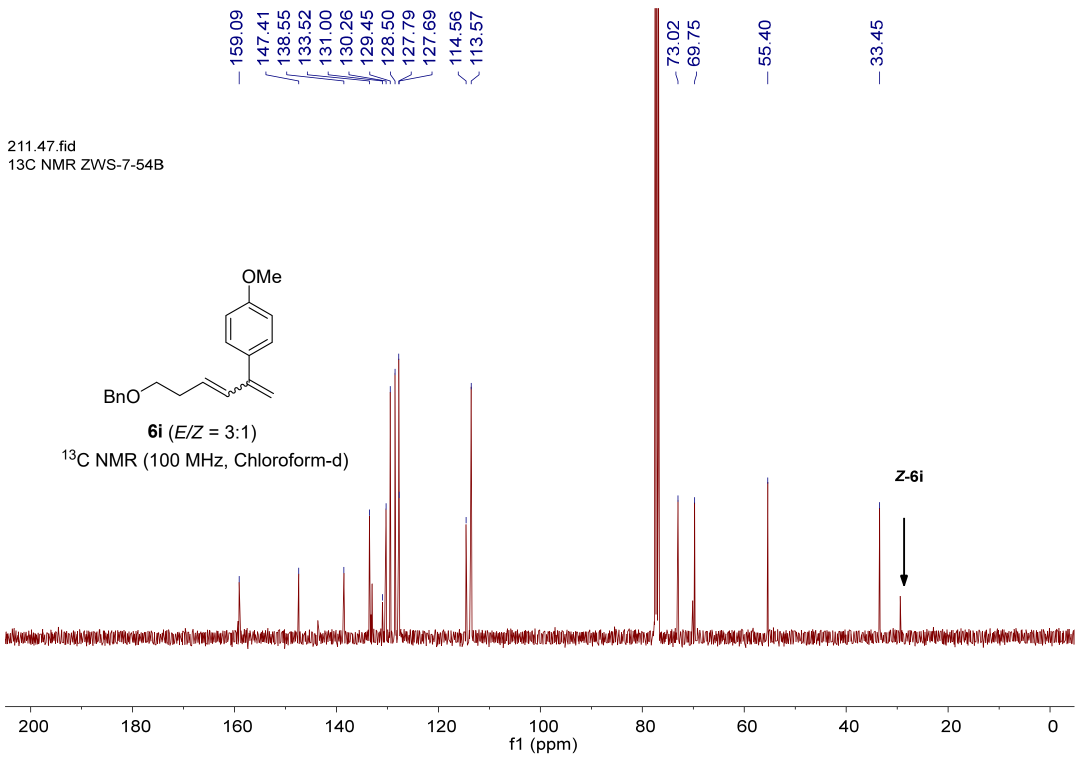

Supplementary Figure 102. ¹³C NMR of compound **6i**

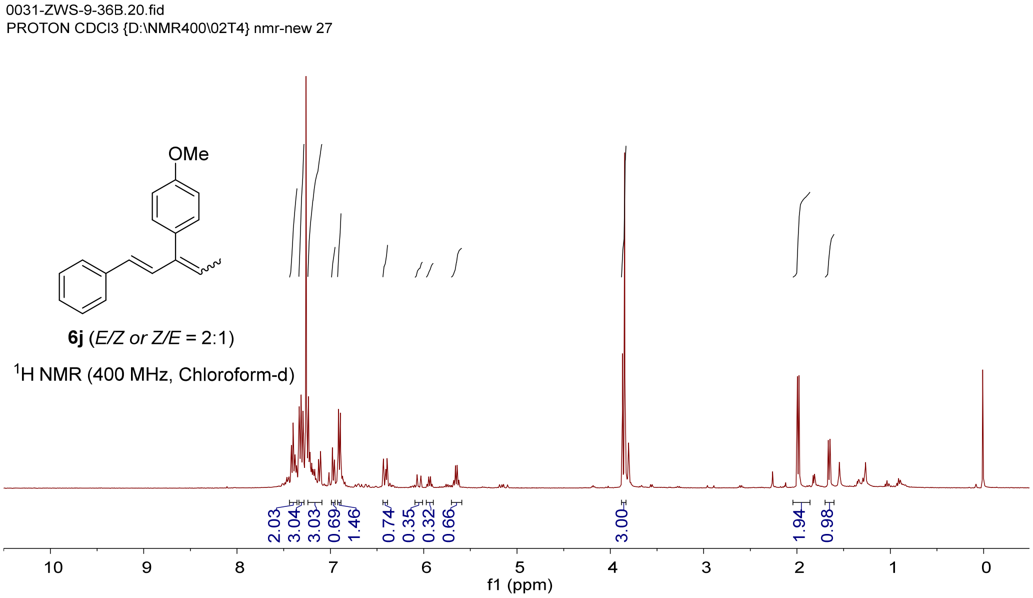

Supplementary Figure 103. ^{1}**H NMR** of compound **6j**

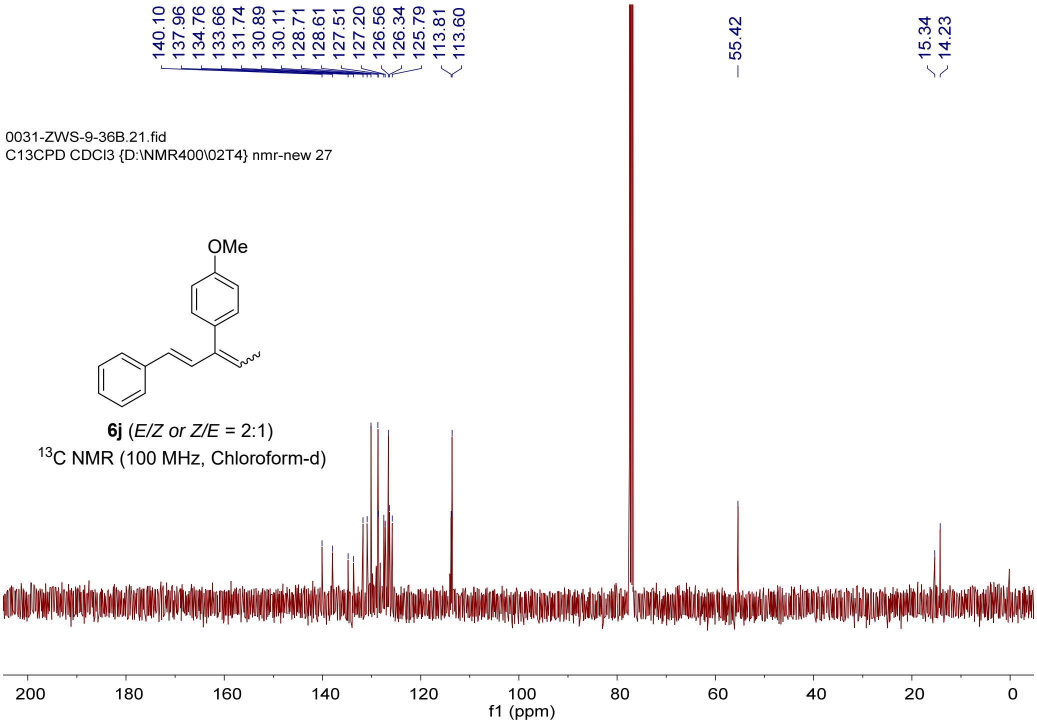

Supplementary Figure 104. ^{13}**C NMR** of compound **6j**

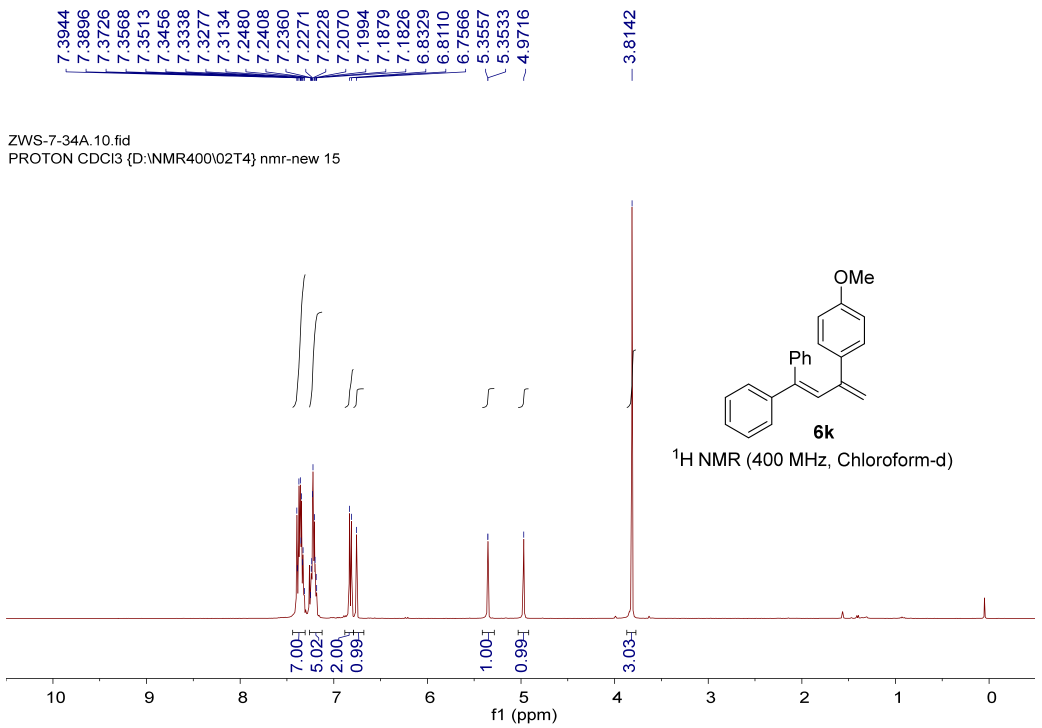

Supplementary Figure 105. ^{1}**H NMR** of compound **6k**

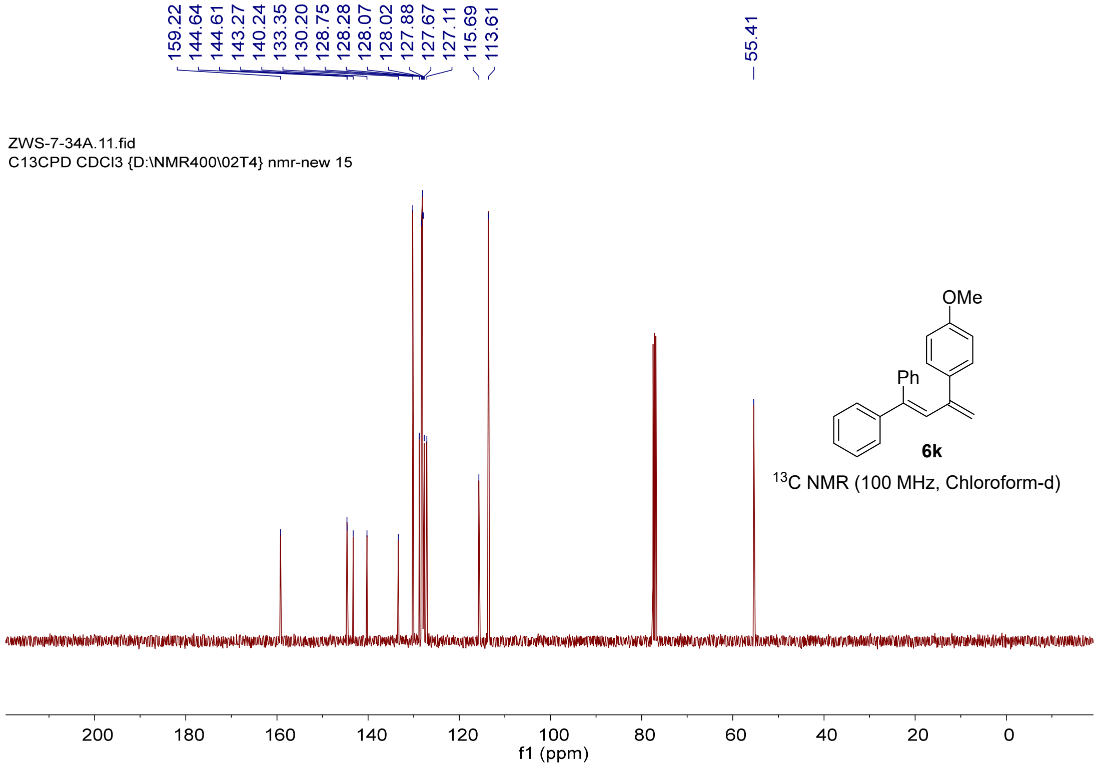

Supplementary Figure 106. ^{13}**C NMR** of compound **6k**

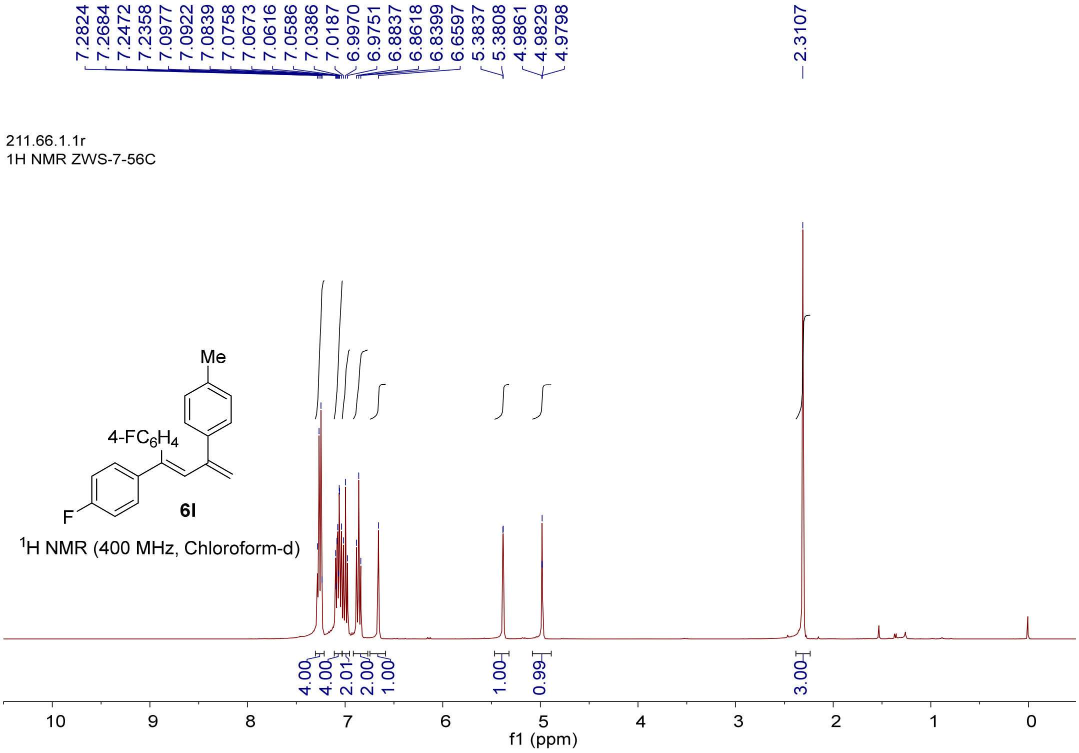

Supplementary Figure 107. ^{1}H NMR of compound **6l**

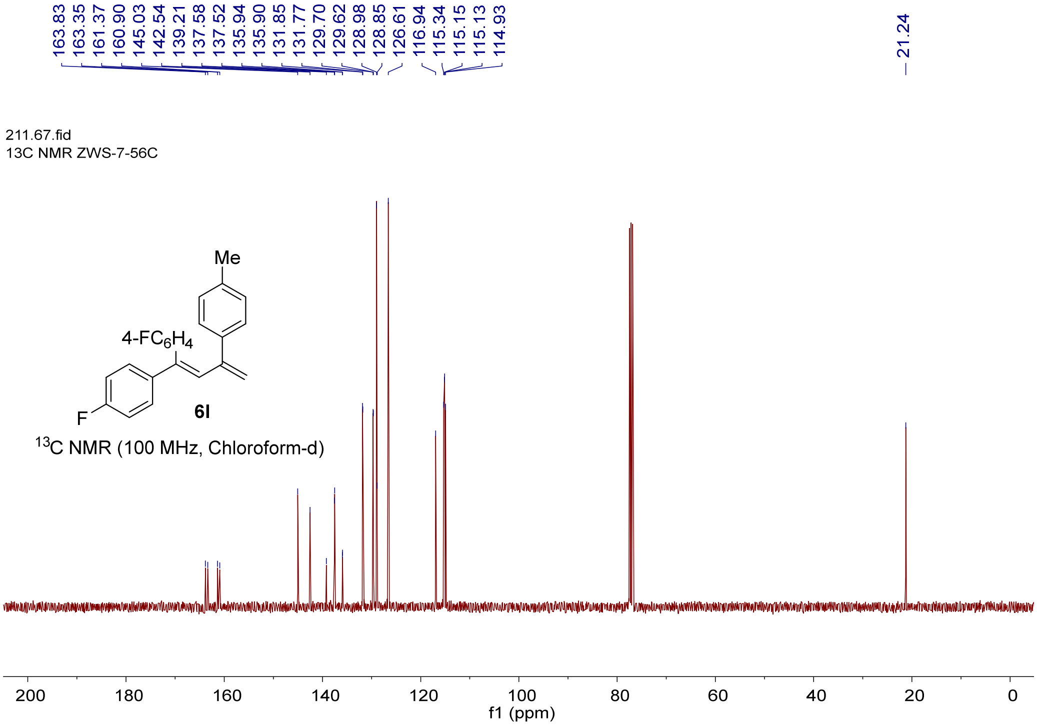

Supplementary Figure 108. ^{13}C NMR of compound **6l**

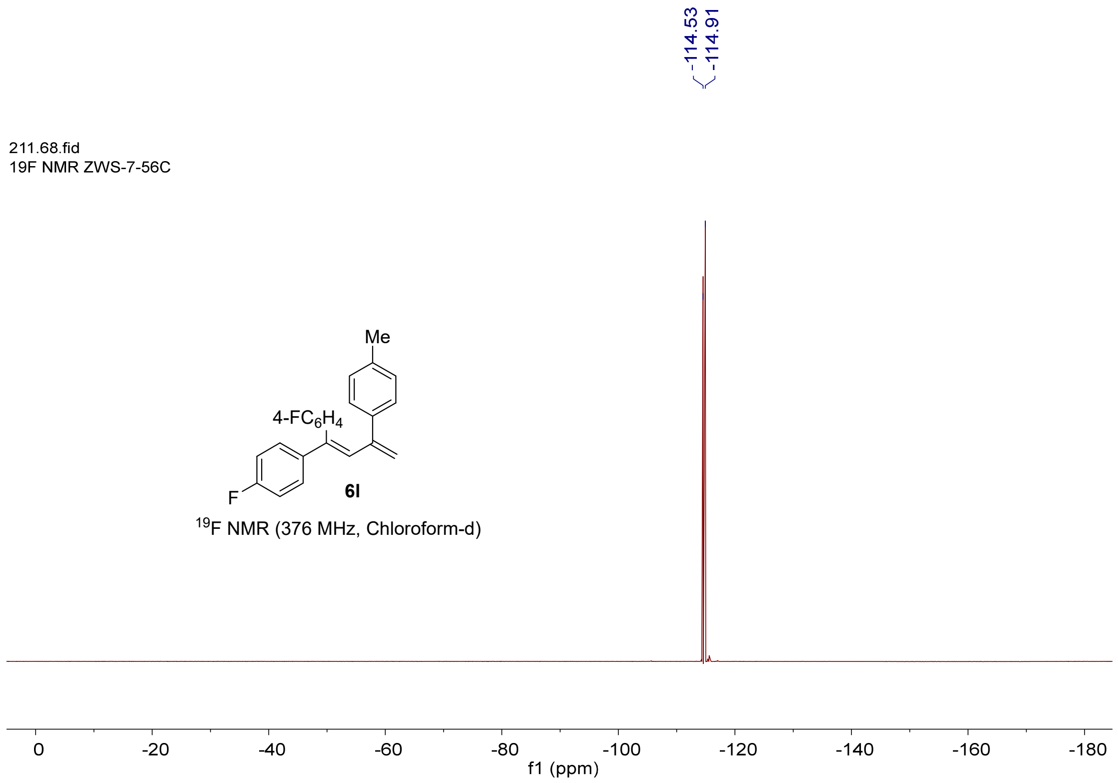

Supplementary Figure 109. ^{19}**F NMR** of compound **6l**

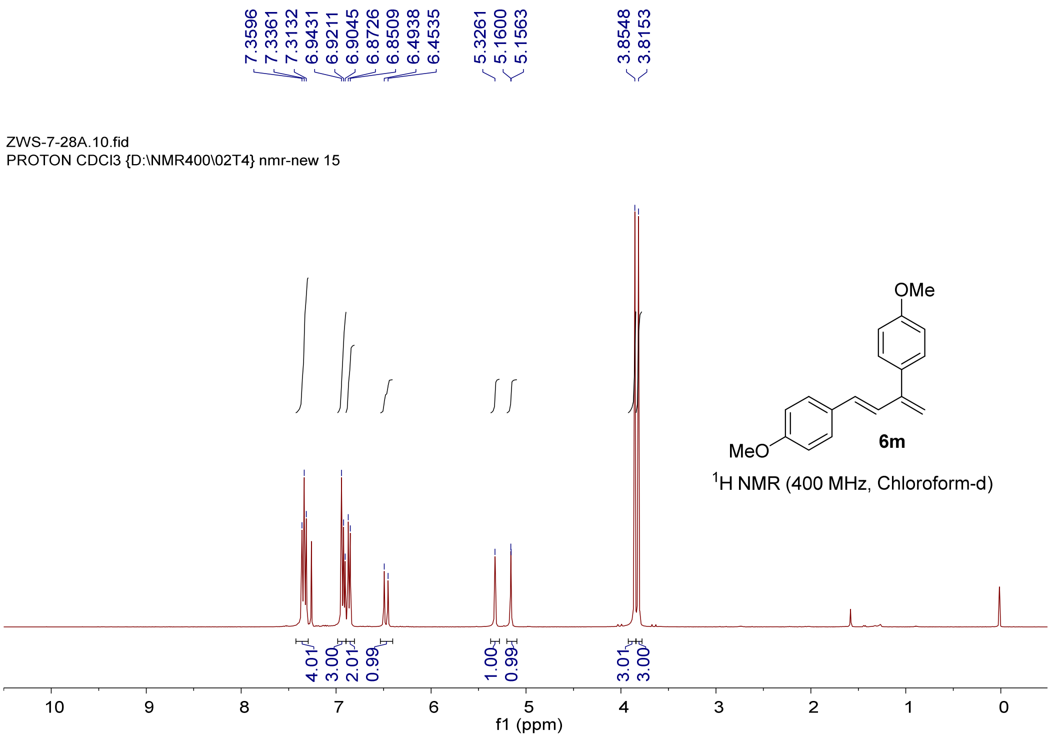

Supplementary Figure 110. ^{1}**H NMR** of compound **6m**

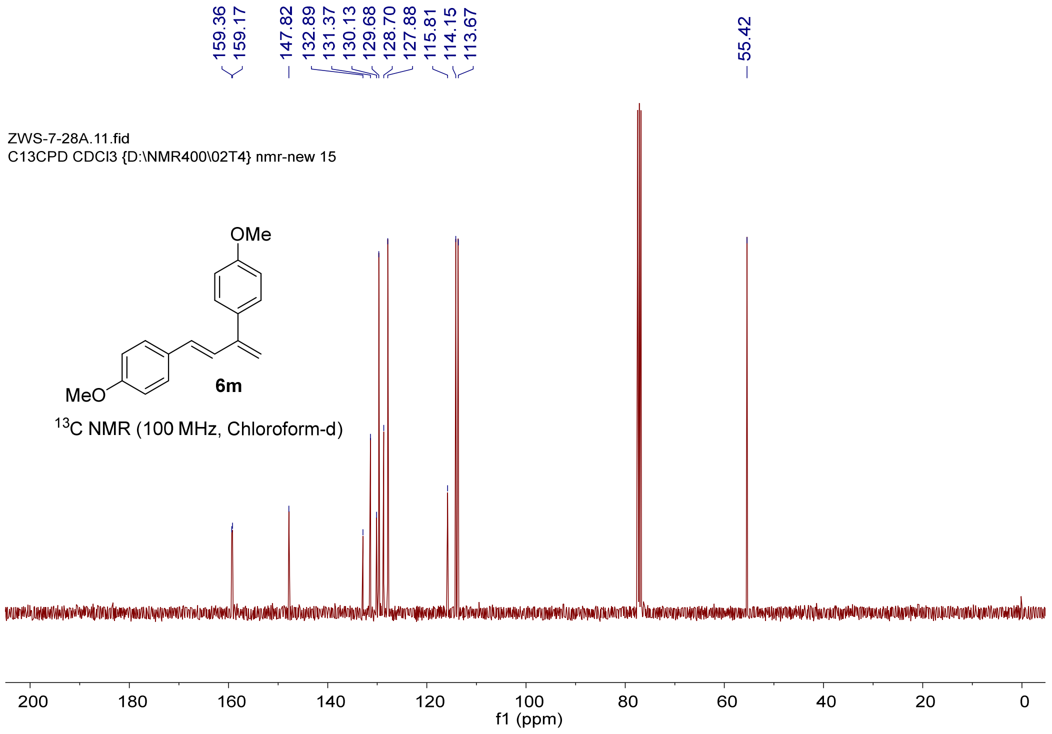

Supplementary Figure 111. ^{13}**C NMR** of compound **6m**

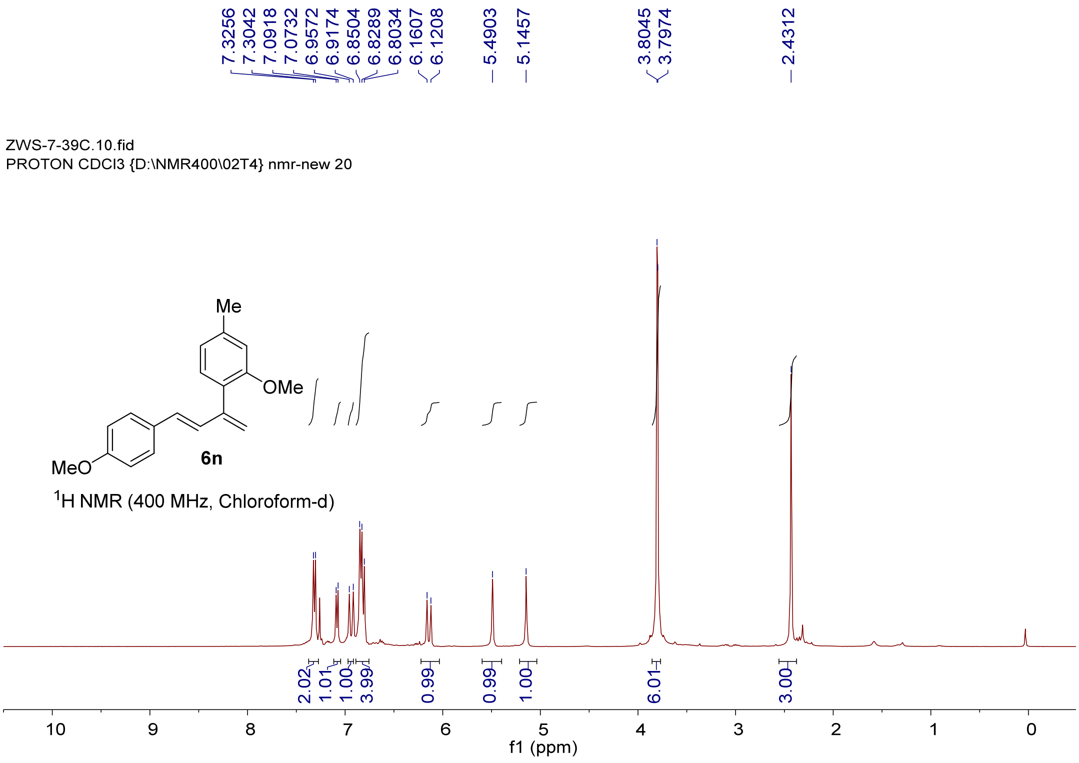

Supplementary Figure 112. ¹H NMR of compound **6n**

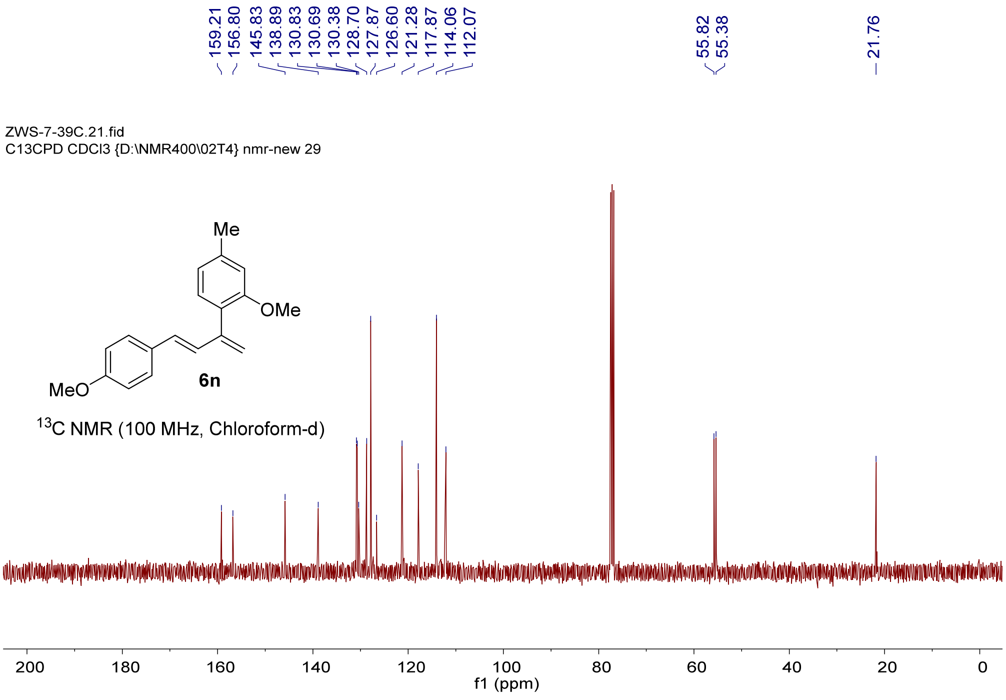

Supplementary Figure 113. ¹³C NMR of compound **6n**

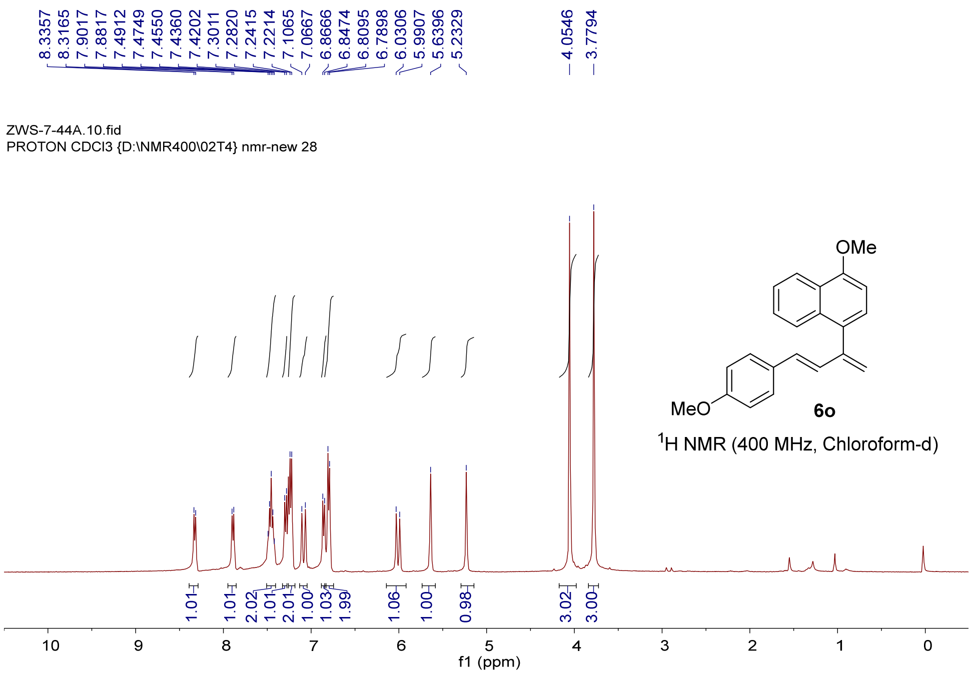

Supplementary Figure 114. ^{1}H NMR of compound **6o**

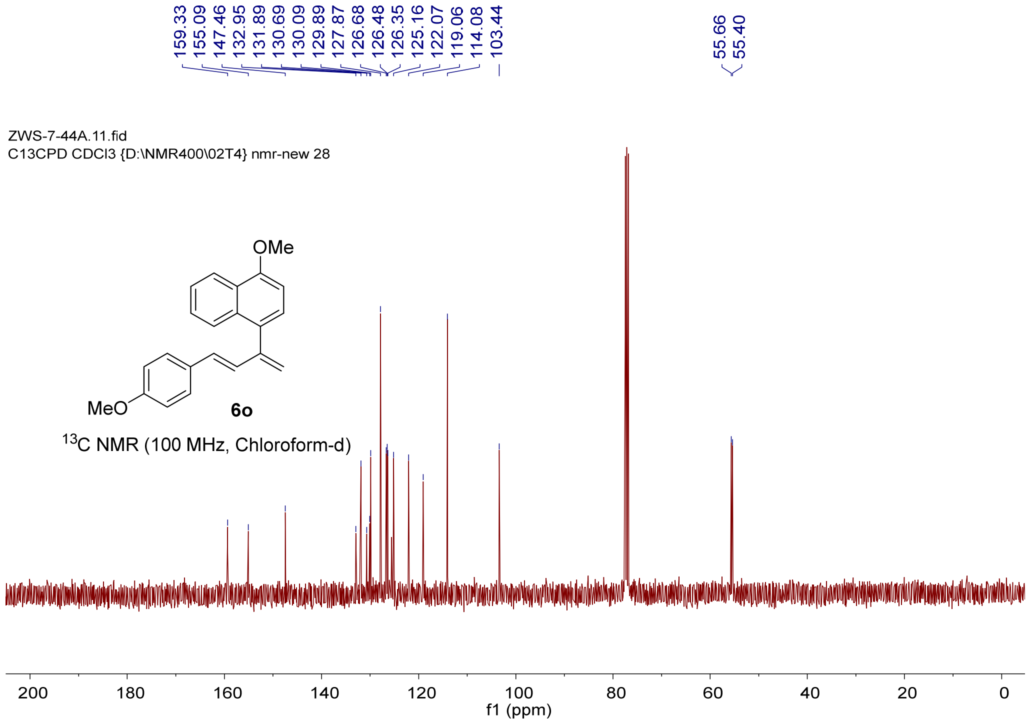

Supplementary Figure 115. ^{13}C NMR of compound **6o**

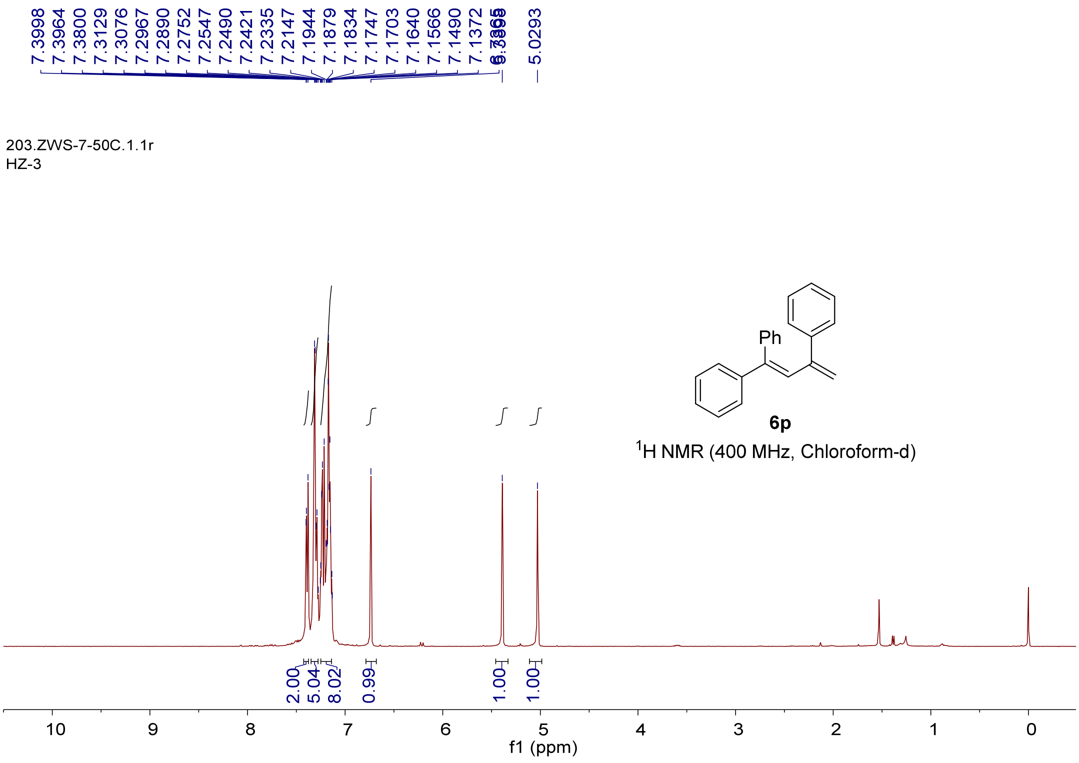

Supplementary Figure 116. ¹H NMR of compound **6p**

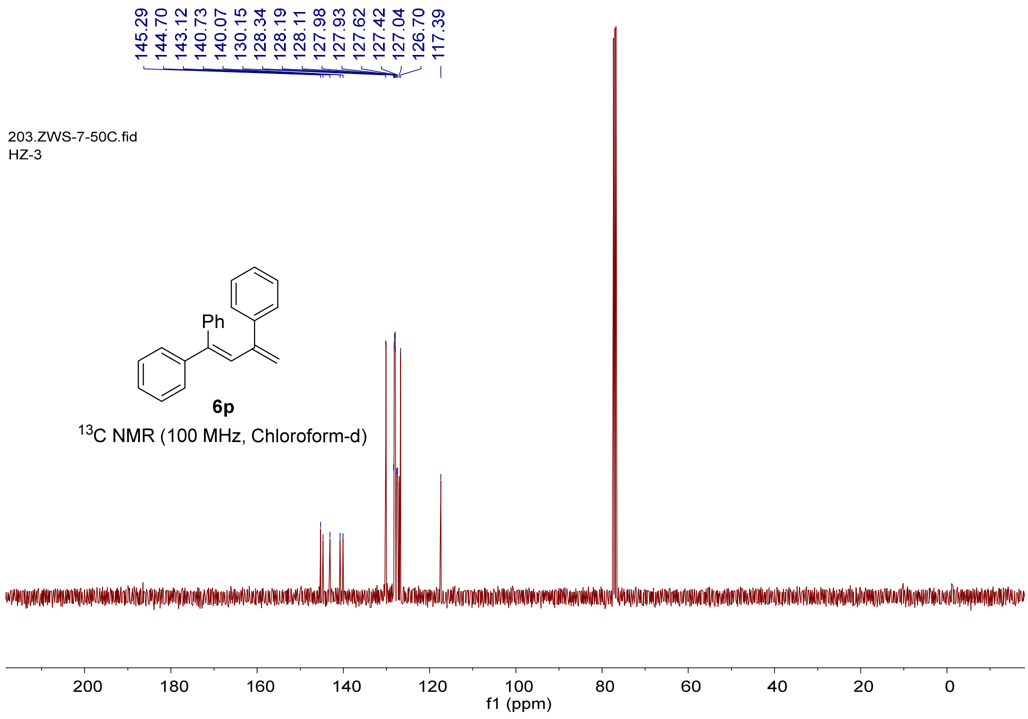

Supplementary Figure 117. ¹³C NMR of compound **6p**

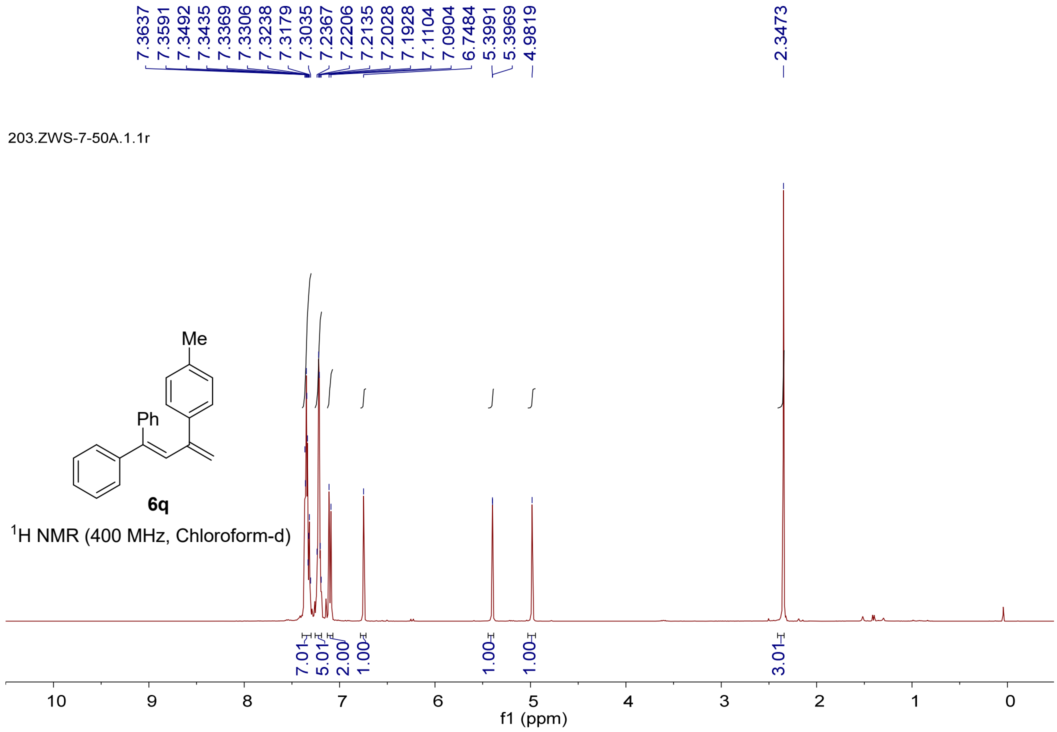

Supplementary Figure 118. **^{1}H NMR** of compound **6q**

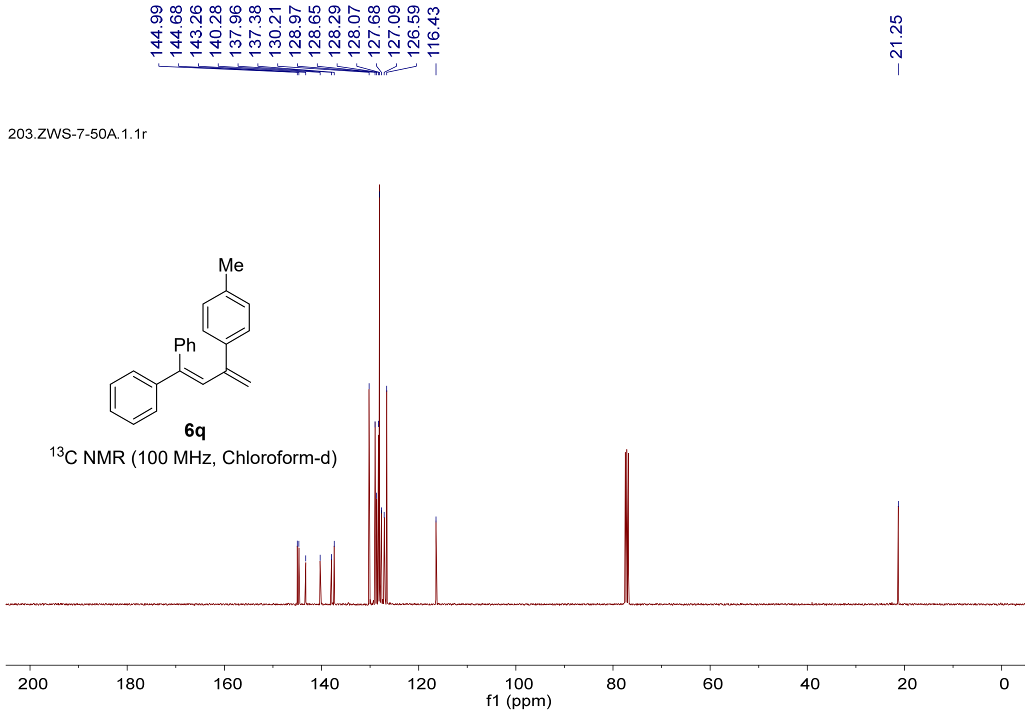

Supplementary Figure 119. **^{13}C NMR** of compound **6q**

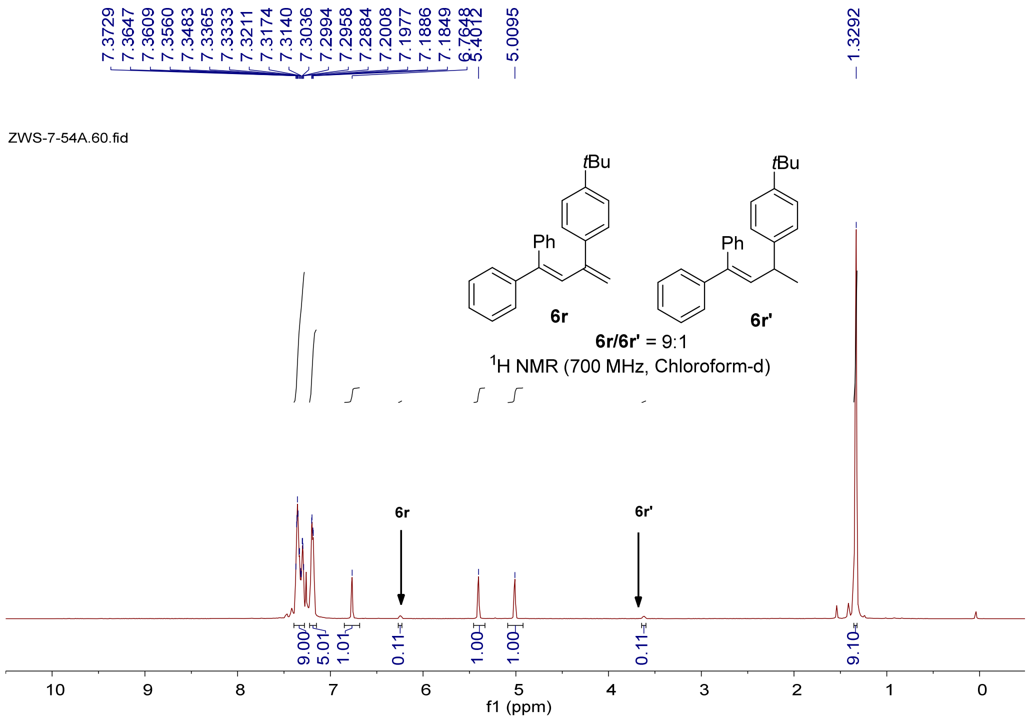

Supplementary Figure 120. **^{1}H NMR** of compound **6r** and **6r'** (**6r/6r'** = 9:1)

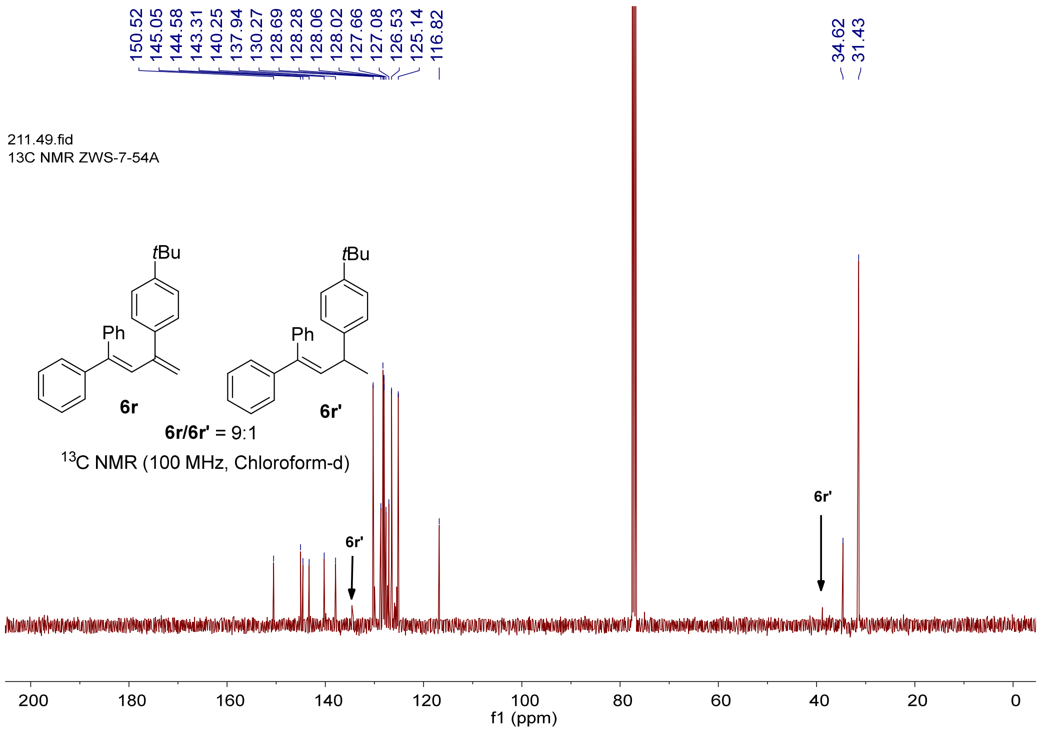

Supplementary Figure 121. **^{13}C NMR** of compound **6r** and **6r'** (**6r/6r'** = 9:1)

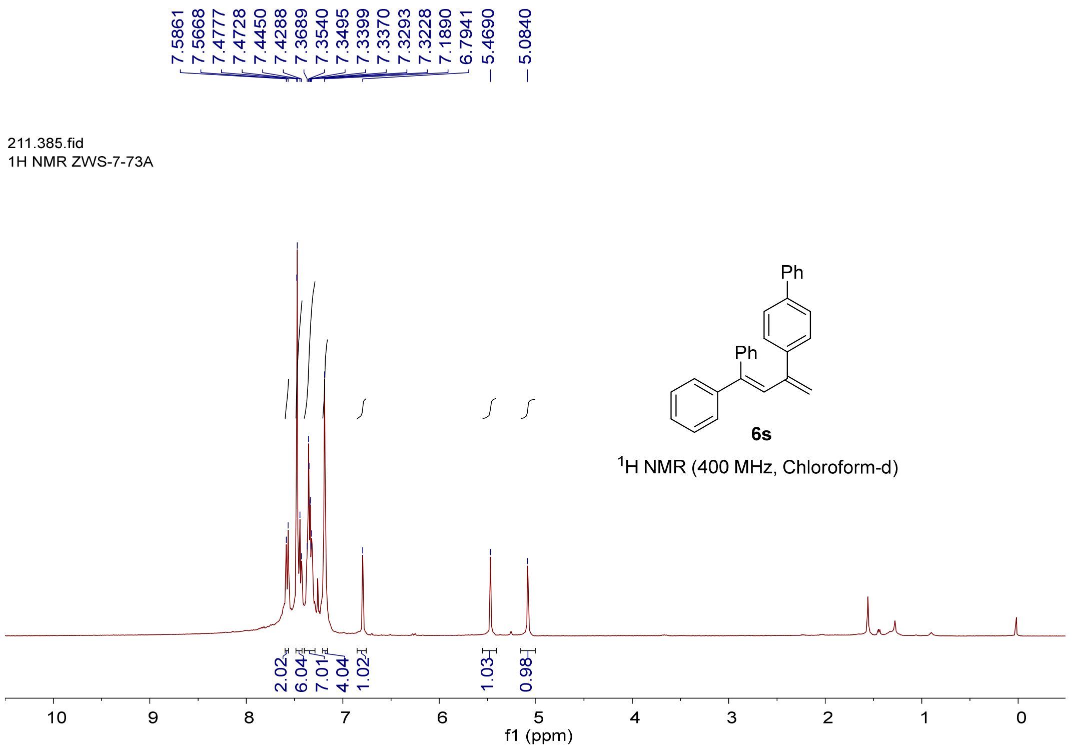

Supplementary Figure 122. ^{1}**H NMR** of compound **6s**

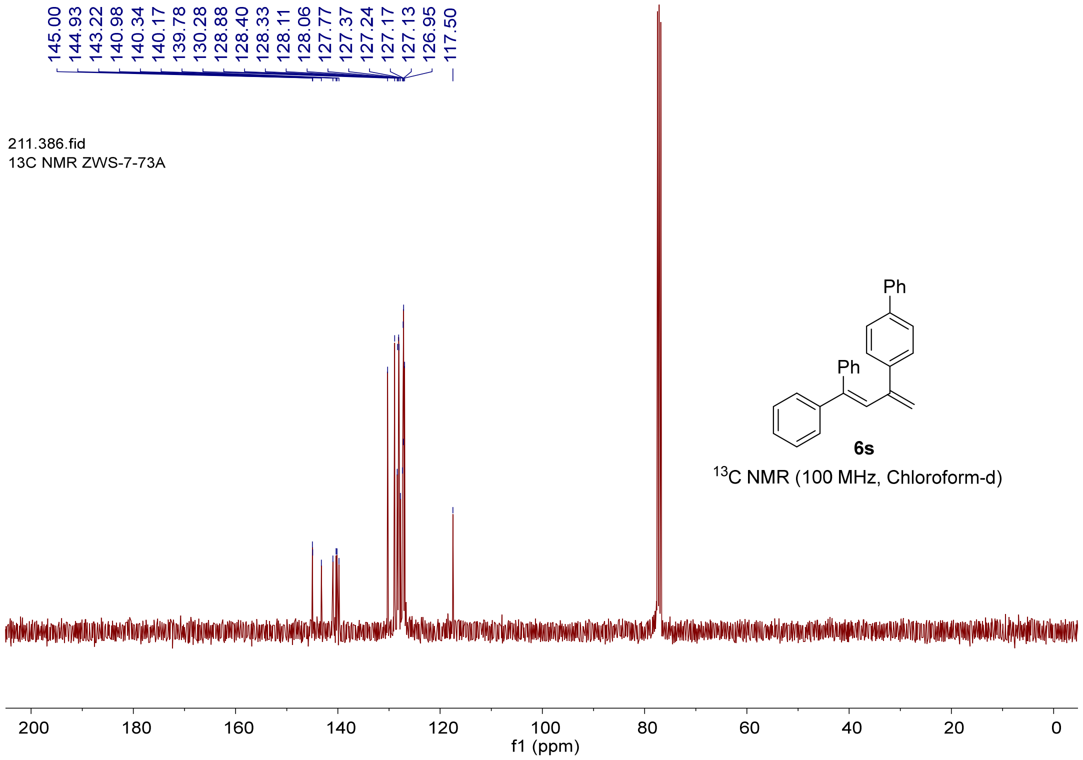

Supplementary Figure 123. ^{13}**C NMR** of compound **6s**

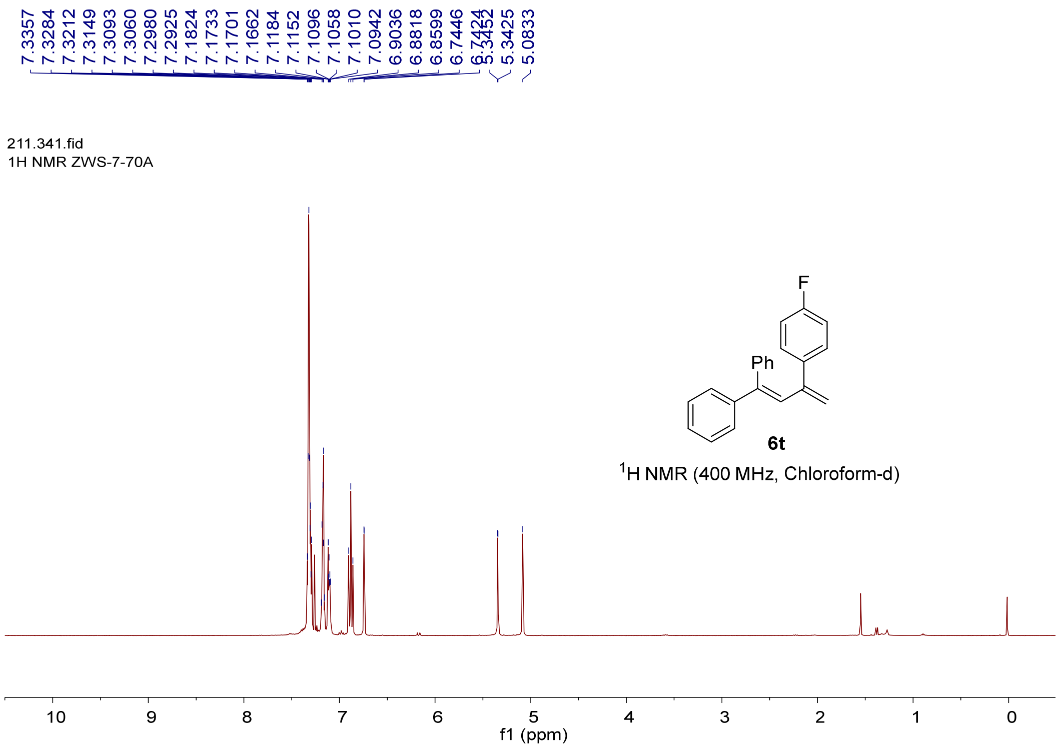

Supplementary Figure 124. 1H NMR of compound **6t**

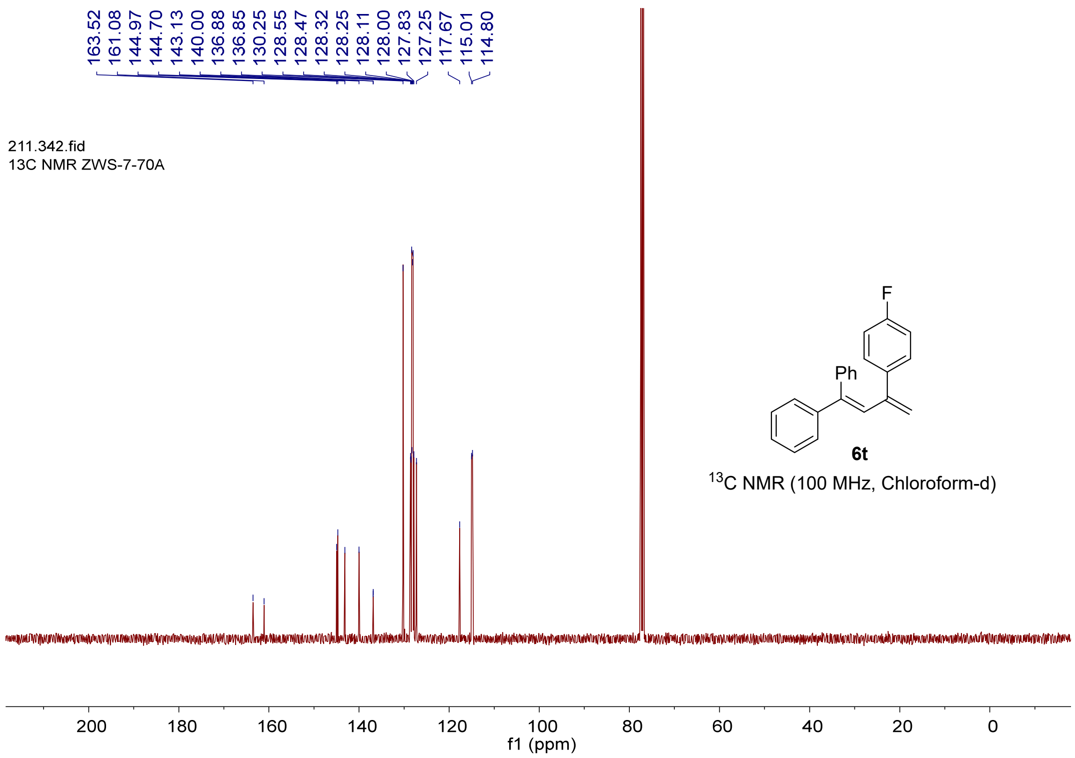

Supplementary Figure 125. 13C NMR of compound **6t**

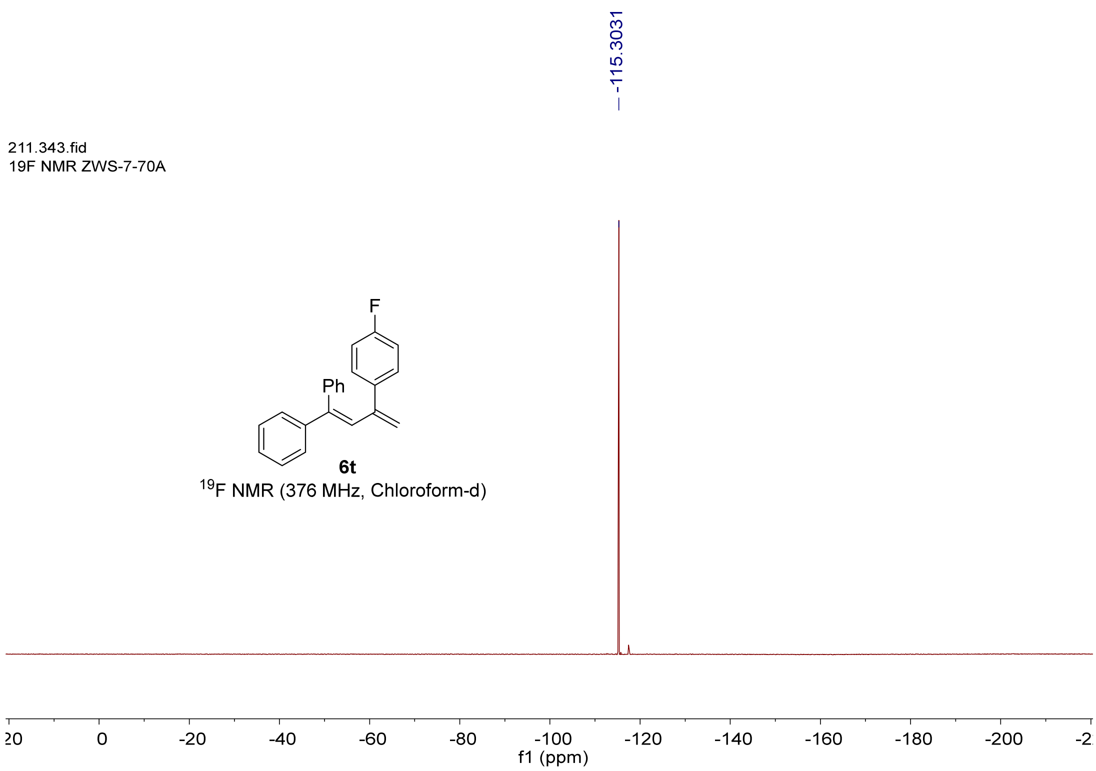

Supplementary Figure 126. ^{19}**F NMR** of compound **6t**

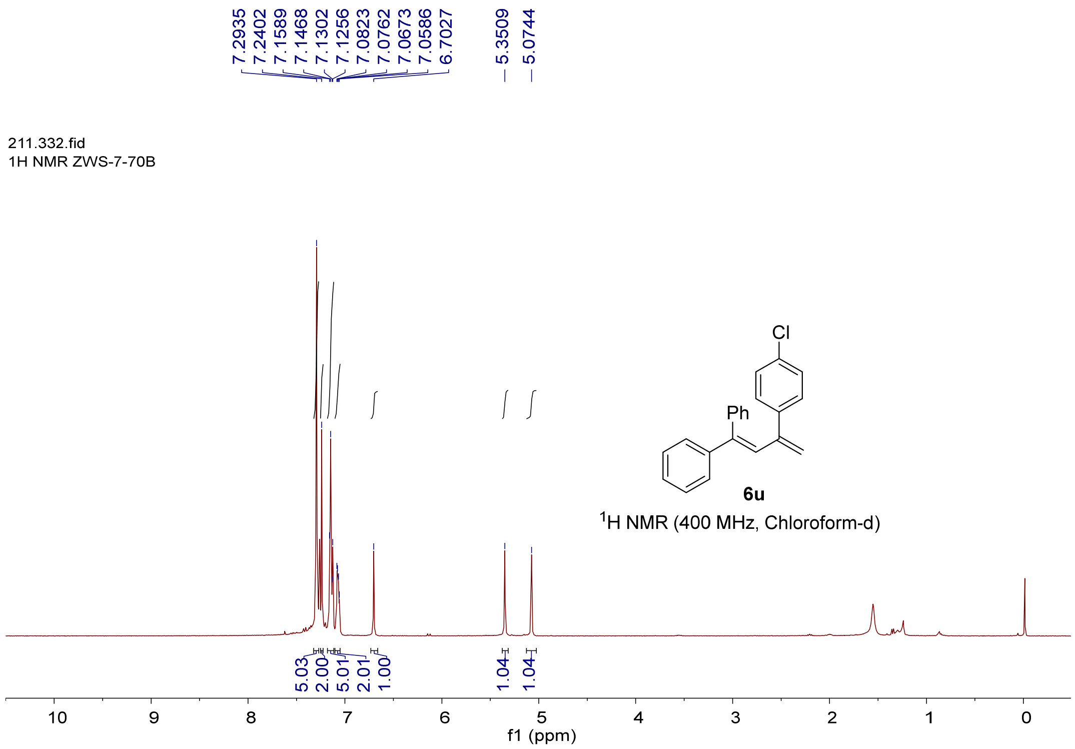

Supplementary Figure 127. **^{1}H NMR** of compound **6u**

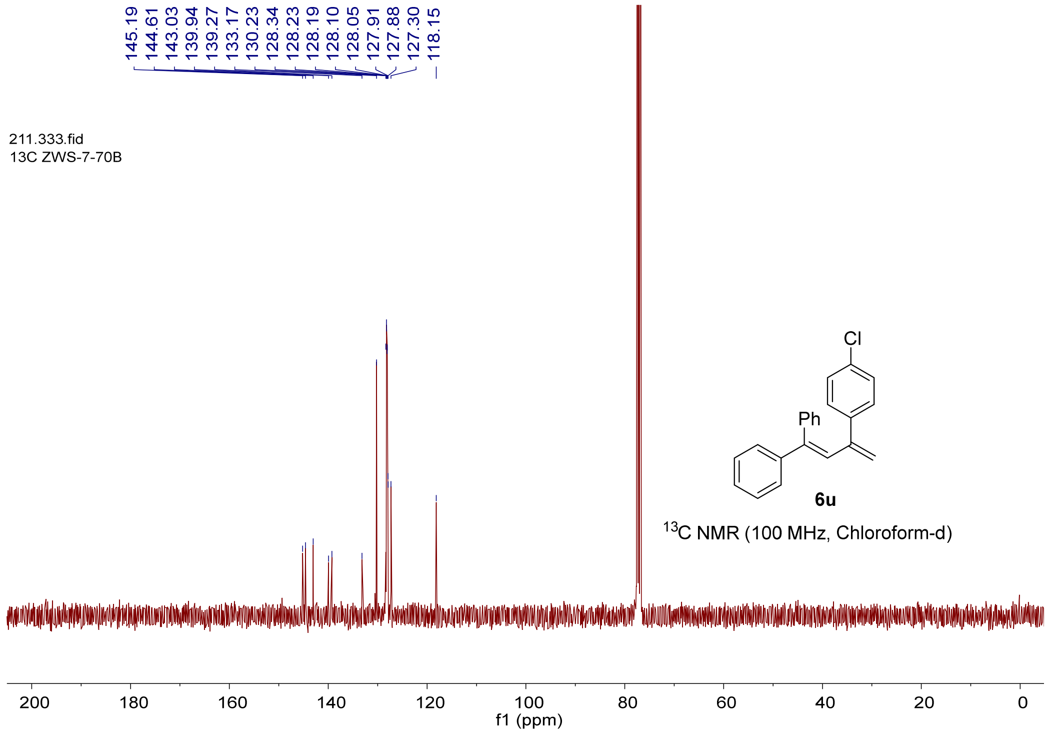

Supplementary Figure 128. **^{13}C NMR** of compound **6u**

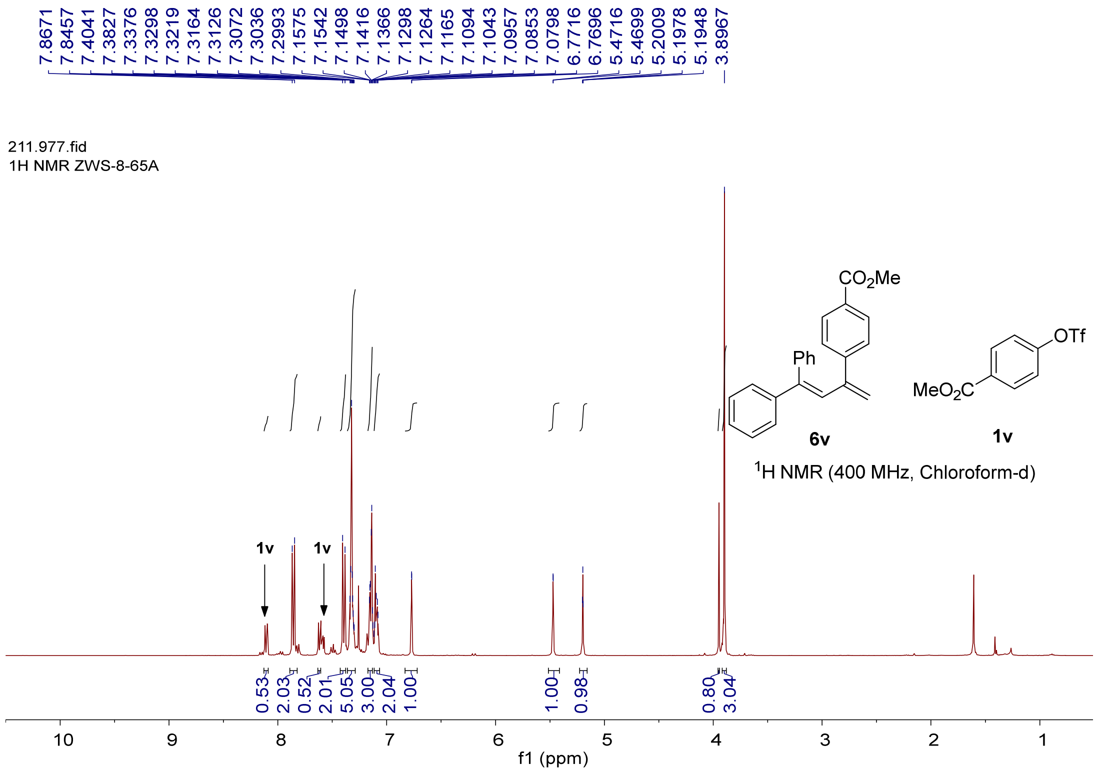

Supplementary Figure 129. ^{1}**H NMR** of compound **6v**

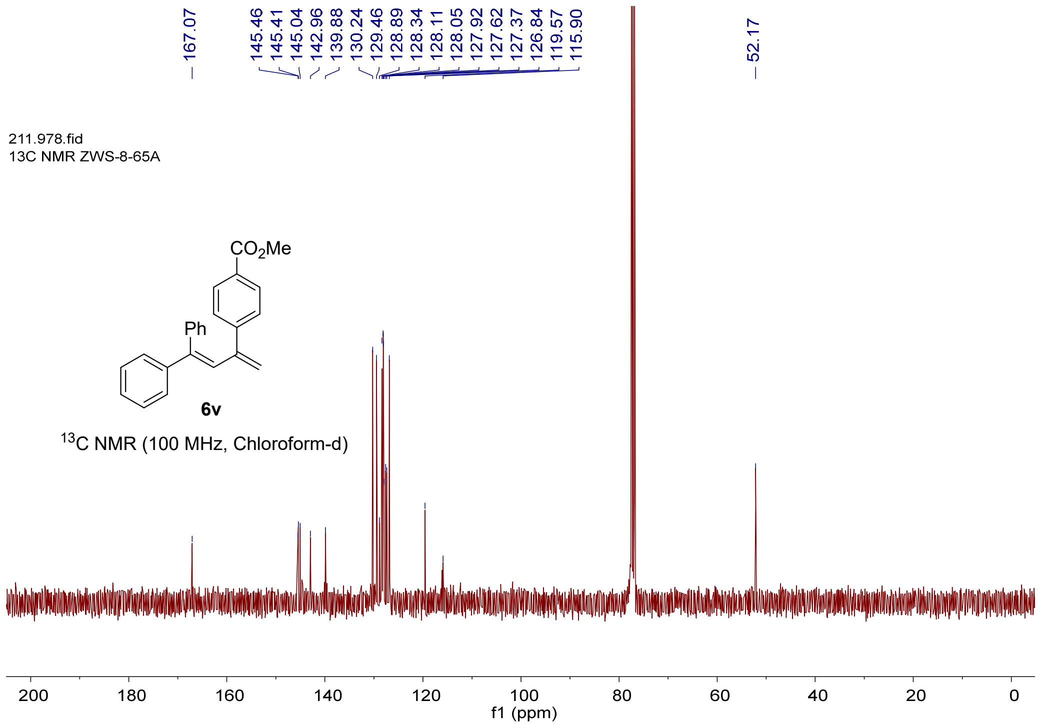

Supplementary Figure 130. ^{13}**C NMR** of compound **6v**

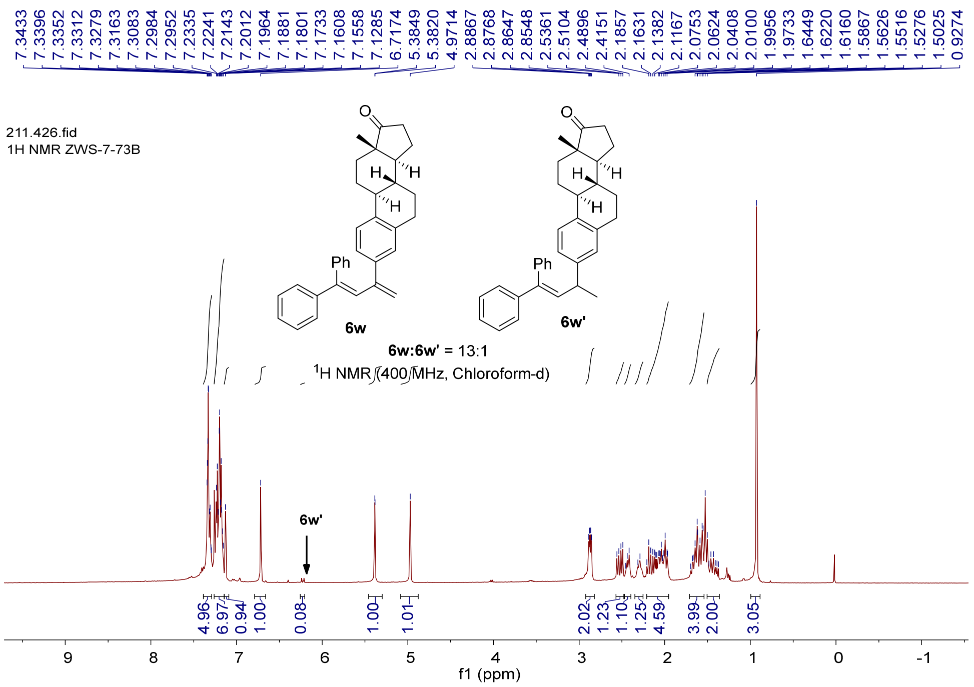

Supplementary Figure 131. **¹H NMR** of compound **6w** and **6w'** (**6w/6w'** = 13:1)

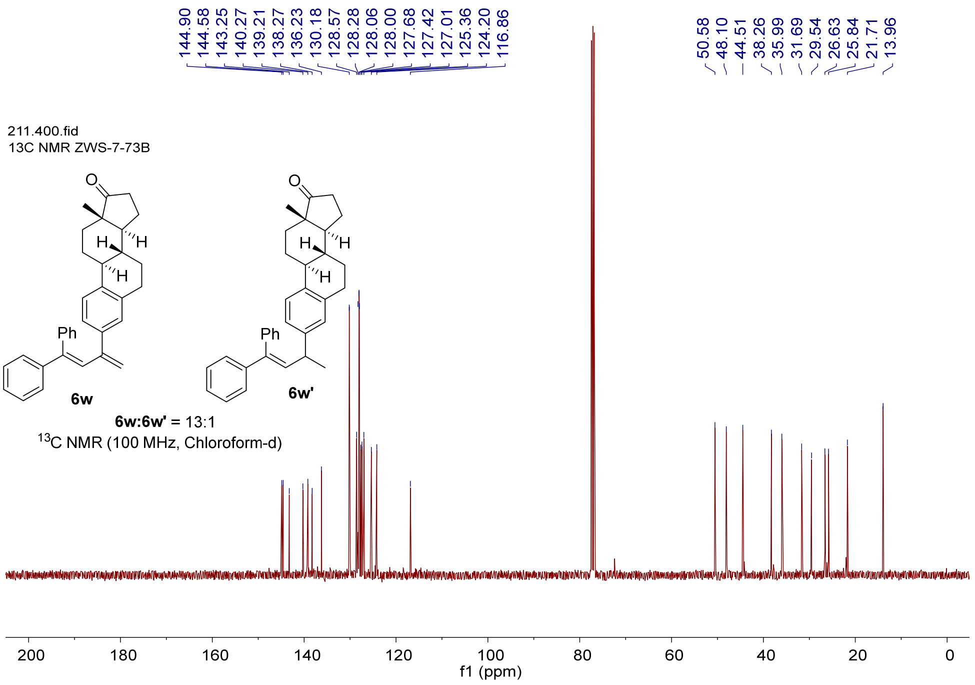

Supplementary Figure 132. **¹³C NMR** of compound **6w** and **6w'** (**6w/6w'** = 13:1)

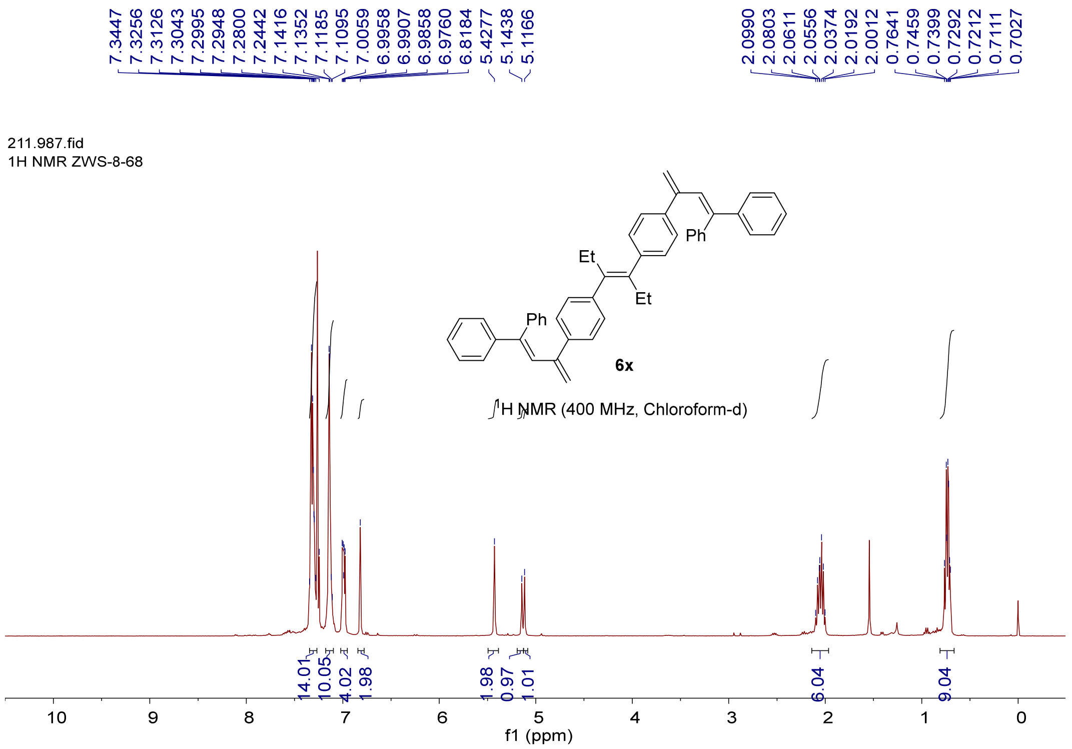

Supplementary Figure 133. ¹H NMR of compound **6x**

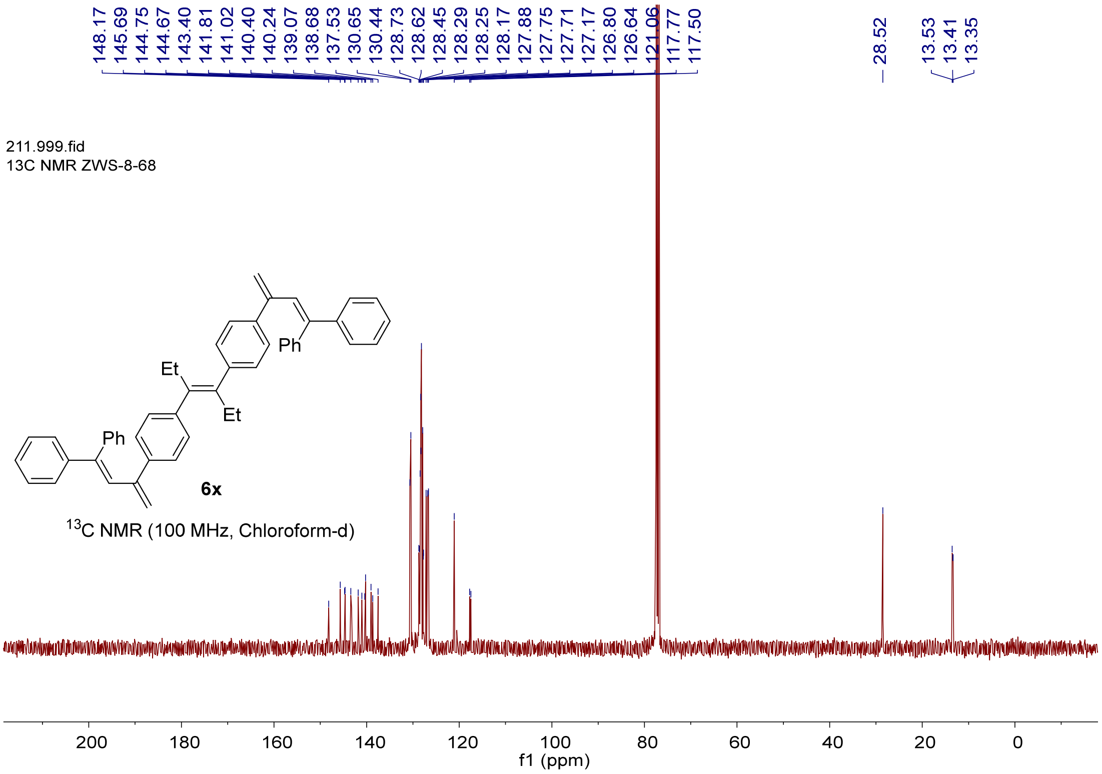

Supplementary Figure 134. ¹³C NMR of compound **6x**

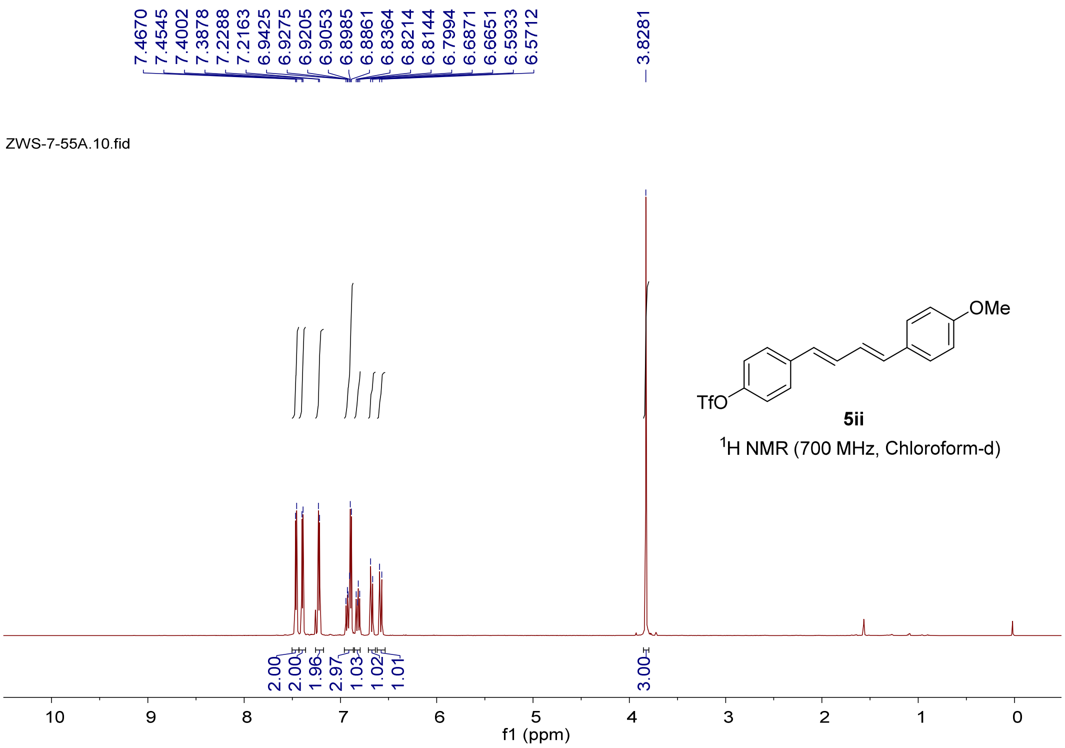

Supplementary Figure 135. ^{1}**H NMR** of compound **5ii**

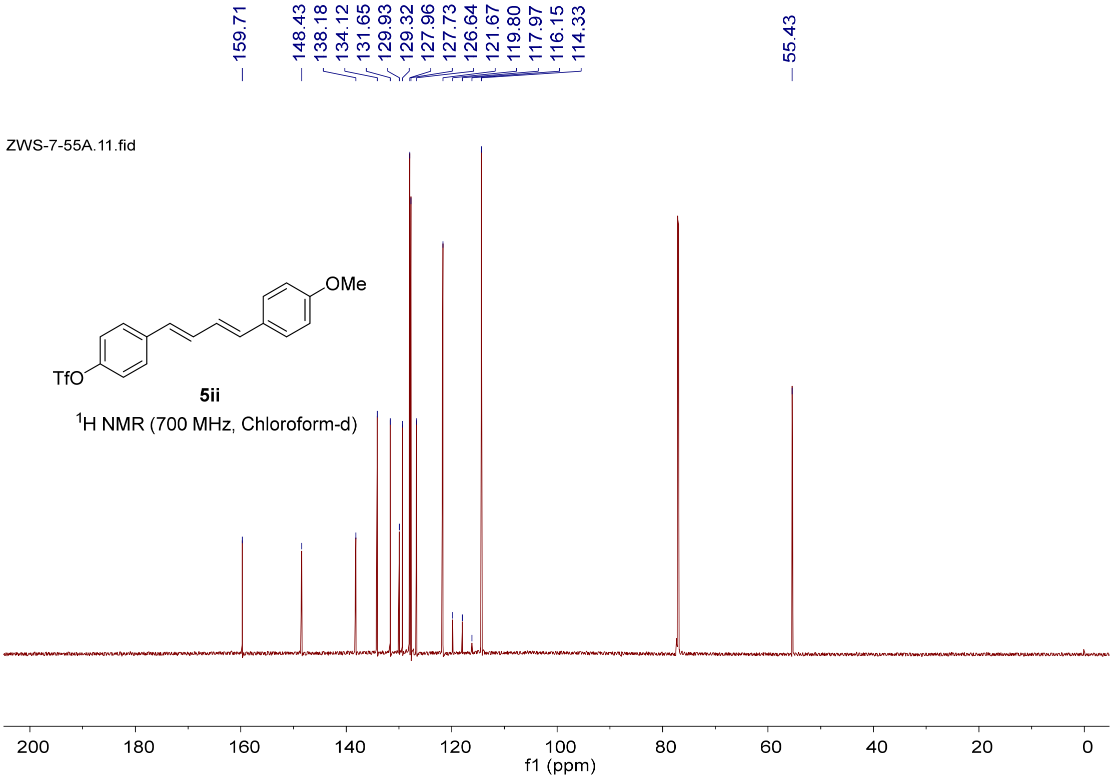

Supplementary Figure 136. ^{13}**C NMR** of compound **5ii**

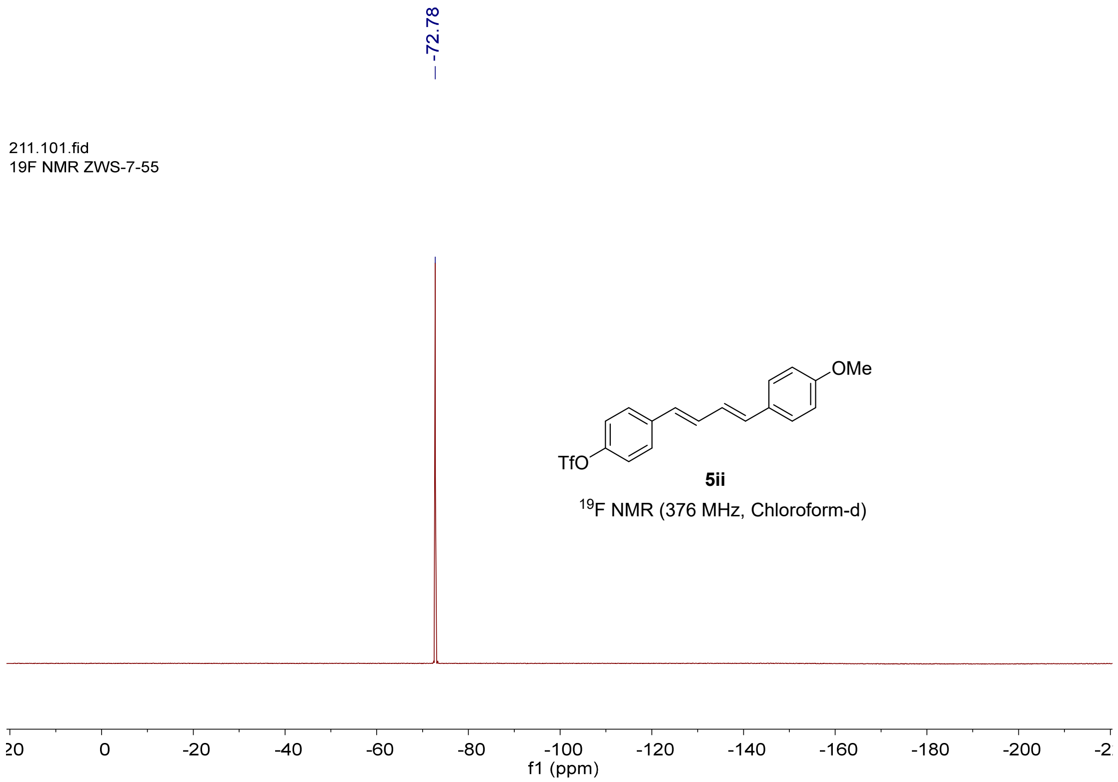

Supplementary Figure 137. ^{19}F NMR of compound **5ii**

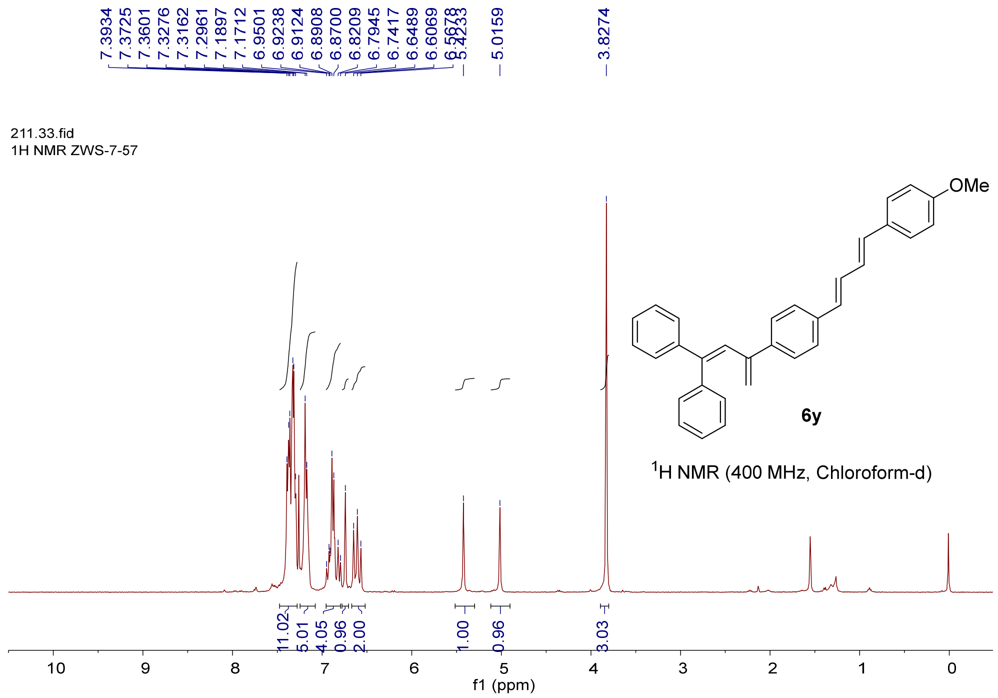

Supplementary Figure 138. ^{1}**H NMR** of compound **6y**

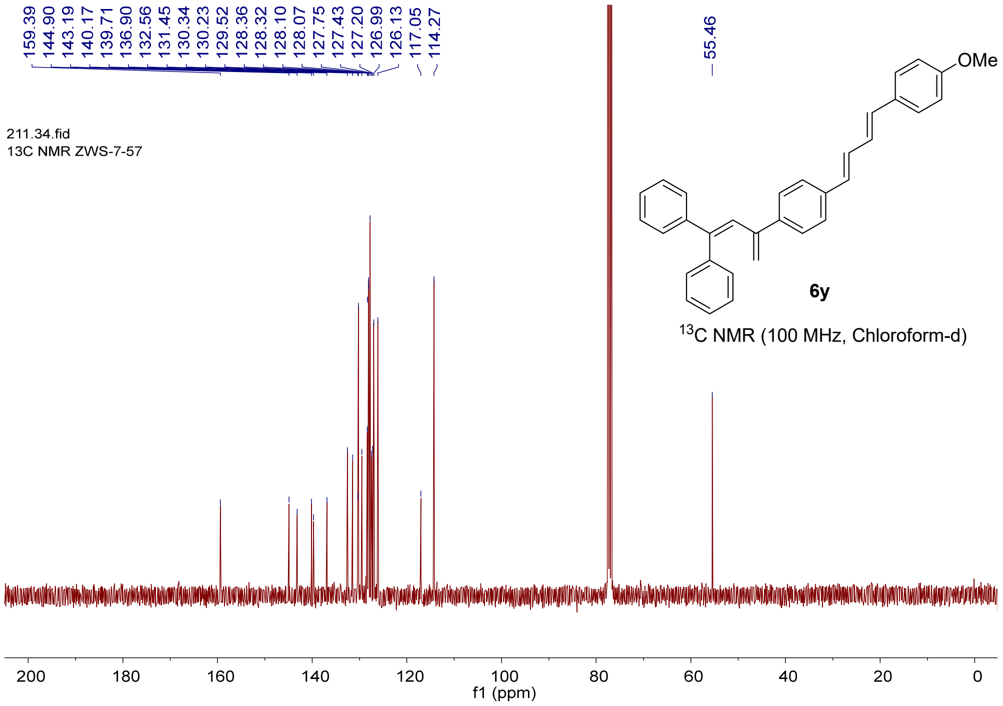

Supplementary Figure 139. ^{13}**C NMR** of compound **6y**

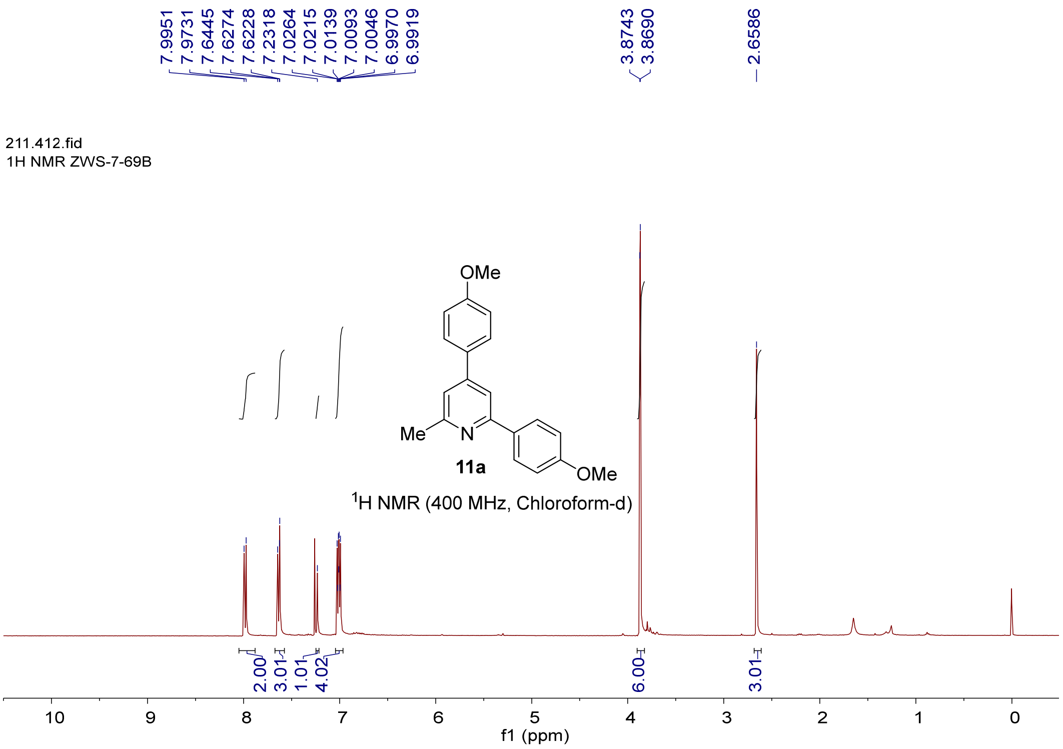

Supplementary Figure 140. ^{1}**H NMR** of compound **11a**

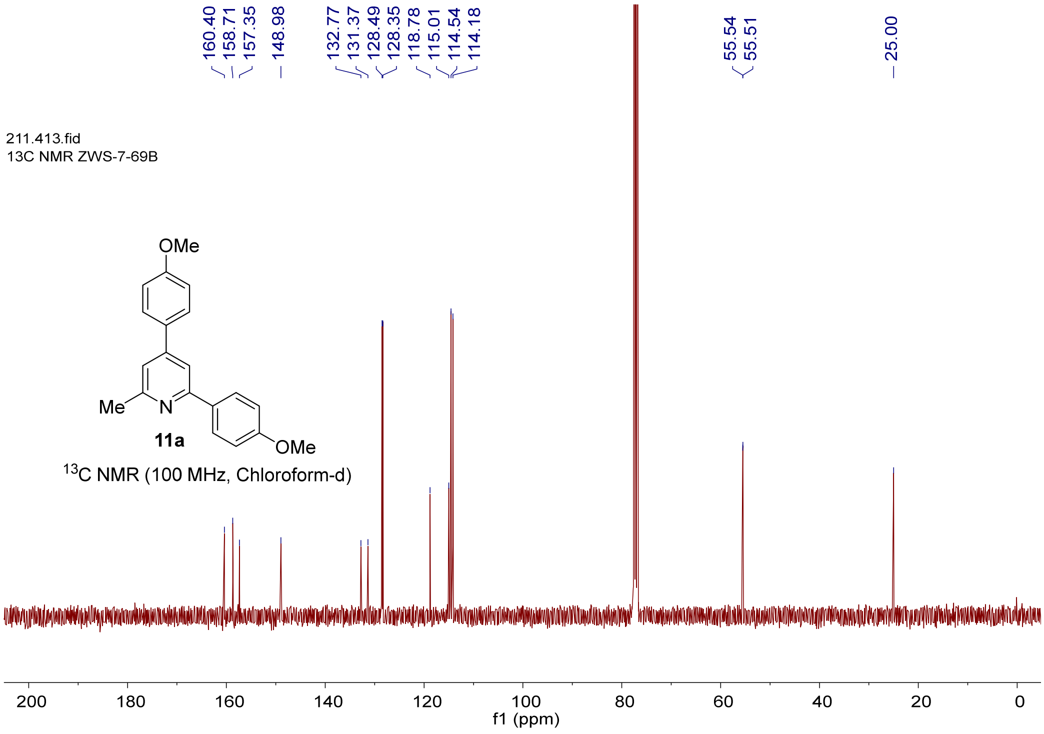

Supplementary Figure 141. ^{13}**C NMR** of compound **11a**

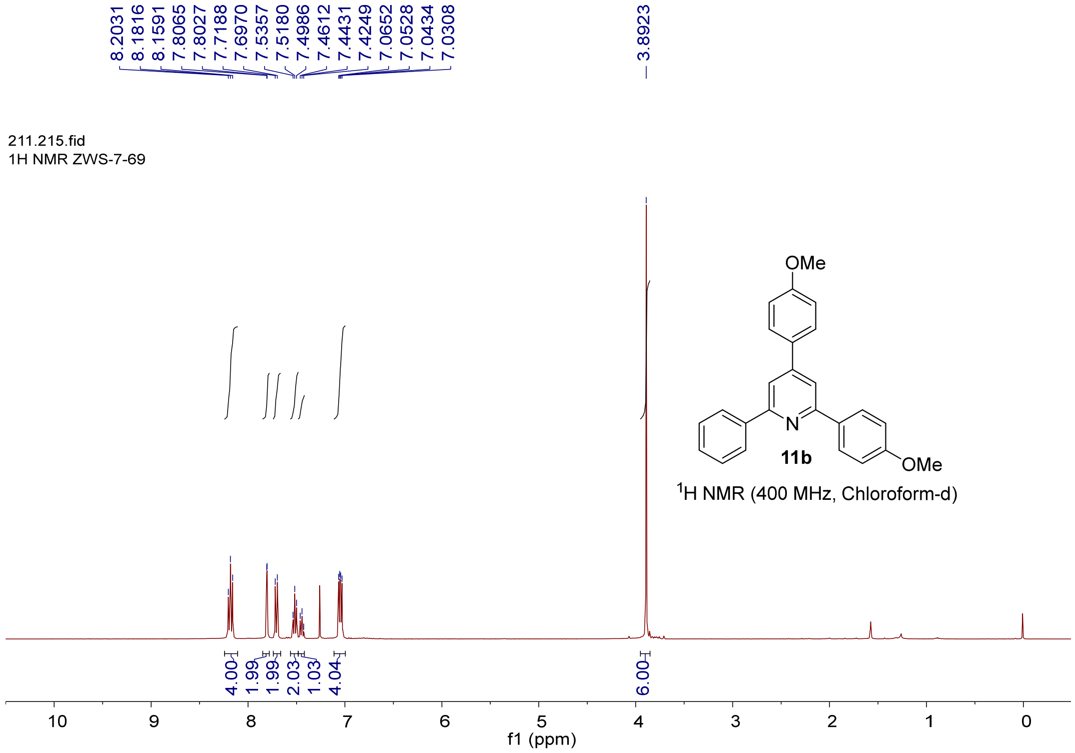

Supplementary Figure 142. **^{1}H NMR** of compound **11b**

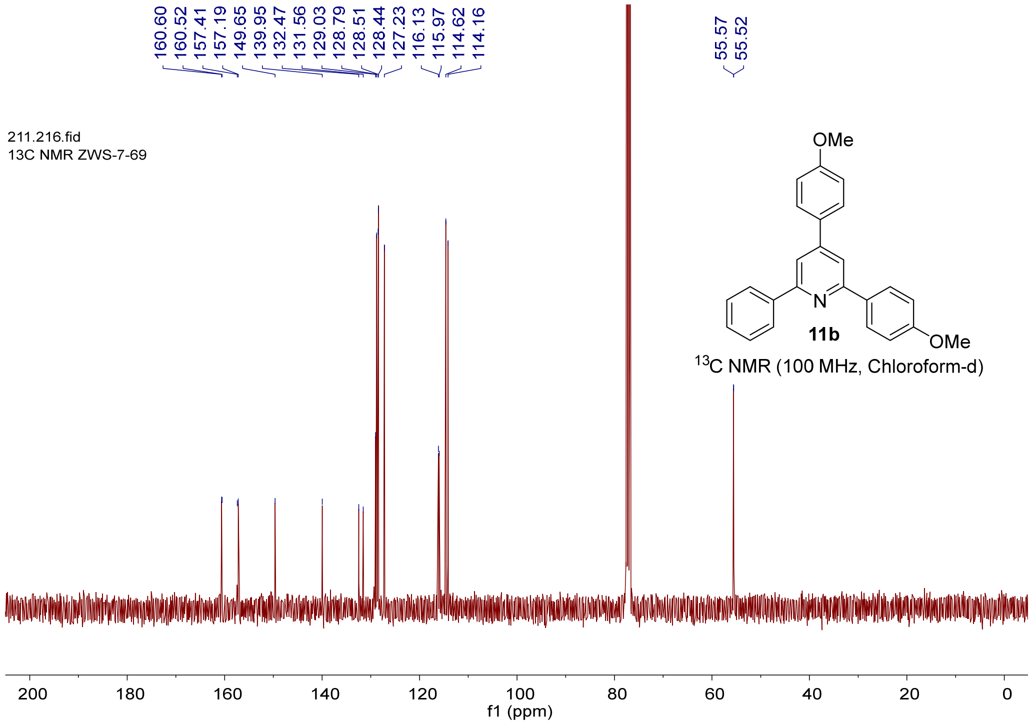

Supplementary Figure 143. **^{13}C NMR** of compound **11b**

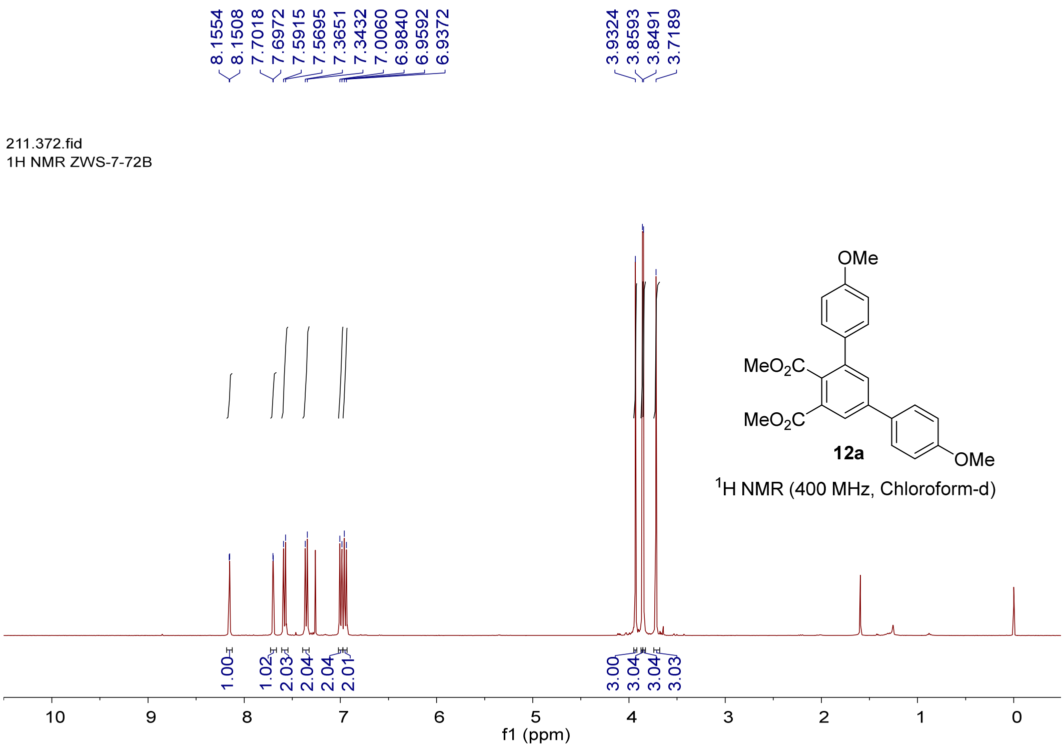

Supplementary Figure 144. **^{1}H NMR** of compound **12a**

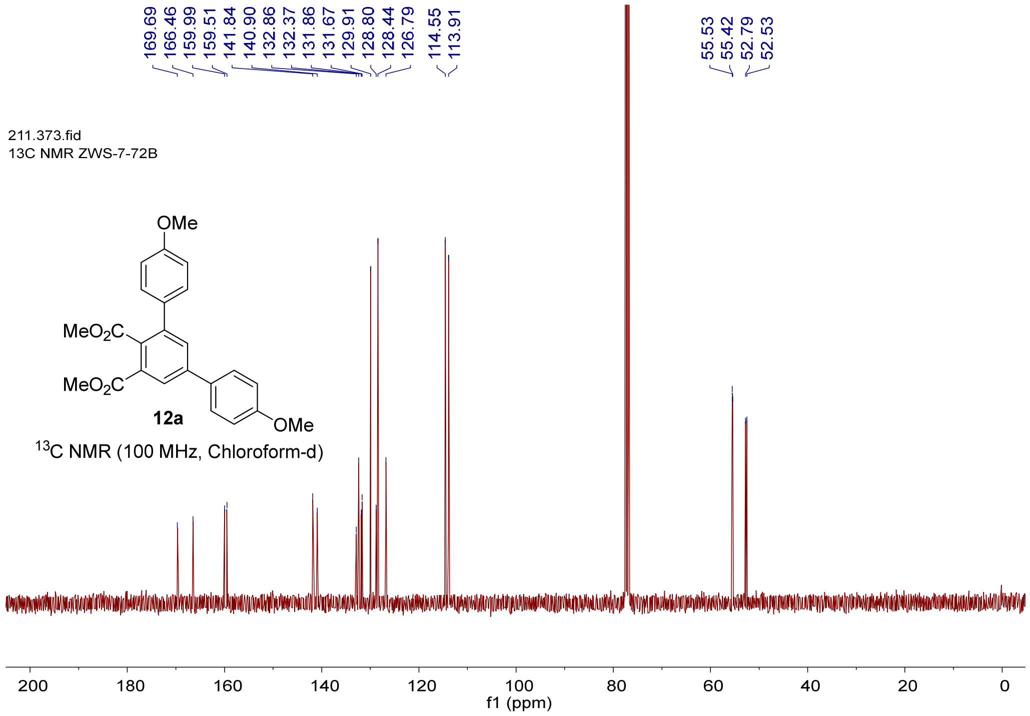

Supplementary Figure 145. **^{13}C NMR** of compound **12a**

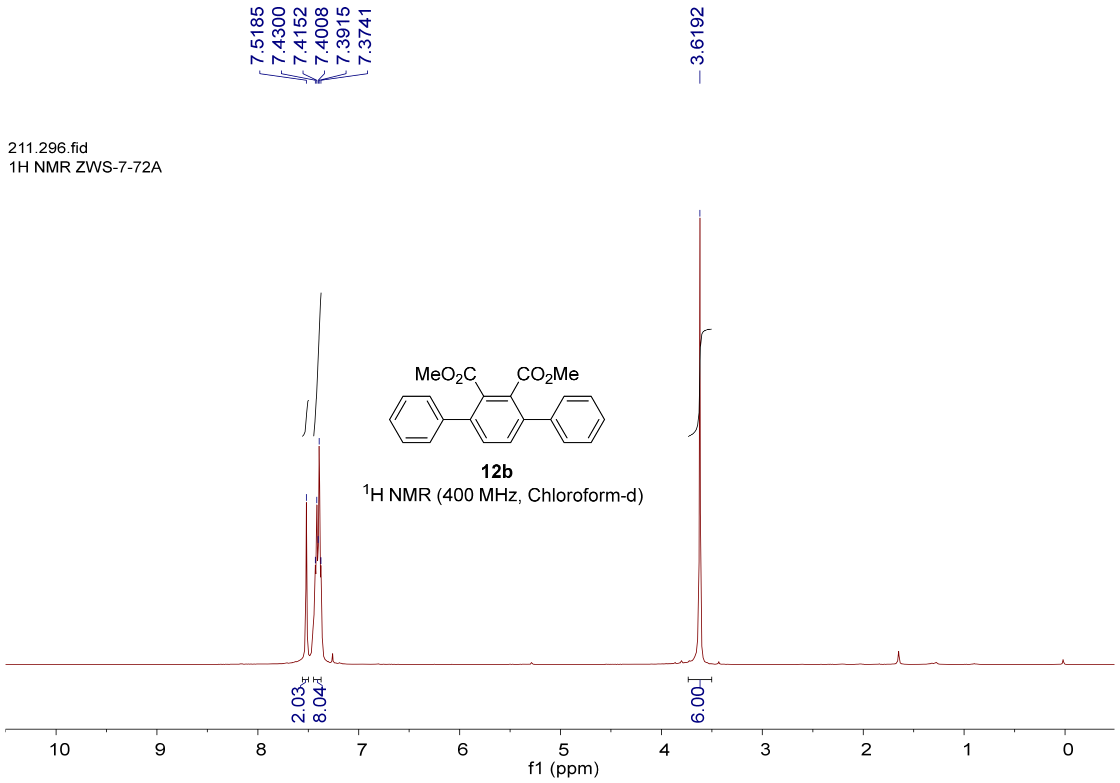

Supplementary Figure 146. **^{1}H NMR** of compound **12b**

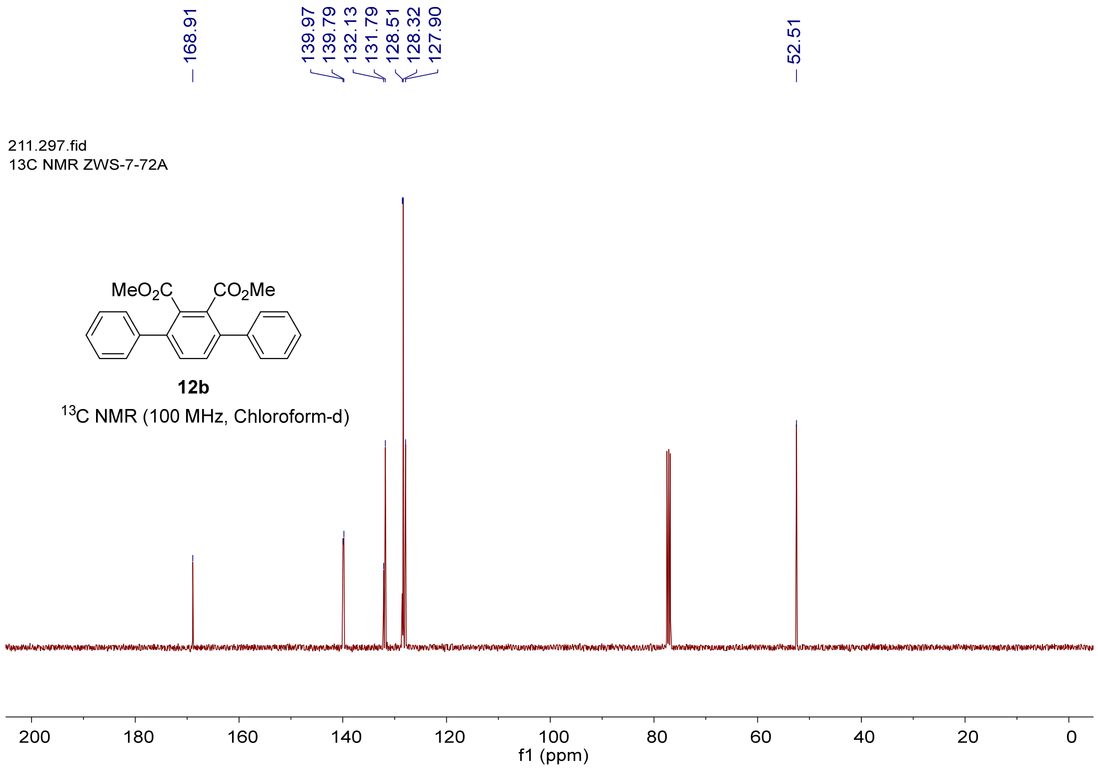

Supplementary Figure 147. **^{13}C NMR** of compound **12b**

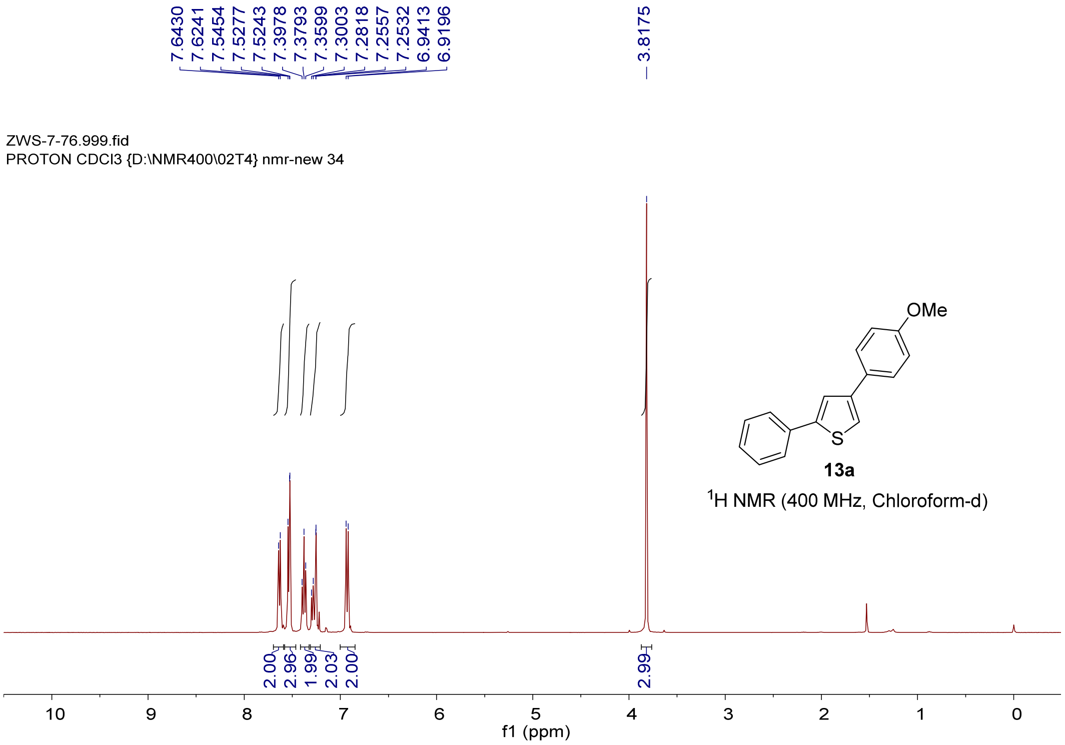

Supplementary Figure 148. ^{1}**H NMR** of compound **13a**

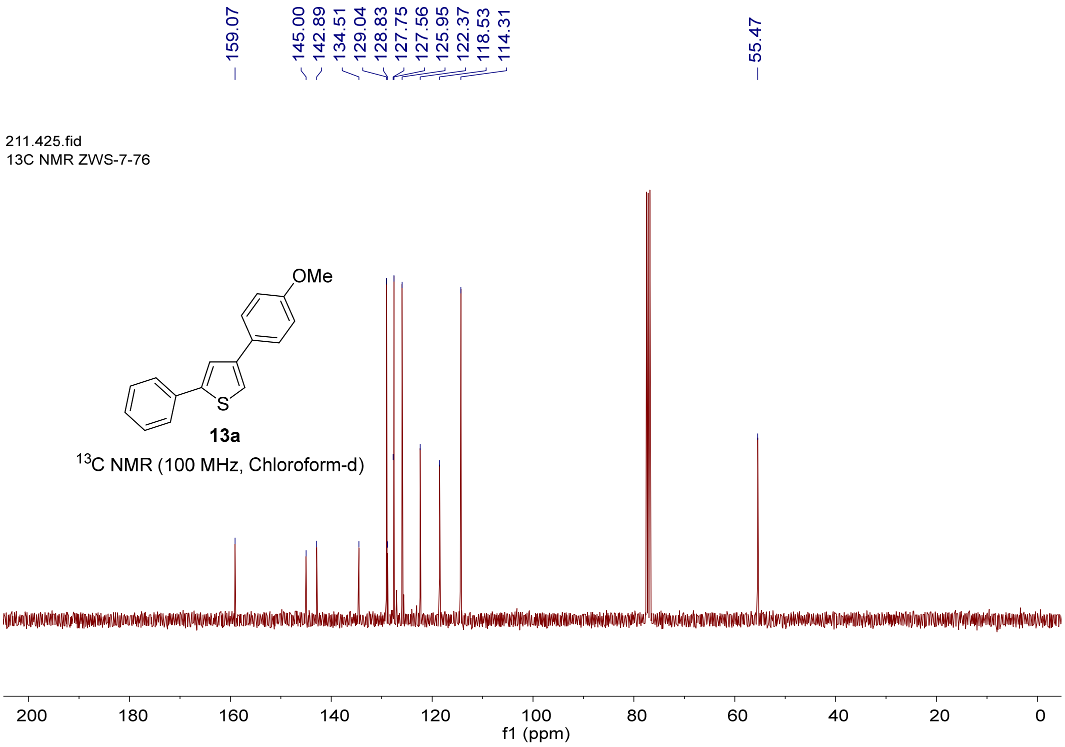

Supplementary Figure 149. ^{13}**C NMR** of compound **13a**

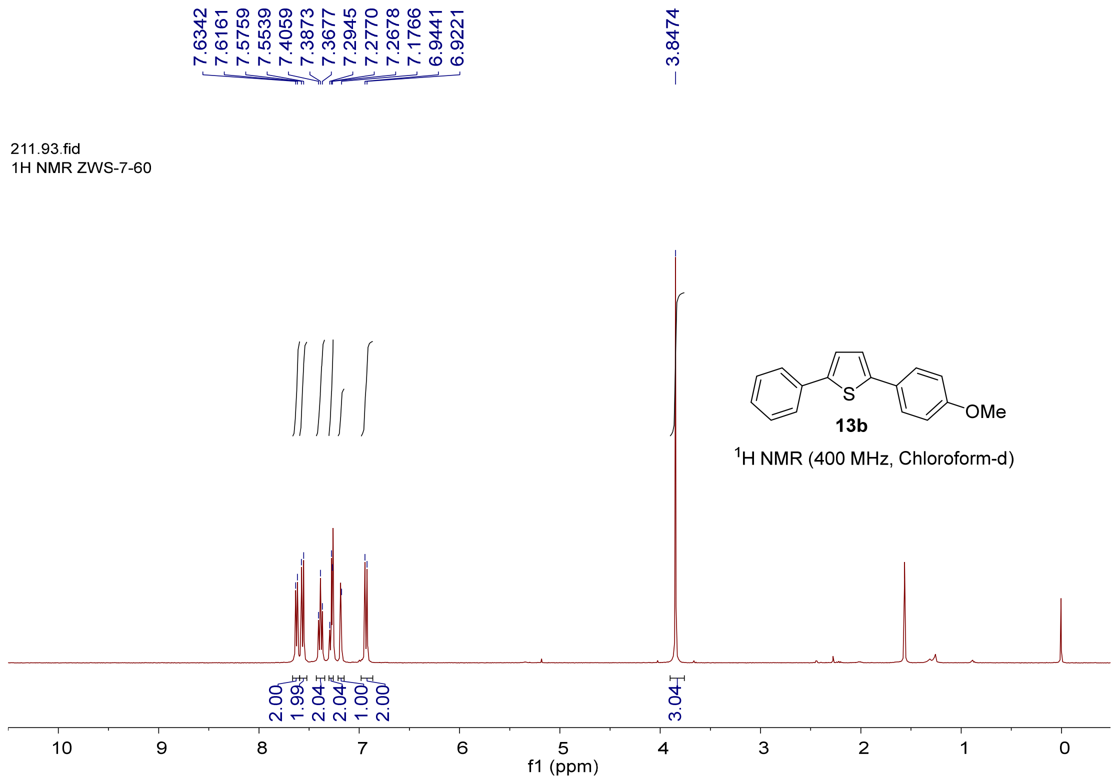

Supplementary Figure 150. **^{1}H NMR** of compound **13b**

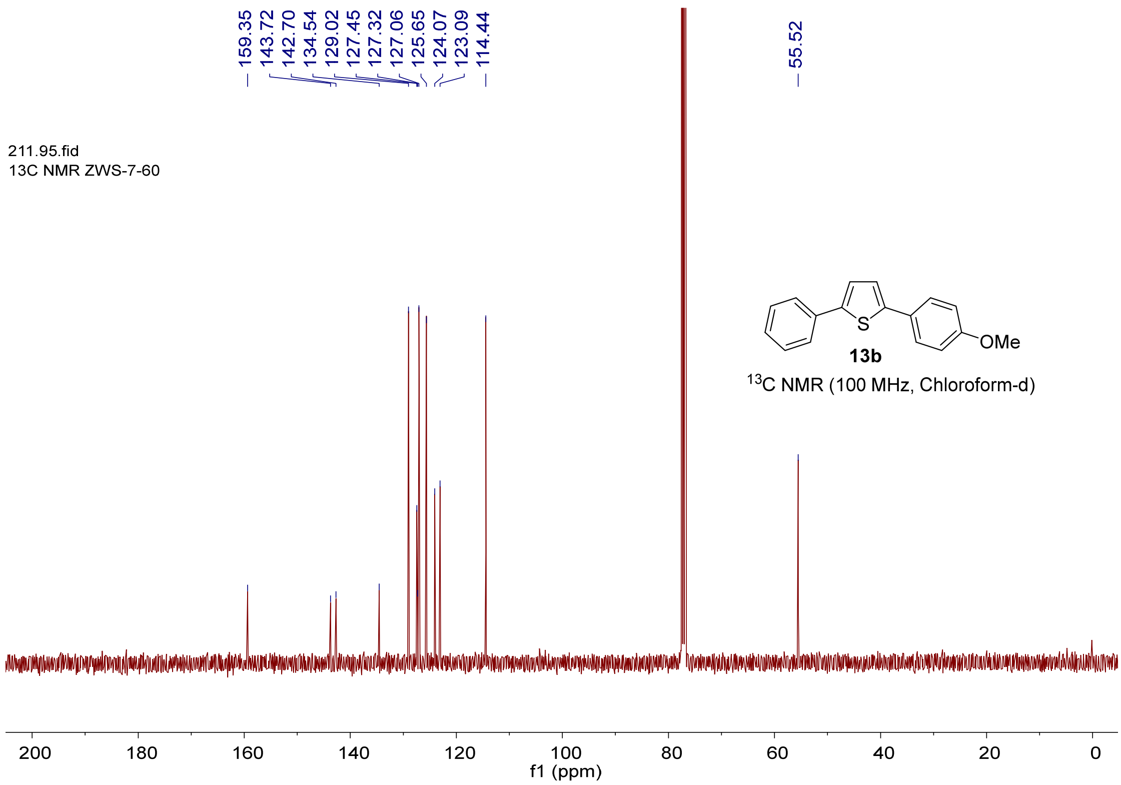

Supplementary Figure 151. **^{13}C NMR** of compound **13b**

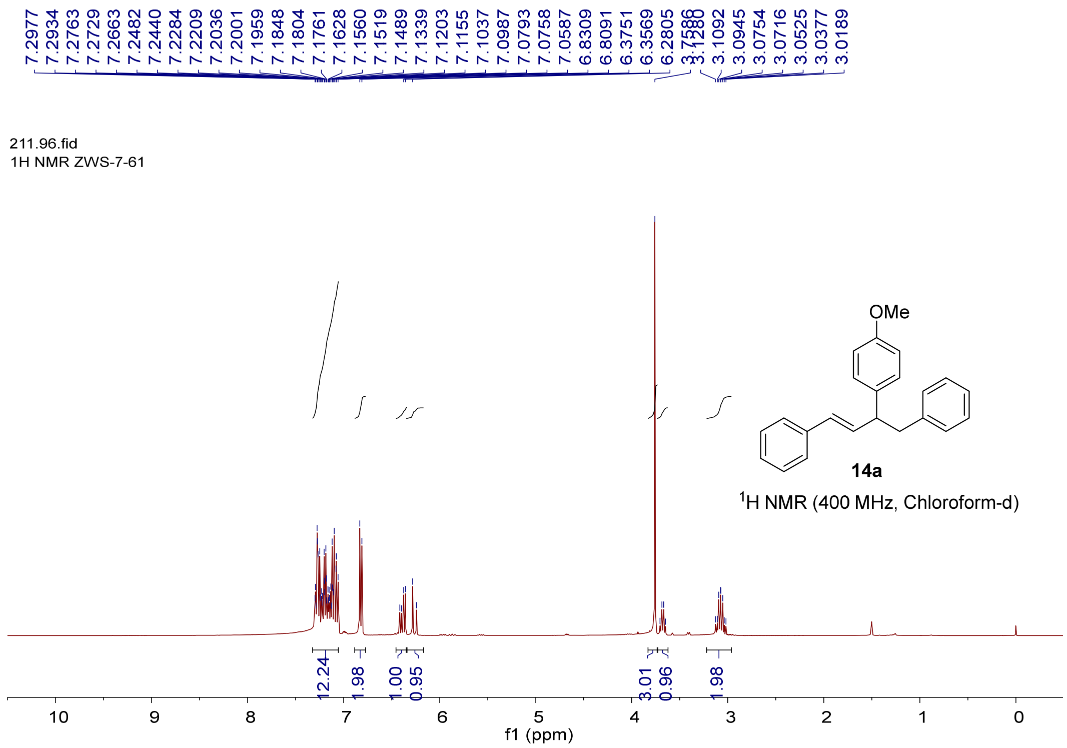

Supplementary Figure 152. ^{1}**H NMR** of compound **14a**

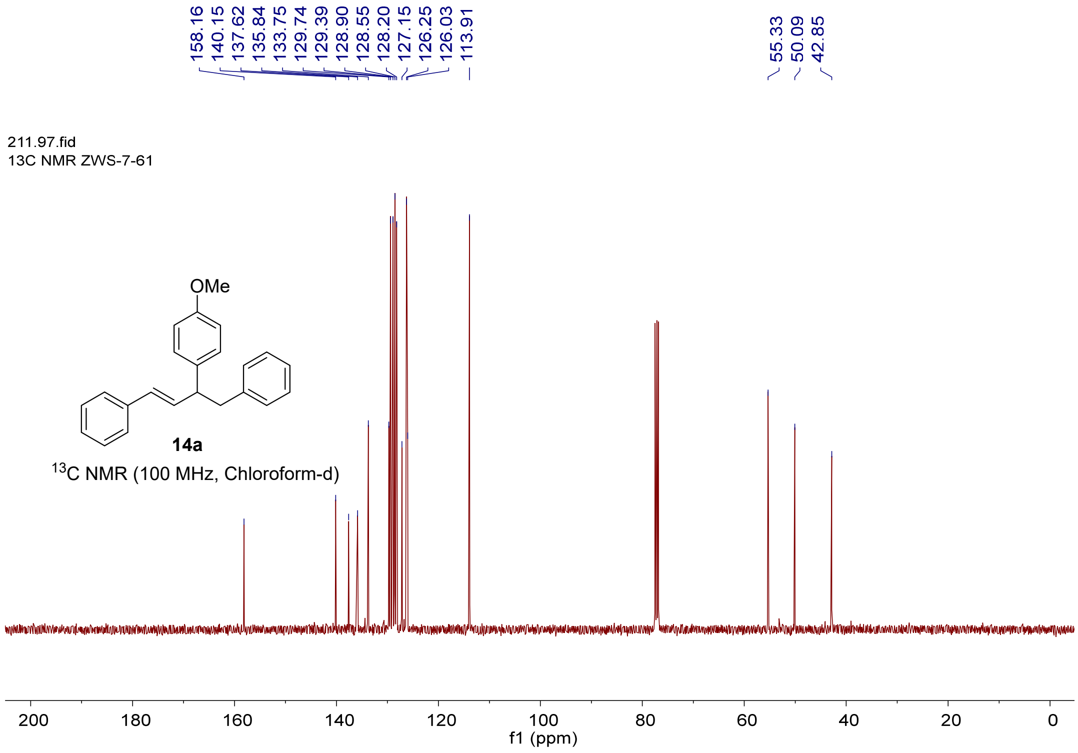

Supplementary Figure 153. ^{13}**C NMR** of compound **14a**

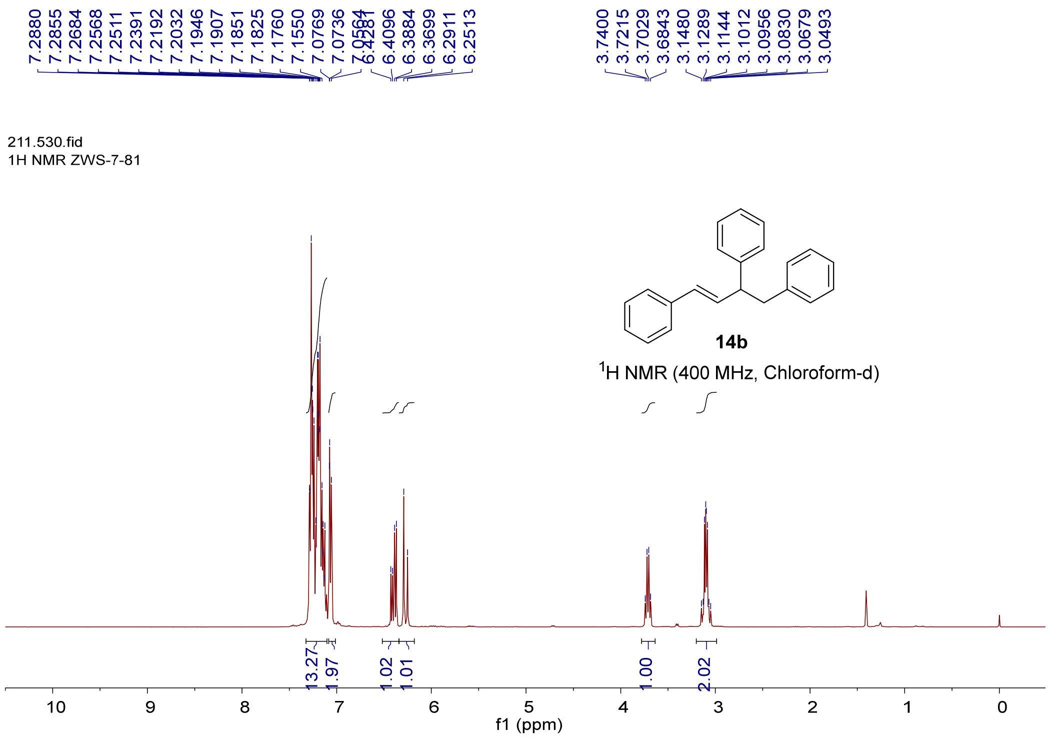

Supplementary Figure 154. ^{1}**H NMR** of compound **14b**

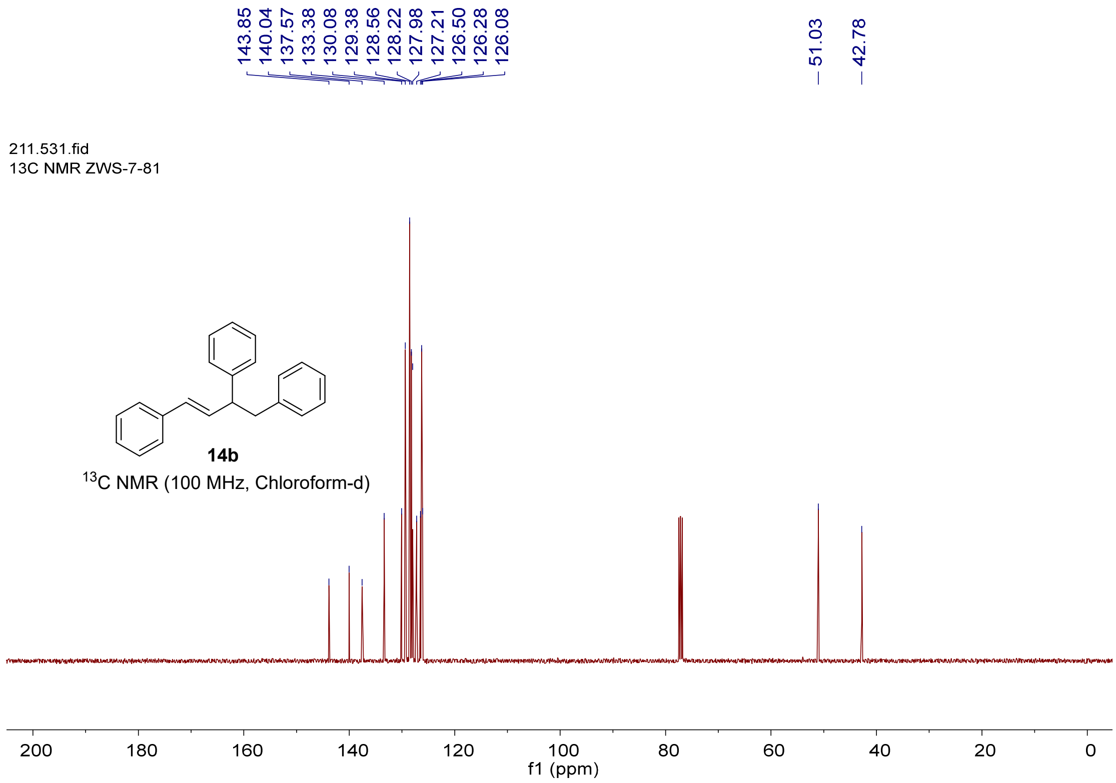

Supplementary Figure 155. ^{13}**C NMR** of compound **14b**

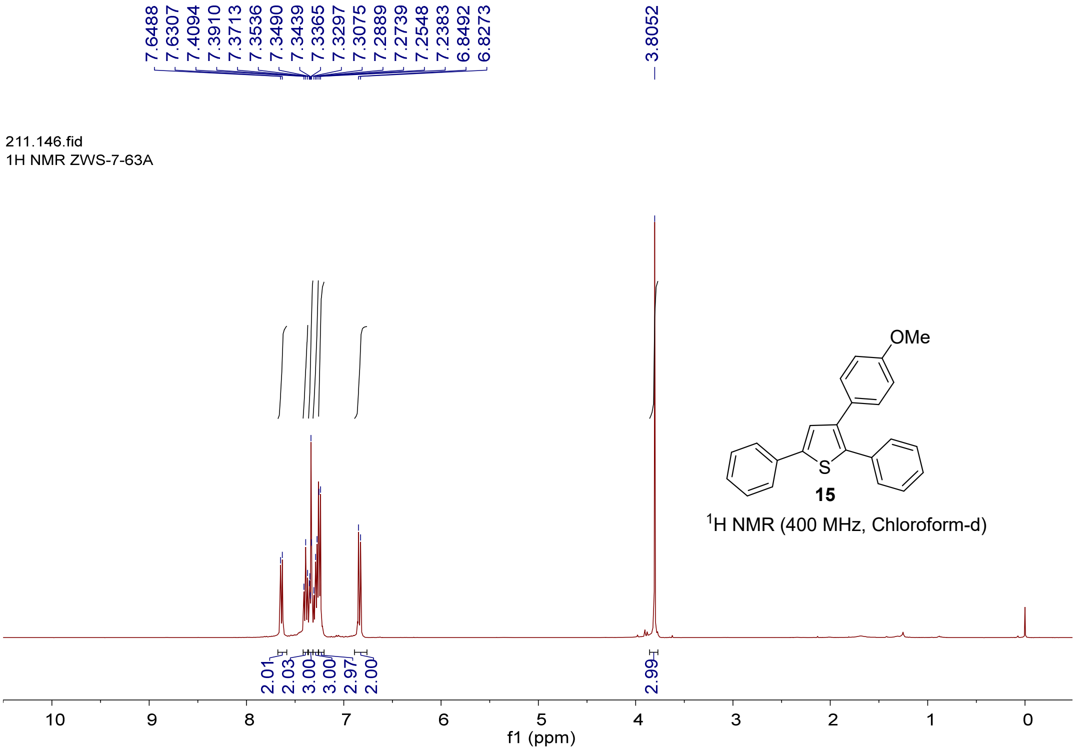

Supplementary Figure 156. ¹H NMR of compound **15**

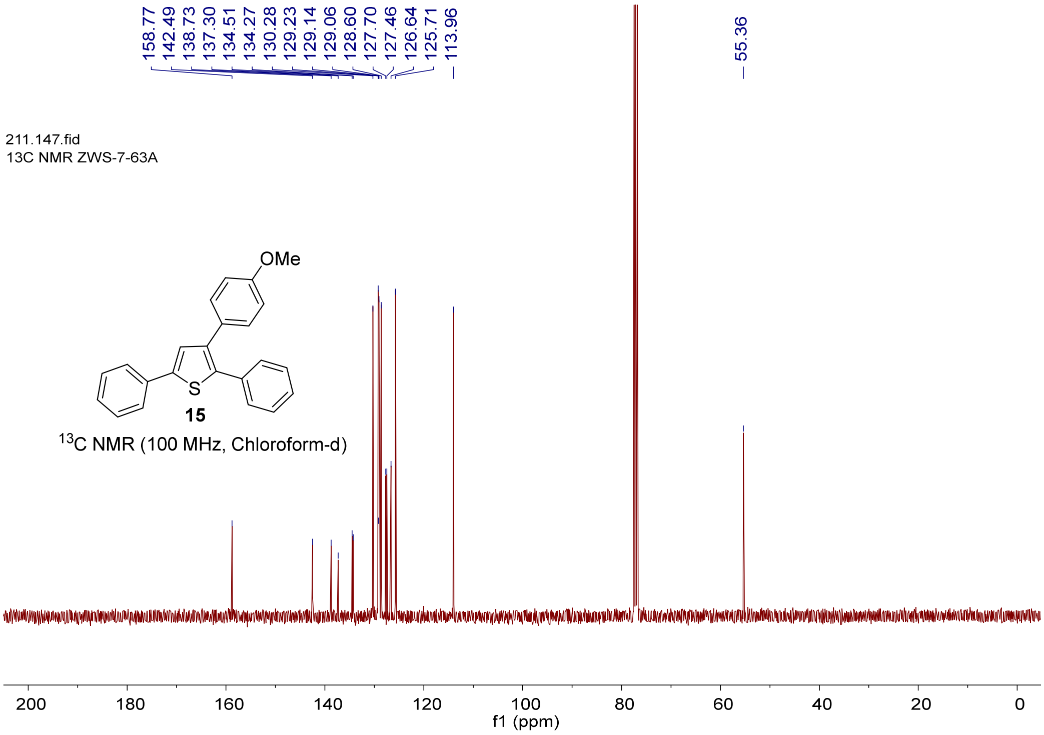

Supplementary Figure 157. ¹³C NMR of compound **15**

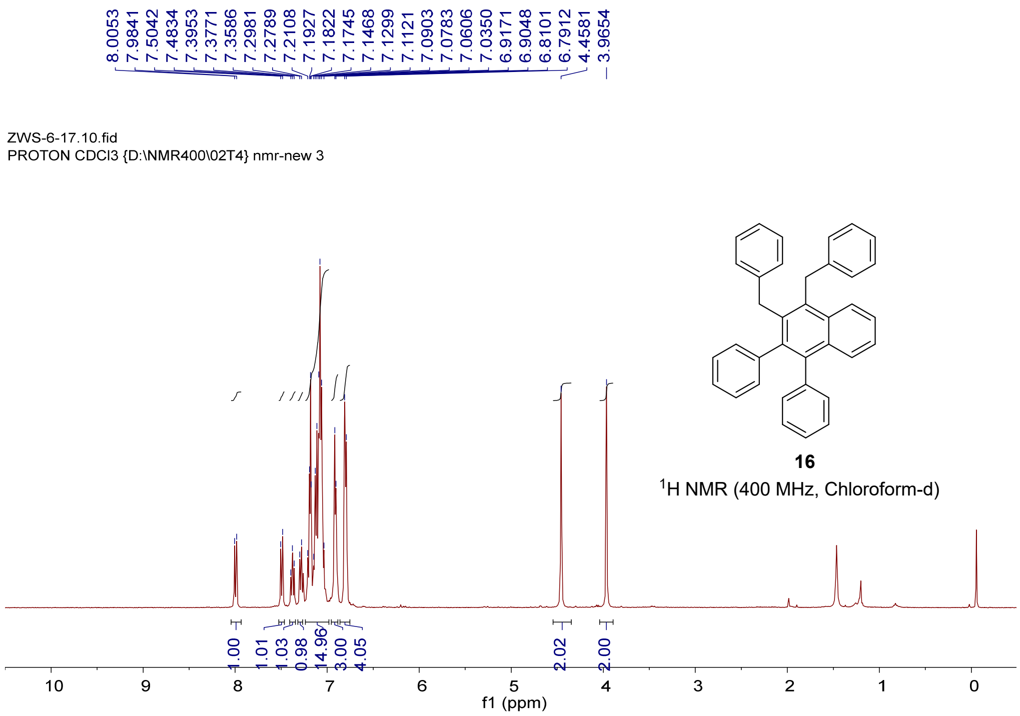

Supplementary Figure 158. **^{1}H NMR** of compound **16**

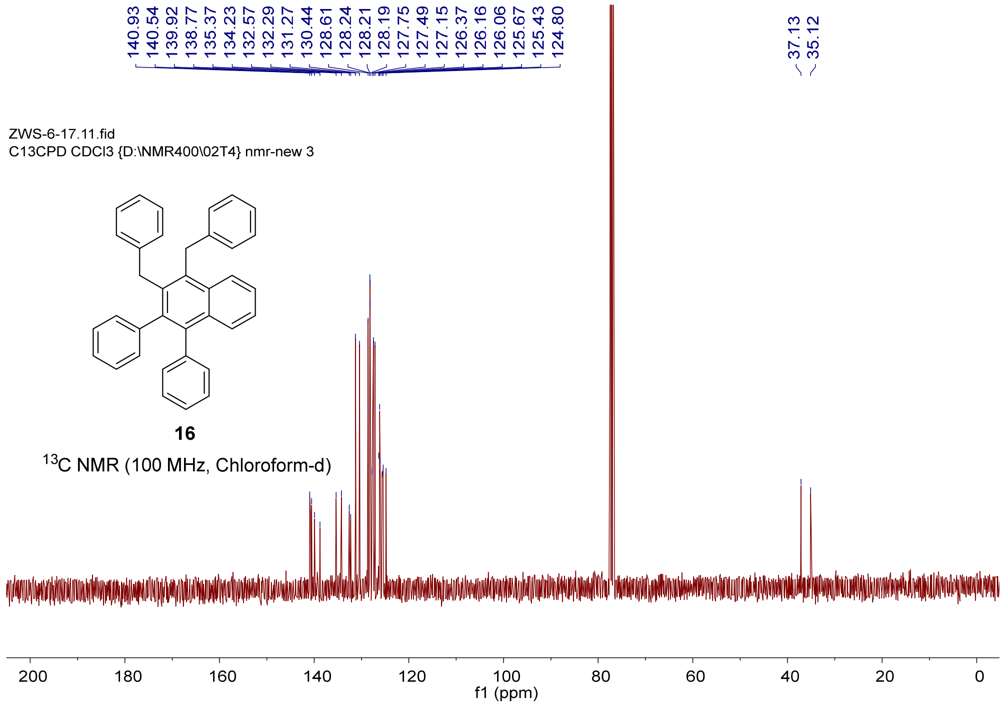

Supplementary Figure 159. **^{13}C NMR** of compound **16**

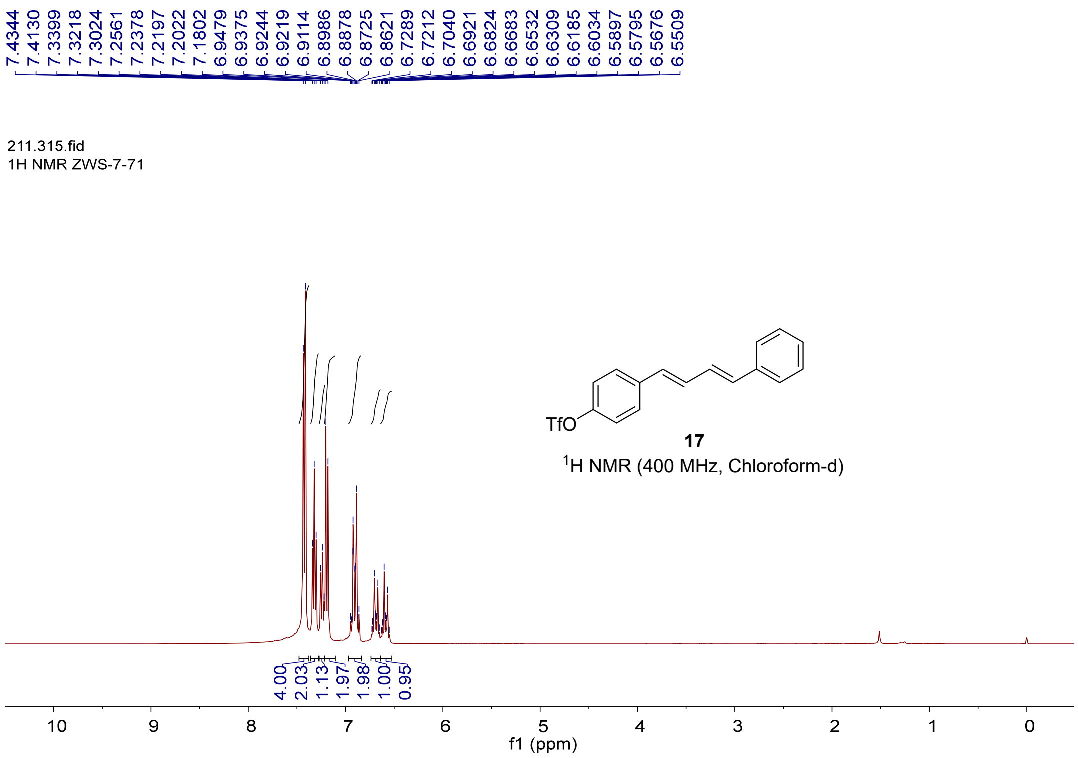

Supplementary Figure 160. ^{1}H NMR of compound **17**

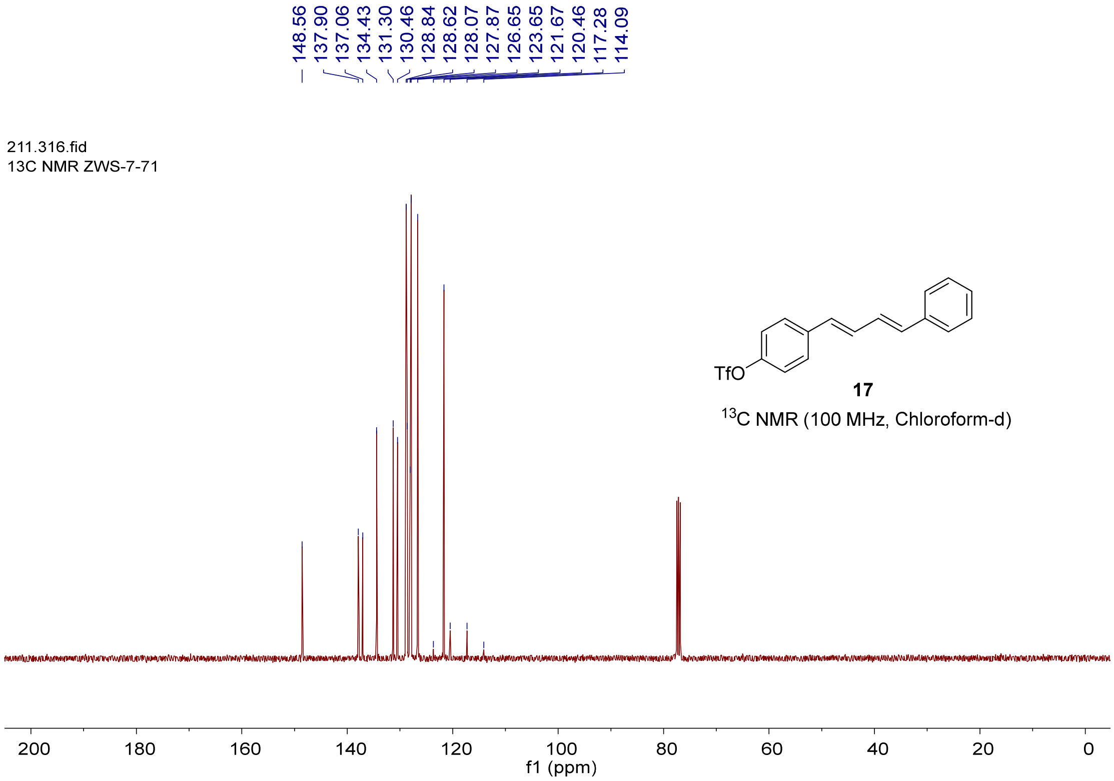

Supplementary Figure 161. ^{13}C NMR of compound **17**

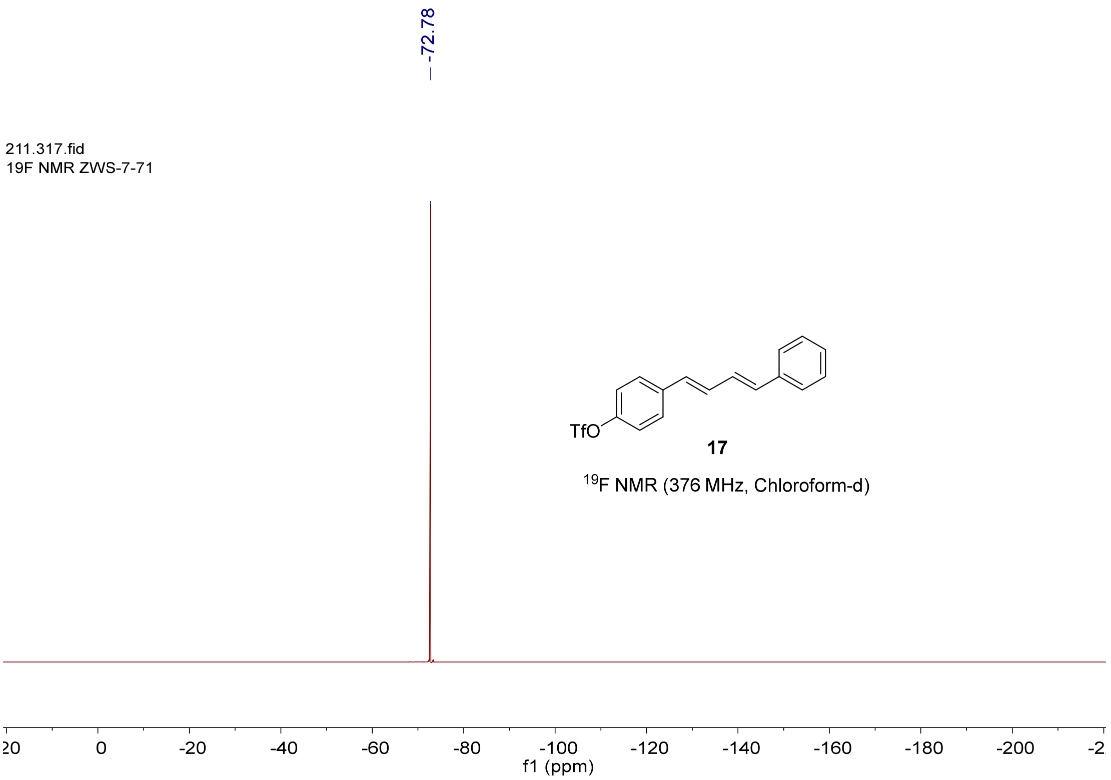

Supplementary Figure 162. **^{19}F NMR** of compound **17**

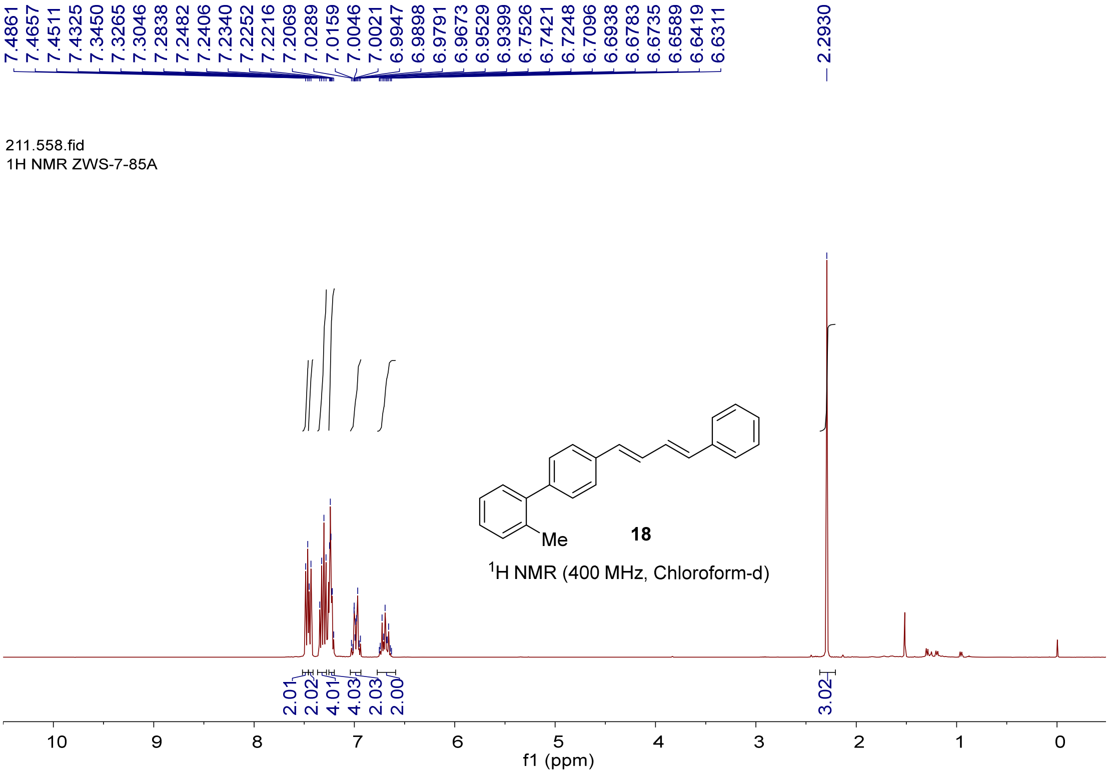

Supplementary Figure 163. ^{1}**H NMR** of compound **18**

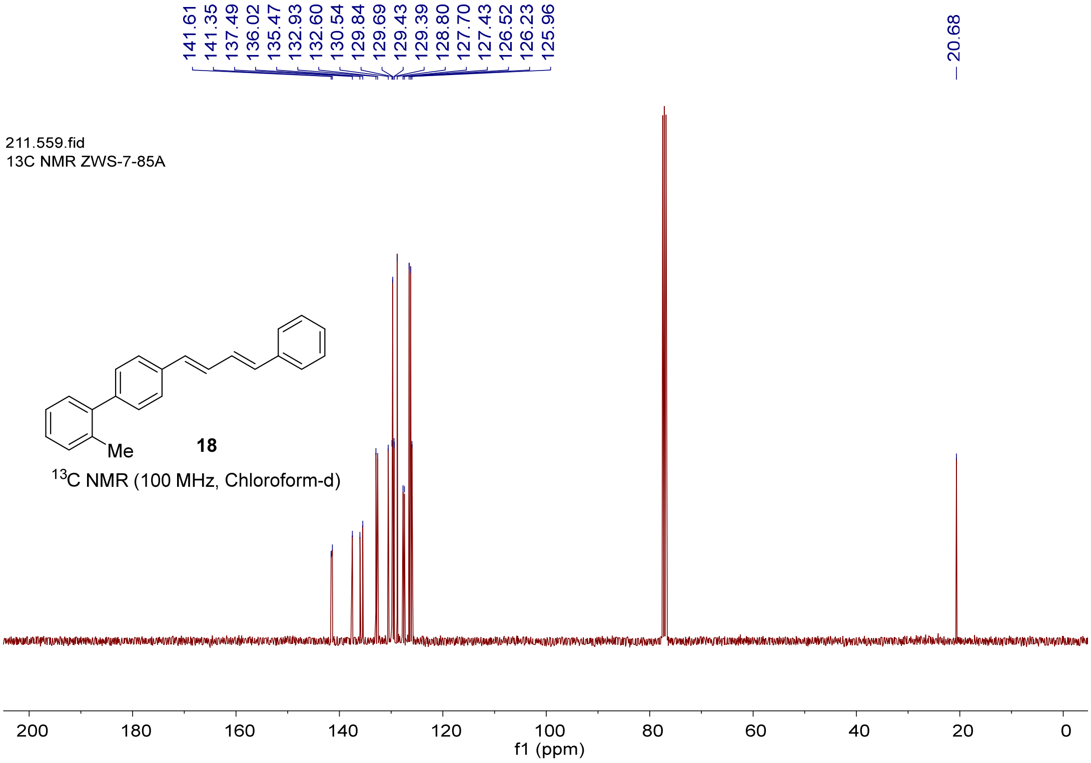

Supplementary Figure 164. ^{13}**C NMR** of compound **18**

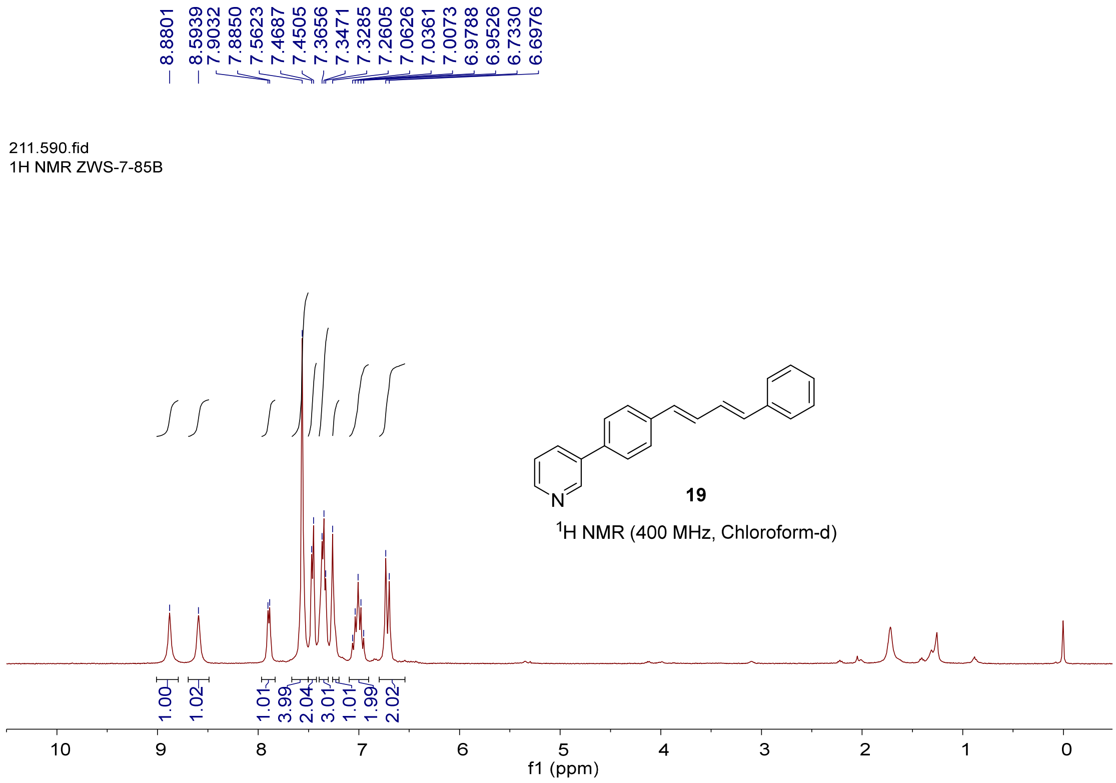

Supplementary Figure 165. ^{1}H NMR of compound **19**

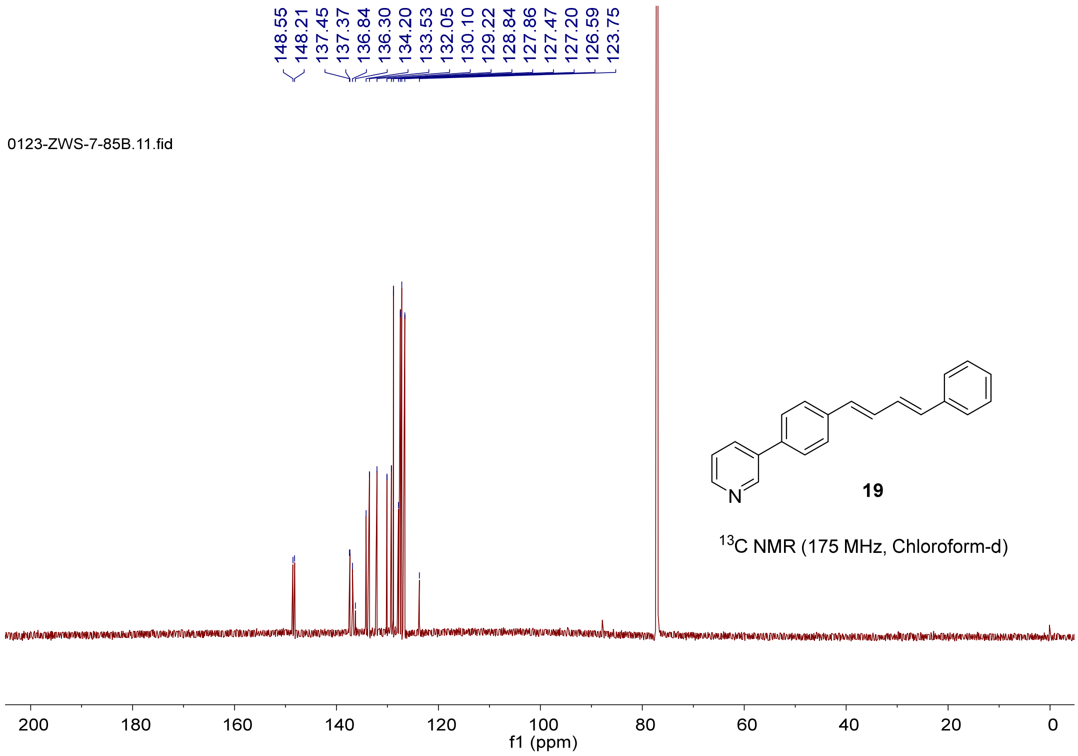

Supplementary Figure 166. ^{13}C NMR of compound **19**

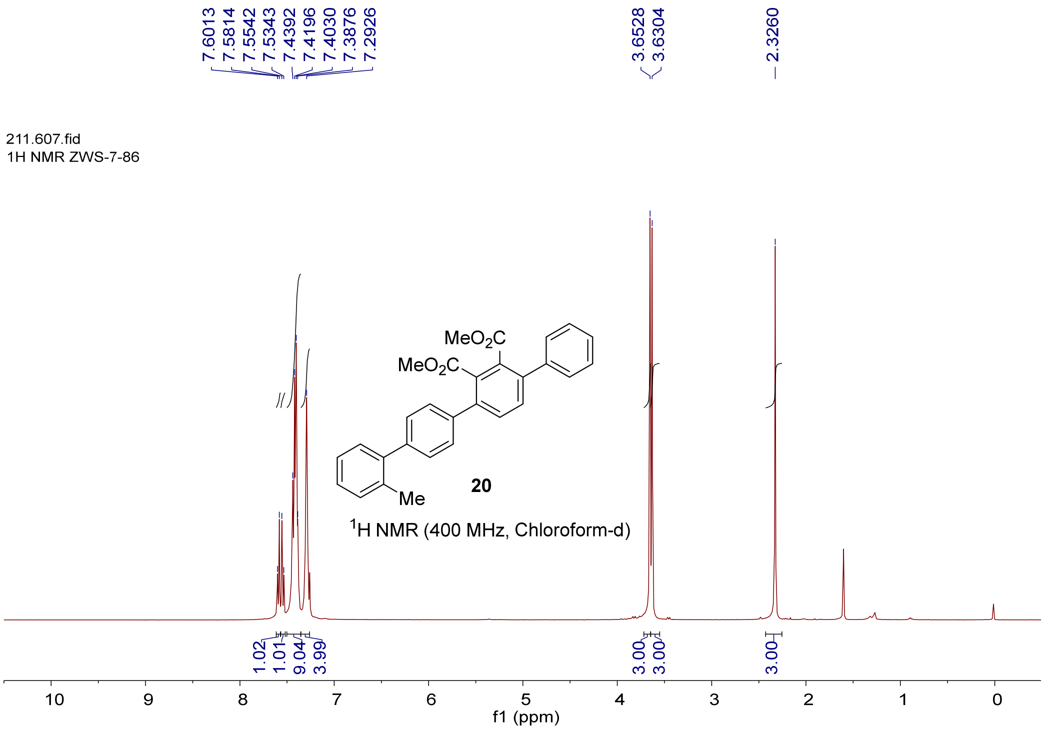

Supplementary Figure 167. ^{1}**H NMR** of compound **20**

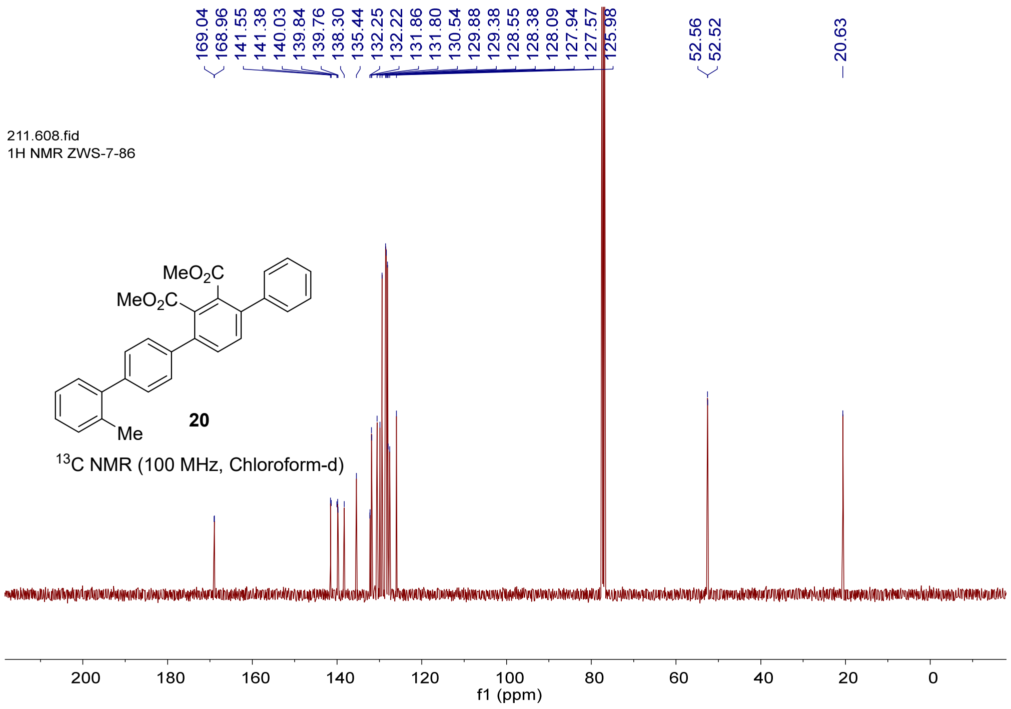

Supplementary Figure 168. ^{13}**C NMR** of compound **20**

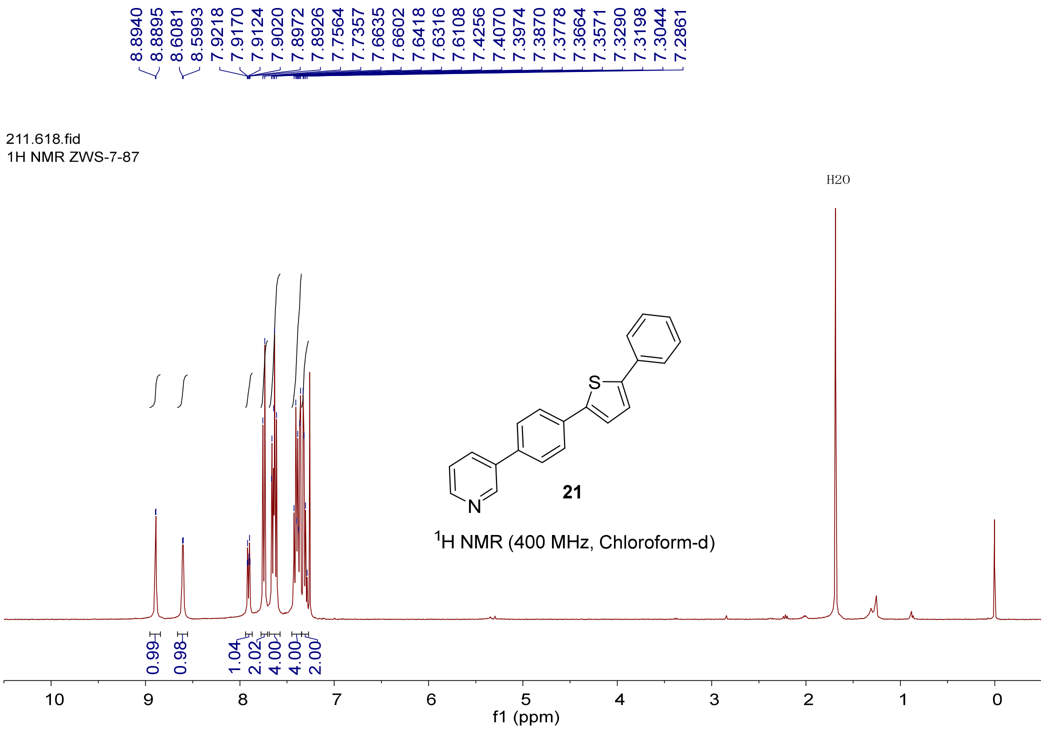

Supplementary Figure 169. ^{1}**H NMR** of compound **21**

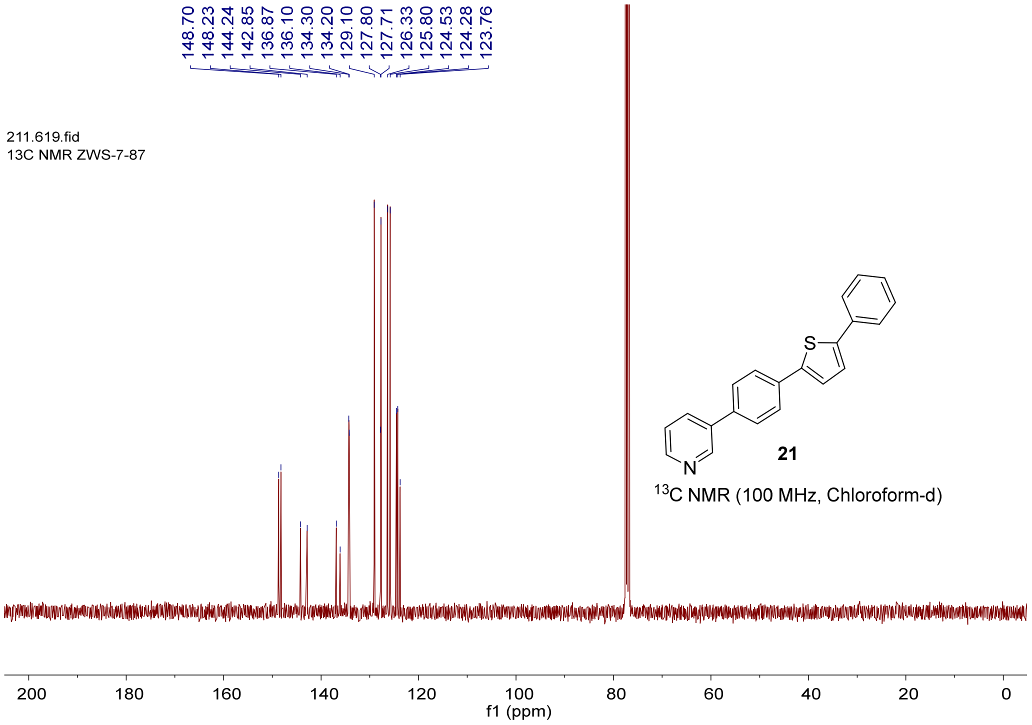

Supplementary Figure 170. ^{13}**C NMR** of compound **21**

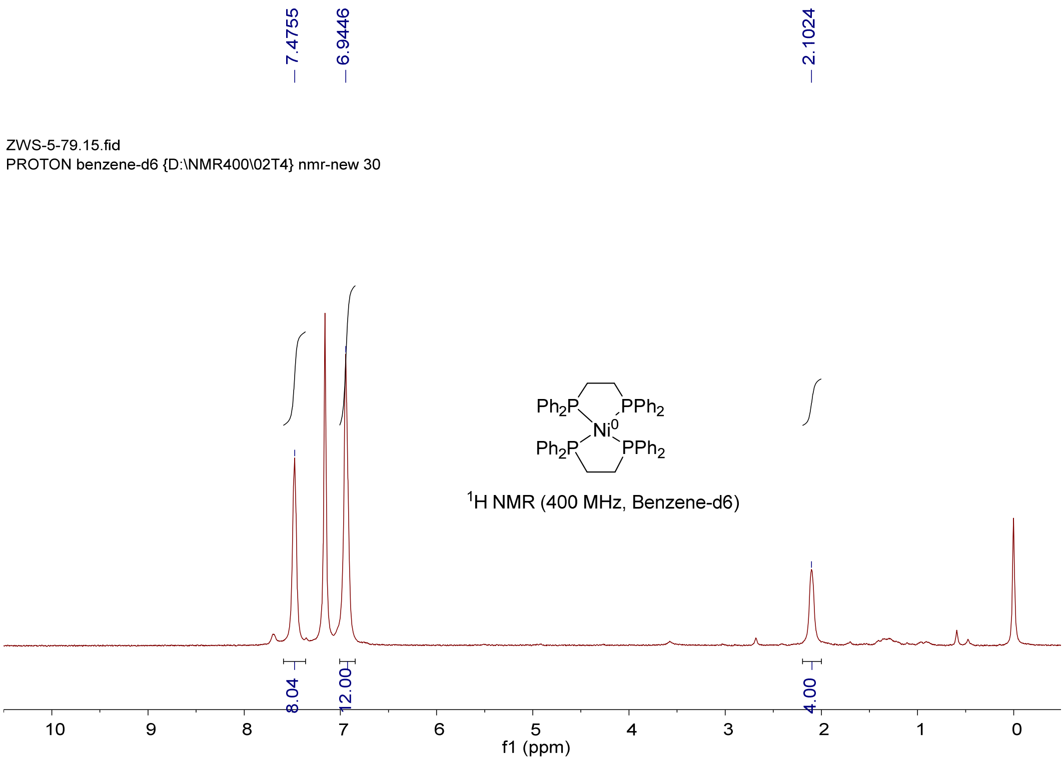

Supplementary Figure 171. ^{1}H NMR of Ni(dppe)$_2$

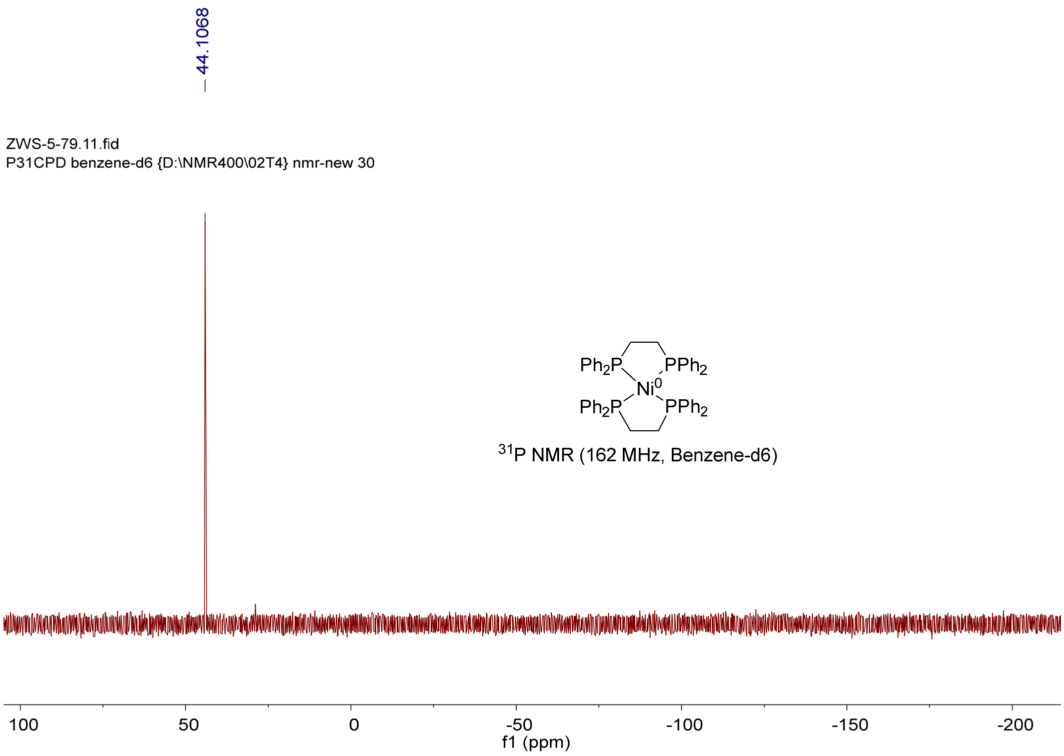

Supplementary Figure 172. ^{31}P NMR of Ni(dppe)$_2$

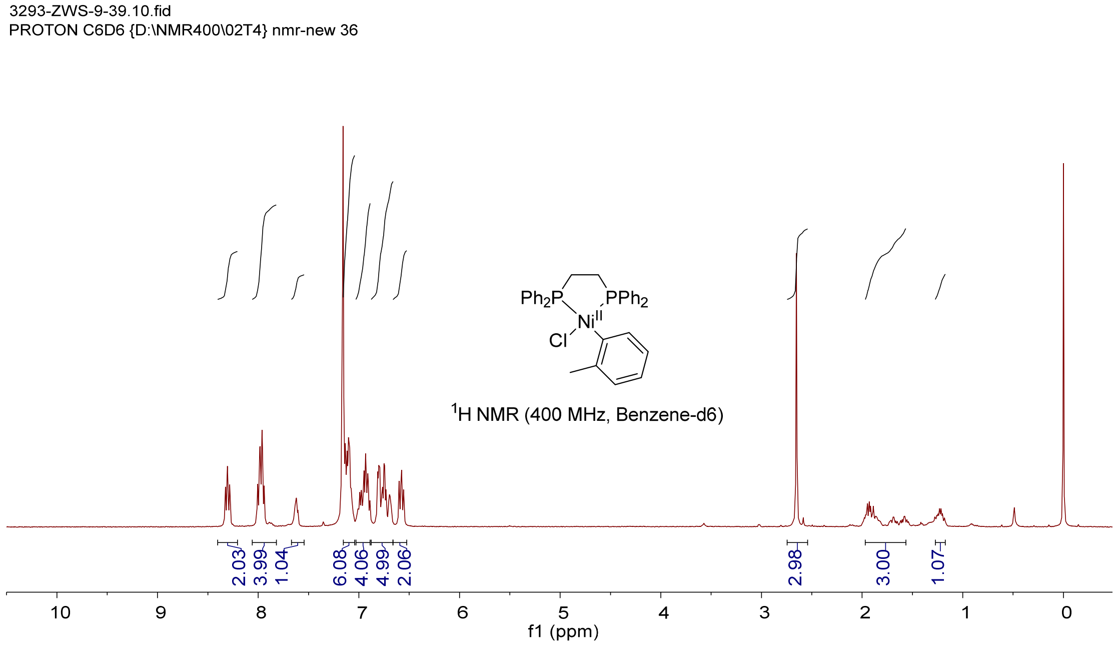

Supplementary Figure 173. ^{1}H NMR of Ni(2-MeC$_6$H$_4$)Cl(dppe)

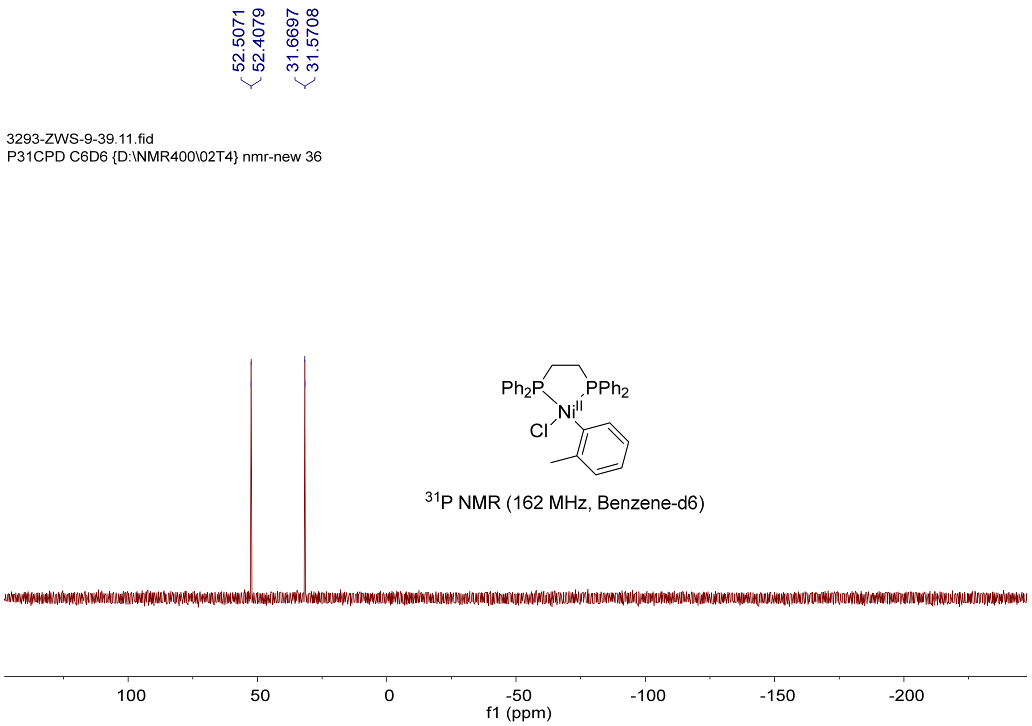

Supplementary Figure 174. ^{31}P NMR of Ni(2-MeC$_6$H$_4$)Cl(dppe)

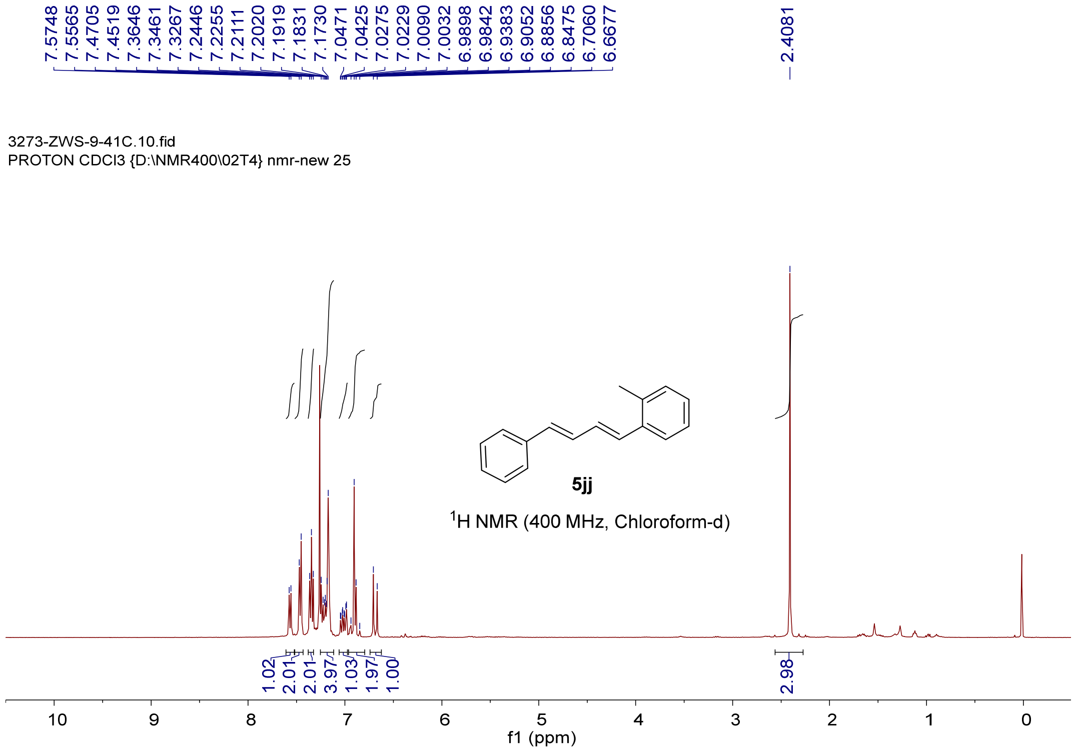

Supplementary Figure 175. ^{1}H NMR of compound **5jj**

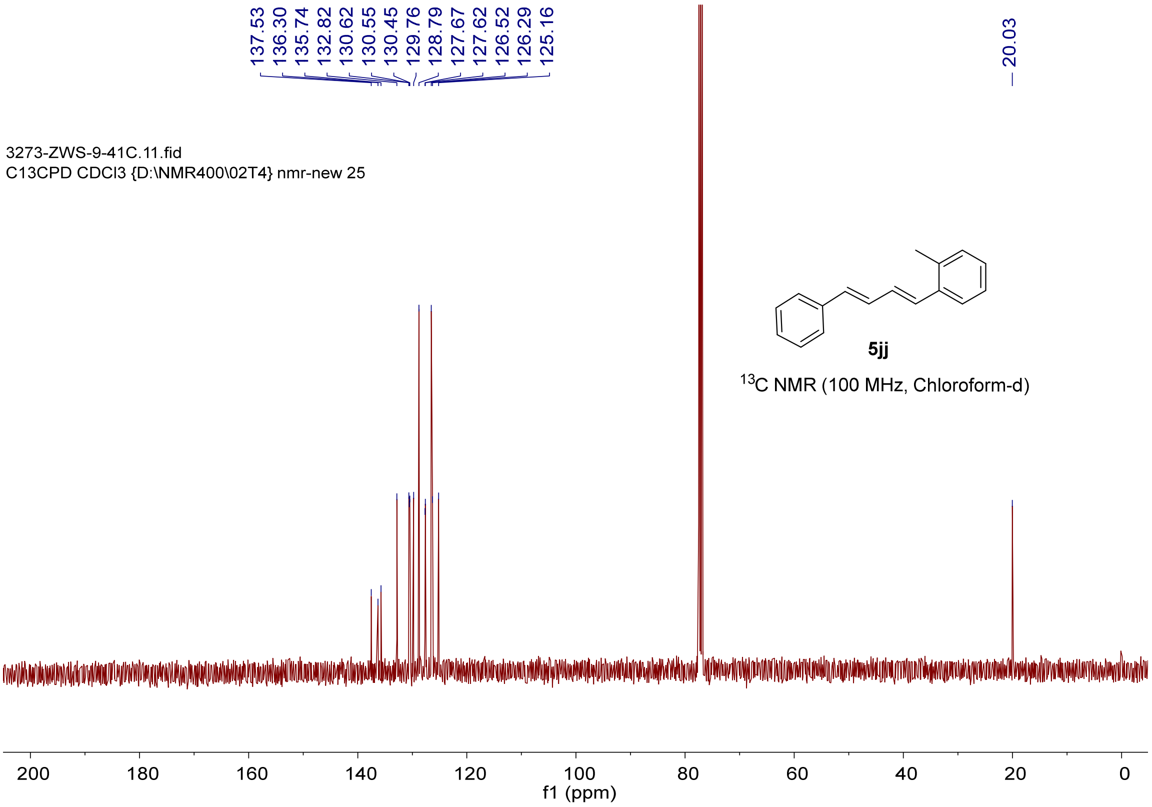

Supplementary Figure 176. ^{13}C NMR of compound **5jj**

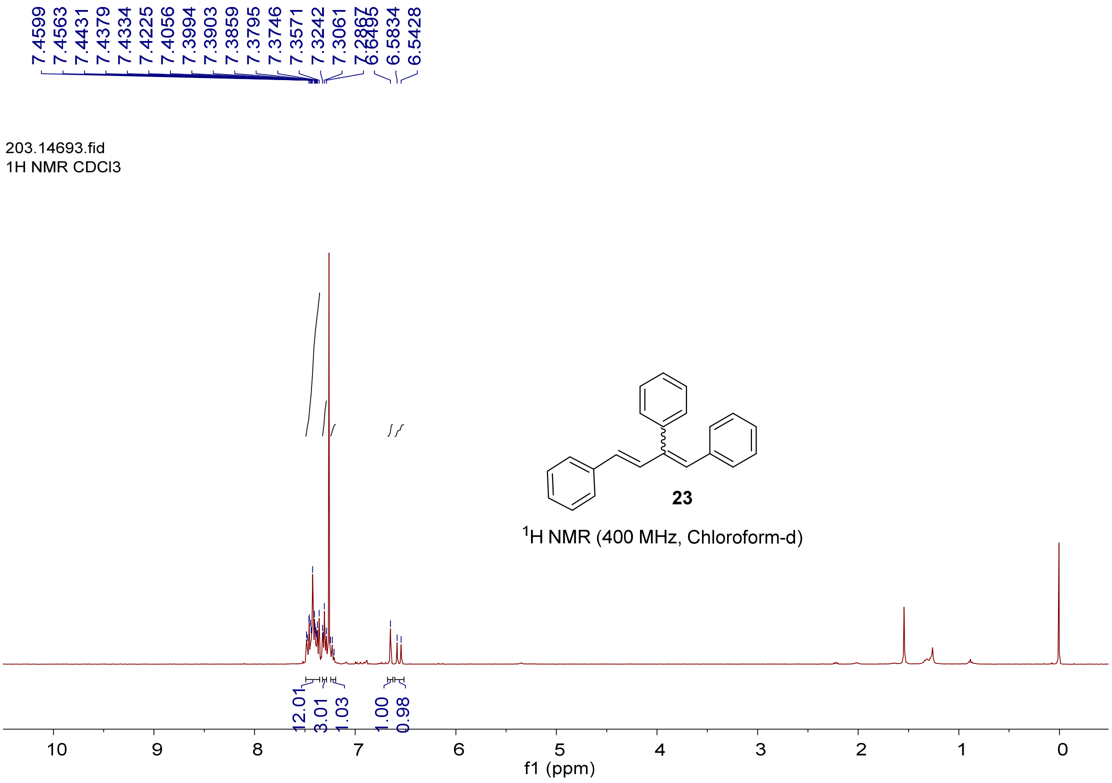

Supplementary Figure 177. ^{1}H NMR of compound **23**

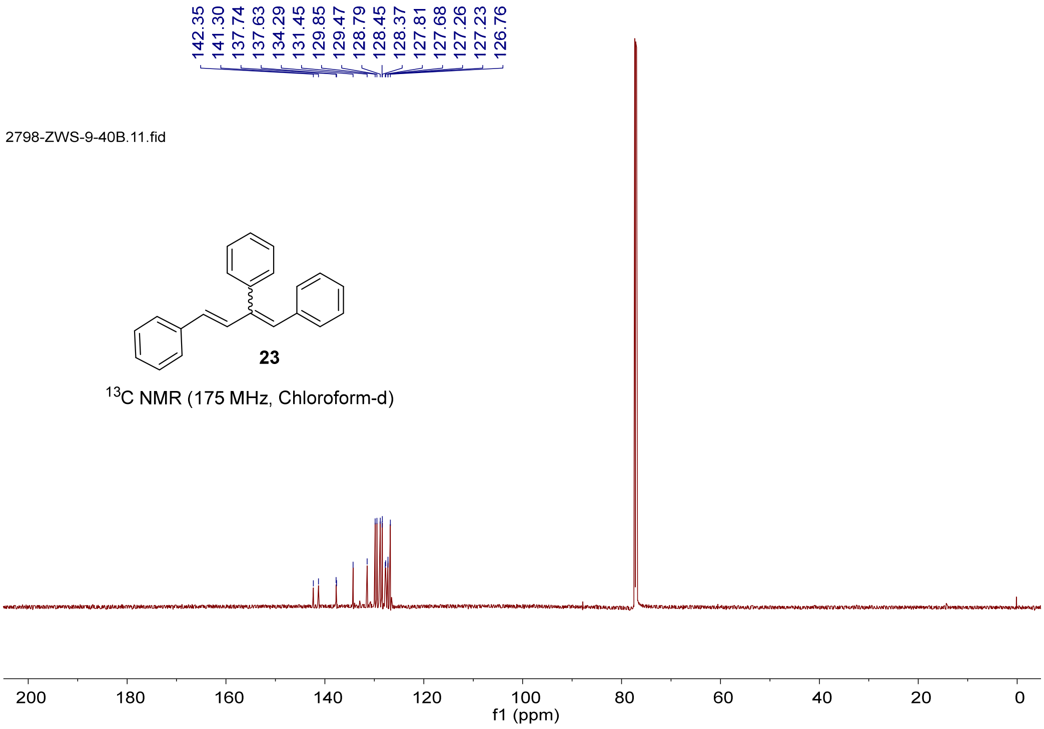

Supplementary Figure 178. ^{13}C NMR of compound **23**

5. Supplementary References

1 Standley, E. A., Smith, S. J., Muller, P. & Jamison, T. F. A broadly applicable strategy for entry into homogeneous nickel(0) catalysts from air-stable nickel(II) complexes. *Organometallics* **33**, 2012-2018 (2014).

2 Bhowmik, A. & Fernandes, R. A. Iron(III)/O_2-mediated regioselective oxidative cleavage of 1-arylbutadienes to cinnamaldehydes. *Org. Lett.* **21**, 9203-9207 (2019).

3 Denmark, S. E. & Tymonko, S. A. Sequential cross-coupling of 1,4-bissilylbutadienes: Synthesis of unsymmetrical 1,4-disubstituted 1,3-butadienes. *J. Am. Chem. Soc.* **127**, 8004-8005 (2005).

4 Ji, D.-W. *et al.* Nickel-catalyzed allyl-allyl coupling reactions between 1,3-dienes and allylboronates. *Chem. Commun.* **56**, 7431-7434 (2020).

5 Kojima, R. *et al.* Regioselective 1,4-addition of ammonia to 1-arylalka-1,3-dienes and 1-aryl-4-phenylbuta-1,3-dienes by photoinduced electron transfer. *J Chem Soc Perk T 1*, 217-222 (1997).

6 Huang, F., Huang, Z., Liu, G. & Huang, Z. Iridium-catalyzed selective trans-semihydrogenation of 1,3-enynes with ethanol: access to (*E,E*)-1,4-diarylbutadienes. *Org. Lett.* **24**, 5486-5490 (2022).

7 Zhang, N., Quan, Z.-J., Zhang, Z., Da, Y.-X. & Wang, X.-C. Synthesis of stilbene derivatives via visible-light-induced cross-coupling of aryl diazonium salts with nitroalkenes using -NO_2 as a leaving group. *Chem. Commun.* **52**, 14234-14237 (2016).

8 Thiel, N. O., Kemper, S. & Teichert, J. F. Copper(I)-catalyzed stereoselective hydrogenation of 1,3-diynes and enynes. *Tetrahedron* **73**, 5023-5028 (2017).

9 Leznoff, C. C. & Hayward, R. J. Photocyclization reactions of aryl polyenes. The photocychzation of 1-substitutedphenyl-4-phenyl-1,3-butadienes. *Can. J. Chem.* **48**, 1842-1849 (1970).

10 Wang, J.-X. *et al.* Palladium-catalyzed stereoselective synthesis of (*E*)-stilbenes via organozinc reagents and carbonyl compounds. *Adv. Synth. Catal.* **348**, 1262-1270 (2006).

11 Singh, A. K., Darshi, M. & Kanvah, S. α,ω-Diphenylpolyenes cabable of exhibiting twisted intramolecular charge transfer fluorescence: A fluorescence and fluorescence probe study of nitro- and nitrocyano-substituted 1,4-diphenylbutadienes. *J. Phy. Chem. A* **104**, 464-471 (2000).

12 Zhu, Q. & Nocera, D. G. Photocatalytic hydromethylation and hydroalkylation of olefins enabled by titanium dioxide mediated decarboxylation. *J. Am. Chem. Soc.* **142**, 17913-17918 (2020).

13 Nakabayashi, K., Arimura, J., Yasuda, M., Shima, K. & Takamuku, S. Cycloreversion of 1-(1-naphthyl)-5-phenylbicyclo 3.2.0 hept-6-ene to 1-(1-naphthyl)-4-phenyl-1,3-cycloheptadiene by triplet sensitization and direct irradiation. *Bull. Chem. Soc. Jpn.* **69**, 1313-1317 (1996).

14 Du, L. *et al.* C Cascade 8pi electrocyclization/benzannulation to access highly substituted phenylpyridines. *Org. Lett.* **23**, 7966-7971 (2021).

15 Gordillo, A. *et al.* Mechanistic studies on the Pd-catalyzed vinylation of aryl halides with vinylalkoxysilanes in water: the effect of the solvent and NaOH promoter. *J. Am. Chem. Soc.* **135**, 13749-13763 (2013).

16 Santelli, M. *et al.* Palladium-tetraphosphine complex catalysed Heck reaction of vinyl bromides with alkenes: A powerful access to conjugated dienes. *Synthesis* **2008**, 1142-1152 (2008).

17 Zhang, M. *et al.* Lewis acid catalyzed ring‑opening reaction of cyclobutanones towards

conjugated enones. *Eur. J. Org. Chem.* **2021**, 6111-6114 (2021).

18 Ohashi, M., Takeda, I., Ikawa, M. & Ogoshi, S. Nickel-catalyzed dehydrogenative [4 + 2] cycloaddition of 1,3-dienes with nitriles. *J. Am. Chem. Soc.* **133**, 18018-18021 (2011).

19 Gao, S. *et al.* Accessing 1,3-dienes via palladium-catalyzed allylic alkylation of pronucleophiles with skipped enynes. *Org. Lett.* **19**, 4710-4713 (2017).

20 Chen, L. *et al.* Transition-metal-free sulfuration/annulation of alkenes: Economical access to thiophenes enabled by the cleavage of multiple C-H bonds. *Org. Lett.* **20**, 7392-7395 (2018).

21 Xiao, L. J. *et al.* Nickel(0)-catalyzed hydroarylation of styrenes and 1,3-dienes with organoboron compounds. *Angew. Chem. Int. Ed.* **57**, 461-464 (2018).

22 Gandeepan, P. & Cheng, C.-H. Pd-catalyzed π-chelation assisted ortho-C-H activation and annulation of allylarenes with internal alkynes. *Org. Lett.* **15**, 2084-2087 (2013).

23 Nguyen, H. N., Huang, X. H. & Buchwald, S. L. The first general palladium catalyst for the Suzuki-Miyaura and carbonyl enolate coupling of aryl arenesulfonates. *J. Am. Chem. Soc.* **125**, 11818-11819 (2003).

24 Kirchberg, S., Frohlich, R. & Studer, A. 1,2,3-trisubstituted indanes by highly diastereoselective palladium-catalyzed oxyarylation of indenes with arylboronic acids and nitroxides. *Angew. Chem. Int. Ed.* **49**, 6877-6880 (2010).

25 Endo, Y., Yoshimi, T., Ohta, K., Suzuki, T. & Ohta, S. Potent estrogen receptor ligands based on bisphenols with a globular hydrophobic core. *J. Med. Chem.* **48**, 3941-3944 (2005).

26 Cabri, W., Candiani, I., Bedeschi, A. & Santi, R. Palladium-catalyzed arylation of unsymmetrical olefins. Bidentate phosphine ligand controlled regioselectivity. *J. Org. Chem.* **57**, 3558-3563 (1992).

27 Zou, Y. *et al.* Selective arylation and vinylation at the alpha position of vinylarenes. *Chem. Eur. J.* **19**, 3504-3511 (2013).

28 Tasker, S. Z., Gutierrez, A. C. & Jamison, T. F. Nickel-catalyzed Mizoroki-Heck reaction of aryl sulfonates and chlorides with electronically unbiased terminal olefins: high selectivity for branched products. *Angew. Chem. Int. Ed.* **53**, 1858-1861 (2014).

29 Ehle, A. R., Zhou, Q. & Watson, M. P. Nickel(0)-catalyzed Heck cross-coupling via activation of aryl C-OPiv bonds. *Org. Lett.* **14**, 1202-1205 (2012).

30 Mannathan, S. & Cheng, C. H. Cobalt-catalyzed regio- and stereoselective intermolecular enyne coupling: an efficient route to 1,3-diene derivatives. *Chem. Commun.* **46**, 1923-1925 (2010).

31 Baljak, S. *et al.* Ring-opening polymerization of a strained [3]nickelocenophane: a route to polynickelocenes, a class of S = 1 metallopolymers. *J. Am. Chem. Soc.* **136**, 5864-5867 (2014).

32 Uttech, R. & Dietrich, H. Kristall- und molekulstruktur von bis-methallylnickel $Ni[(CH_2)_2CCH_3]_2$. *Z. Kristallogr. Bd.* **122**, 60-72 (1965).
